# Supplementary figures and images for: Deep convolutional neural networks for regular texture recognition (part 2 of 8)
Source: PeerJ Comput Sci. 2022 Feb 9;8:e869. doi: 10.7717/peerj-cs.869 (PMC9044313; doi:10.7717/peerj-cs.869)

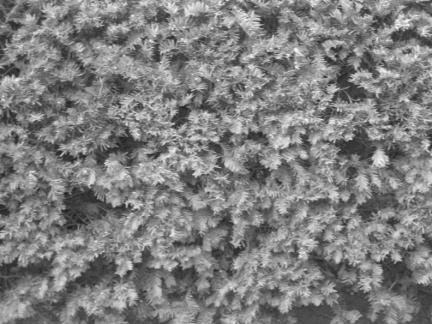

Supplement: Supplemental Information 1 [file peerj-cs-08-869-s001.zip › 0_part1/590_Flora31_11.jpg]

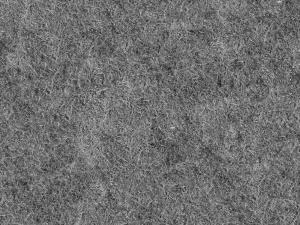

Supplement: Supplemental Information 1 [file peerj-cs-08-869-s001.zip › 0_part1/591_grass_grass_0077_01_thumb.jpg]

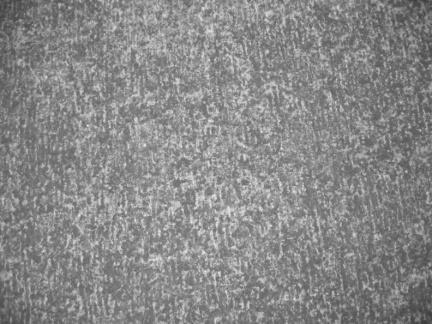

Supplement: Supplemental Information 1 [file peerj-cs-08-869-s001.zip › 0_part1/592_texture_14.jpg]

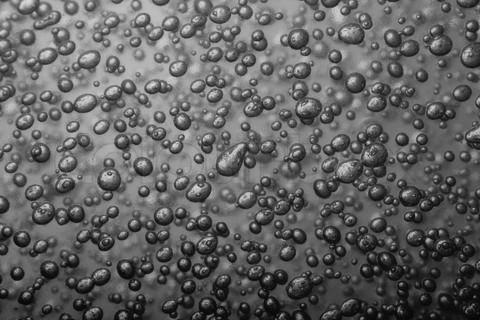

Supplement: Supplemental Information 1 [file peerj-cs-08-869-s001.zip › 0_part1/593_bubbly_0118.jpg]

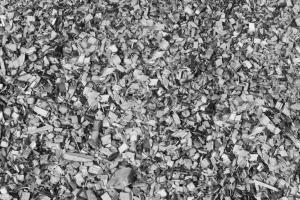

Supplement: Supplemental Information 1 [file peerj-cs-08-869-s001.zip › 0_part1/594_debris_wood_chips_0002_01_thumb.jpg]

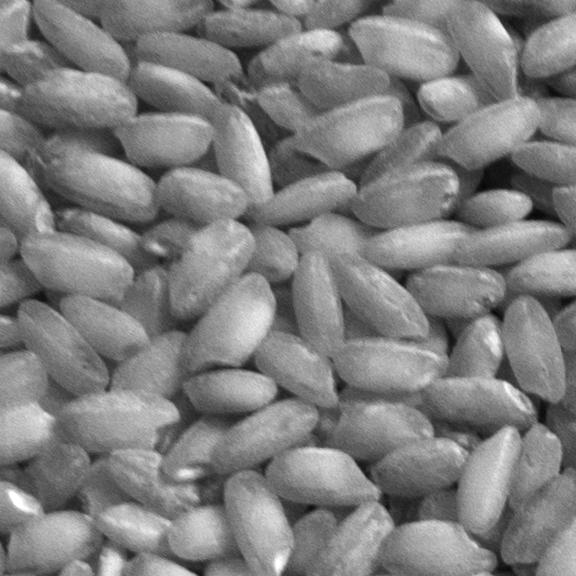

Supplement: Supplemental Information 1 [file peerj-cs-08-869-s001.zip › 0_part1/595_rice2-a-p002.jpg]

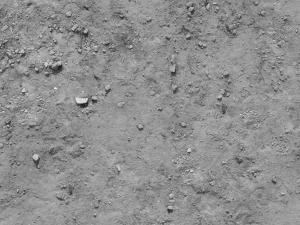

Supplement: Supplemental Information 1 [file peerj-cs-08-869-s001.zip › 0_part1/596_debris_stone_debris_0019_01_thumb.jpg]

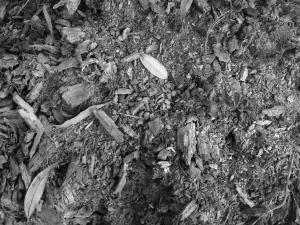

Supplement: Supplemental Information 1 [file peerj-cs-08-869-s001.zip › 0_part1/597_wood_rotten_0017_01_thumb.jpg]

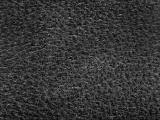

Supplement: Supplemental Information 1 [file peerj-cs-08-869-s001.zip › 0_part1/598_S_S_D92_Pigskin.jpg]

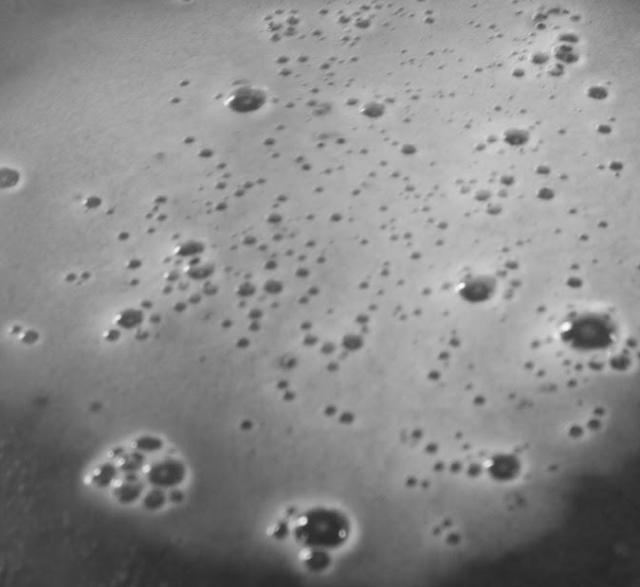

Supplement: Supplemental Information 1 [file peerj-cs-08-869-s001.zip › 0_part1/599_bubbly_0103.jpg]

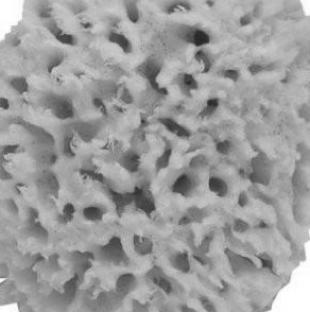

Supplement: Supplemental Information 1 [file peerj-cs-08-869-s001.zip › 0_part1/600_porous_0140.jpg]

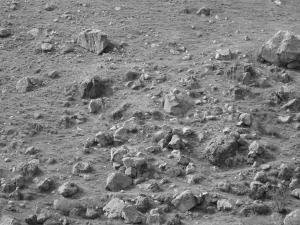

Supplement: Supplemental Information 1 [file peerj-cs-08-869-s001.zip › 0_part1/601_ground_slope_0056_01_thumb.jpg]

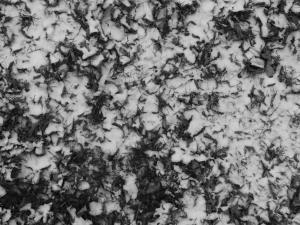

Supplement: Supplemental Information 1 [file peerj-cs-08-869-s001.zip › 0_part1/602_ground_frozen_ground_0036_01_thumb.jpg]

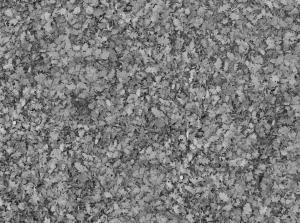

Supplement: Supplemental Information 1 [file peerj-cs-08-869-s001.zip › 0_part1/603_ground_ground_leaves_0019_01_thumb.jpg]

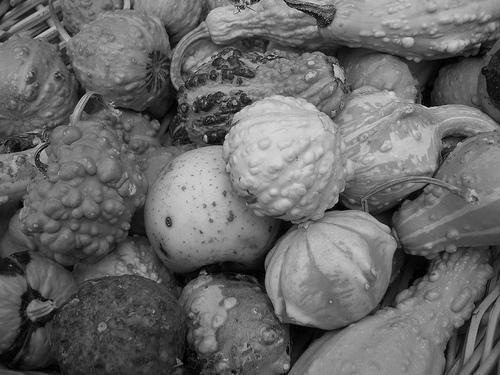

Supplement: Supplemental Information 1 [file peerj-cs-08-869-s001.zip › 0_part1/604_bumpy_0203.jpg]

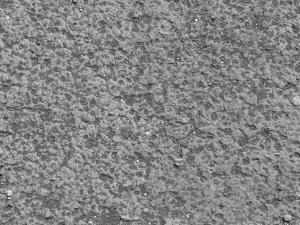

Supplement: Supplemental Information 1 [file peerj-cs-08-869-s001.zip › 0_part1/605_ground_stone_ground_0065_01_thumb.jpg]

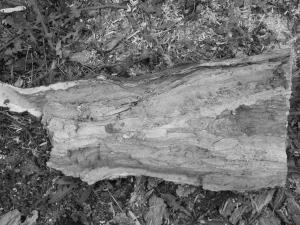

Supplement: Supplemental Information 1 [file peerj-cs-08-869-s001.zip › 0_part1/606_wood_damaged_0007_01_thumb.jpg]

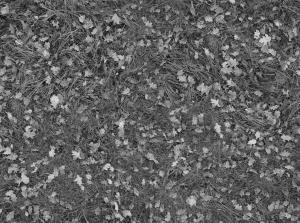

Supplement: Supplemental Information 1 [file peerj-cs-08-869-s001.zip › 0_part1/607_grass_leaves_0010_01_thumb.jpg]

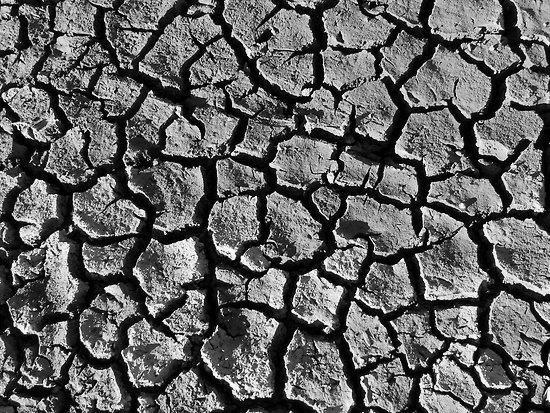

Supplement: Supplemental Information 1 [file peerj-cs-08-869-s001.zip › 0_part1/608_cracked_0162.jpg]

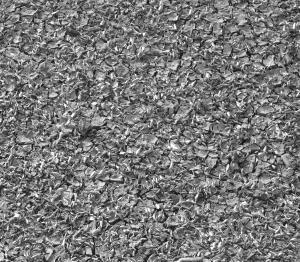

Supplement: Supplemental Information 1 [file peerj-cs-08-869-s001.zip › 0_part1/609_ground_ground_leaves_0037_01_thumb.jpg]

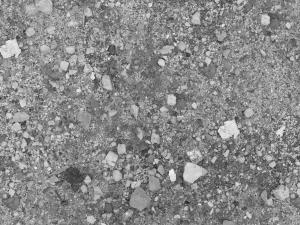

Supplement: Supplemental Information 1 [file peerj-cs-08-869-s001.zip › 0_part1/610_debris_stone_debris_0021_01_thumb.jpg]

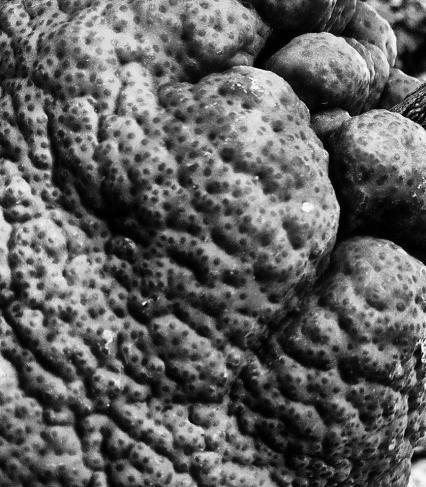

Supplement: Supplemental Information 1 [file peerj-cs-08-869-s001.zip › 0_part1/611_bumpy_0024.jpg]

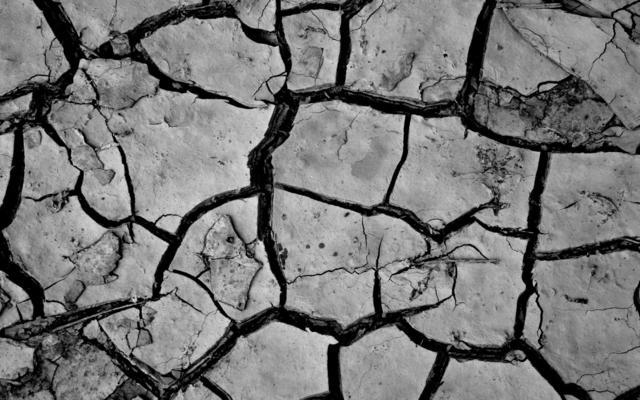

Supplement: Supplemental Information 1 [file peerj-cs-08-869-s001.zip › 0_part1/612_cracked_0098.jpg]

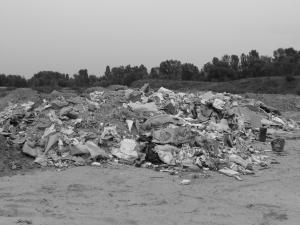

Supplement: Supplemental Information 1 [file peerj-cs-08-869-s001.zip › 0_part1/613_debris_garbage_0025_01_thumb.jpg]

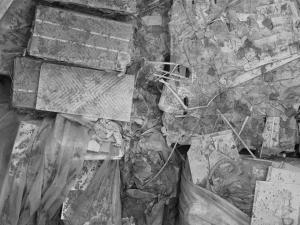

Supplement: Supplemental Information 1 [file peerj-cs-08-869-s001.zip › 0_part1/614_debris_garbage_0023_01_thumb.jpg]

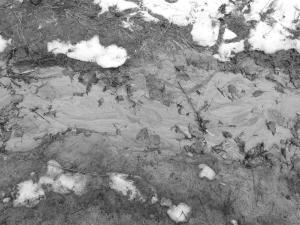

Supplement: Supplemental Information 1 [file peerj-cs-08-869-s001.zip › 0_part1/615_ground_other_ground_0002_01_thumb.jpg]

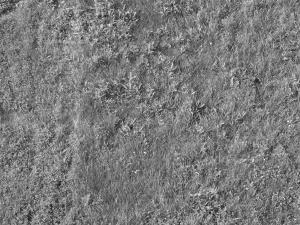

Supplement: Supplemental Information 1 [file peerj-cs-08-869-s001.zip › 0_part1/616_grass_grass_0094_01_thumb.jpg]

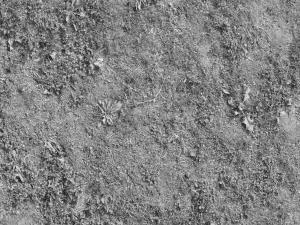

Supplement: Supplemental Information 1 [file peerj-cs-08-869-s001.zip › 0_part1/617_grass_grass_0069_01_thumb.jpg]

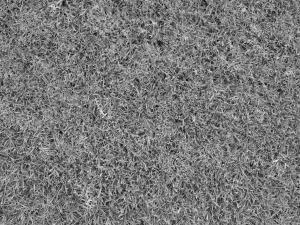

Supplement: Supplemental Information 1 [file peerj-cs-08-869-s001.zip › 0_part1/618_grass_grass_0110_01_thumb.jpg]

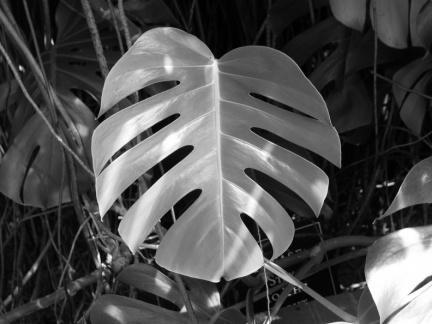

Supplement: Supplemental Information 1 [file peerj-cs-08-869-s001.zip › 0_part1/619_Flora31_68.jpg]

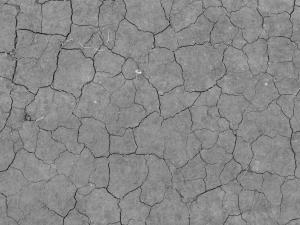

Supplement: Supplemental Information 1 [file peerj-cs-08-869-s001.zip › 0_part1/620_soil_cracked_0033_01_thumb.jpg]

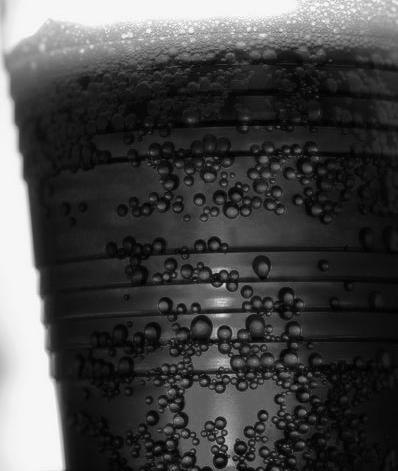

Supplement: Supplemental Information 1 [file peerj-cs-08-869-s001.zip › 0_part1/621_bubbly_0137.jpg]

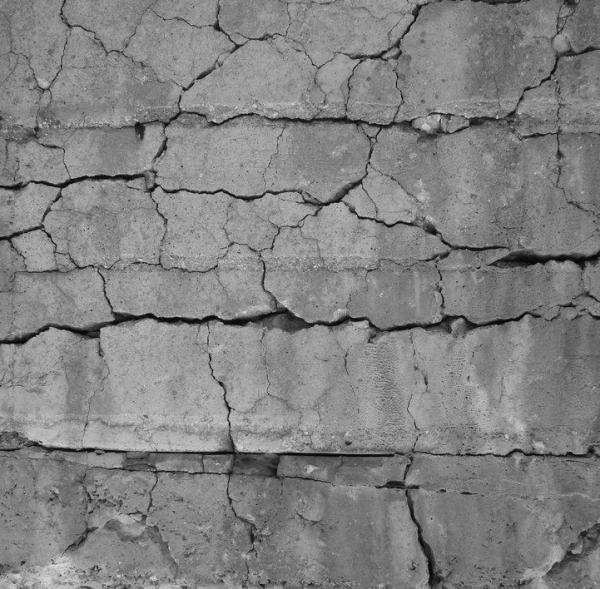

Supplement: Supplemental Information 1 [file peerj-cs-08-869-s001.zip › 0_part1/622_cracked_0136.jpg]

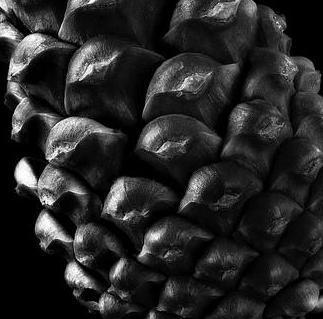

Supplement: Supplemental Information 1 [file peerj-cs-08-869-s001.zip › 0_part1/623_bumpy_0181.jpg]

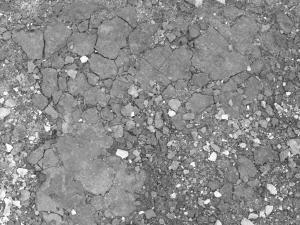

Supplement: Supplemental Information 1 [file peerj-cs-08-869-s001.zip › 0_part1/624_debris_metal_debris_0005_01_thumb.jpg]

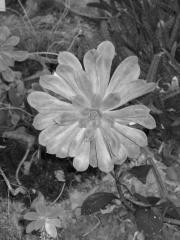

Supplement: Supplemental Information 1 [file peerj-cs-08-869-s001.zip › 0_part1/625_Flora31_49.jpg]

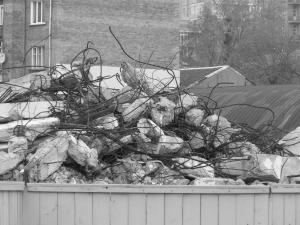

Supplement: Supplemental Information 1 [file peerj-cs-08-869-s001.zip › 0_part1/626_debris_other_0002_01_thumb.jpg]

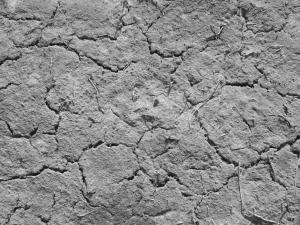

Supplement: Supplemental Information 1 [file peerj-cs-08-869-s001.zip › 0_part1/627_soil_cracked_0041_01_thumb.jpg]

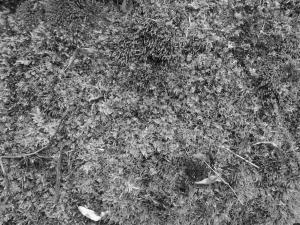

Supplement: Supplemental Information 1 [file peerj-cs-08-869-s001.zip › 0_part1/628_nature_moss_0007_01_thumb.jpg]

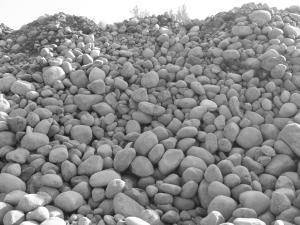

Supplement: Supplemental Information 1 [file peerj-cs-08-869-s001.zip › 0_part1/629_debris_stone_debris_0042_01_thumb.jpg]

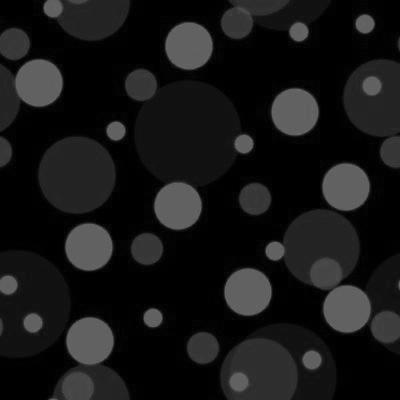

Supplement: Supplemental Information 1 [file peerj-cs-08-869-s001.zip › 0_part1/630_dotted_0186.jpg]

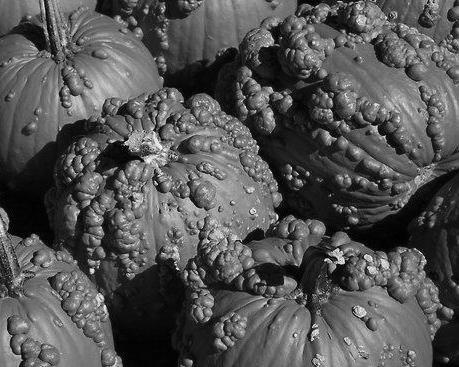

Supplement: Supplemental Information 1 [file peerj-cs-08-869-s001.zip › 0_part1/631_bumpy_0156.jpg]

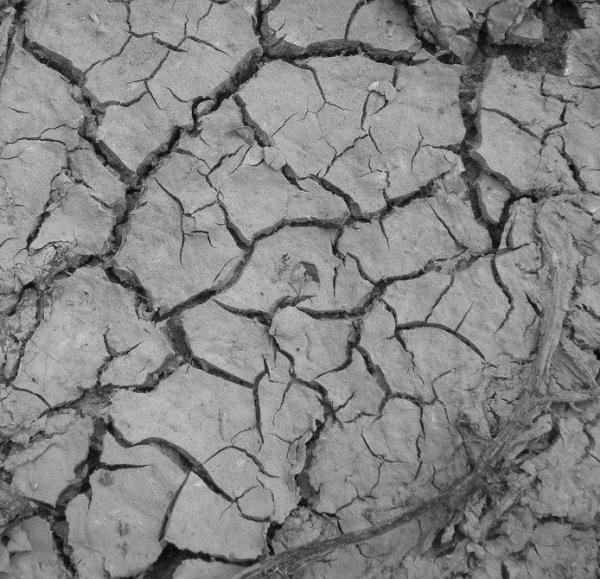

Supplement: Supplemental Information 1 [file peerj-cs-08-869-s001.zip › 0_part1/632_cracked_0106.jpg]

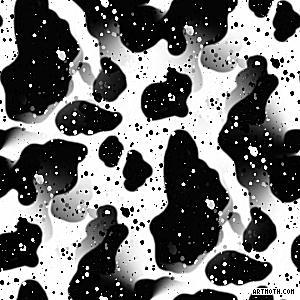

Supplement: Supplemental Information 1 [file peerj-cs-08-869-s001.zip › 0_part1/633_flecked_0055.jpg]

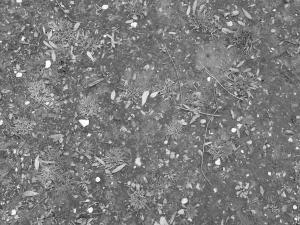

Supplement: Supplemental Information 1 [file peerj-cs-08-869-s001.zip › 0_part1/634_grass_other_grass_0035_01_thumb.jpg]

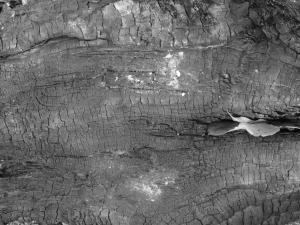

Supplement: Supplemental Information 1 [file peerj-cs-08-869-s001.zip › 0_part1/635_wood_burnt_0003_01_thumb.jpg]

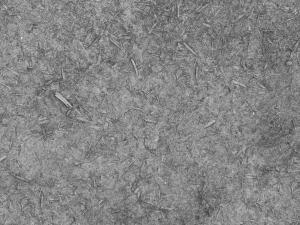

Supplement: Supplemental Information 1 [file peerj-cs-08-869-s001.zip › 0_part1/636_debris_wood_chips_0019_01_thumb.jpg]

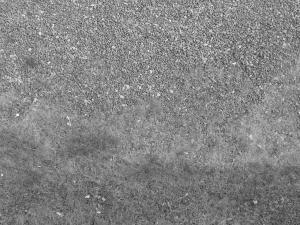

Supplement: Supplemental Information 1 [file peerj-cs-08-869-s001.zip › 0_part1/637_grass_on_stones_0014_01_thumb.jpg]

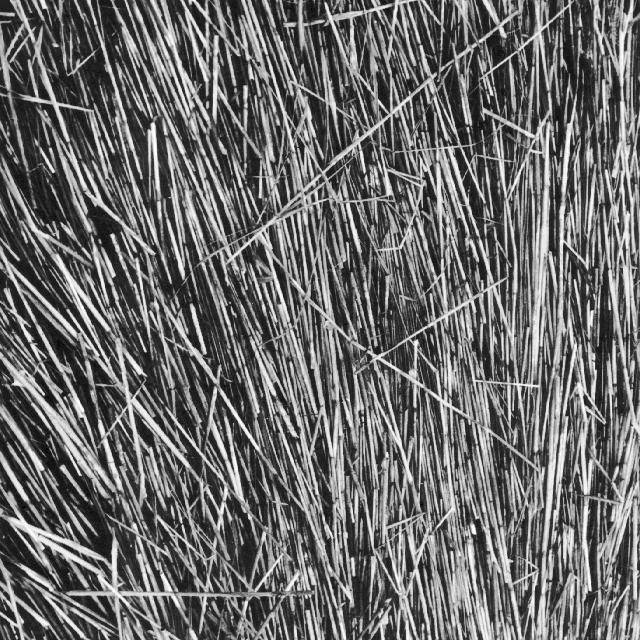

Supplement: Supplemental Information 1 [file peerj-cs-08-869-s001.zip › 0_part1/638_D15.jpg]

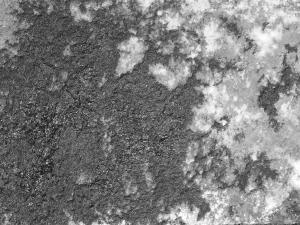

Supplement: Supplemental Information 1 [file peerj-cs-08-869-s001.zip › 0_part1/639_nature_moss_0047_01_thumb.jpg]

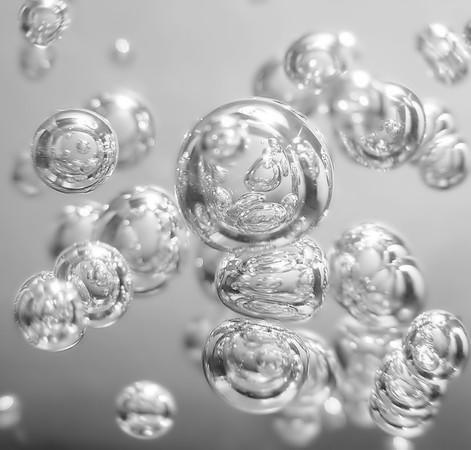

Supplement: Supplemental Information 1 [file peerj-cs-08-869-s001.zip › 0_part1/640_bubbly_0116.jpg]

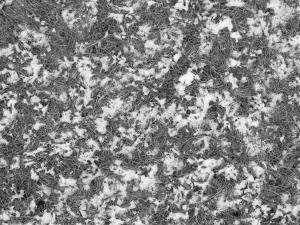

Supplement: Supplemental Information 1 [file peerj-cs-08-869-s001.zip › 0_part1/641_ground_frozen_ground_0029_01_thumb.jpg]

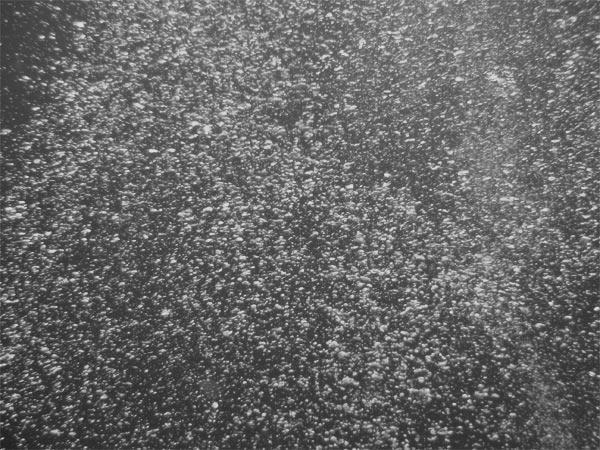

Supplement: Supplemental Information 1 [file peerj-cs-08-869-s001.zip › 0_part1/642_bubbly_0171.jpg]

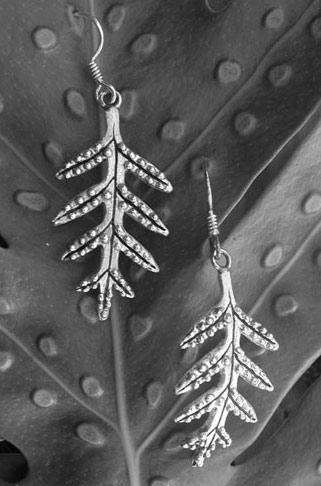

Supplement: Supplemental Information 1 [file peerj-cs-08-869-s001.zip › 0_part1/643_bumpy_0081.jpg]

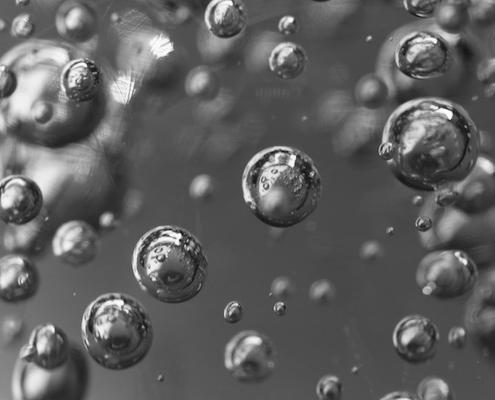

Supplement: Supplemental Information 1 [file peerj-cs-08-869-s001.zip › 0_part1/644_bubbly_0073.jpg]

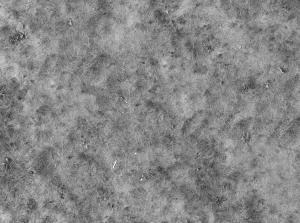

Supplement: Supplemental Information 1 [file peerj-cs-08-869-s001.zip › 0_part1/645_grass_grass_0008_01_thumb.jpg]

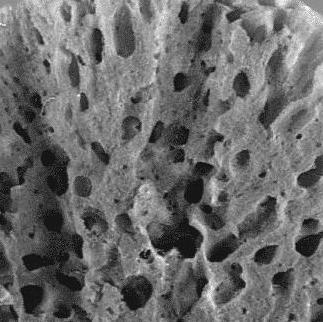

Supplement: Supplemental Information 1 [file peerj-cs-08-869-s001.zip › 0_part1/646_porous_0174.jpg]

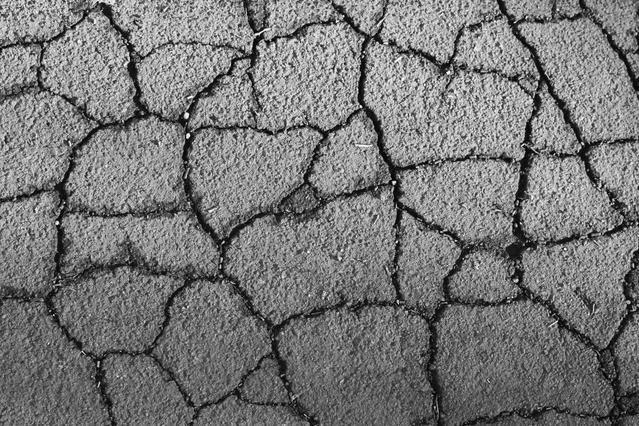

Supplement: Supplemental Information 1 [file peerj-cs-08-869-s001.zip › 0_part1/647_cracked_0066.jpg]

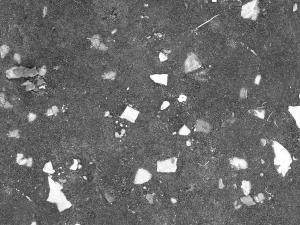

Supplement: Supplemental Information 1 [file peerj-cs-08-869-s001.zip › 0_part1/648_nature_moss_0021_01_thumb.jpg]

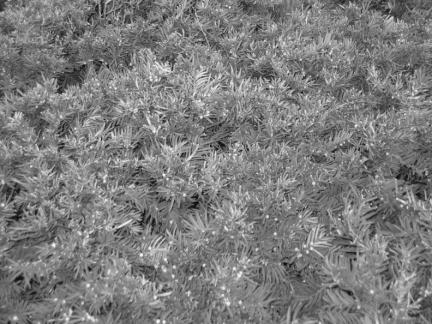

Supplement: Supplemental Information 1 [file peerj-cs-08-869-s001.zip › 0_part1/649_Flora31_1.jpg]

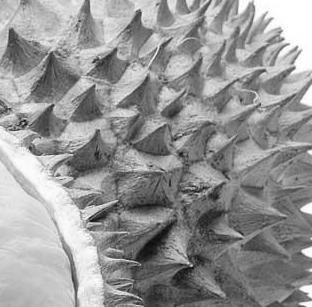

Supplement: Supplemental Information 1 [file peerj-cs-08-869-s001.zip › 0_part1/650_bumpy_0173.jpg]

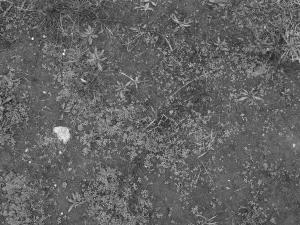

Supplement: Supplemental Information 1 [file peerj-cs-08-869-s001.zip › 0_part1/651_grass_grass_0024_01_thumb.jpg]

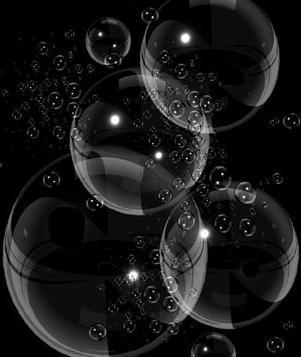

Supplement: Supplemental Information 1 [file peerj-cs-08-869-s001.zip › 0_part1/652_bubbly_0071.jpg]

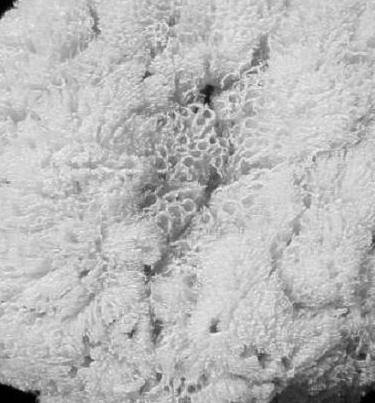

Supplement: Supplemental Information 1 [file peerj-cs-08-869-s001.zip › 0_part1/653_porous_0105.jpg]

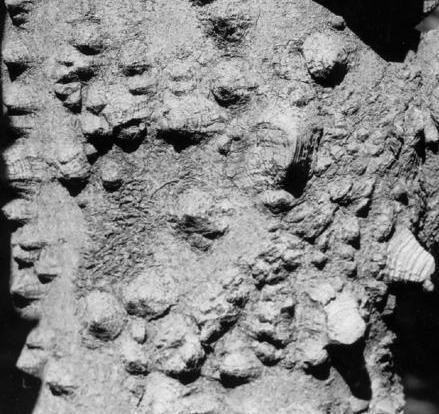

Supplement: Supplemental Information 1 [file peerj-cs-08-869-s001.zip › 0_part1/654_bumpy_0135.jpg]

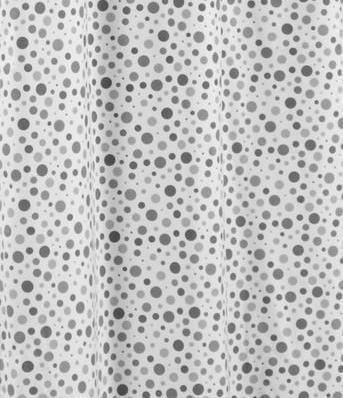

Supplement: Supplemental Information 1 [file peerj-cs-08-869-s001.zip › 0_part1/655_dotted_0165.jpg]

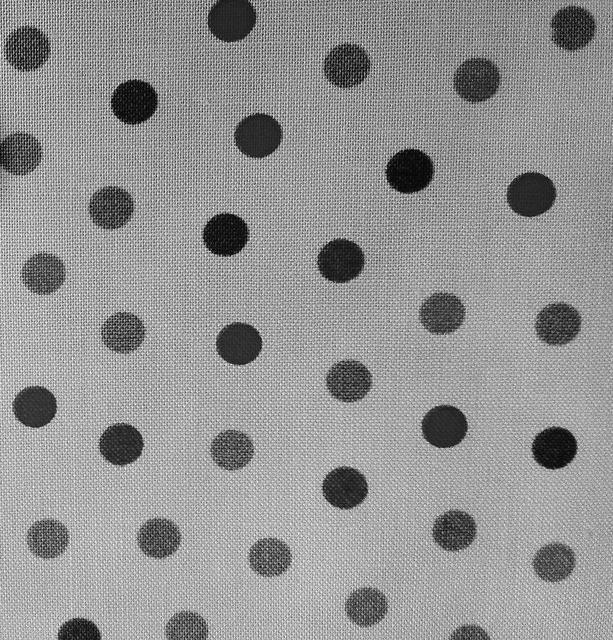

Supplement: Supplemental Information 1 [file peerj-cs-08-869-s001.zip › 0_part1/656_dotted_0163.jpg]

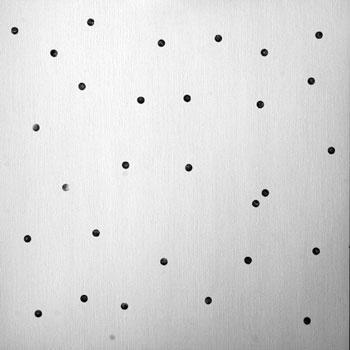

Supplement: Supplemental Information 1 [file peerj-cs-08-869-s001.zip › 0_part1/657_dotted_0189.jpg]

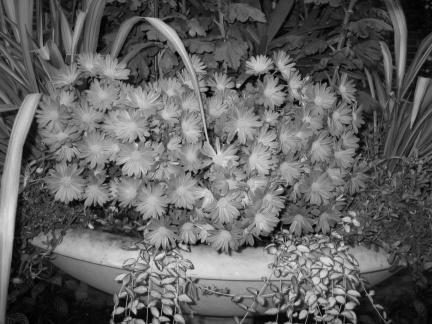

Supplement: Supplemental Information 1 [file peerj-cs-08-869-s001.zip › 0_part1/658_Flora31_24.jpg]

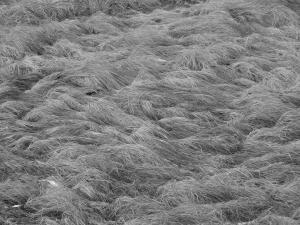

Supplement: Supplemental Information 1 [file peerj-cs-08-869-s001.zip › 0_part1/659_grass_grass_0020_01_thumb.jpg]

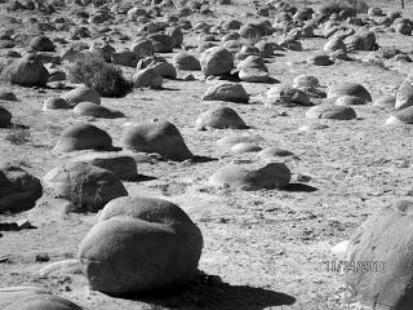

Supplement: Supplemental Information 1 [file peerj-cs-08-869-s001.zip › 0_part1/660_bumpy_0153.jpg]

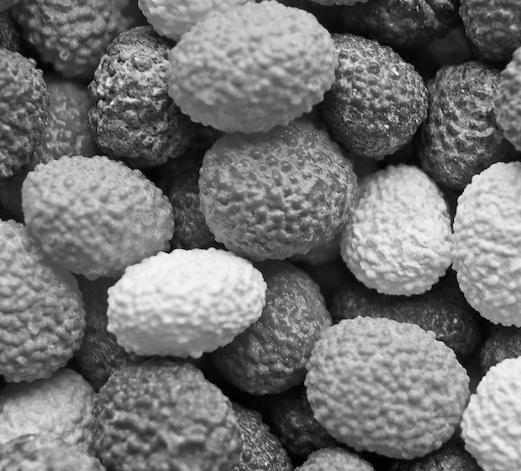

Supplement: Supplemental Information 1 [file peerj-cs-08-869-s001.zip › 0_part1/661_bumpy_0180.jpg]

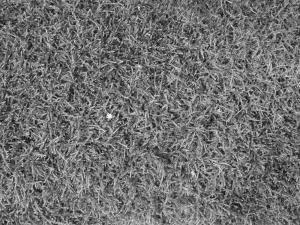

Supplement: Supplemental Information 1 [file peerj-cs-08-869-s001.zip › 0_part1/662_nature_moss_0042_01_thumb.jpg]

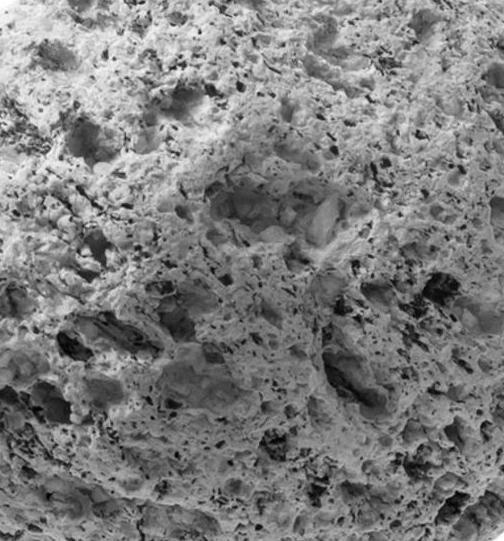

Supplement: Supplemental Information 1 [file peerj-cs-08-869-s001.zip › 0_part1/663_porous_0173.jpg]

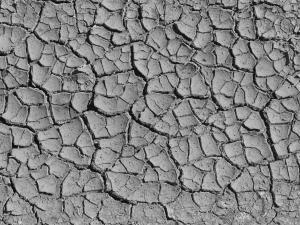

Supplement: Supplemental Information 1 [file peerj-cs-08-869-s001.zip › 0_part1/664_soil_cracked_0037_01_thumb.jpg]

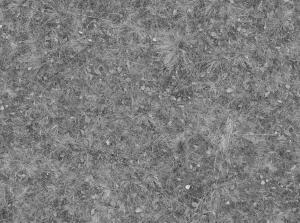

Supplement: Supplemental Information 1 [file peerj-cs-08-869-s001.zip › 0_part1/665_grass_leaves_0019_01_thumb.jpg]

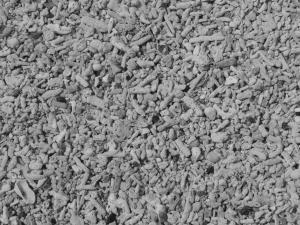

Supplement: Supplemental Information 1 [file peerj-cs-08-869-s001.zip › 0_part1/666_ground_pebble_0051_01_thumb.jpg]

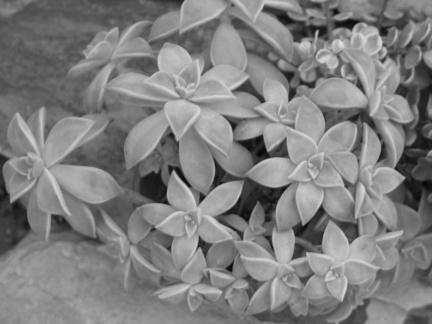

Supplement: Supplemental Information 1 [file peerj-cs-08-869-s001.zip › 0_part1/667_Flora31_30.jpg]

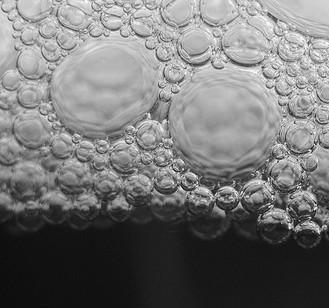

Supplement: Supplemental Information 1 [file peerj-cs-08-869-s001.zip › 0_part1/668_bubbly_0164.jpg]

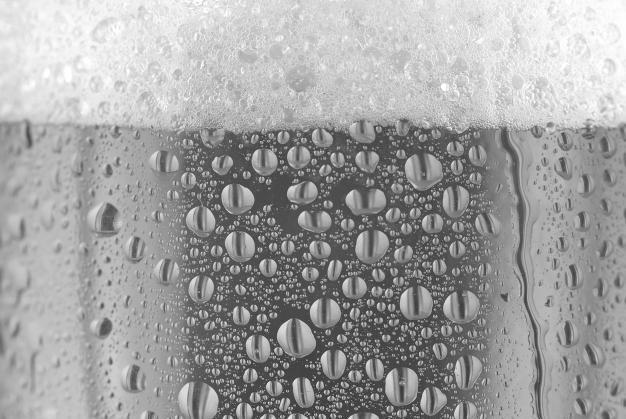

Supplement: Supplemental Information 1 [file peerj-cs-08-869-s001.zip › 0_part1/669_bubbly_0120.jpg]

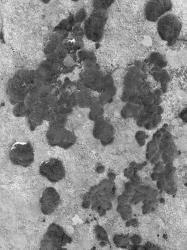

Supplement: Supplemental Information 1 [file peerj-cs-08-869-s001.zip › 0_part1/670_nature_moss_0034_01_thumb.jpg]

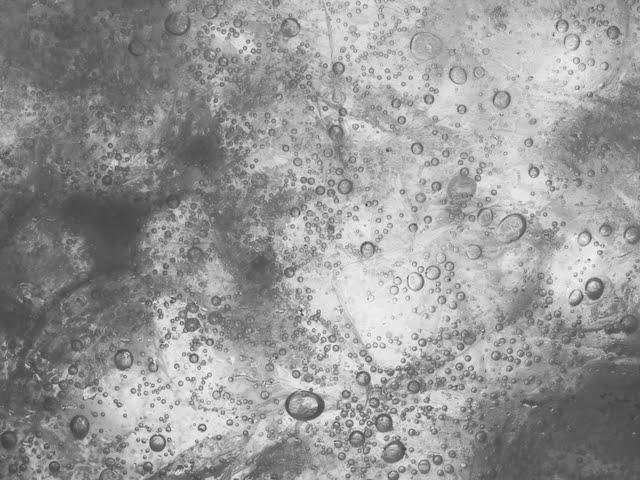

Supplement: Supplemental Information 1 [file peerj-cs-08-869-s001.zip › 0_part1/671_bubbly_0122.jpg]

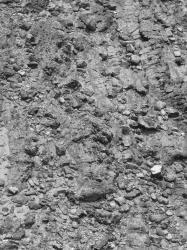

Supplement: Supplemental Information 1 [file peerj-cs-08-869-s001.zip › 0_part1/672_ground_stone_ground_0054_01_thumb.jpg]

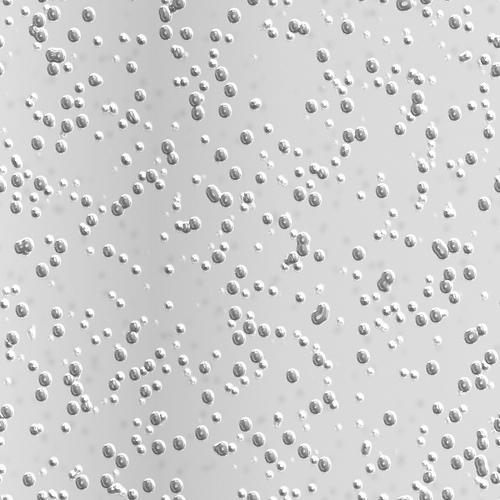

Supplement: Supplemental Information 1 [file peerj-cs-08-869-s001.zip › 0_part1/673_bubbly_0055.jpg]

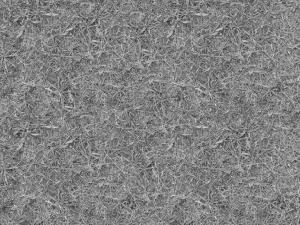

Supplement: Supplemental Information 1 [file peerj-cs-08-869-s001.zip › 0_part1/674_grass_grass_0057_01_thumb.jpg]

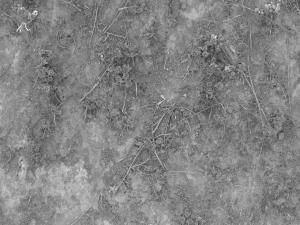

Supplement: Supplemental Information 1 [file peerj-cs-08-869-s001.zip › 0_part1/676_ground_other_ground_0011_01_thumb.jpg]

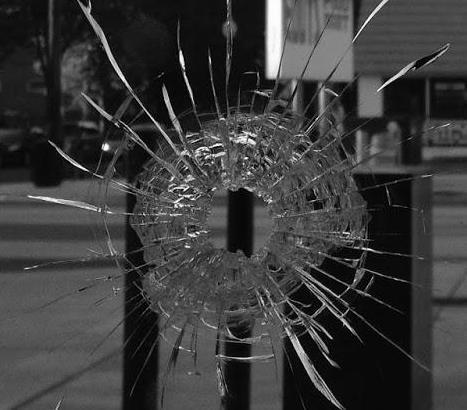

Supplement: Supplemental Information 1 [file peerj-cs-08-869-s001.zip › 0_part1/677_cracked_0164.jpg]

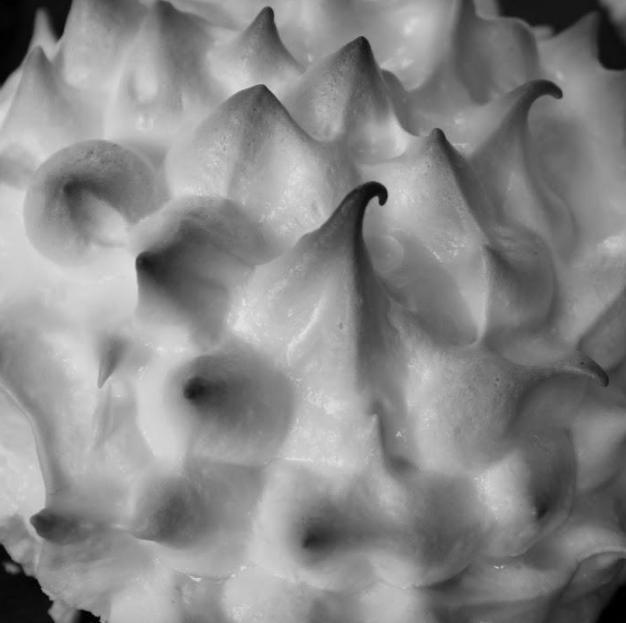

Supplement: Supplemental Information 1 [file peerj-cs-08-869-s001.zip › 0_part1/678_bumpy_0198.jpg]

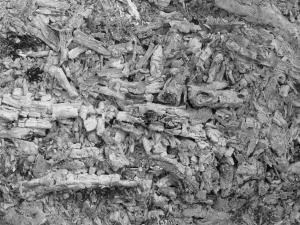

Supplement: Supplemental Information 1 [file peerj-cs-08-869-s001.zip › 0_part1/679_debris_wood_chips_0023_01_thumb.jpg]

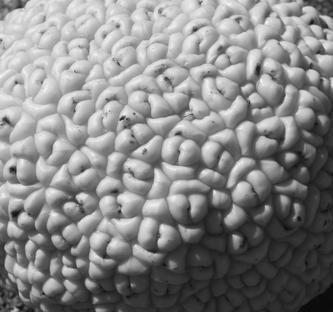

Supplement: Supplemental Information 1 [file peerj-cs-08-869-s001.zip › 0_part1/680_bumpy_0073.jpg]

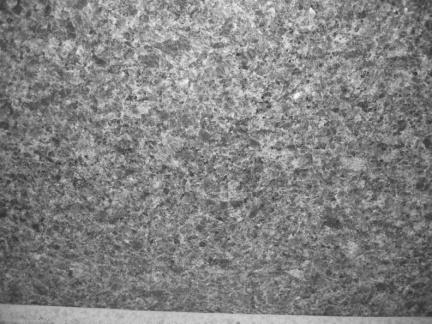

Supplement: Supplemental Information 1 [file peerj-cs-08-869-s001.zip › 0_part1/681_texture_17.jpg]

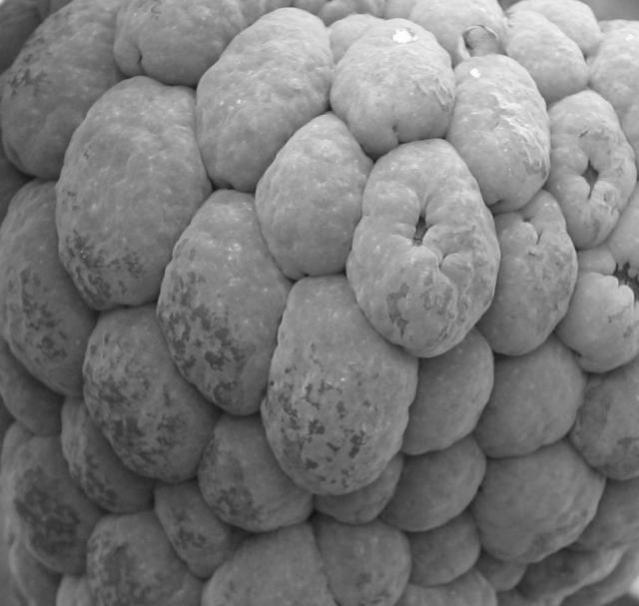

Supplement: Supplemental Information 1 [file peerj-cs-08-869-s001.zip › 0_part1/682_bumpy_0189.jpg]

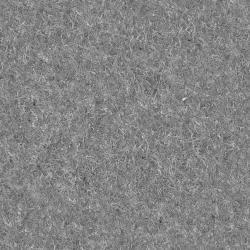

Supplement: Supplemental Information 1 [file peerj-cs-08-869-s001.zip › 0_part1/683_grass_grass_0103_01_thumb.jpg]

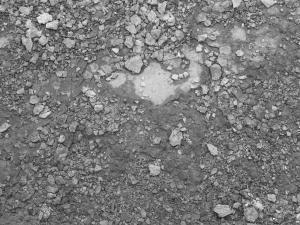

Supplement: Supplemental Information 1 [file peerj-cs-08-869-s001.zip › 0_part1/684_debris_stone_debris_0049_01_thumb.jpg]

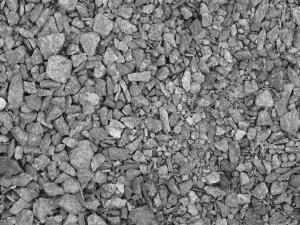

Supplement: Supplemental Information 1 [file peerj-cs-08-869-s001.zip › 0_part1/685_ground_pebble_0011_01_thumb.jpg]

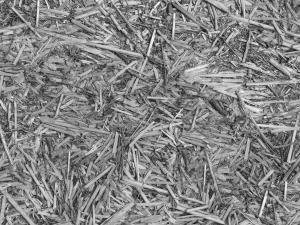

Supplement: Supplemental Information 1 [file peerj-cs-08-869-s001.zip › 0_part1/686_debris_wood_chips_0015_01_thumb.jpg]

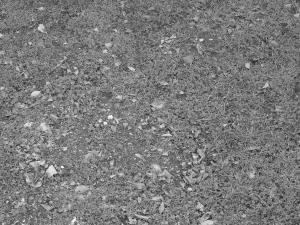

Supplement: Supplemental Information 1 [file peerj-cs-08-869-s001.zip › 0_part1/687_grass_leaves_0042_01_thumb.jpg]

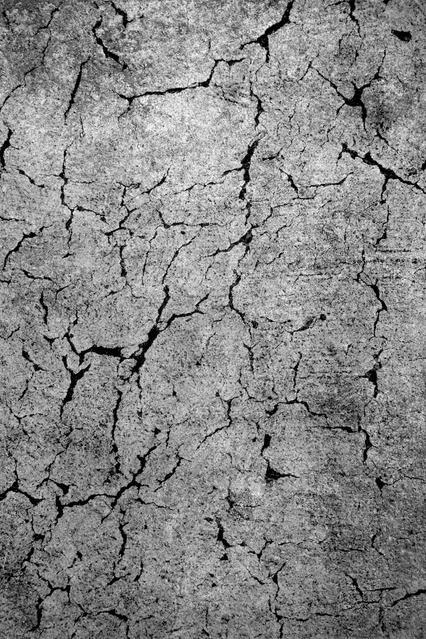

Supplement: Supplemental Information 1 [file peerj-cs-08-869-s001.zip › 0_part1/688_cracked_0053.jpg]

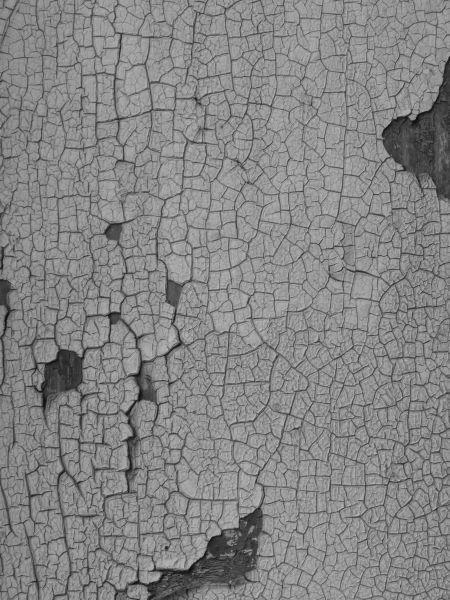

Supplement: Supplemental Information 1 [file peerj-cs-08-869-s001.zip › 0_part1/689_cracked_0161.jpg]

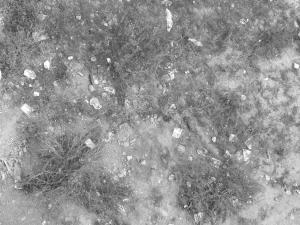

Supplement: Supplemental Information 2 [file peerj-cs-08-869-s002.zip › 0_part2/100_grass_other_grass_0016_01_thumb.jpg]
