# Supplementary figures and images for: Deep convolutional neural networks for regular texture recognition (part 1 of 8)
Source: PeerJ Comput Sci. 2022 Feb 9;8:e869. doi: 10.7717/peerj-cs.869 (PMC9044313; doi:10.7717/peerj-cs.869)

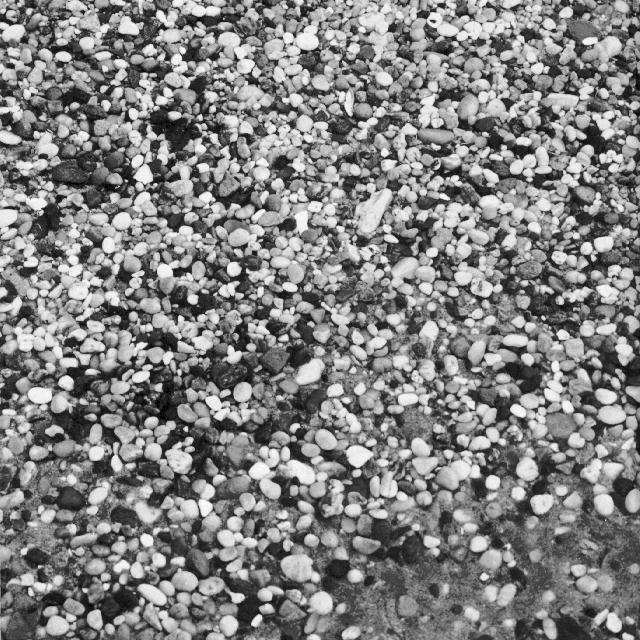

Supplement: Supplemental Information 1 [file peerj-cs-08-869-s001.zip › 0_part1/1000_D54.jpg]

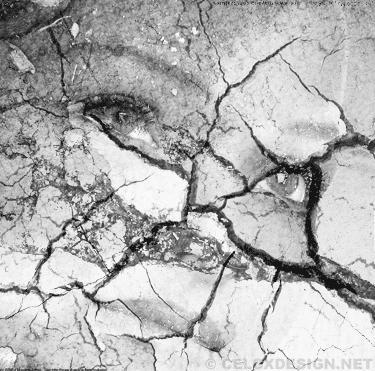

Supplement: Supplemental Information 1 [file peerj-cs-08-869-s001.zip › 0_part1/491_cracked_0064.jpg]

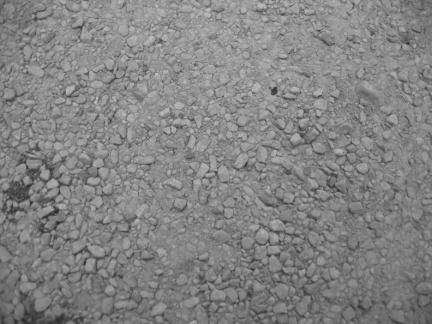

Supplement: Supplemental Information 1 [file peerj-cs-08-869-s001.zip › 0_part1/492_texture_22.jpg]

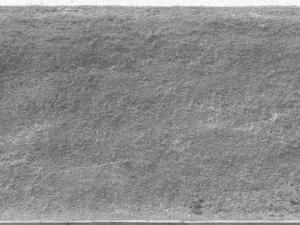

Supplement: Supplemental Information 1 [file peerj-cs-08-869-s001.zip › 0_part1/493_ground_slope_0029_01_thumb.jpg]

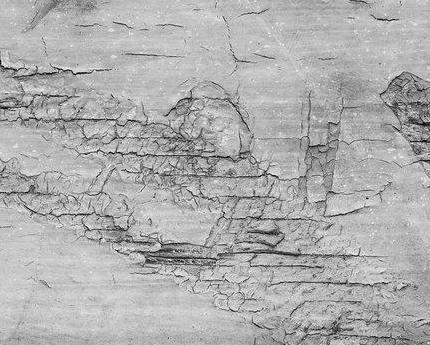

Supplement: Supplemental Information 1 [file peerj-cs-08-869-s001.zip › 0_part1/494_cracked_0103.jpg]

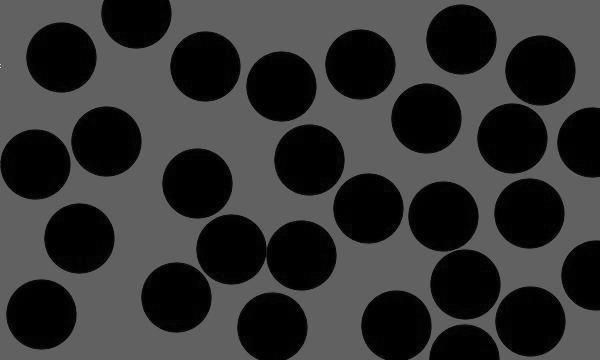

Supplement: Supplemental Information 1 [file peerj-cs-08-869-s001.zip › 0_part1/495_dotted_0122.jpg]

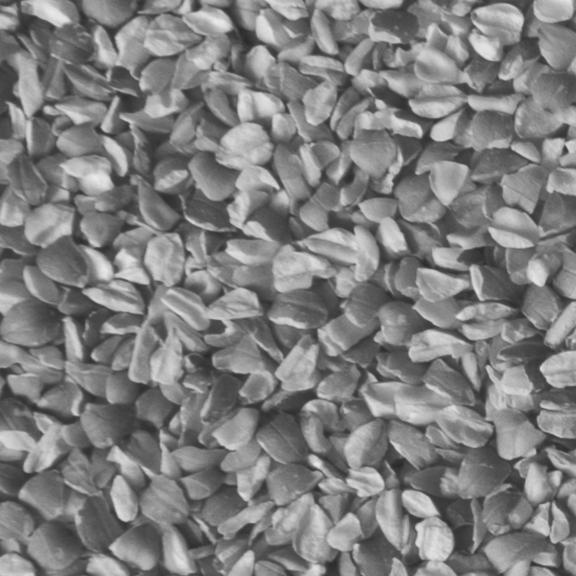

Supplement: Supplemental Information 1 [file peerj-cs-08-869-s001.zip › 0_part1/496_linseeds1-a-p010.jpg]

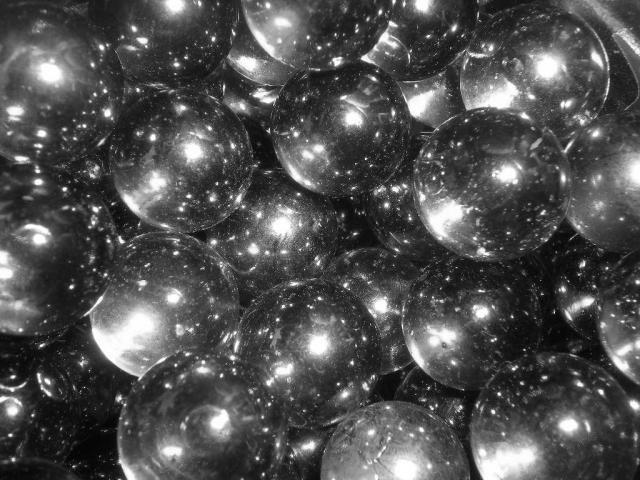

Supplement: Supplemental Information 1 [file peerj-cs-08-869-s001.zip › 0_part1/497_bubbly_0126.jpg]

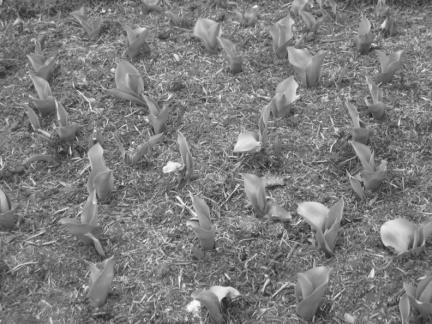

Supplement: Supplemental Information 1 [file peerj-cs-08-869-s001.zip › 0_part1/498_Flora31_6.jpg]

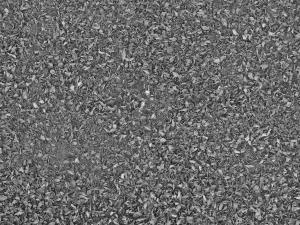

Supplement: Supplemental Information 1 [file peerj-cs-08-869-s001.zip › 0_part1/499_ground_ground_leaves_0003_01_thumb.jpg]

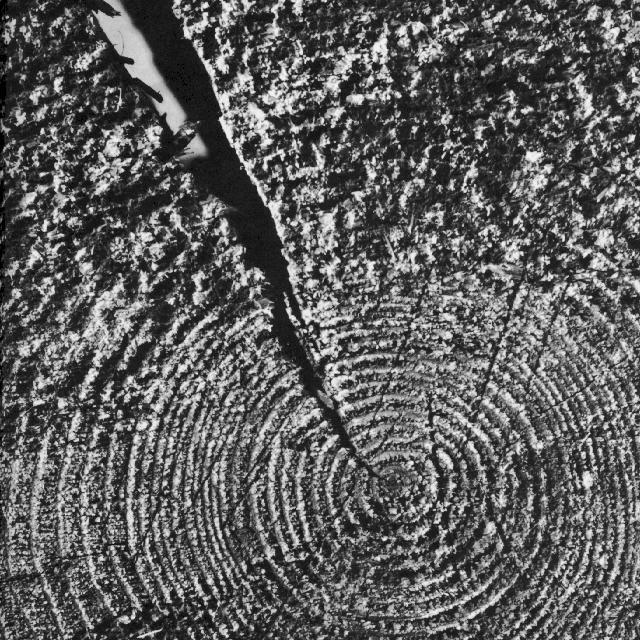

Supplement: Supplemental Information 1 [file peerj-cs-08-869-s001.zip › 0_part1/500_D97.jpg]

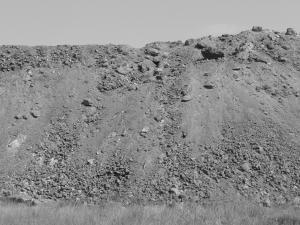

Supplement: Supplemental Information 1 [file peerj-cs-08-869-s001.zip › 0_part1/501_ground_slope_0049_01_thumb.jpg]

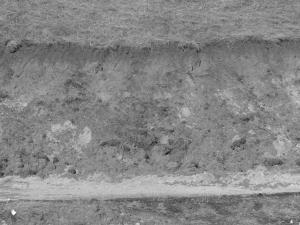

Supplement: Supplemental Information 1 [file peerj-cs-08-869-s001.zip › 0_part1/502_ground_slope_0041_01_thumb.jpg]

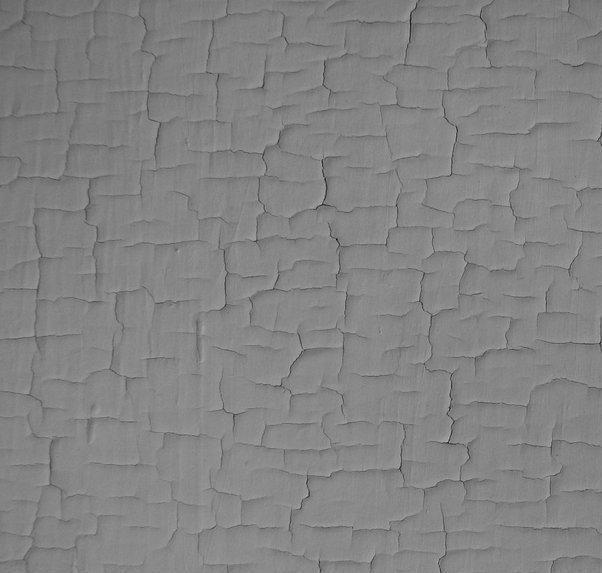

Supplement: Supplemental Information 1 [file peerj-cs-08-869-s001.zip › 0_part1/503_cracked_0111.jpg]

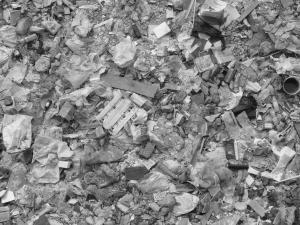

Supplement: Supplemental Information 1 [file peerj-cs-08-869-s001.zip › 0_part1/504_debris_garbage_0020_01_thumb.jpg]

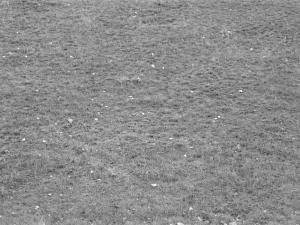

Supplement: Supplemental Information 1 [file peerj-cs-08-869-s001.zip › 0_part1/505_ground_slope_0010_01_thumb.jpg]

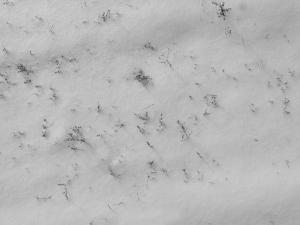

Supplement: Supplemental Information 1 [file peerj-cs-08-869-s001.zip › 0_part1/506_grass_other_grass_0031_01_thumb.jpg]

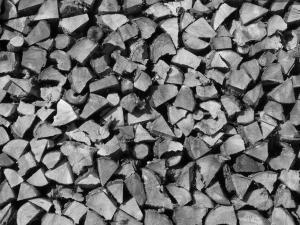

Supplement: Supplemental Information 1 [file peerj-cs-08-869-s001.zip › 0_part1/507_wood_constructions_0035_01_thumb.jpg]

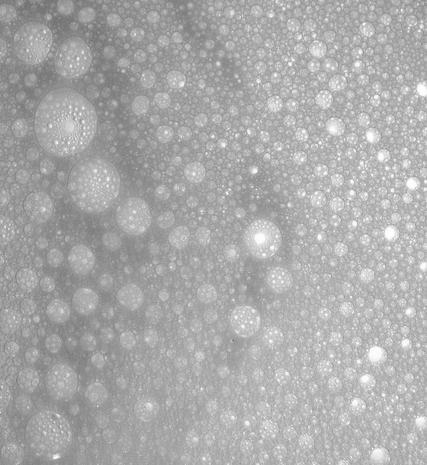

Supplement: Supplemental Information 1 [file peerj-cs-08-869-s001.zip › 0_part1/508_bubbly_0079.jpg]

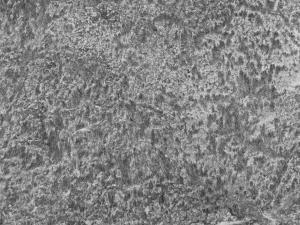

Supplement: Supplemental Information 1 [file peerj-cs-08-869-s001.zip › 0_part1/509_nature_moss_0052_01_thumb.jpg]

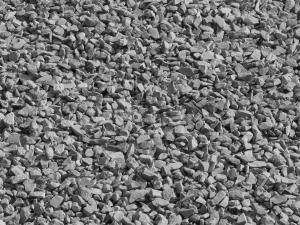

Supplement: Supplemental Information 1 [file peerj-cs-08-869-s001.zip › 0_part1/510_ground_pebble_0044_01_thumb.jpg]

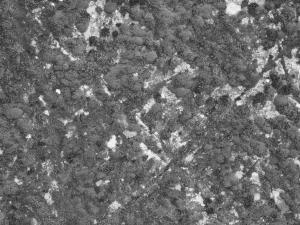

Supplement: Supplemental Information 1 [file peerj-cs-08-869-s001.zip › 0_part1/511_nature_moss_0040_01_thumb.jpg]

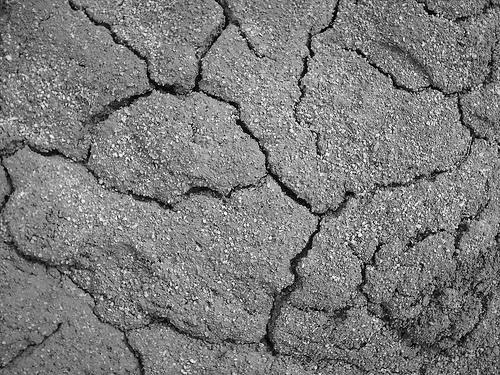

Supplement: Supplemental Information 1 [file peerj-cs-08-869-s001.zip › 0_part1/512_cracked_0048.jpg]

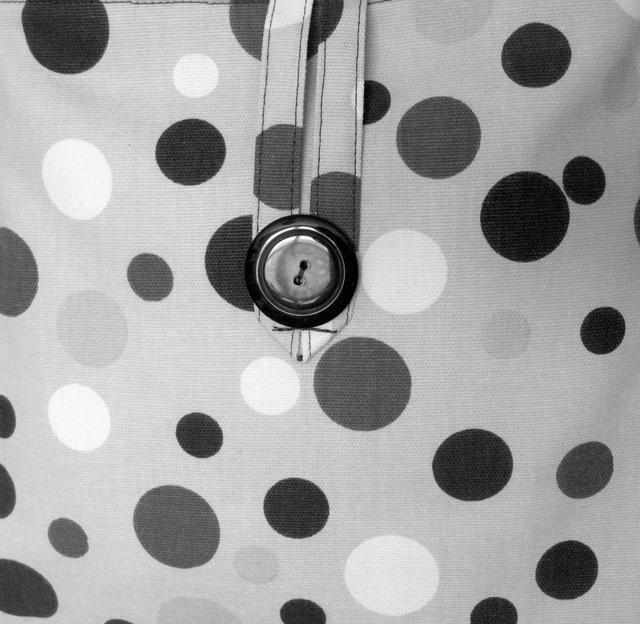

Supplement: Supplemental Information 1 [file peerj-cs-08-869-s001.zip › 0_part1/513_dotted_0117.jpg]

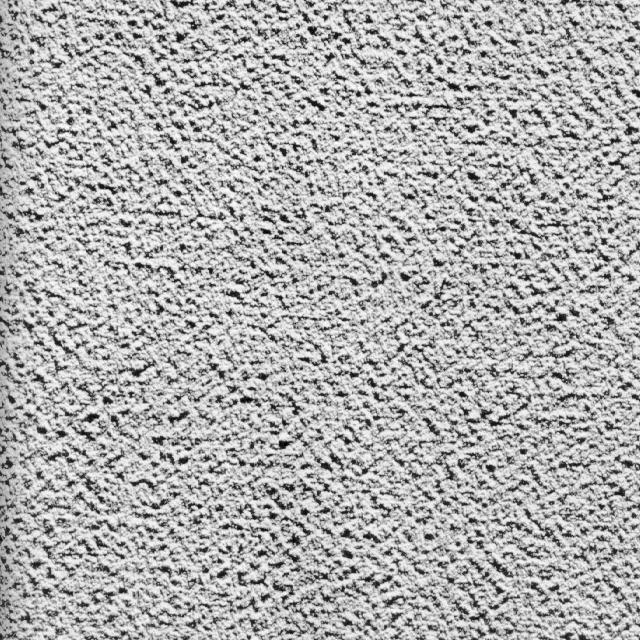

Supplement: Supplemental Information 1 [file peerj-cs-08-869-s001.zip › 0_part1/514_D57.jpg]

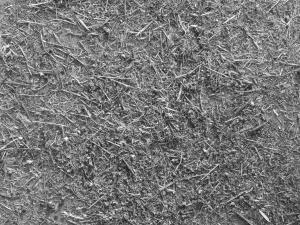

Supplement: Supplemental Information 1 [file peerj-cs-08-869-s001.zip › 0_part1/515_debris_wood_chips_0020_01_thumb.jpg]

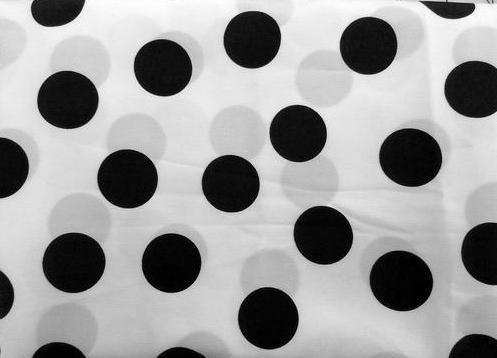

Supplement: Supplemental Information 1 [file peerj-cs-08-869-s001.zip › 0_part1/516_dotted_0103.jpg]

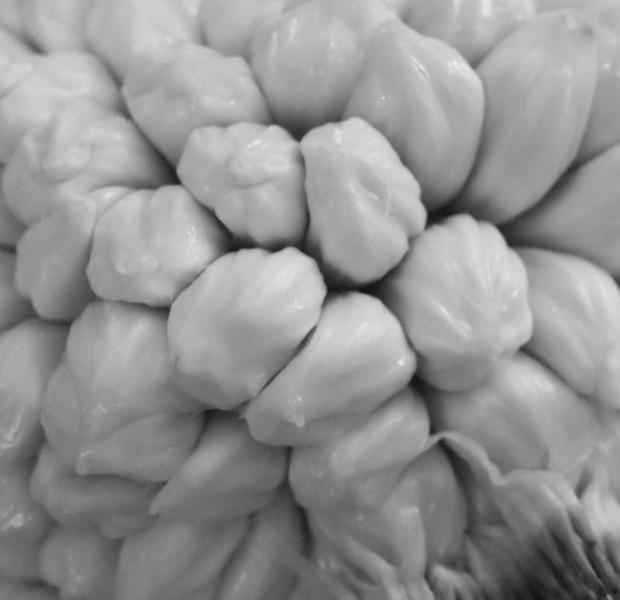

Supplement: Supplemental Information 1 [file peerj-cs-08-869-s001.zip › 0_part1/517_bumpy_0104.jpg]

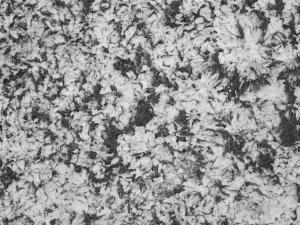

Supplement: Supplemental Information 1 [file peerj-cs-08-869-s001.zip › 0_part1/518_ground_frozen_ground_0031_01_thumb.jpg]

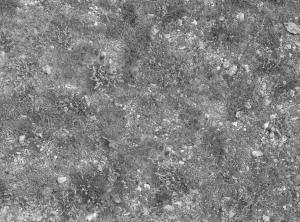

Supplement: Supplemental Information 1 [file peerj-cs-08-869-s001.zip › 0_part1/519_grass_on_stones_0002_01_thumb.jpg]

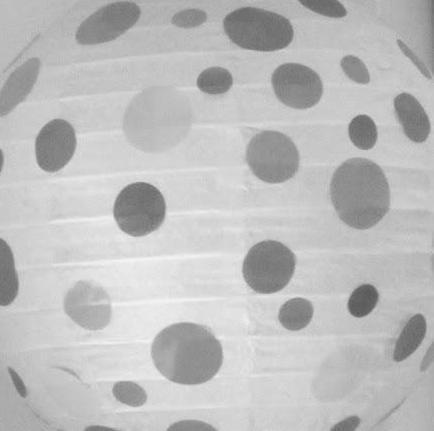

Supplement: Supplemental Information 1 [file peerj-cs-08-869-s001.zip › 0_part1/520_dotted_0119.jpg]

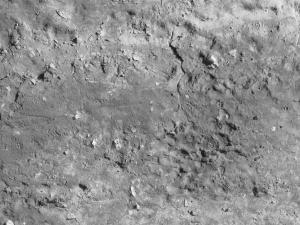

Supplement: Supplemental Information 1 [file peerj-cs-08-869-s001.zip › 0_part1/521_ground_ground_garbage_0026_01_thumb.jpg]

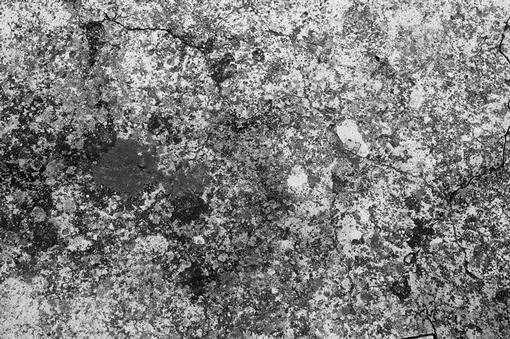

Supplement: Supplemental Information 1 [file peerj-cs-08-869-s001.zip › 0_part1/522_porous_0060.jpg]

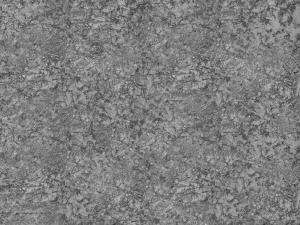

Supplement: Supplemental Information 1 [file peerj-cs-08-869-s001.zip › 0_part1/523_grass_grass_0060_01_thumb.jpg]

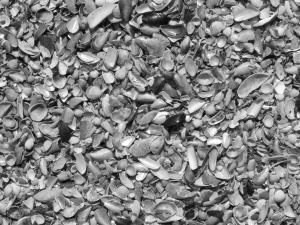

Supplement: Supplemental Information 1 [file peerj-cs-08-869-s001.zip › 0_part1/524_ground_pebble_0015_01_thumb.jpg]

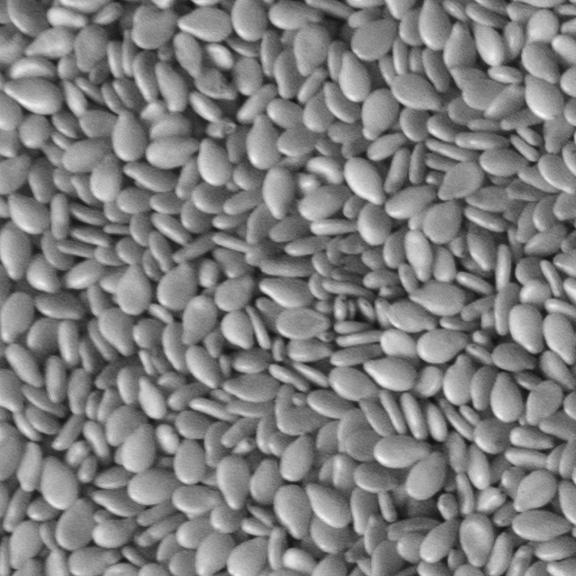

Supplement: Supplemental Information 1 [file peerj-cs-08-869-s001.zip › 0_part1/525_sesameseeds1-a-p010.jpg]

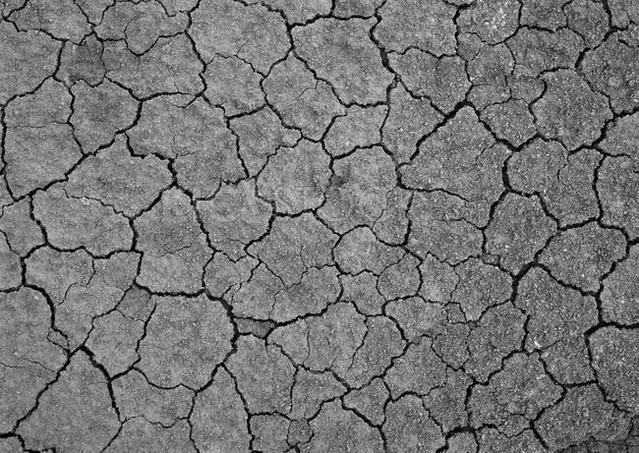

Supplement: Supplemental Information 1 [file peerj-cs-08-869-s001.zip › 0_part1/526_cracked_0052.jpg]

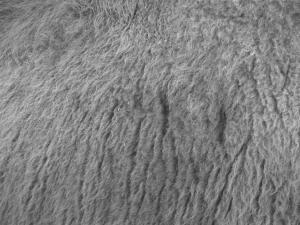

Supplement: Supplemental Information 1 [file peerj-cs-08-869-s001.zip › 0_part1/527_animals_fur_0023_01_thumb.jpg]

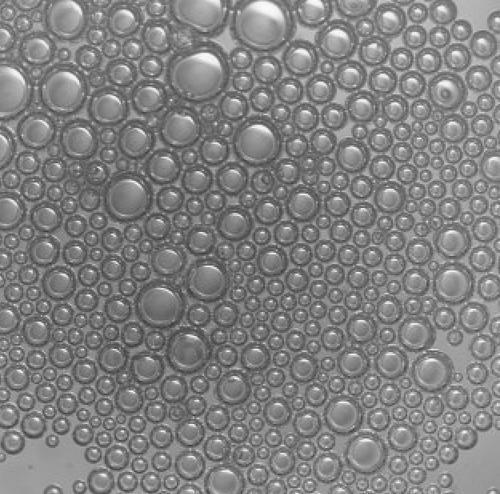

Supplement: Supplemental Information 1 [file peerj-cs-08-869-s001.zip › 0_part1/528_bubbly_0056.jpg]

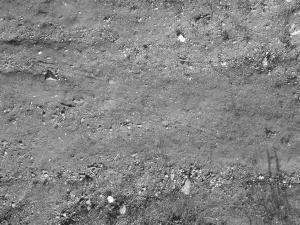

Supplement: Supplemental Information 1 [file peerj-cs-08-869-s001.zip › 0_part1/529_ground_ground_garbage_0032_01_thumb.jpg]

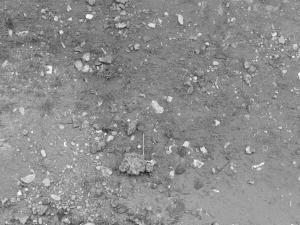

Supplement: Supplemental Information 1 [file peerj-cs-08-869-s001.zip › 0_part1/530_ground_ground_garbage_0029_01_thumb.jpg]

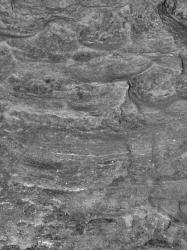

Supplement: Supplemental Information 1 [file peerj-cs-08-869-s001.zip › 0_part1/531_rock_cave_0020_01_thumb.jpg]

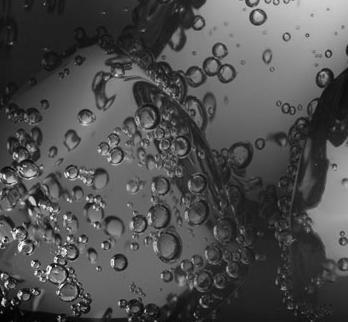

Supplement: Supplemental Information 1 [file peerj-cs-08-869-s001.zip › 0_part1/532_bubbly_0082.jpg]

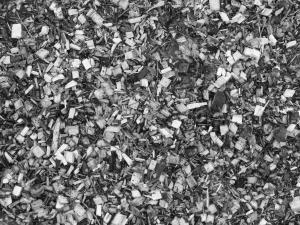

Supplement: Supplemental Information 1 [file peerj-cs-08-869-s001.zip › 0_part1/533_debris_wood_chips_0001_01_thumb.jpg]

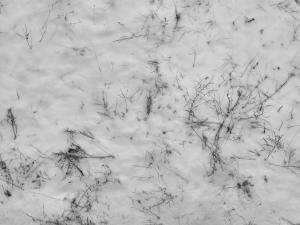

Supplement: Supplemental Information 1 [file peerj-cs-08-869-s001.zip › 0_part1/534_ground_frozen_ground_0005_01_thumb.jpg]

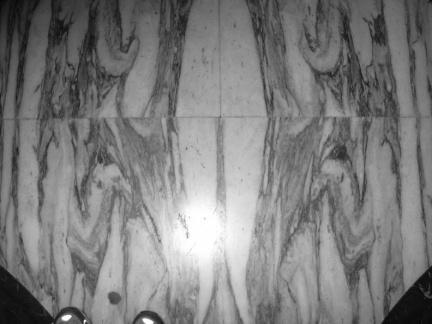

Supplement: Supplemental Information 1 [file peerj-cs-08-869-s001.zip › 0_part1/535_texture_8.jpg]

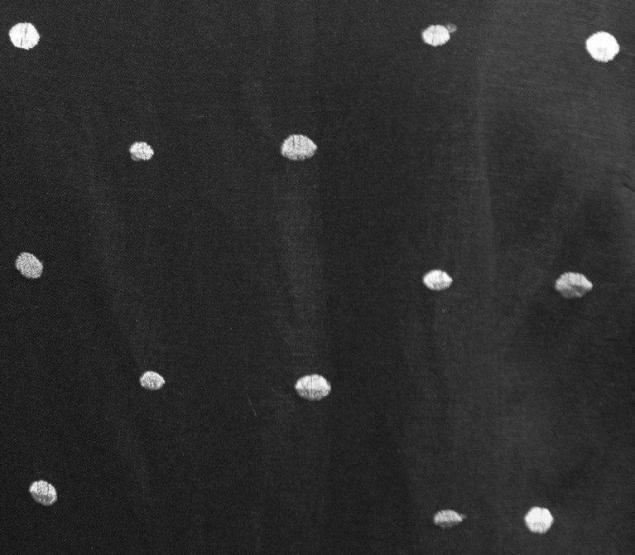

Supplement: Supplemental Information 1 [file peerj-cs-08-869-s001.zip › 0_part1/536_dotted_0148.jpg]

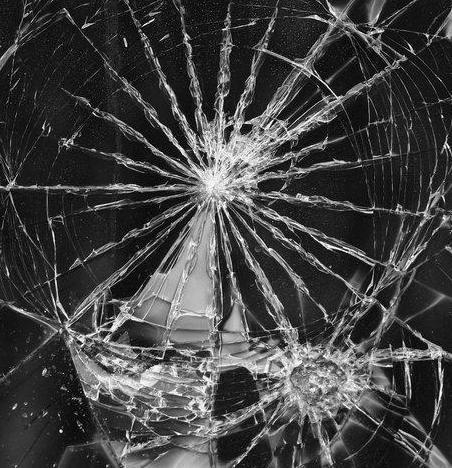

Supplement: Supplemental Information 1 [file peerj-cs-08-869-s001.zip › 0_part1/537_cracked_0145.jpg]

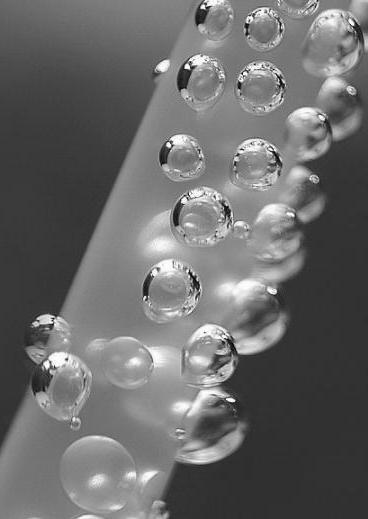

Supplement: Supplemental Information 1 [file peerj-cs-08-869-s001.zip › 0_part1/538_bubbly_0154.jpg]

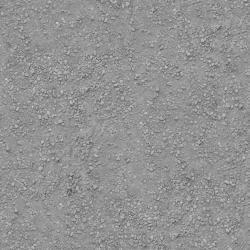

Supplement: Supplemental Information 1 [file peerj-cs-08-869-s001.zip › 0_part1/539_ground_stone_ground_0028_02_thumb.jpg]

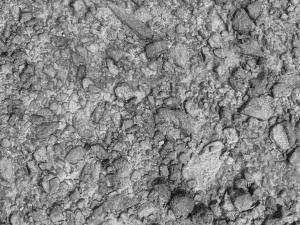

Supplement: Supplemental Information 1 [file peerj-cs-08-869-s001.zip › 0_part1/540_ground_stone_ground_0046_01_thumb.jpg]

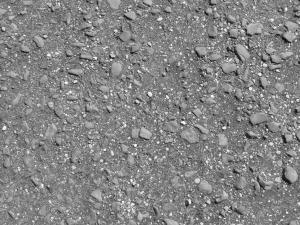

Supplement: Supplemental Information 1 [file peerj-cs-08-869-s001.zip › 0_part1/541_ground_stone_ground_0068_01_thumb.jpg]

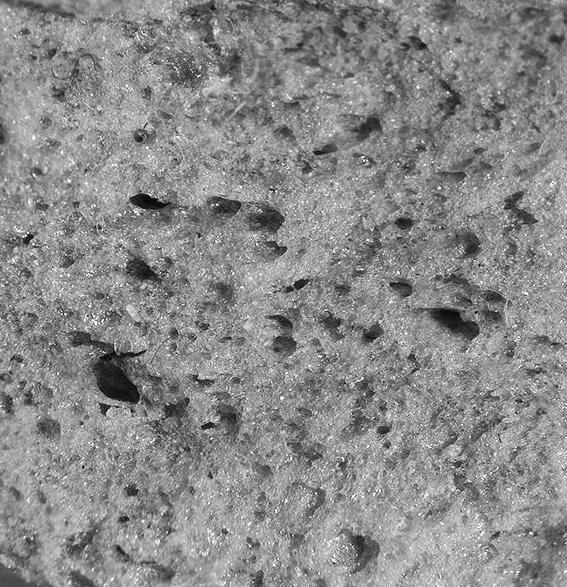

Supplement: Supplemental Information 1 [file peerj-cs-08-869-s001.zip › 0_part1/542_porous_0104.jpg]

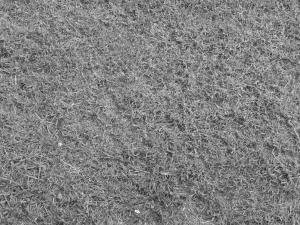

Supplement: Supplemental Information 1 [file peerj-cs-08-869-s001.zip › 0_part1/543_grass_grass_0093_04_thumb.jpg]

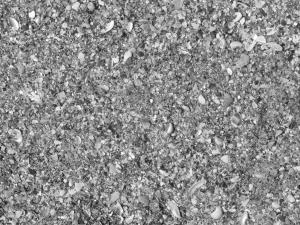

Supplement: Supplemental Information 1 [file peerj-cs-08-869-s001.zip › 0_part1/544_ground_pebble_0017_01_thumb.jpg]

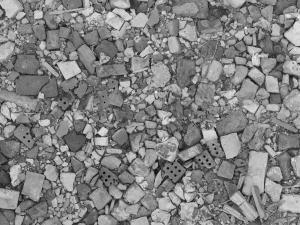

Supplement: Supplemental Information 1 [file peerj-cs-08-869-s001.zip › 0_part1/545_debris_stone_debris_0027_01_thumb.jpg]

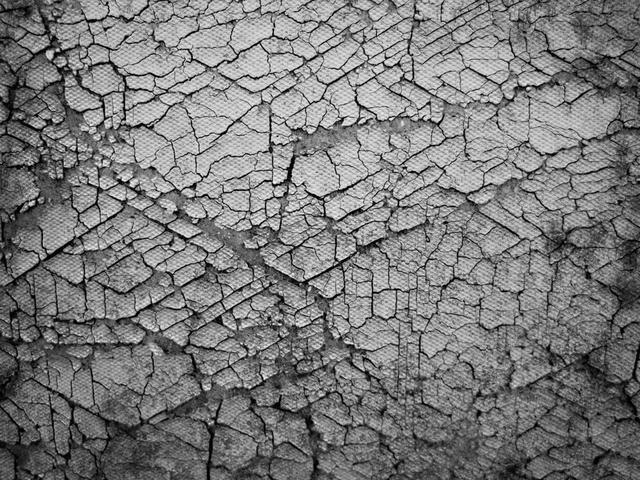

Supplement: Supplemental Information 1 [file peerj-cs-08-869-s001.zip › 0_part1/546_cracked_0054.jpg]

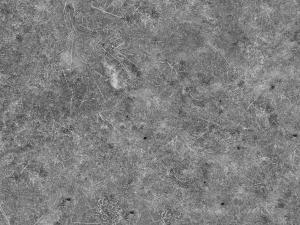

Supplement: Supplemental Information 1 [file peerj-cs-08-869-s001.zip › 0_part1/547_grass_grass_0034_01_thumb.jpg]

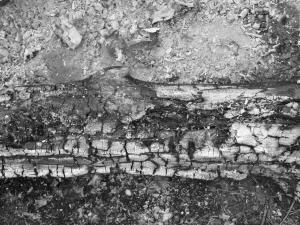

Supplement: Supplemental Information 1 [file peerj-cs-08-869-s001.zip › 0_part1/548_wood_burnt_0007_01_thumb.jpg]

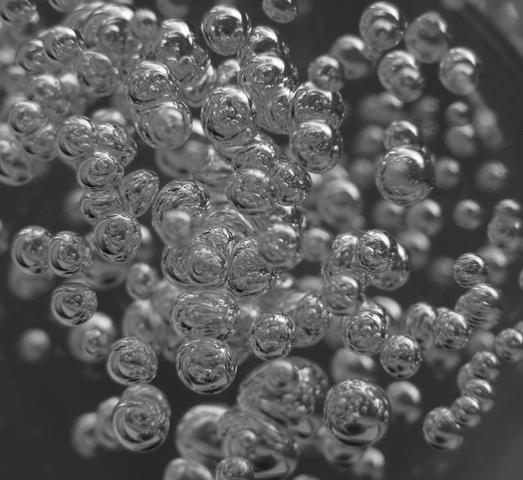

Supplement: Supplemental Information 1 [file peerj-cs-08-869-s001.zip › 0_part1/549_bubbly_0066.jpg]

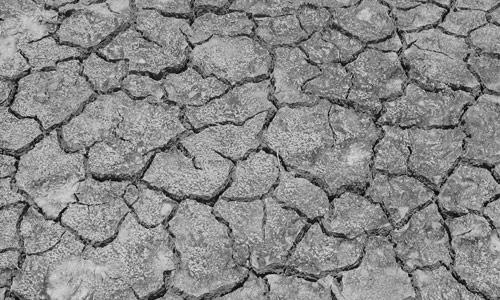

Supplement: Supplemental Information 1 [file peerj-cs-08-869-s001.zip › 0_part1/550_cracked_0076.jpg]

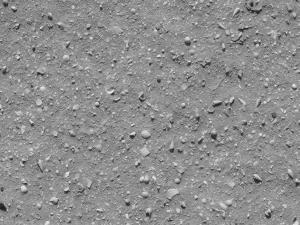

Supplement: Supplemental Information 1 [file peerj-cs-08-869-s001.zip › 0_part1/551_ground_pebble_0043_01_thumb.jpg]

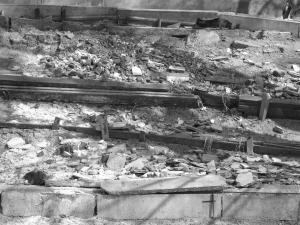

Supplement: Supplemental Information 1 [file peerj-cs-08-869-s001.zip › 0_part1/552_ground_slope_0013_01_thumb.jpg]

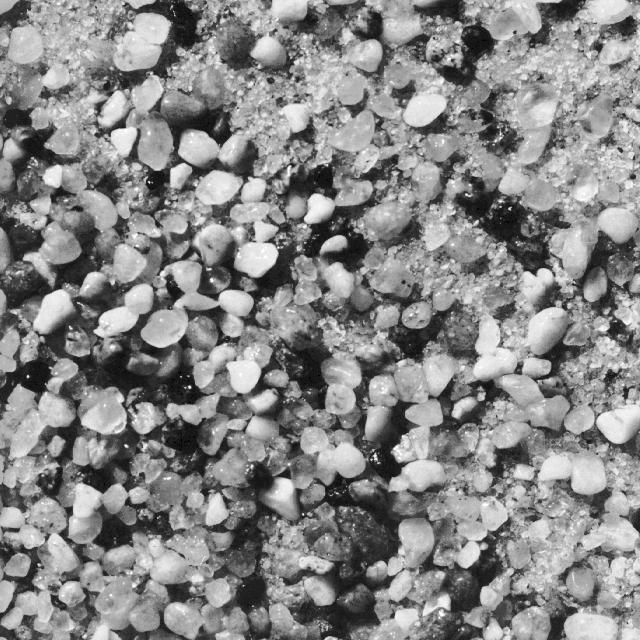

Supplement: Supplemental Information 1 [file peerj-cs-08-869-s001.zip › 0_part1/553_D27.jpg]

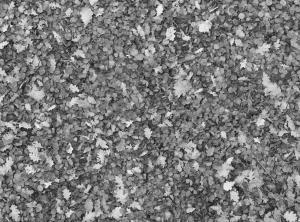

Supplement: Supplemental Information 1 [file peerj-cs-08-869-s001.zip › 0_part1/554_ground_ground_leaves_0020_01_thumb.jpg]

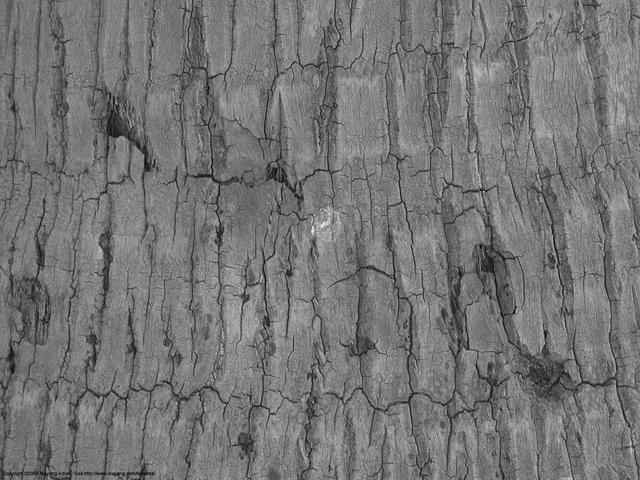

Supplement: Supplemental Information 1 [file peerj-cs-08-869-s001.zip › 0_part1/555_cracked_0073.jpg]

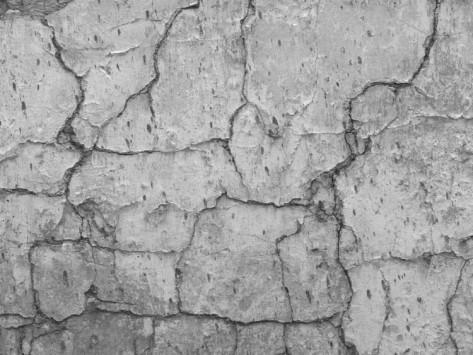

Supplement: Supplemental Information 1 [file peerj-cs-08-869-s001.zip › 0_part1/556_cracked_0133.jpg]

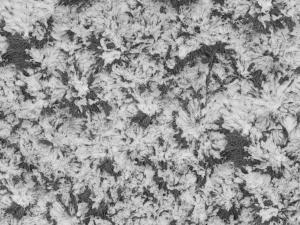

Supplement: Supplemental Information 1 [file peerj-cs-08-869-s001.zip › 0_part1/557_ground_frozen_ground_0048_01_thumb.jpg]

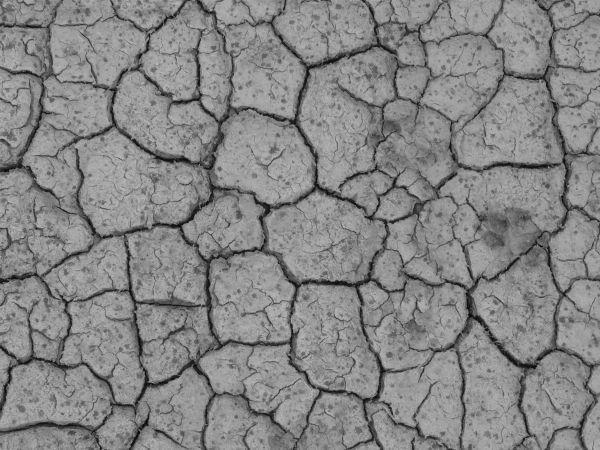

Supplement: Supplemental Information 1 [file peerj-cs-08-869-s001.zip › 0_part1/558_cracked_0155.jpg]

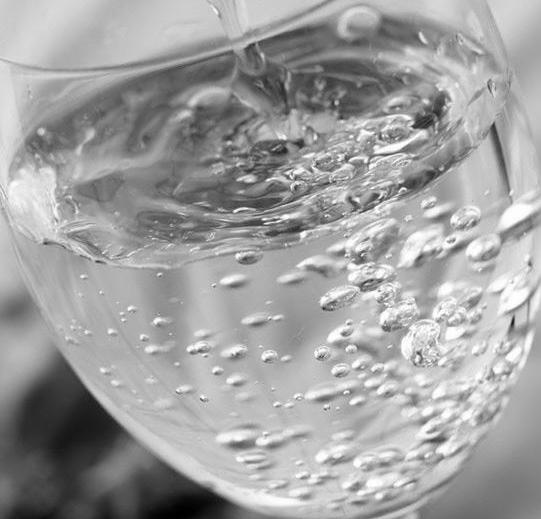

Supplement: Supplemental Information 1 [file peerj-cs-08-869-s001.zip › 0_part1/559_bubbly_0163.jpg]

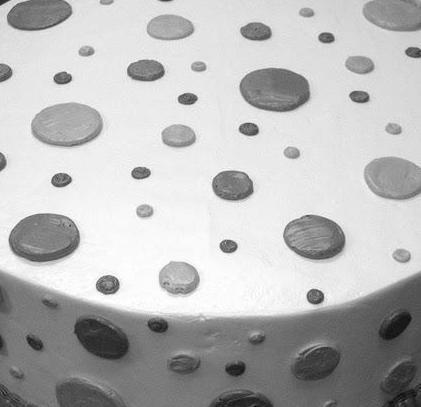

Supplement: Supplemental Information 1 [file peerj-cs-08-869-s001.zip › 0_part1/560_dotted_0110.jpg]

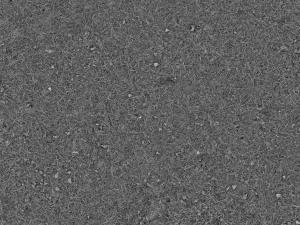

Supplement: Supplemental Information 1 [file peerj-cs-08-869-s001.zip › 0_part1/561_ground_ground_leaves_0026_01_thumb.jpg]

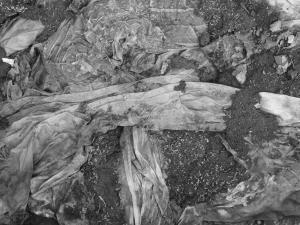

Supplement: Supplemental Information 1 [file peerj-cs-08-869-s001.zip › 0_part1/562_debris_garbage_0024_01_thumb.jpg]

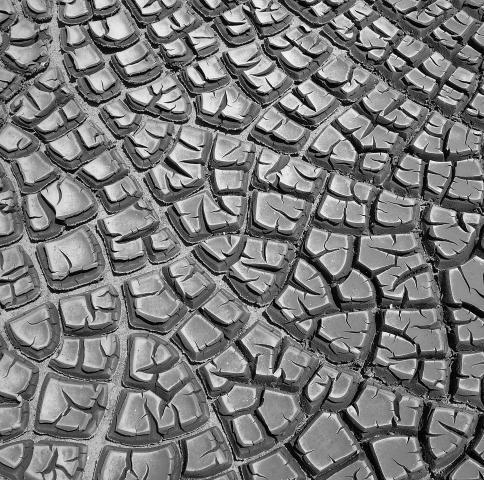

Supplement: Supplemental Information 1 [file peerj-cs-08-869-s001.zip › 0_part1/563_cracked_0129.jpg]

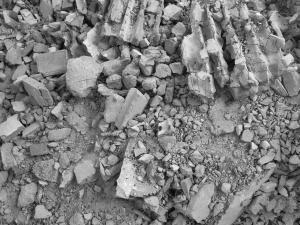

Supplement: Supplemental Information 1 [file peerj-cs-08-869-s001.zip › 0_part1/564_debris_stone_debris_0005_01_thumb.jpg]

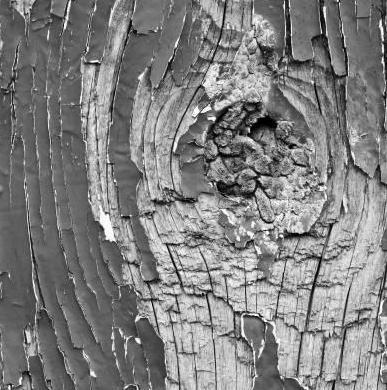

Supplement: Supplemental Information 1 [file peerj-cs-08-869-s001.zip › 0_part1/565_cracked_0105.jpg]

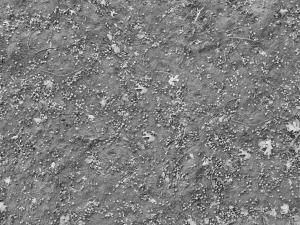

Supplement: Supplemental Information 1 [file peerj-cs-08-869-s001.zip › 0_part1/566_ground_ground_leaves_0027_01_thumb.jpg]

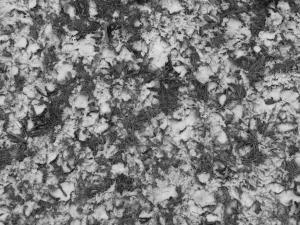

Supplement: Supplemental Information 1 [file peerj-cs-08-869-s001.zip › 0_part1/567_ground_frozen_ground_0035_01_thumb.jpg]

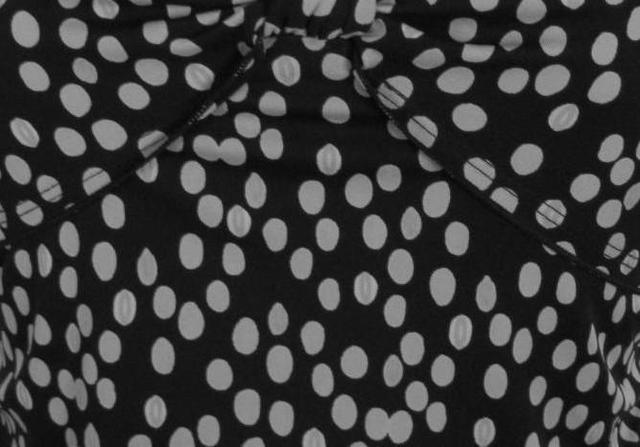

Supplement: Supplemental Information 1 [file peerj-cs-08-869-s001.zip › 0_part1/568_dotted_0115.jpg]

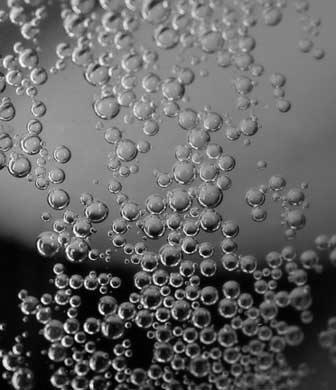

Supplement: Supplemental Information 1 [file peerj-cs-08-869-s001.zip › 0_part1/569_bubbly_0147.jpg]

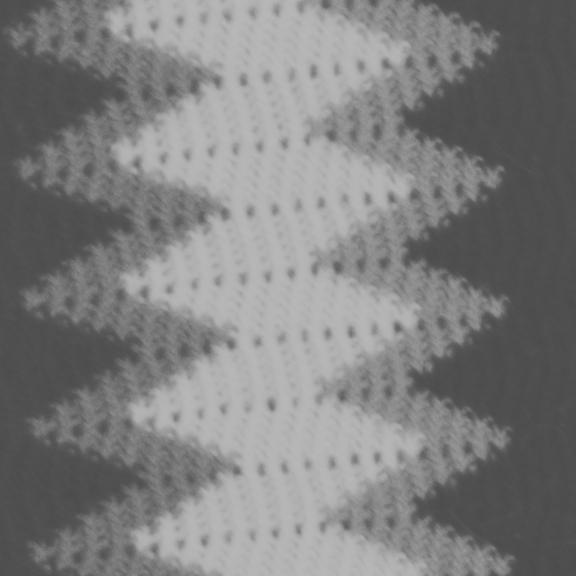

Supplement: Supplemental Information 1 [file peerj-cs-08-869-s001.zip › 0_part1/570_blanket2-a-p008.jpg]

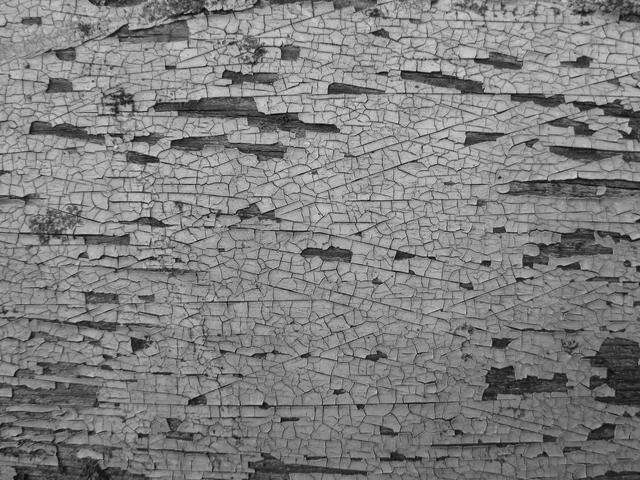

Supplement: Supplemental Information 1 [file peerj-cs-08-869-s001.zip › 0_part1/571_cracked_0059.jpg]

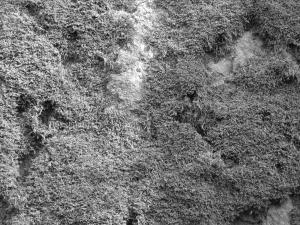

Supplement: Supplemental Information 1 [file peerj-cs-08-869-s001.zip › 0_part1/572_nature_moss_0043_01_thumb.jpg]

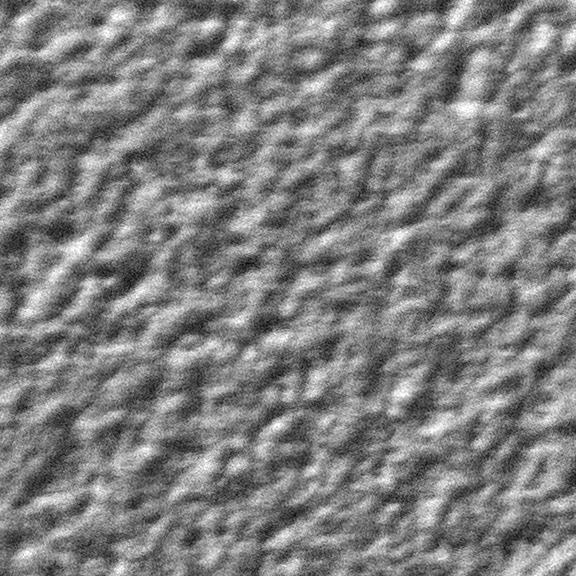

Supplement: Supplemental Information 1 [file peerj-cs-08-869-s001.zip › 0_part1/573_ceiling1-a-p001.jpg]

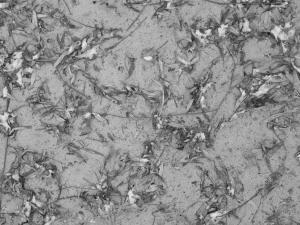

Supplement: Supplemental Information 1 [file peerj-cs-08-869-s001.zip › 0_part1/574_ground_frozen_ground_0016_01_thumb.jpg]

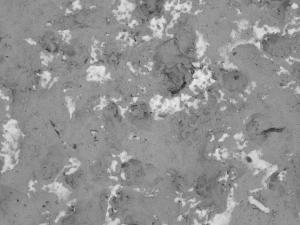

Supplement: Supplemental Information 1 [file peerj-cs-08-869-s001.zip › 0_part1/575_ground_frozen_ground_0019_01_thumb.jpg]

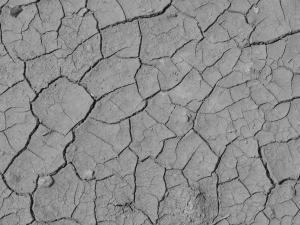

Supplement: Supplemental Information 1 [file peerj-cs-08-869-s001.zip › 0_part1/576_soil_cracked_0031_01_thumb.jpg]

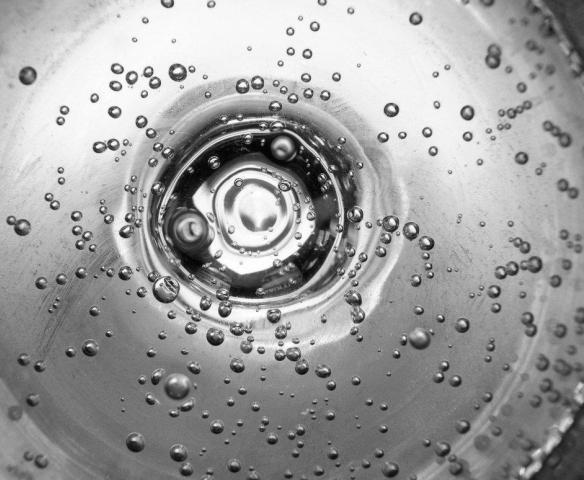

Supplement: Supplemental Information 1 [file peerj-cs-08-869-s001.zip › 0_part1/577_bubbly_0144.jpg]

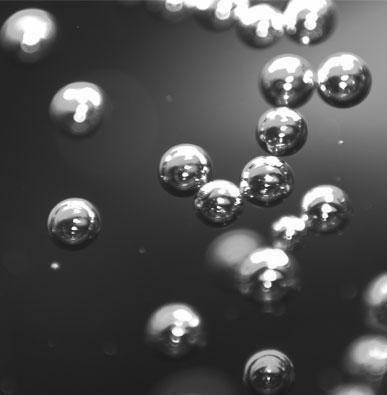

Supplement: Supplemental Information 1 [file peerj-cs-08-869-s001.zip › 0_part1/578_bubbly_0117.jpg]

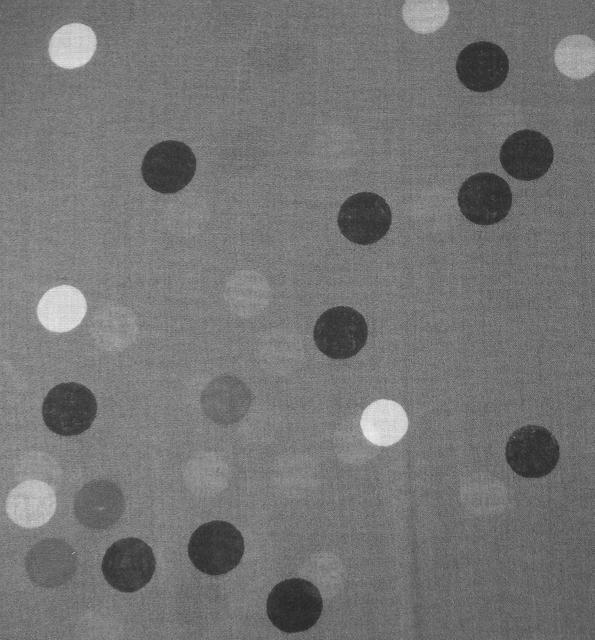

Supplement: Supplemental Information 1 [file peerj-cs-08-869-s001.zip › 0_part1/579_dotted_0130.jpg]

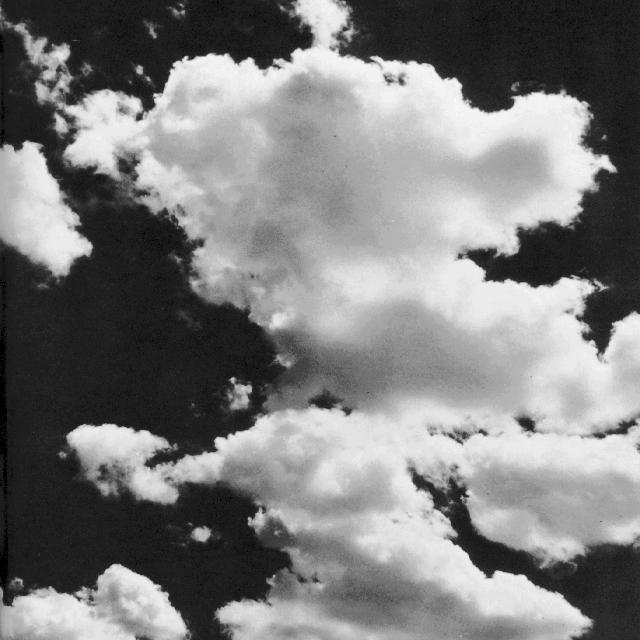

Supplement: Supplemental Information 1 [file peerj-cs-08-869-s001.zip › 0_part1/580_D91.jpg]

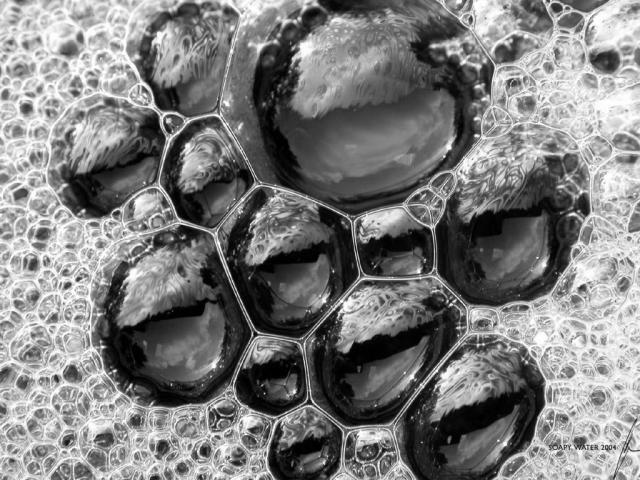

Supplement: Supplemental Information 1 [file peerj-cs-08-869-s001.zip › 0_part1/581_bubbly_0151.jpg]

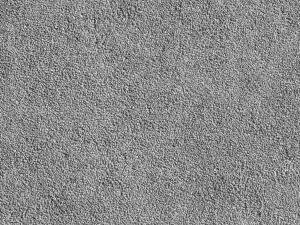

Supplement: Supplemental Information 1 [file peerj-cs-08-869-s001.zip › 0_part1/582_ground_pebble_0032_01_thumb.jpg]

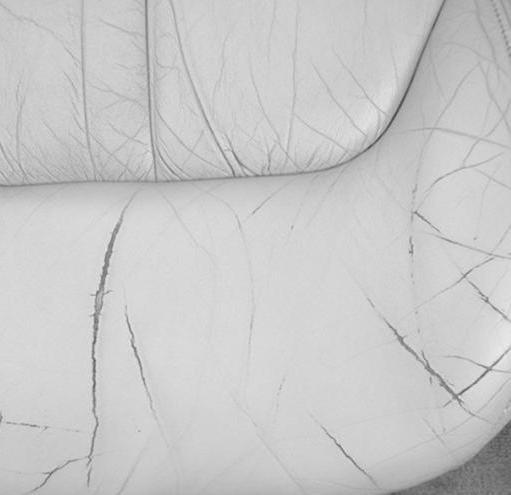

Supplement: Supplemental Information 1 [file peerj-cs-08-869-s001.zip › 0_part1/583_cracked_0160.jpg]

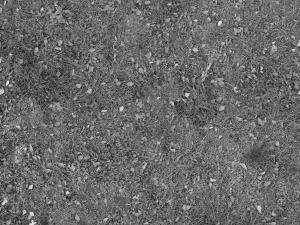

Supplement: Supplemental Information 1 [file peerj-cs-08-869-s001.zip › 0_part1/584_grass_leaves_0001_01_thumb.jpg]

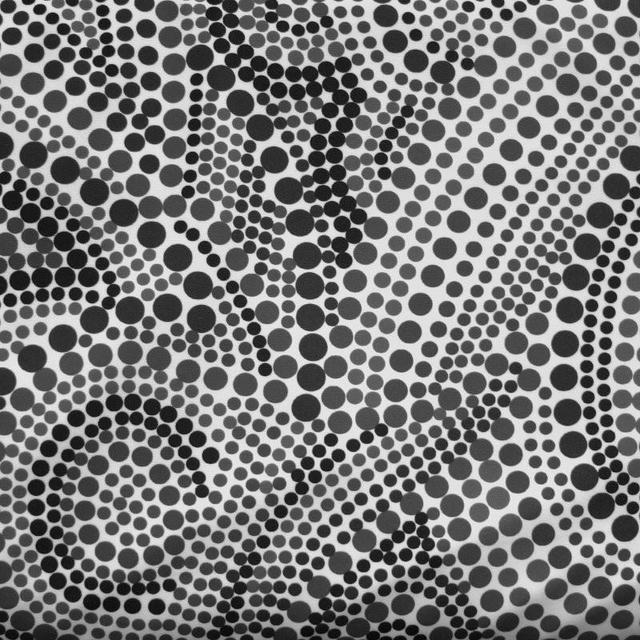

Supplement: Supplemental Information 1 [file peerj-cs-08-869-s001.zip › 0_part1/585_dotted_0106.jpg]

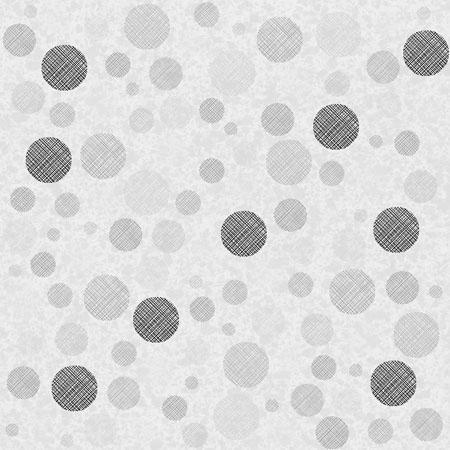

Supplement: Supplemental Information 1 [file peerj-cs-08-869-s001.zip › 0_part1/586_dotted_0097.jpg]

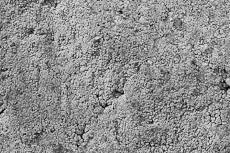

Supplement: Supplemental Information 1 [file peerj-cs-08-869-s001.zip › 0_part1/587_S_S_IMG_0225.jpg]

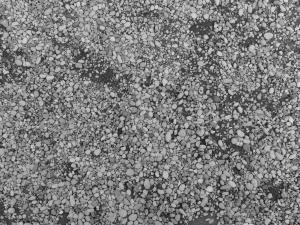

Supplement: Supplemental Information 1 [file peerj-cs-08-869-s001.zip › 0_part1/588_ground_pebble_0054_01_thumb.jpg]

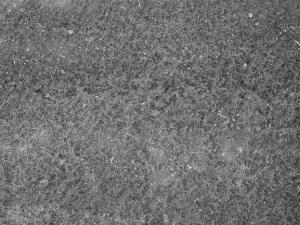

Supplement: Supplemental Information 1 [file peerj-cs-08-869-s001.zip › 0_part1/589_grass_grass_0022_01_thumb.jpg]
