# Supplementary figures and images for: Deep convolutional neural networks for regular texture recognition (part 4 of 8)
Source: PeerJ Comput Sci. 2022 Feb 9;8:e869. doi: 10.7717/peerj-cs.869 (PMC9044313; doi:10.7717/peerj-cs.869)

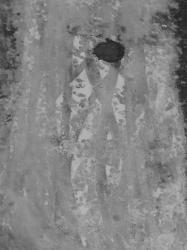

Supplement: Supplemental Information 2 [file peerj-cs-08-869-s002.zip › 0_part2/192_ground_frozen_ground_0062_01_thumb.jpg]

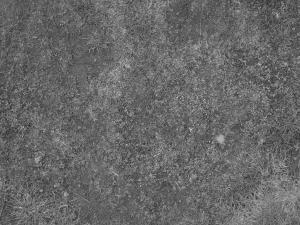

Supplement: Supplemental Information 2 [file peerj-cs-08-869-s002.zip › 0_part2/193_grass_grass_0039_01_thumb.jpg]

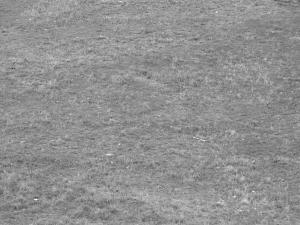

Supplement: Supplemental Information 2 [file peerj-cs-08-869-s002.zip › 0_part2/194_ground_slope_0011_01_thumb.jpg]

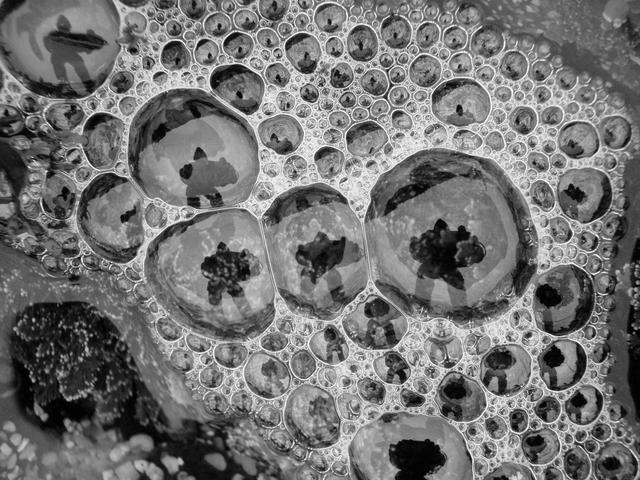

Supplement: Supplemental Information 2 [file peerj-cs-08-869-s002.zip › 0_part2/195_bubbly_0050.jpg]

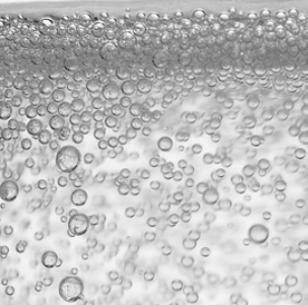

Supplement: Supplemental Information 2 [file peerj-cs-08-869-s002.zip › 0_part2/196_bubbly_0044.jpg]

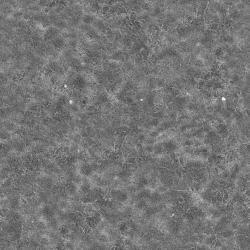

Supplement: Supplemental Information 2 [file peerj-cs-08-869-s002.zip › 0_part2/197_grass_grass_0012_02_thumb.jpg]

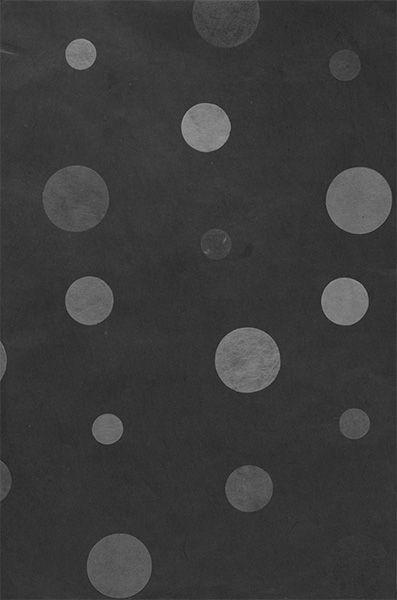

Supplement: Supplemental Information 2 [file peerj-cs-08-869-s002.zip › 0_part2/198_dotted_0166.jpg]

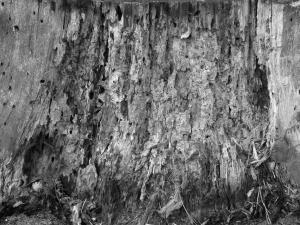

Supplement: Supplemental Information 2 [file peerj-cs-08-869-s002.zip › 0_part2/199_wood_rotten_0011_01_thumb.jpg]

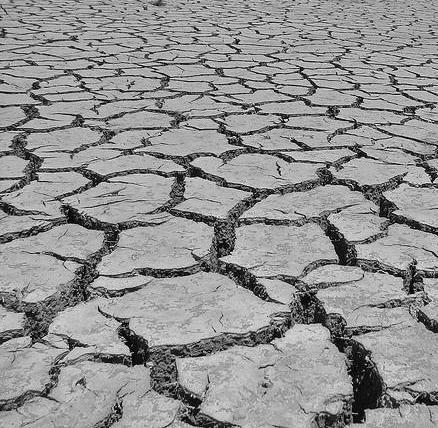

Supplement: Supplemental Information 2 [file peerj-cs-08-869-s002.zip › 0_part2/19_cracked_0128.jpg]

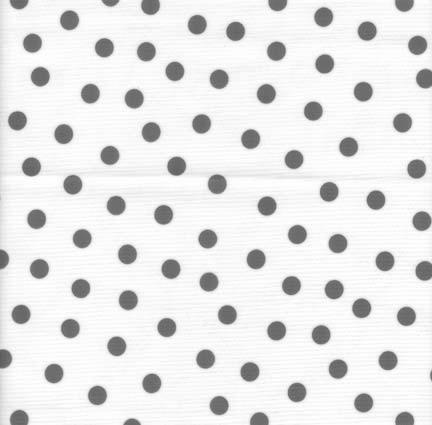

Supplement: Supplemental Information 2 [file peerj-cs-08-869-s002.zip › 0_part2/1_dotted_0181.jpg]

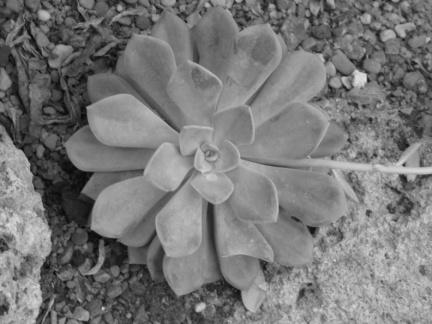

Supplement: Supplemental Information 2 [file peerj-cs-08-869-s002.zip › 0_part2/200_Flora31_37.jpg]

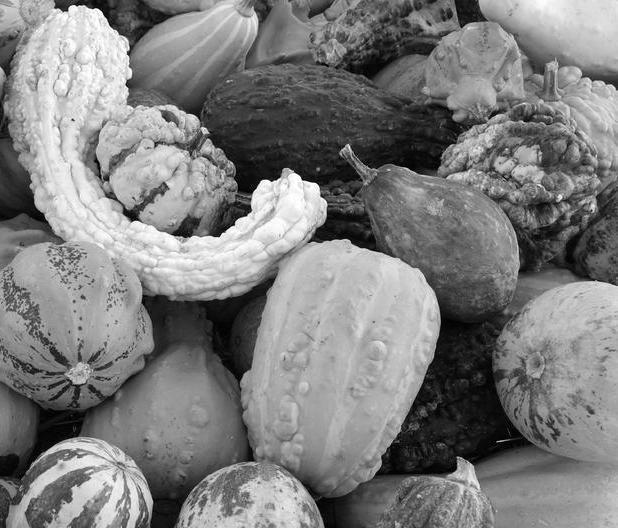

Supplement: Supplemental Information 2 [file peerj-cs-08-869-s002.zip › 0_part2/201_bumpy_0195.jpg]

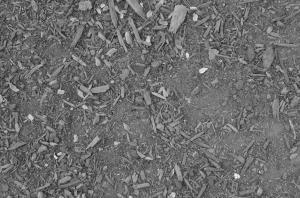

Supplement: Supplemental Information 2 [file peerj-cs-08-869-s002.zip › 0_part2/202_debris_wood_chips_0026_01_thumb.jpg]

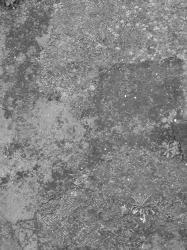

Supplement: Supplemental Information 2 [file peerj-cs-08-869-s002.zip › 0_part2/203_grass_other_grass_0036_01_thumb.jpg]

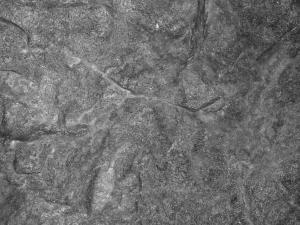

Supplement: Supplemental Information 2 [file peerj-cs-08-869-s002.zip › 0_part2/204_rock_cave_0027_01_thumb.jpg]

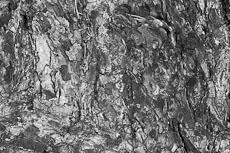

Supplement: Supplemental Information 2 [file peerj-cs-08-869-s002.zip › 0_part2/205_S_S_IMG_0070.jpg]

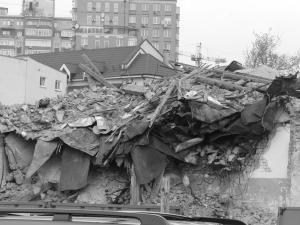

Supplement: Supplemental Information 2 [file peerj-cs-08-869-s002.zip › 0_part2/206_debris_other_0003_01_thumb.jpg]

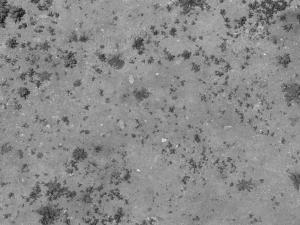

Supplement: Supplemental Information 2 [file peerj-cs-08-869-s002.zip › 0_part2/207_ground_other_ground_0013_01_thumb.jpg]

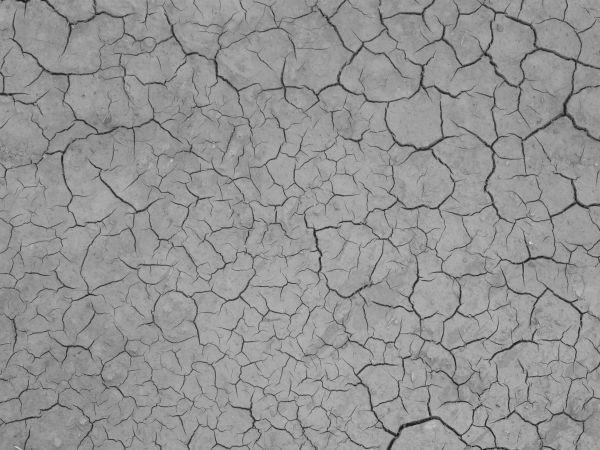

Supplement: Supplemental Information 2 [file peerj-cs-08-869-s002.zip › 0_part2/208_cracked_0158.jpg]

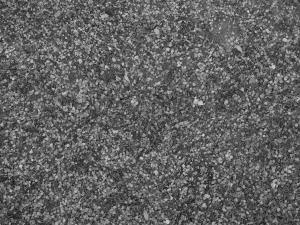

Supplement: Supplemental Information 2 [file peerj-cs-08-869-s002.zip › 0_part2/209_ground_ground_leaves_0002_01_thumb.jpg]

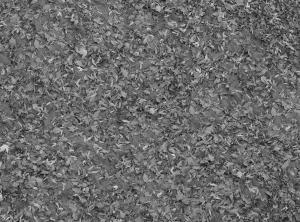

Supplement: Supplemental Information 2 [file peerj-cs-08-869-s002.zip › 0_part2/20_ground_ground_leaves_0030_01_thumb.jpg]

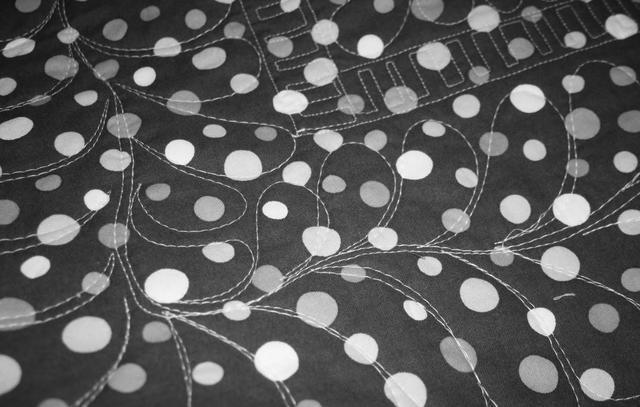

Supplement: Supplemental Information 2 [file peerj-cs-08-869-s002.zip › 0_part2/210_dotted_0143.jpg]

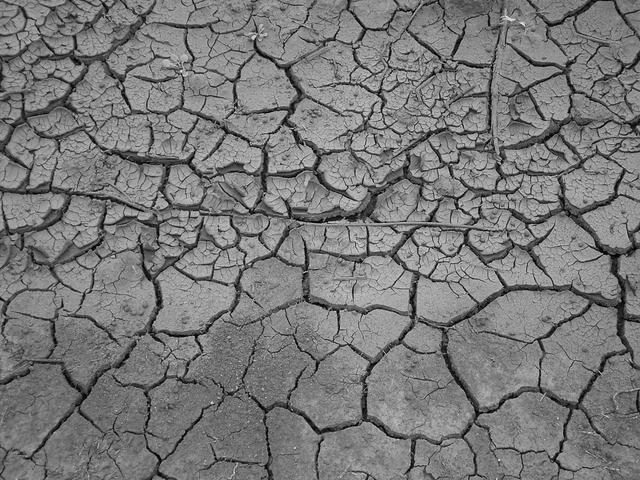

Supplement: Supplemental Information 2 [file peerj-cs-08-869-s002.zip › 0_part2/211_cracked_0100.jpg]

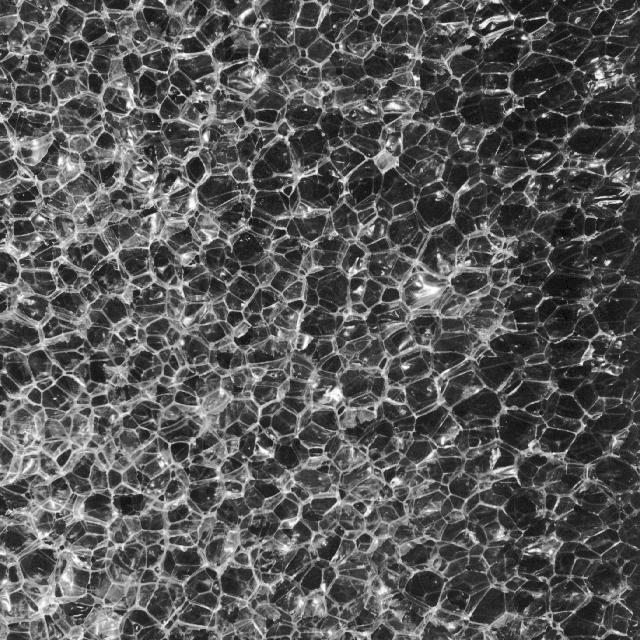

Supplement: Supplemental Information 2 [file peerj-cs-08-869-s002.zip › 0_part2/212_D112.jpg]

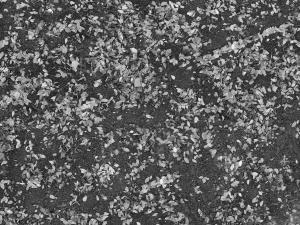

Supplement: Supplemental Information 2 [file peerj-cs-08-869-s002.zip › 0_part2/213_ground_ground_leaves_0004_01_thumb.jpg]

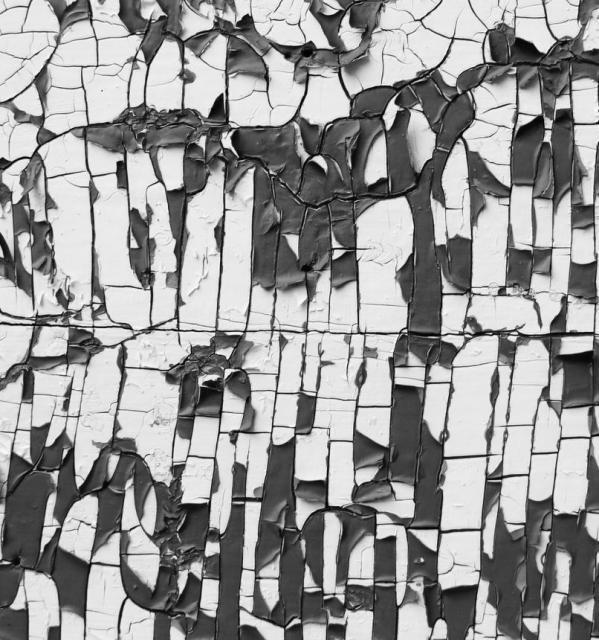

Supplement: Supplemental Information 2 [file peerj-cs-08-869-s002.zip › 0_part2/214_cracked_0131.jpg]

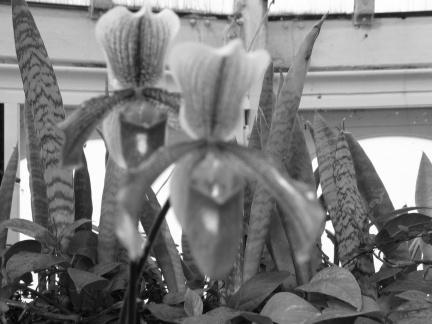

Supplement: Supplemental Information 2 [file peerj-cs-08-869-s002.zip › 0_part2/215_Flora31_70.jpg]

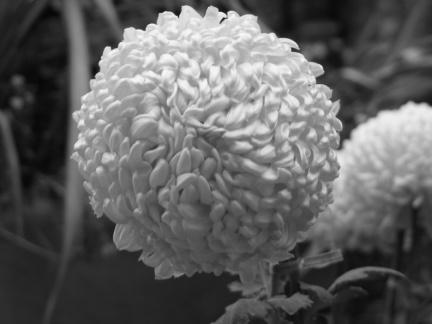

Supplement: Supplemental Information 2 [file peerj-cs-08-869-s002.zip › 0_part2/216_Flora31_22.jpg]

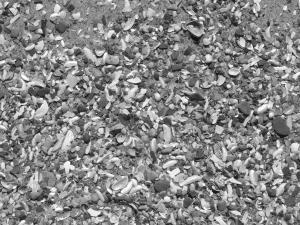

Supplement: Supplemental Information 2 [file peerj-cs-08-869-s002.zip › 0_part2/217_ground_pebble_0024_01_thumb.jpg]

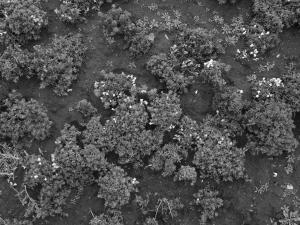

Supplement: Supplemental Information 2 [file peerj-cs-08-869-s002.zip › 0_part2/218_ground_other_ground_0007_01_thumb.jpg]

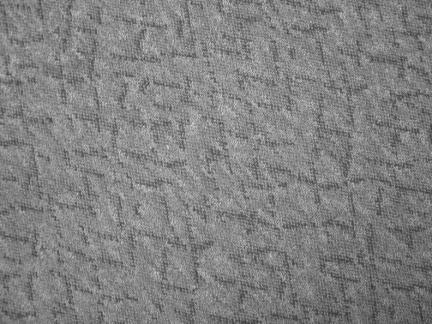

Supplement: Supplemental Information 2 [file peerj-cs-08-869-s002.zip › 0_part2/219_Pure Texture 171_113.jpg]

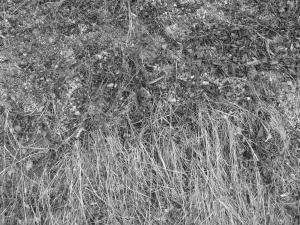

Supplement: Supplemental Information 2 [file peerj-cs-08-869-s002.zip › 0_part2/21_ground_slope_0012_01_thumb.jpg]

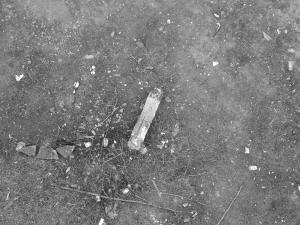

Supplement: Supplemental Information 2 [file peerj-cs-08-869-s002.zip › 0_part2/220_ground_ground_garbage_0020_01_thumb.jpg]

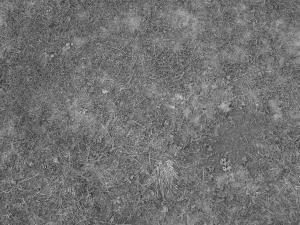

Supplement: Supplemental Information 2 [file peerj-cs-08-869-s002.zip › 0_part2/221_grass_grass_0040_01_thumb.jpg]

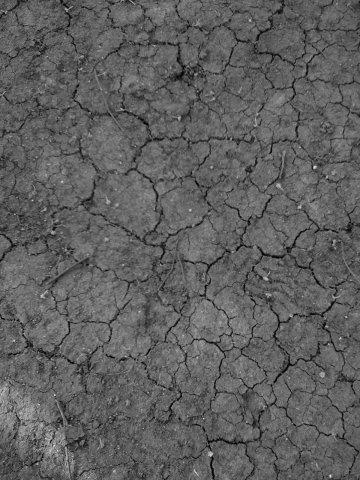

Supplement: Supplemental Information 2 [file peerj-cs-08-869-s002.zip › 0_part2/222_cracked_0062.jpg]

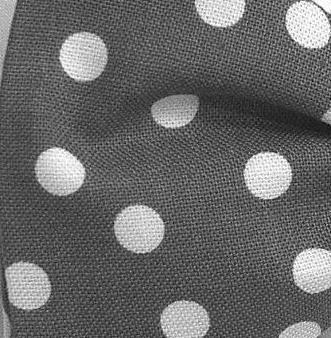

Supplement: Supplemental Information 2 [file peerj-cs-08-869-s002.zip › 0_part2/223_dotted_0134.jpg]

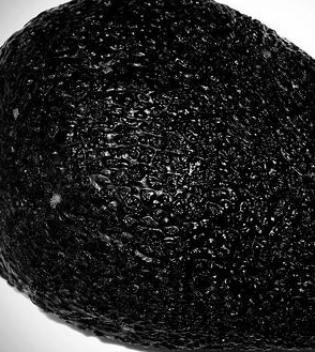

Supplement: Supplemental Information 2 [file peerj-cs-08-869-s002.zip › 0_part2/224_bumpy_0174.jpg]

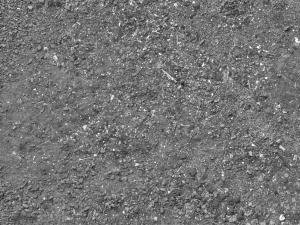

Supplement: Supplemental Information 2 [file peerj-cs-08-869-s002.zip › 0_part2/225_ground_ground_garbage_0001_01_thumb.jpg]

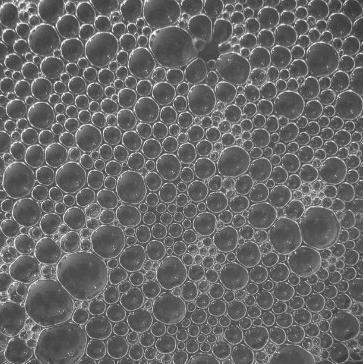

Supplement: Supplemental Information 2 [file peerj-cs-08-869-s002.zip › 0_part2/226_bubbly_0097.jpg]

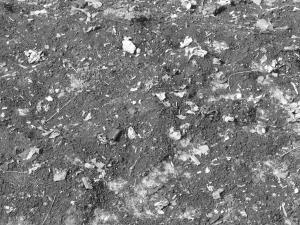

Supplement: Supplemental Information 2 [file peerj-cs-08-869-s002.zip › 0_part2/227_ground_ground_garbage_0023_01_thumb.jpg]

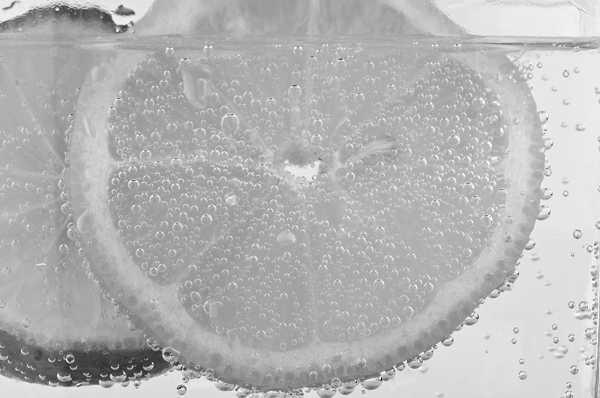

Supplement: Supplemental Information 2 [file peerj-cs-08-869-s002.zip › 0_part2/228_bubbly_0142.jpg]

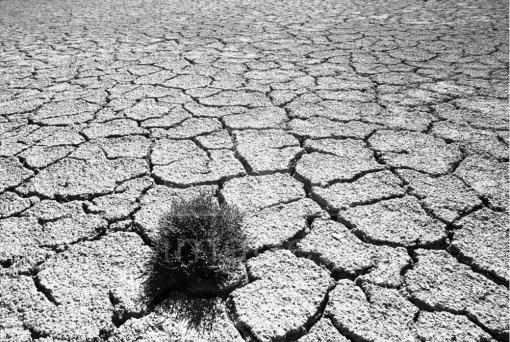

Supplement: Supplemental Information 2 [file peerj-cs-08-869-s002.zip › 0_part2/229_cracked_0090.jpg]

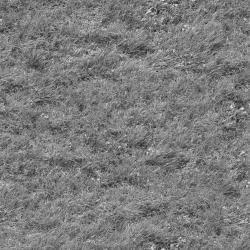

Supplement: Supplemental Information 2 [file peerj-cs-08-869-s002.zip › 0_part2/22_grass_grass_0071_02_thumb.jpg]

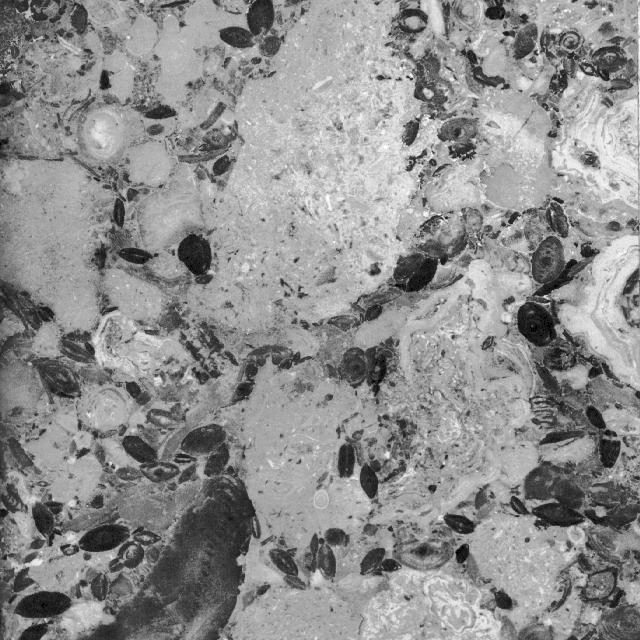

Supplement: Supplemental Information 2 [file peerj-cs-08-869-s002.zip › 0_part2/230_D61.jpg]

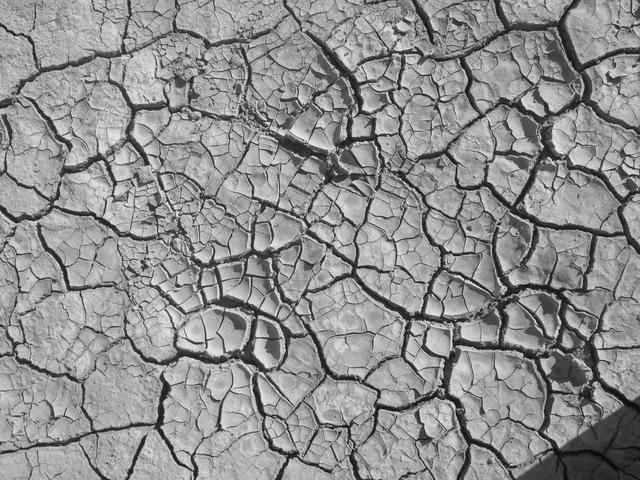

Supplement: Supplemental Information 2 [file peerj-cs-08-869-s002.zip › 0_part2/231_cracked_0047.jpg]

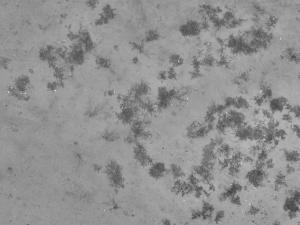

Supplement: Supplemental Information 2 [file peerj-cs-08-869-s002.zip › 0_part2/232_ground_other_ground_0006_01_thumb.jpg]

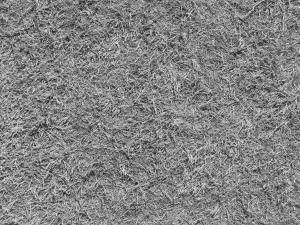

Supplement: Supplemental Information 2 [file peerj-cs-08-869-s002.zip › 0_part2/233_grass_grass_0106_01_thumb.jpg]

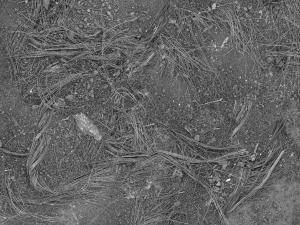

Supplement: Supplemental Information 2 [file peerj-cs-08-869-s002.zip › 0_part2/234_debris_metal_debris_0008_01_thumb.jpg]

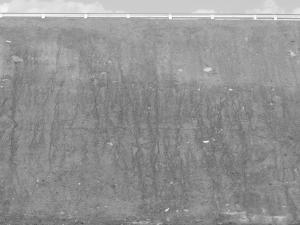

Supplement: Supplemental Information 2 [file peerj-cs-08-869-s002.zip › 0_part2/235_ground_slope_0024_01_thumb.jpg]

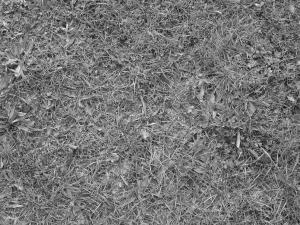

Supplement: Supplemental Information 2 [file peerj-cs-08-869-s002.zip › 0_part2/236_grass_grass_0079_01_thumb.jpg]

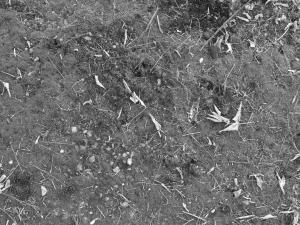

Supplement: Supplemental Information 2 [file peerj-cs-08-869-s002.zip › 0_part2/237_nature_moss_0012_01_thumb.jpg]

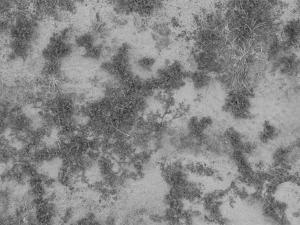

Supplement: Supplemental Information 2 [file peerj-cs-08-869-s002.zip › 0_part2/238_grass_grass_0059_01_thumb.jpg]

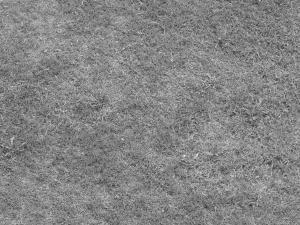

Supplement: Supplemental Information 2 [file peerj-cs-08-869-s002.zip › 0_part2/239_grass_grass_0098_01_thumb.jpg]

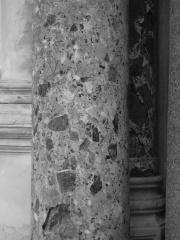

Supplement: Supplemental Information 2 [file peerj-cs-08-869-s002.zip › 0_part2/23_texture_13.jpg]

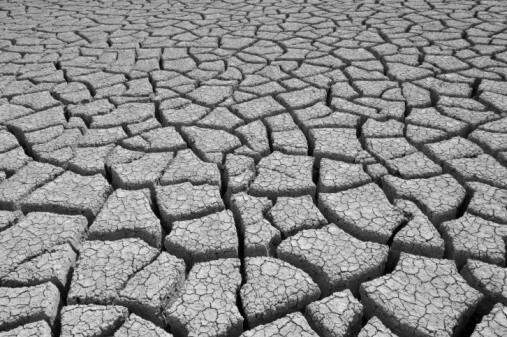

Supplement: Supplemental Information 2 [file peerj-cs-08-869-s002.zip › 0_part2/240_cracked_0084.jpg]

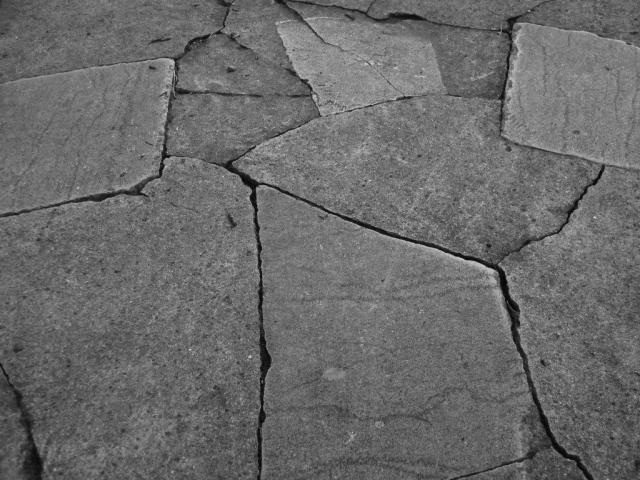

Supplement: Supplemental Information 2 [file peerj-cs-08-869-s002.zip › 0_part2/241_cracked_0132.jpg]

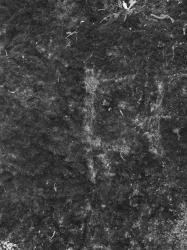

Supplement: Supplemental Information 2 [file peerj-cs-08-869-s002.zip › 0_part2/242_nature_moss_0031_01_thumb.jpg]

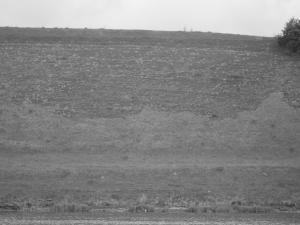

Supplement: Supplemental Information 2 [file peerj-cs-08-869-s002.zip › 0_part2/243_ground_slope_0034_01_thumb.jpg]

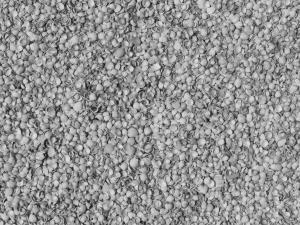

Supplement: Supplemental Information 2 [file peerj-cs-08-869-s002.zip › 0_part2/244_ground_pebble_0018_01_thumb.jpg]

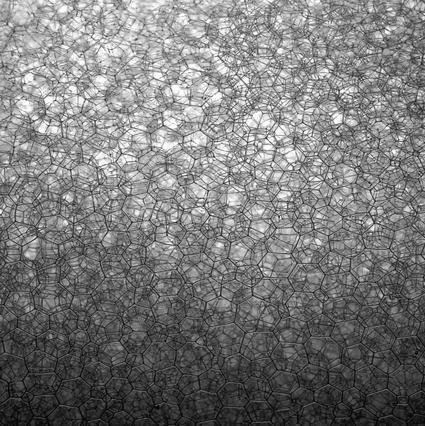

Supplement: Supplemental Information 2 [file peerj-cs-08-869-s002.zip › 0_part2/245_bubbly_0042.jpg]

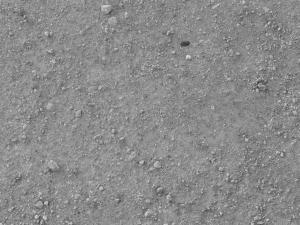

Supplement: Supplemental Information 2 [file peerj-cs-08-869-s002.zip › 0_part2/246_ground_stone_ground_0021_02_thumb.jpg]

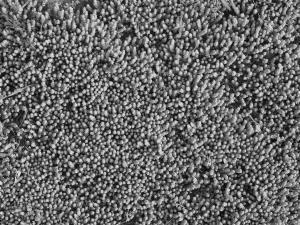

Supplement: Supplemental Information 2 [file peerj-cs-08-869-s002.zip › 0_part2/247_nature_moss_0018_01_thumb.jpg]

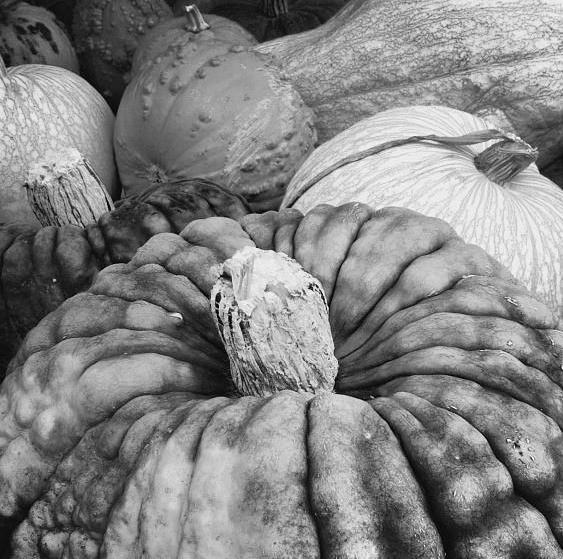

Supplement: Supplemental Information 2 [file peerj-cs-08-869-s002.zip › 0_part2/248_bumpy_0167.jpg]

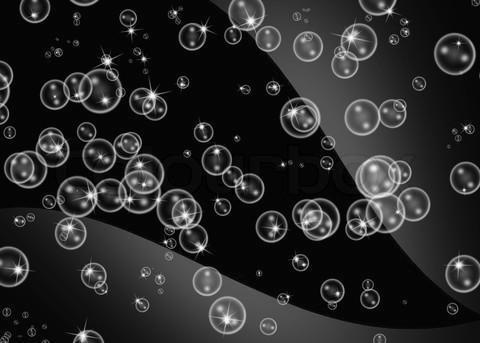

Supplement: Supplemental Information 2 [file peerj-cs-08-869-s002.zip › 0_part2/249_bubbly_0109.jpg]

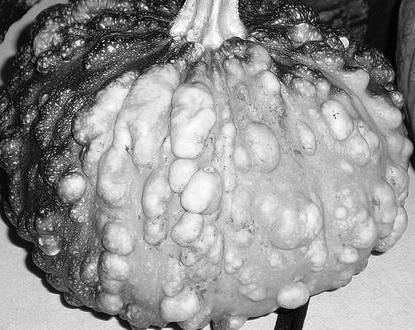

Supplement: Supplemental Information 2 [file peerj-cs-08-869-s002.zip › 0_part2/24_bumpy_0172.jpg]

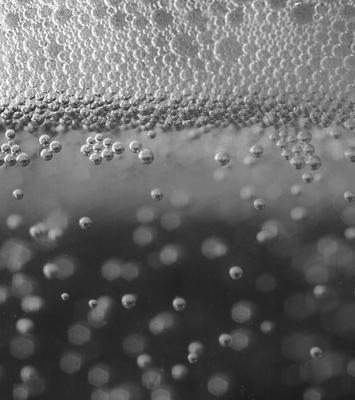

Supplement: Supplemental Information 2 [file peerj-cs-08-869-s002.zip › 0_part2/250_bubbly_0063.jpg]

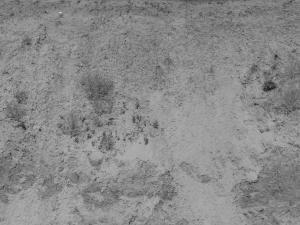

Supplement: Supplemental Information 2 [file peerj-cs-08-869-s002.zip › 0_part2/251_ground_slope_0015_01_thumb.jpg]

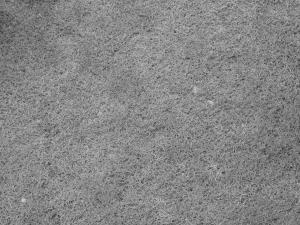

Supplement: Supplemental Information 2 [file peerj-cs-08-869-s002.zip › 0_part2/252_grass_grass_0119_01_thumb.jpg]

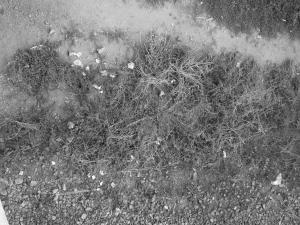

Supplement: Supplemental Information 2 [file peerj-cs-08-869-s002.zip › 0_part2/253_ground_ground_garbage_0012_01_thumb.jpg]

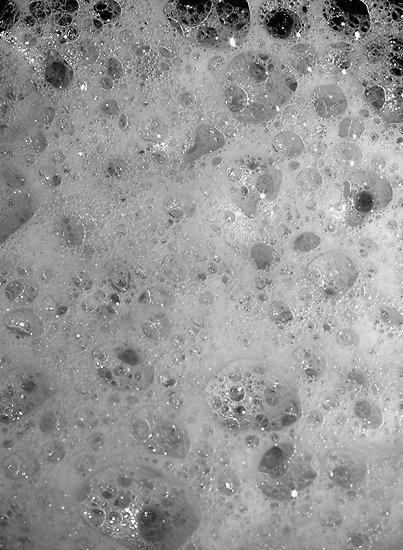

Supplement: Supplemental Information 2 [file peerj-cs-08-869-s002.zip › 0_part2/254_bubbly_0148.jpg]

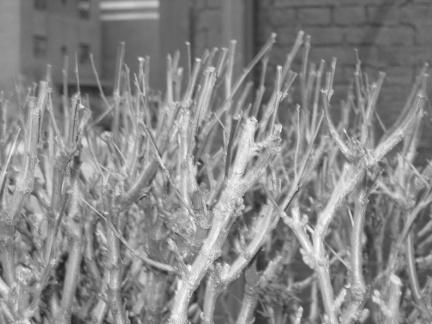

Supplement: Supplemental Information 2 [file peerj-cs-08-869-s002.zip › 0_part2/255_Flora31_0.jpg]

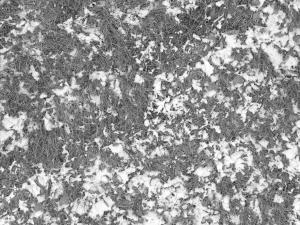

Supplement: Supplemental Information 2 [file peerj-cs-08-869-s002.zip › 0_part2/256_ground_frozen_ground_0024_01_thumb.jpg]

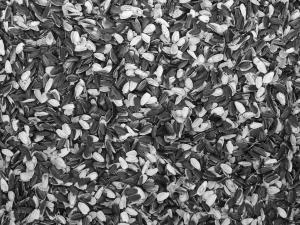

Supplement: Supplemental Information 2 [file peerj-cs-08-869-s002.zip › 0_part2/257_debris_other_0015_02_thumb.jpg]

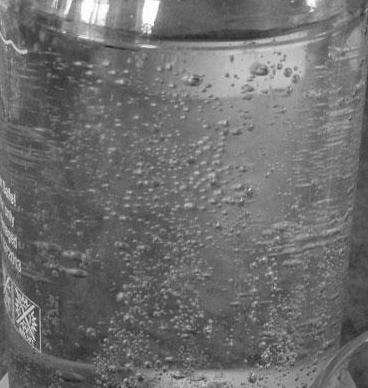

Supplement: Supplemental Information 2 [file peerj-cs-08-869-s002.zip › 0_part2/258_bubbly_0107.jpg]

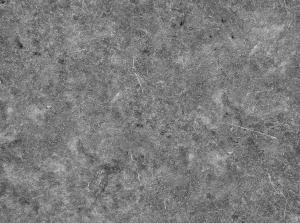

Supplement: Supplemental Information 2 [file peerj-cs-08-869-s002.zip › 0_part2/259_grass_grass_0013_01_thumb.jpg]

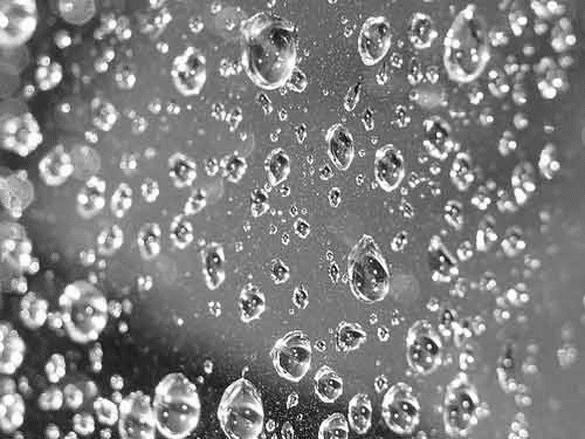

Supplement: Supplemental Information 2 [file peerj-cs-08-869-s002.zip › 0_part2/25_bubbly_0168.jpg]

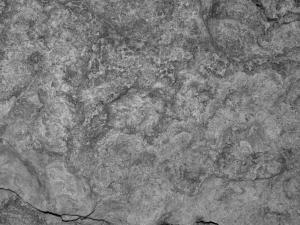

Supplement: Supplemental Information 2 [file peerj-cs-08-869-s002.zip › 0_part2/260_rock_cave_0005_01_thumb.jpg]

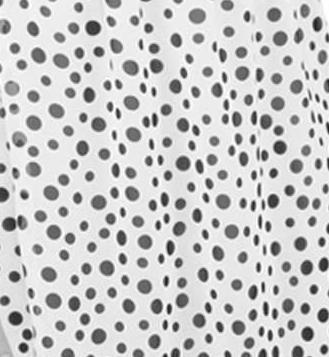

Supplement: Supplemental Information 2 [file peerj-cs-08-869-s002.zip › 0_part2/261_dotted_0164.jpg]

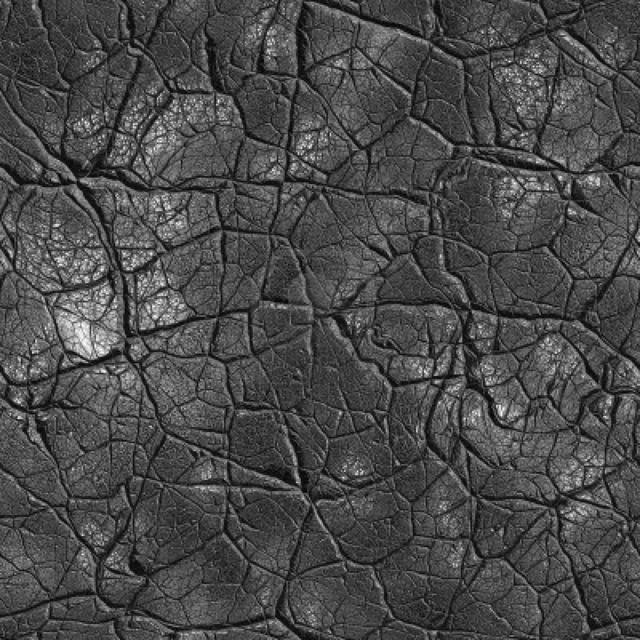

Supplement: Supplemental Information 2 [file peerj-cs-08-869-s002.zip › 0_part2/262_cracked_0095.jpg]

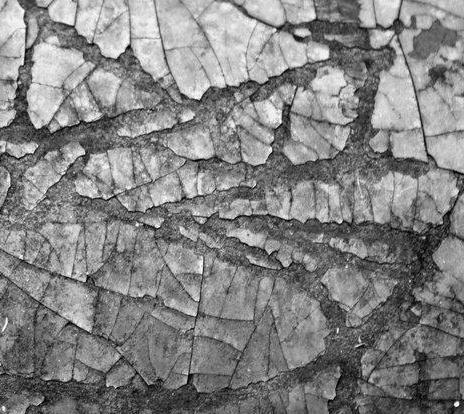

Supplement: Supplemental Information 2 [file peerj-cs-08-869-s002.zip › 0_part2/263_cracked_0104.jpg]

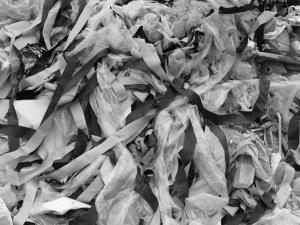

Supplement: Supplemental Information 2 [file peerj-cs-08-869-s002.zip › 0_part2/264_debris_other_0006_01_thumb.jpg]

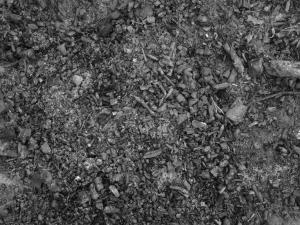

Supplement: Supplemental Information 2 [file peerj-cs-08-869-s002.zip › 0_part2/265_debris_wood_chips_0010_01_thumb.jpg]

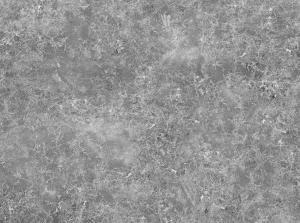

Supplement: Supplemental Information 2 [file peerj-cs-08-869-s002.zip › 0_part2/266_grass_grass_0009_01_thumb.jpg]

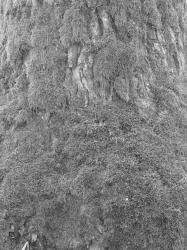

Supplement: Supplemental Information 2 [file peerj-cs-08-869-s002.zip › 0_part2/267_nature_moss_0022_01_thumb.jpg]

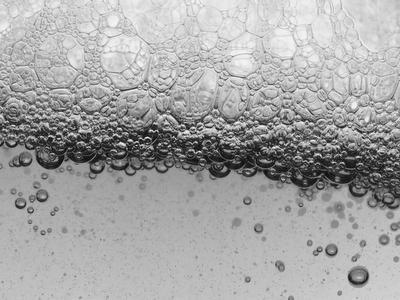

Supplement: Supplemental Information 2 [file peerj-cs-08-869-s002.zip › 0_part2/268_bubbly_0140.jpg]

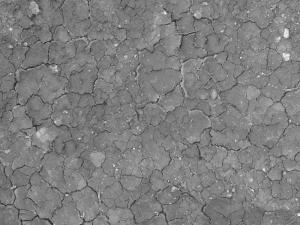

Supplement: Supplemental Information 2 [file peerj-cs-08-869-s002.zip › 0_part2/269_soil_cracked_0014_01_thumb.jpg]

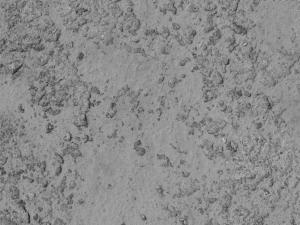

Supplement: Supplemental Information 2 [file peerj-cs-08-869-s002.zip › 0_part2/26_ground_stone_ground_0003_01_thumb.jpg]

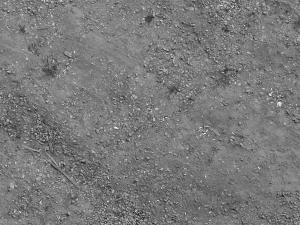

Supplement: Supplemental Information 2 [file peerj-cs-08-869-s002.zip › 0_part2/270_ground_ground_garbage_0003_01_thumb.jpg]

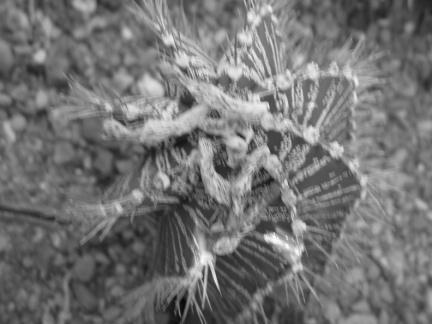

Supplement: Supplemental Information 2 [file peerj-cs-08-869-s002.zip › 0_part2/271_Flora31_46.jpg]

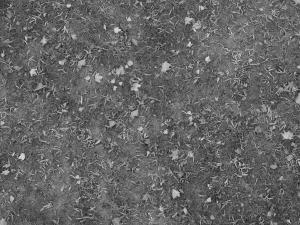

Supplement: Supplemental Information 2 [file peerj-cs-08-869-s002.zip › 0_part2/272_grass_leaves_0005_01_thumb.jpg]

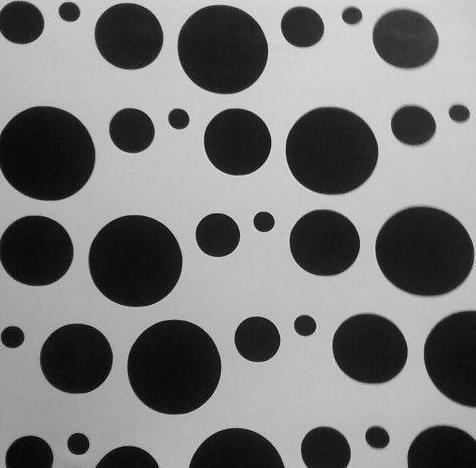

Supplement: Supplemental Information 2 [file peerj-cs-08-869-s002.zip › 0_part2/273_dotted_0112.jpg]

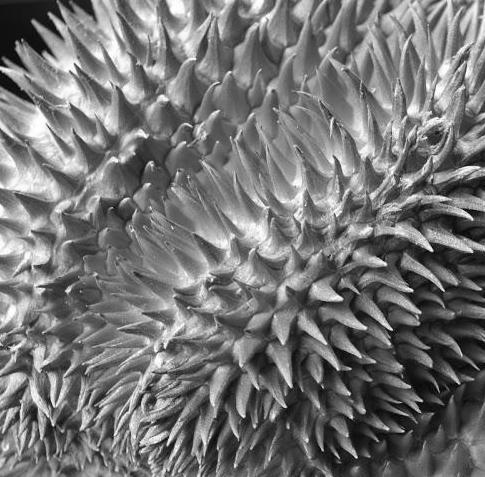

Supplement: Supplemental Information 2 [file peerj-cs-08-869-s002.zip › 0_part2/274_bumpy_0147.jpg]

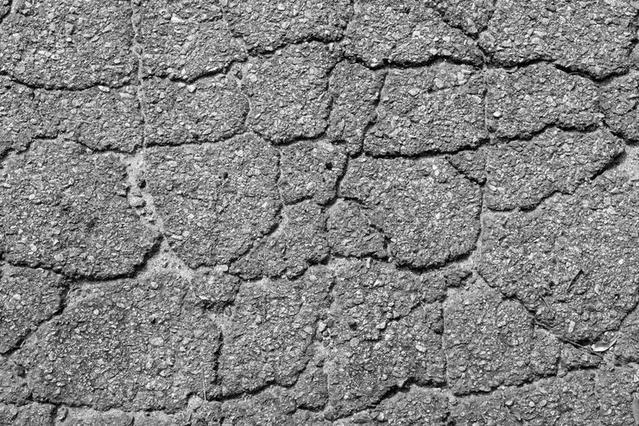

Supplement: Supplemental Information 2 [file peerj-cs-08-869-s002.zip › 0_part2/275_cracked_0065.jpg]

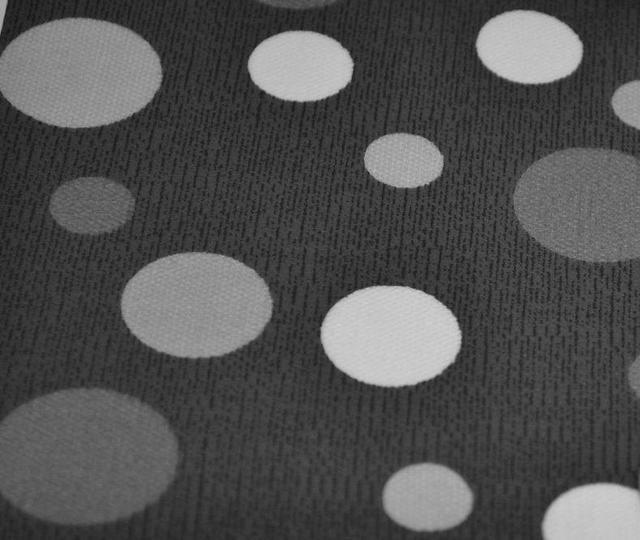

Supplement: Supplemental Information 2 [file peerj-cs-08-869-s002.zip › 0_part2/276_dotted_0146.jpg]

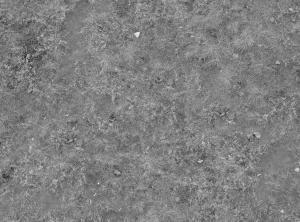

Supplement: Supplemental Information 2 [file peerj-cs-08-869-s002.zip › 0_part2/277_grass_grass_0011_01_thumb.jpg]

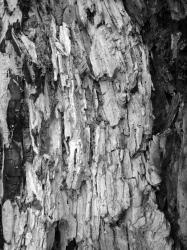

Supplement: Supplemental Information 2 [file peerj-cs-08-869-s002.zip › 0_part2/278_wood_rotten_0021_01_thumb.jpg]

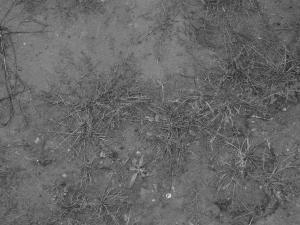

Supplement: Supplemental Information 2 [file peerj-cs-08-869-s002.zip › 0_part2/279_grass_other_grass_0029_02_thumb.jpg]

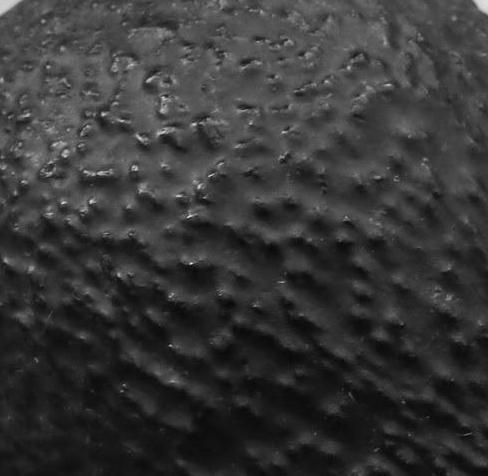

Supplement: Supplemental Information 2 [file peerj-cs-08-869-s002.zip › 0_part2/27_bumpy_0151.jpg]

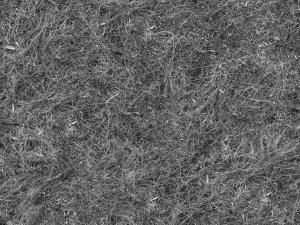

Supplement: Supplemental Information 2 [file peerj-cs-08-869-s002.zip › 0_part2/280_grass_grass_0035_01_thumb.jpg]

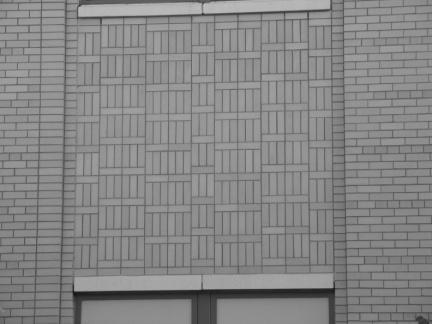

Supplement: Supplemental Information 3 [file peerj-cs-08-869-s003.zip › 1_part1/246_Pure Texture 171_102.jpg]
