# Supplementary figures and images for: Deep convolutional neural networks for regular texture recognition (part 3 of 8)
Source: PeerJ Comput Sci. 2022 Feb 9;8:e869. doi: 10.7717/peerj-cs.869 (PMC9044313; doi:10.7717/peerj-cs.869)

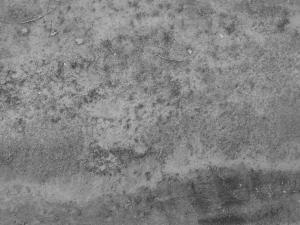

Supplement: Supplemental Information 2 [file peerj-cs-08-869-s002.zip › 0_part2/101_grass_other_grass_0027_01_thumb.jpg]

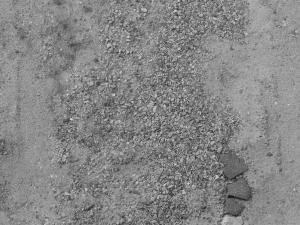

Supplement: Supplemental Information 2 [file peerj-cs-08-869-s002.zip › 0_part2/102_ground_stone_ground_0006_01_thumb.jpg]

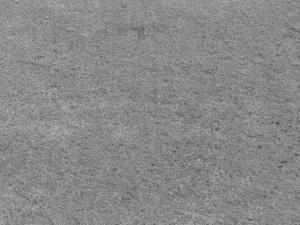

Supplement: Supplemental Information 2 [file peerj-cs-08-869-s002.zip › 0_part2/103_ground_slope_0030_01_thumb.jpg]

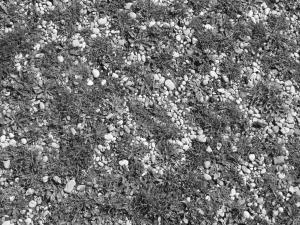

Supplement: Supplemental Information 2 [file peerj-cs-08-869-s002.zip › 0_part2/104_grass_on_stones_0022_01_thumb.jpg]

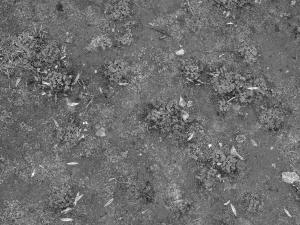

Supplement: Supplemental Information 2 [file peerj-cs-08-869-s002.zip › 0_part2/105_grass_leaves_0025_01_thumb.jpg]

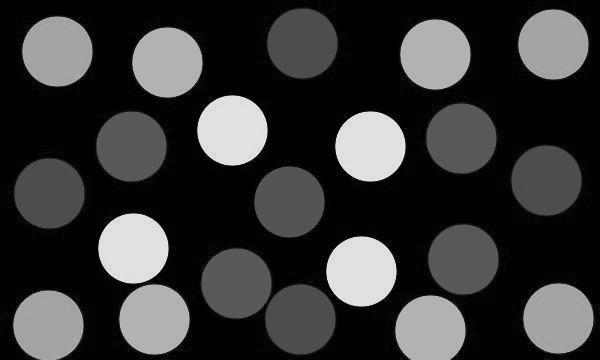

Supplement: Supplemental Information 2 [file peerj-cs-08-869-s002.zip › 0_part2/106_dotted_0156.jpg]

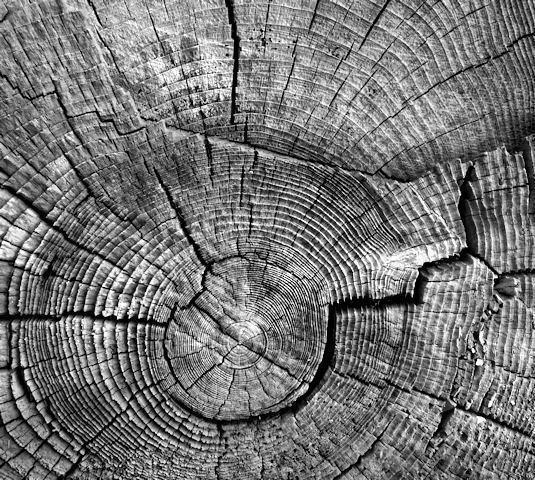

Supplement: Supplemental Information 2 [file peerj-cs-08-869-s002.zip › 0_part2/107_cracked_0156.jpg]

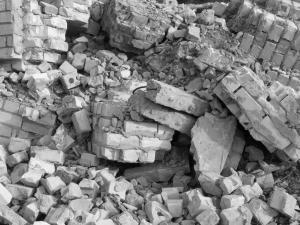

Supplement: Supplemental Information 2 [file peerj-cs-08-869-s002.zip › 0_part2/108_debris_other_0017_01_thumb.jpg]

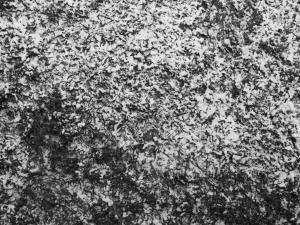

Supplement: Supplemental Information 2 [file peerj-cs-08-869-s002.zip › 0_part2/109_ground_frozen_ground_0003_01_thumb.jpg]

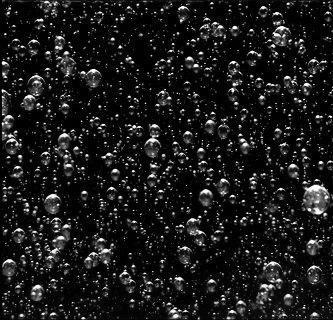

Supplement: Supplemental Information 2 [file peerj-cs-08-869-s002.zip › 0_part2/10_bubbly_0095.jpg]

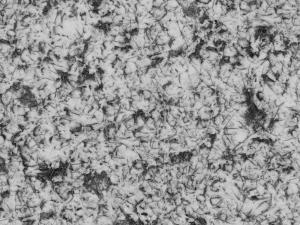

Supplement: Supplemental Information 2 [file peerj-cs-08-869-s002.zip › 0_part2/110_ground_frozen_ground_0046_01_thumb.jpg]

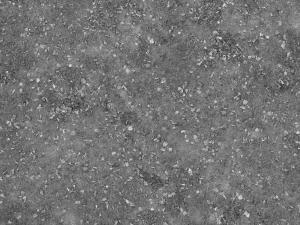

Supplement: Supplemental Information 2 [file peerj-cs-08-869-s002.zip › 0_part2/111_grass_leaves_0008_01_thumb.jpg]

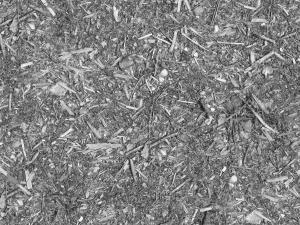

Supplement: Supplemental Information 2 [file peerj-cs-08-869-s002.zip › 0_part2/112_debris_wood_chips_0014_01_thumb.jpg]

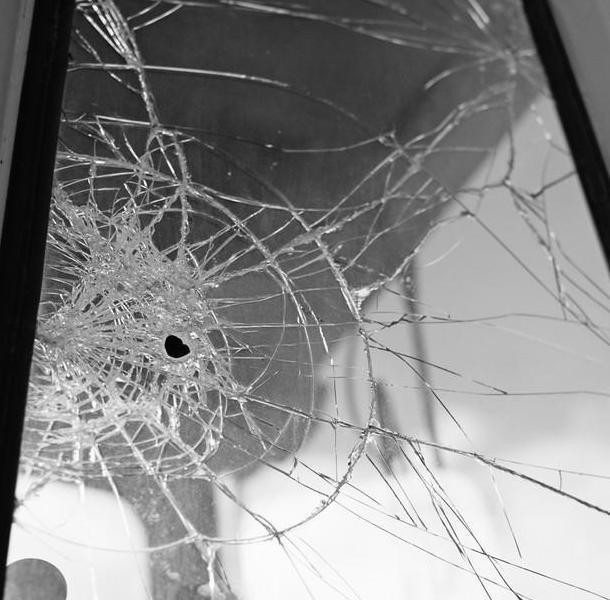

Supplement: Supplemental Information 2 [file peerj-cs-08-869-s002.zip › 0_part2/113_cracked_0134.jpg]

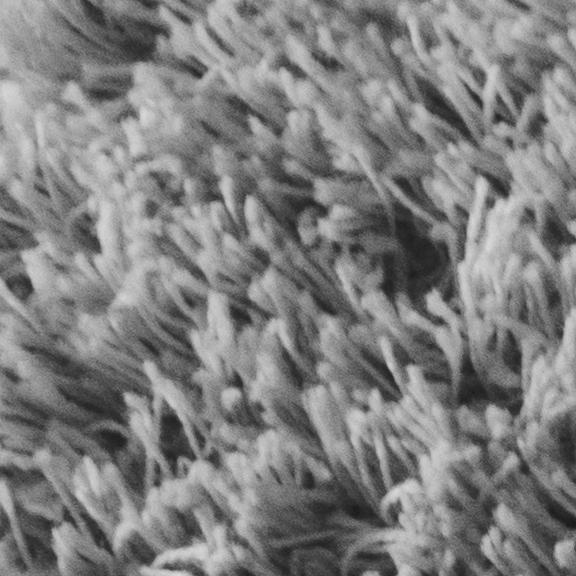

Supplement: Supplemental Information 2 [file peerj-cs-08-869-s002.zip › 0_part2/114_rug1-a-p002.jpg]

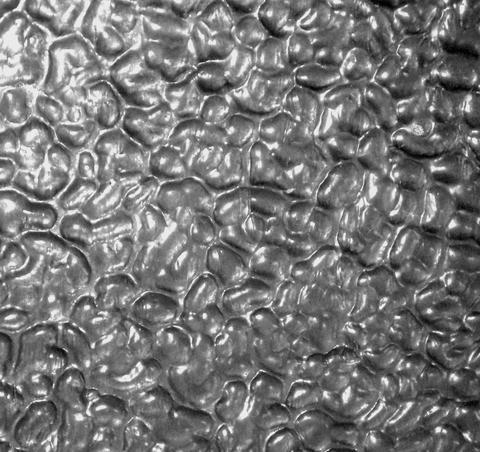

Supplement: Supplemental Information 2 [file peerj-cs-08-869-s002.zip › 0_part2/115_bubbly_0083.jpg]

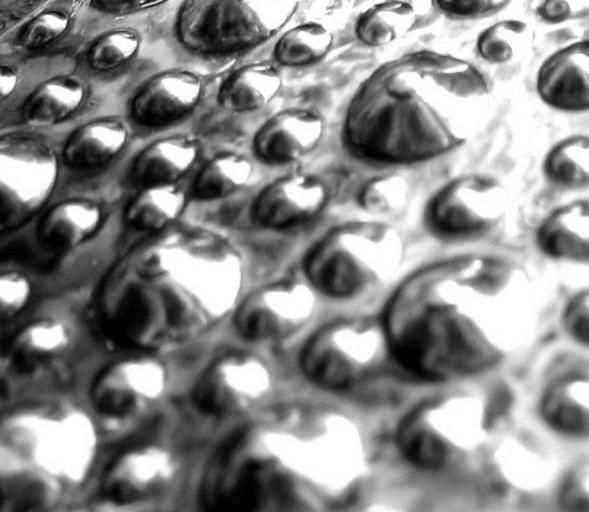

Supplement: Supplemental Information 2 [file peerj-cs-08-869-s002.zip › 0_part2/116_bumpy_0191.jpg]

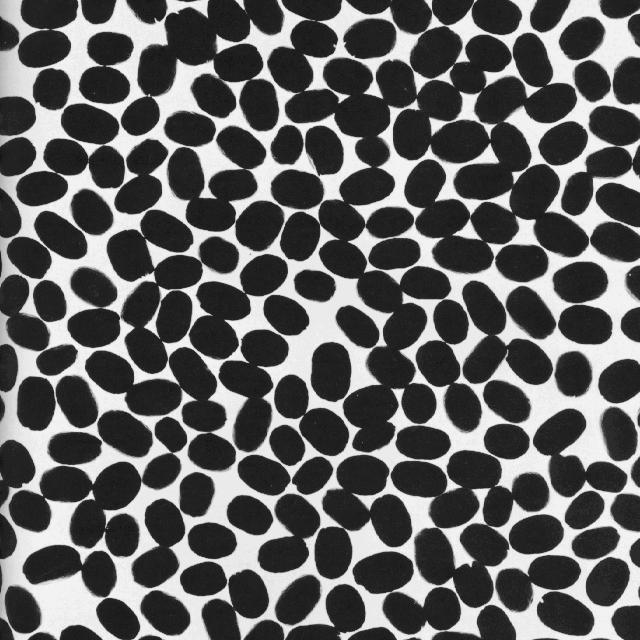

Supplement: Supplemental Information 2 [file peerj-cs-08-869-s002.zip › 0_part2/117_D75.jpg]

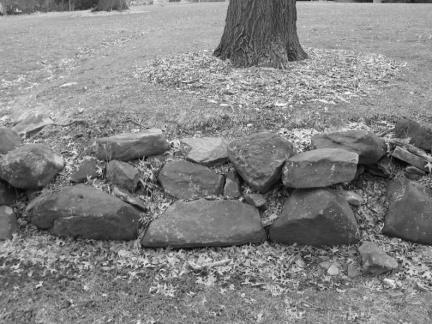

Supplement: Supplemental Information 2 [file peerj-cs-08-869-s002.zip › 0_part2/118_Borderline Near-Regular Textures 65_51.jpg]

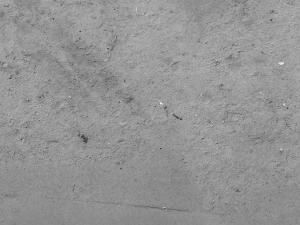

Supplement: Supplemental Information 2 [file peerj-cs-08-869-s002.zip › 0_part2/119_ground_other_ground_0004_01_thumb.jpg]

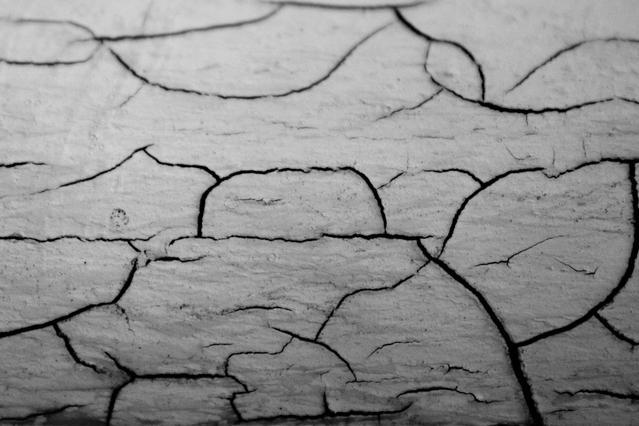

Supplement: Supplemental Information 2 [file peerj-cs-08-869-s002.zip › 0_part2/11_cracked_0060.jpg]

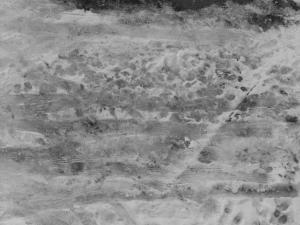

Supplement: Supplemental Information 2 [file peerj-cs-08-869-s002.zip › 0_part2/120_ground_frozen_ground_0061_01_thumb.jpg]

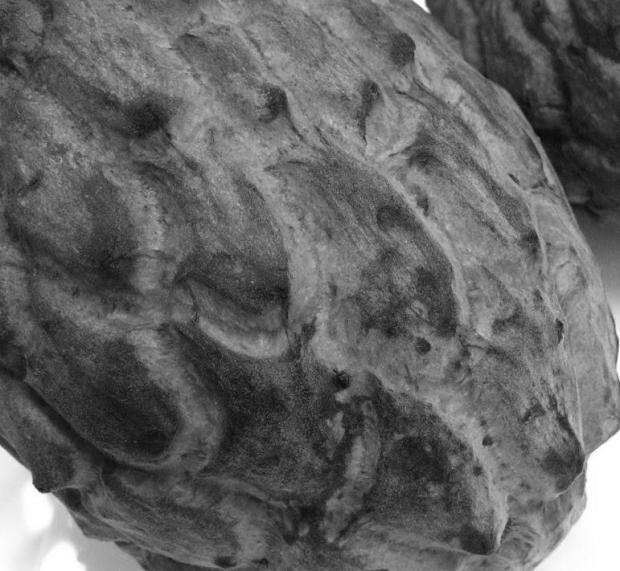

Supplement: Supplemental Information 2 [file peerj-cs-08-869-s002.zip › 0_part2/121_bumpy_0113.jpg]

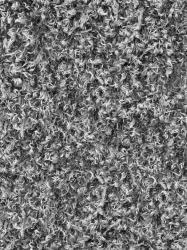

Supplement: Supplemental Information 2 [file peerj-cs-08-869-s002.zip › 0_part2/122_nature_moss_0024_01_thumb.jpg]

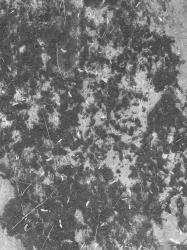

Supplement: Supplemental Information 2 [file peerj-cs-08-869-s002.zip › 0_part2/123_nature_moss_0019_01_thumb.jpg]

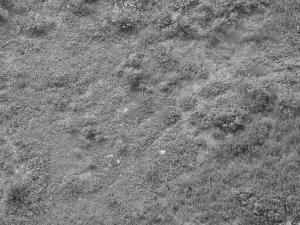

Supplement: Supplemental Information 2 [file peerj-cs-08-869-s002.zip › 0_part2/124_grass_grass_0097_01_thumb.jpg]

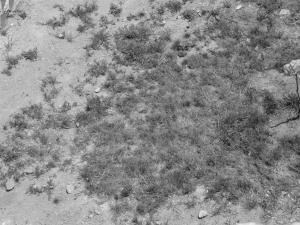

Supplement: Supplemental Information 2 [file peerj-cs-08-869-s002.zip › 0_part2/125_grass_other_grass_0039_01_thumb.jpg]

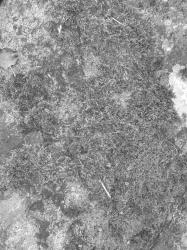

Supplement: Supplemental Information 2 [file peerj-cs-08-869-s002.zip › 0_part2/126_nature_moss_0039_01_thumb.jpg]

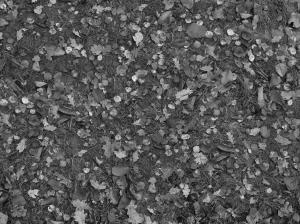

Supplement: Supplemental Information 2 [file peerj-cs-08-869-s002.zip › 0_part2/127_grass_leaves_0011_01_thumb.jpg]

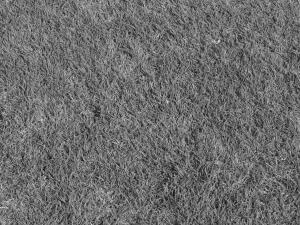

Supplement: Supplemental Information 2 [file peerj-cs-08-869-s002.zip › 0_part2/128_grass_grass_0115_01_thumb.jpg]

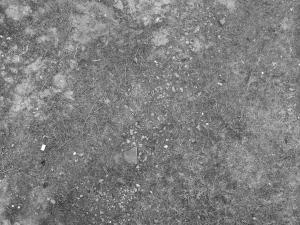

Supplement: Supplemental Information 2 [file peerj-cs-08-869-s002.zip › 0_part2/129_grass_on_stones_0011_01_thumb.jpg]

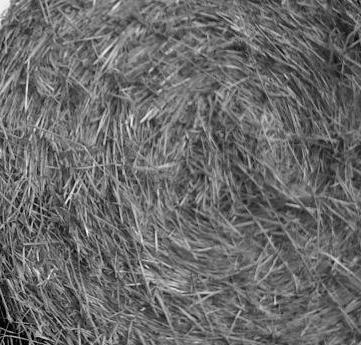

Supplement: Supplemental Information 2 [file peerj-cs-08-869-s002.zip › 0_part2/12_fibrous_0120.jpg]

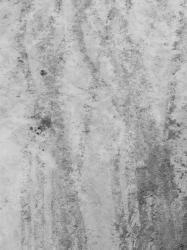

Supplement: Supplemental Information 2 [file peerj-cs-08-869-s002.zip › 0_part2/130_ground_frozen_ground_0007_01_thumb.jpg]

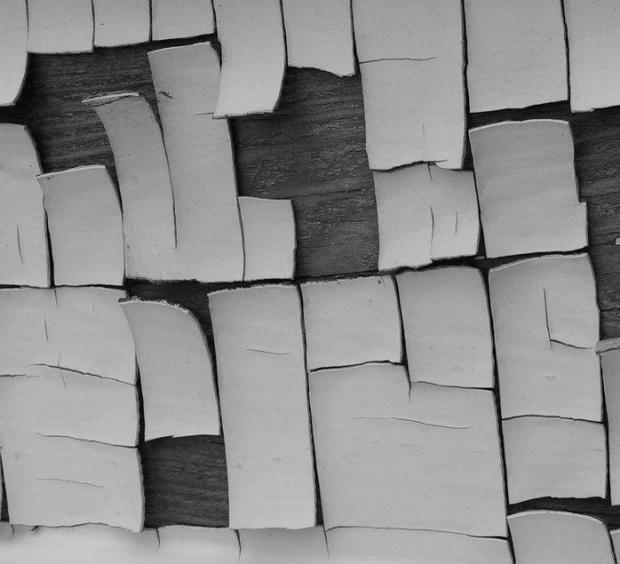

Supplement: Supplemental Information 2 [file peerj-cs-08-869-s002.zip › 0_part2/131_cracked_0151.jpg]

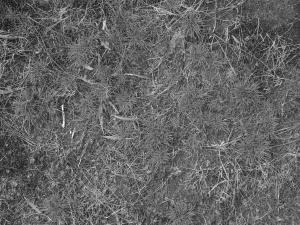

Supplement: Supplemental Information 2 [file peerj-cs-08-869-s002.zip › 0_part2/132_grass_grass_0038_01_thumb.jpg]

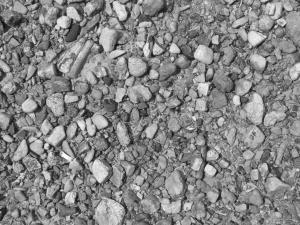

Supplement: Supplemental Information 2 [file peerj-cs-08-869-s002.zip › 0_part2/133_debris_stone_debris_0053_01_thumb.jpg]

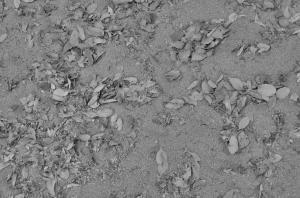

Supplement: Supplemental Information 2 [file peerj-cs-08-869-s002.zip › 0_part2/134_ground_ground_leaves_0044_01_thumb.jpg]

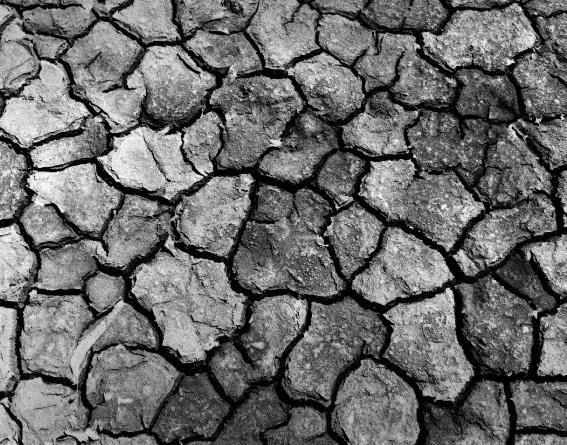

Supplement: Supplemental Information 2 [file peerj-cs-08-869-s002.zip › 0_part2/135_cracked_0063.jpg]

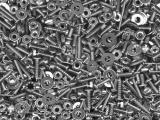

Supplement: Supplemental Information 2 [file peerj-cs-08-869-s002.zip › 0_part2/136_S_S_Metal05l.jpg]

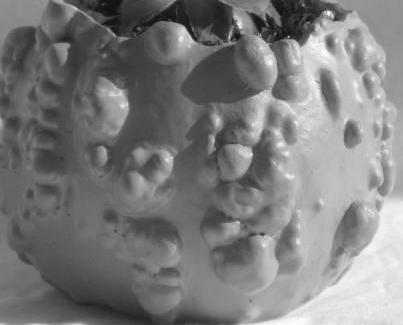

Supplement: Supplemental Information 2 [file peerj-cs-08-869-s002.zip › 0_part2/137_bumpy_0128.jpg]

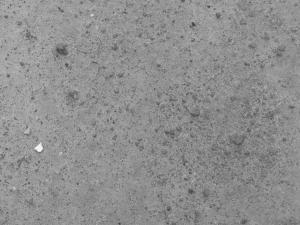

Supplement: Supplemental Information 2 [file peerj-cs-08-869-s002.zip › 0_part2/138_ground_stone_ground_0024_01_thumb.jpg]

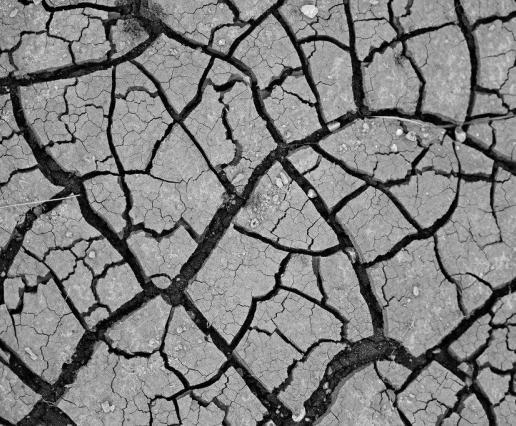

Supplement: Supplemental Information 2 [file peerj-cs-08-869-s002.zip › 0_part2/139_cracked_0122.jpg]

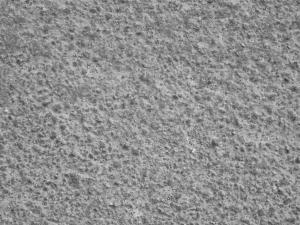

Supplement: Supplemental Information 2 [file peerj-cs-08-869-s002.zip › 0_part2/13_ground_stone_ground_0011_01_thumb.jpg]

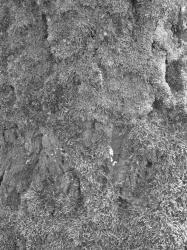

Supplement: Supplemental Information 2 [file peerj-cs-08-869-s002.zip › 0_part2/140_nature_moss_0017_01_thumb.jpg]

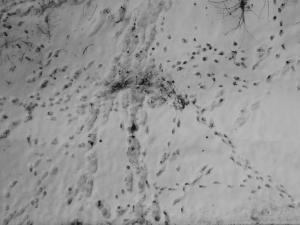

Supplement: Supplemental Information 2 [file peerj-cs-08-869-s002.zip › 0_part2/141_ground_frozen_ground_0053_04_thumb.jpg]

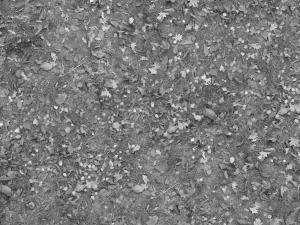

Supplement: Supplemental Information 2 [file peerj-cs-08-869-s002.zip › 0_part2/142_grass_leaves_0031_01_thumb.jpg]

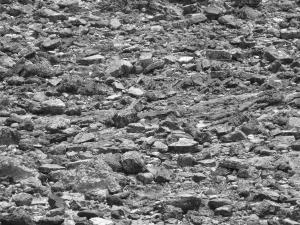

Supplement: Supplemental Information 2 [file peerj-cs-08-869-s002.zip › 0_part2/143_debris_stone_debris_0054_01_thumb.jpg]

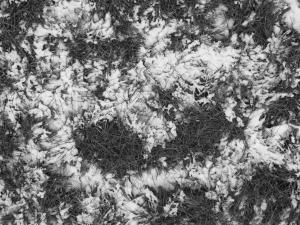

Supplement: Supplemental Information 2 [file peerj-cs-08-869-s002.zip › 0_part2/144_ground_frozen_ground_0022_01_thumb.jpg]

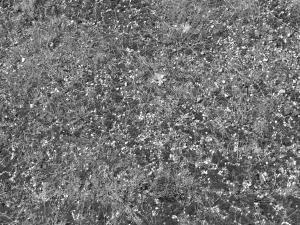

Supplement: Supplemental Information 2 [file peerj-cs-08-869-s002.zip › 0_part2/145_grass_on_stones_0028_01_thumb.jpg]

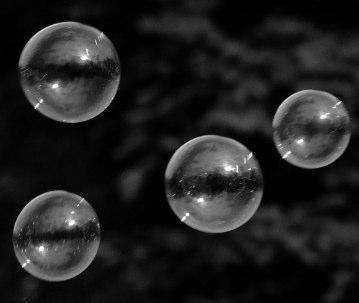

Supplement: Supplemental Information 2 [file peerj-cs-08-869-s002.zip › 0_part2/146_bubbly_0152.jpg]

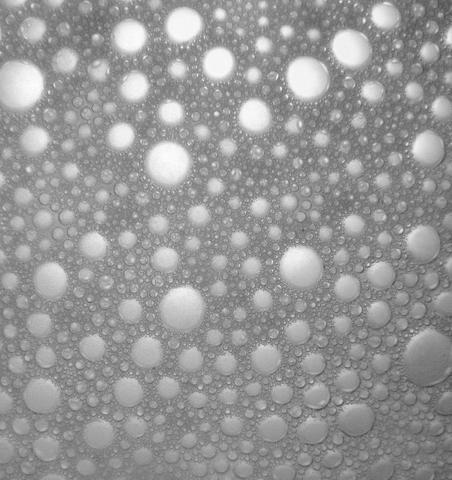

Supplement: Supplemental Information 2 [file peerj-cs-08-869-s002.zip › 0_part2/147_bubbly_0077.jpg]

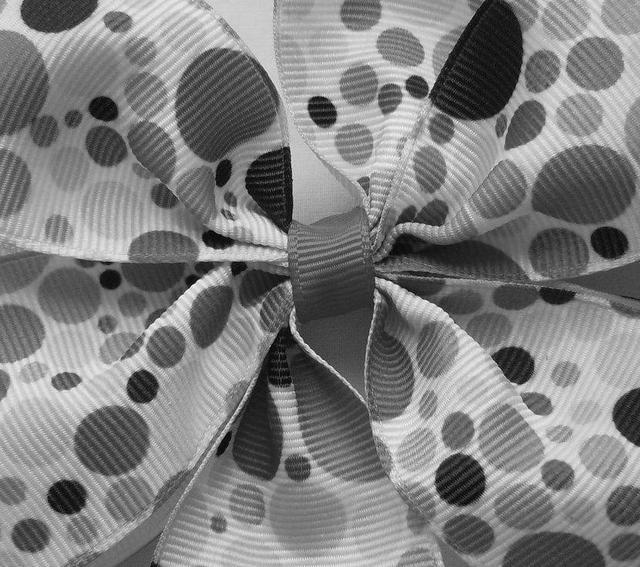

Supplement: Supplemental Information 2 [file peerj-cs-08-869-s002.zip › 0_part2/148_dotted_0157.jpg]

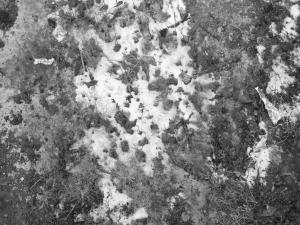

Supplement: Supplemental Information 2 [file peerj-cs-08-869-s002.zip › 0_part2/149_ground_frozen_ground_0008_01_thumb.jpg]

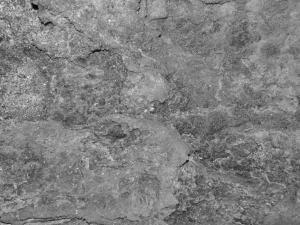

Supplement: Supplemental Information 2 [file peerj-cs-08-869-s002.zip › 0_part2/14_rock_cave_0014_01_thumb.jpg]

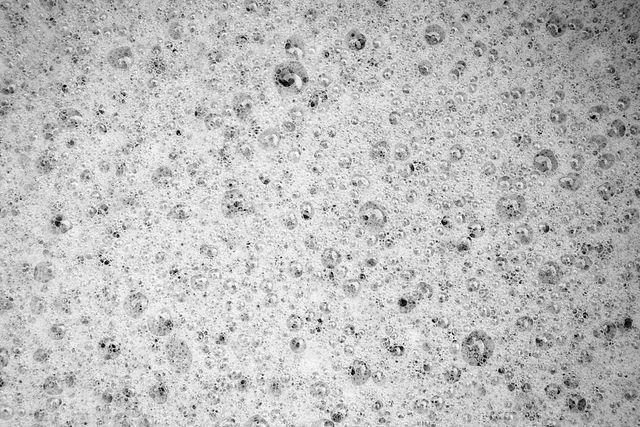

Supplement: Supplemental Information 2 [file peerj-cs-08-869-s002.zip › 0_part2/150_bubbly_0160.jpg]

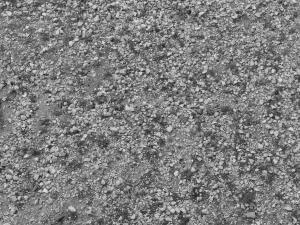

Supplement: Supplemental Information 2 [file peerj-cs-08-869-s002.zip › 0_part2/151_grass_on_stones_0029_01_thumb.jpg]

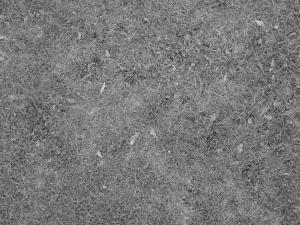

Supplement: Supplemental Information 2 [file peerj-cs-08-869-s002.zip › 0_part2/152_grass_grass_0085_01_thumb.jpg]

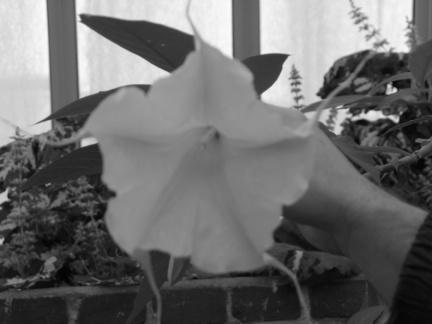

Supplement: Supplemental Information 2 [file peerj-cs-08-869-s002.zip › 0_part2/153_Flora31_23.jpg]

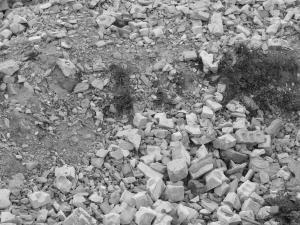

Supplement: Supplemental Information 2 [file peerj-cs-08-869-s002.zip › 0_part2/154_debris_stone_debris_0038_01_thumb.jpg]

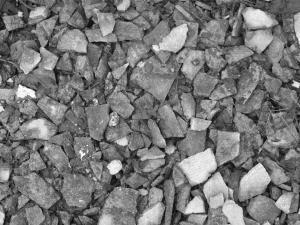

Supplement: Supplemental Information 2 [file peerj-cs-08-869-s002.zip › 0_part2/155_debris_stone_debris_0016_01_thumb.jpg]

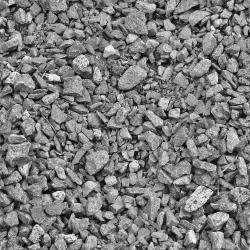

Supplement: Supplemental Information 2 [file peerj-cs-08-869-s002.zip › 0_part2/156_ground_pebble_0009_01_thumb.jpg]

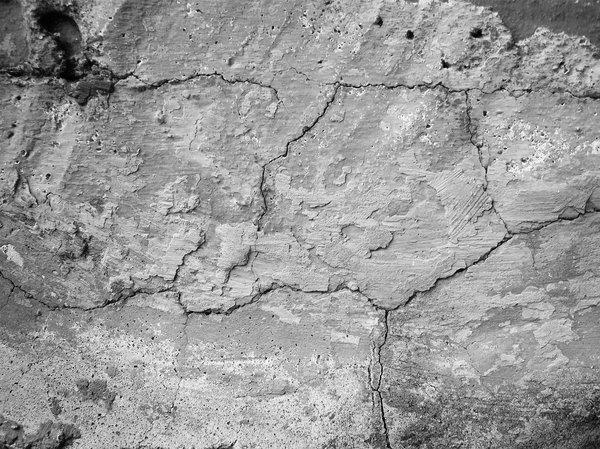

Supplement: Supplemental Information 2 [file peerj-cs-08-869-s002.zip › 0_part2/157_cracked_0107.jpg]

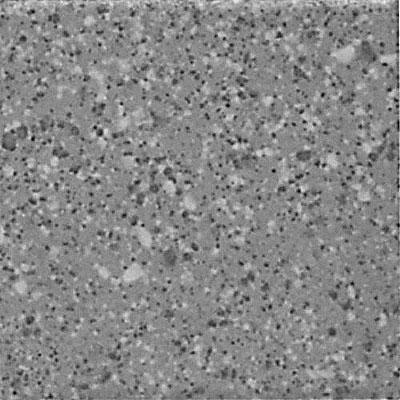

Supplement: Supplemental Information 2 [file peerj-cs-08-869-s002.zip › 0_part2/158_flecked_0042.jpg]

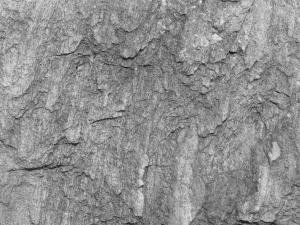

Supplement: Supplemental Information 2 [file peerj-cs-08-869-s002.zip › 0_part2/159_rock_cave_0030_01_thumb.jpg]

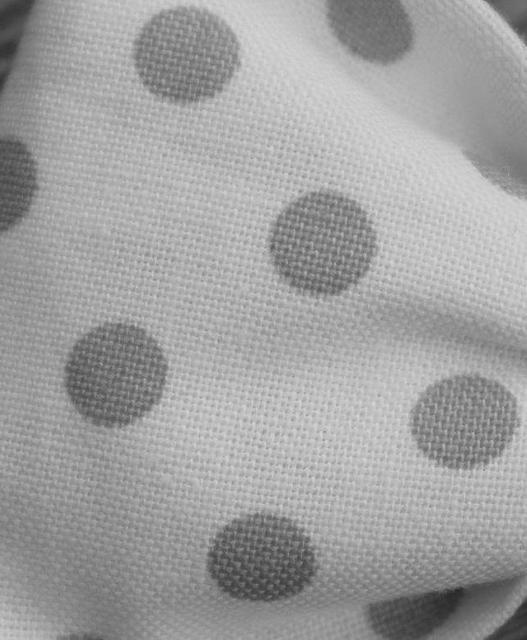

Supplement: Supplemental Information 2 [file peerj-cs-08-869-s002.zip › 0_part2/15_dotted_0108.jpg]

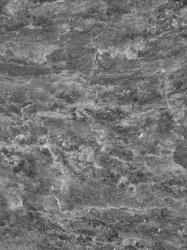

Supplement: Supplemental Information 2 [file peerj-cs-08-869-s002.zip › 0_part2/160_rock_cave_0022_01_thumb.jpg]

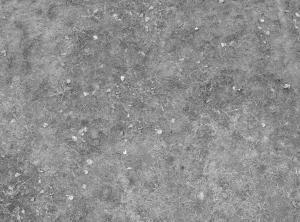

Supplement: Supplemental Information 2 [file peerj-cs-08-869-s002.zip › 0_part2/161_grass_grass_0010_01_thumb.jpg]

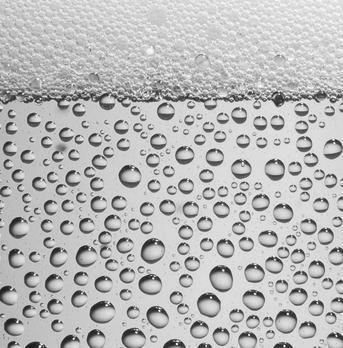

Supplement: Supplemental Information 2 [file peerj-cs-08-869-s002.zip › 0_part2/162_bubbly_0061.jpg]

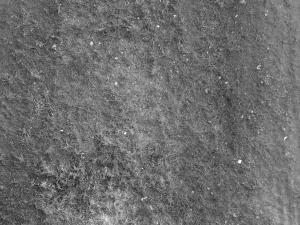

Supplement: Supplemental Information 2 [file peerj-cs-08-869-s002.zip › 0_part2/163_grass_grass_0023_01_thumb.jpg]

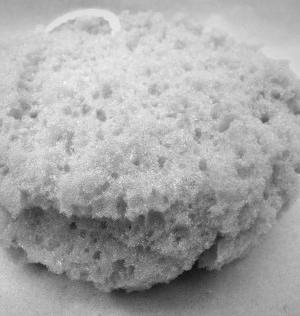

Supplement: Supplemental Information 2 [file peerj-cs-08-869-s002.zip › 0_part2/164_porous_0044.jpg]

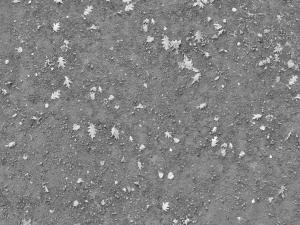

Supplement: Supplemental Information 2 [file peerj-cs-08-869-s002.zip › 0_part2/165_ground_ground_leaves_0029_01_thumb.jpg]

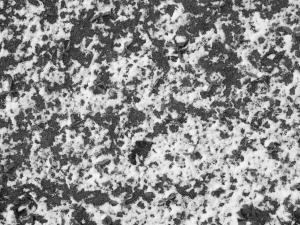

Supplement: Supplemental Information 2 [file peerj-cs-08-869-s002.zip › 0_part2/166_ground_frozen_ground_0044_01_thumb.jpg]

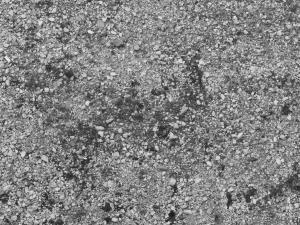

Supplement: Supplemental Information 2 [file peerj-cs-08-869-s002.zip › 0_part2/167_ground_stone_ground_0070_01_thumb.jpg]

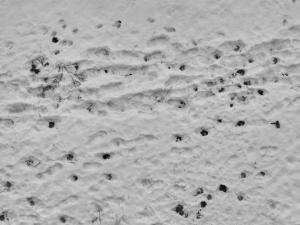

Supplement: Supplemental Information 2 [file peerj-cs-08-869-s002.zip › 0_part2/168_ground_frozen_ground_0055_01_thumb.jpg]

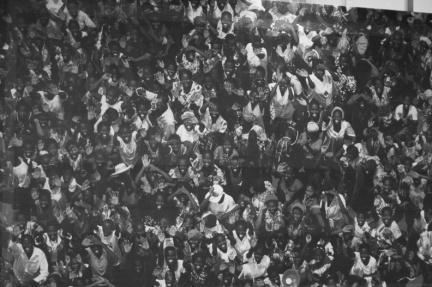

Supplement: Supplemental Information 2 [file peerj-cs-08-869-s002.zip › 0_part2/169_Borderline Near-Regular Textures 65_14.jpg]

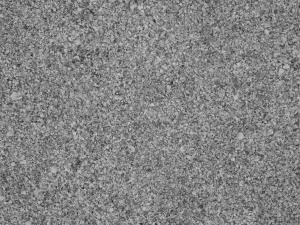

Supplement: Supplemental Information 2 [file peerj-cs-08-869-s002.zip › 0_part2/16_ground_pebble_0020_01_thumb.jpg]

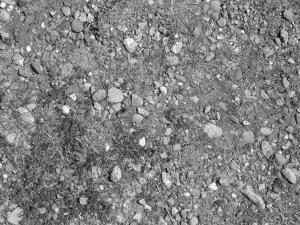

Supplement: Supplemental Information 2 [file peerj-cs-08-869-s002.zip › 0_part2/170_grass_on_stones_0012_01_thumb.jpg]

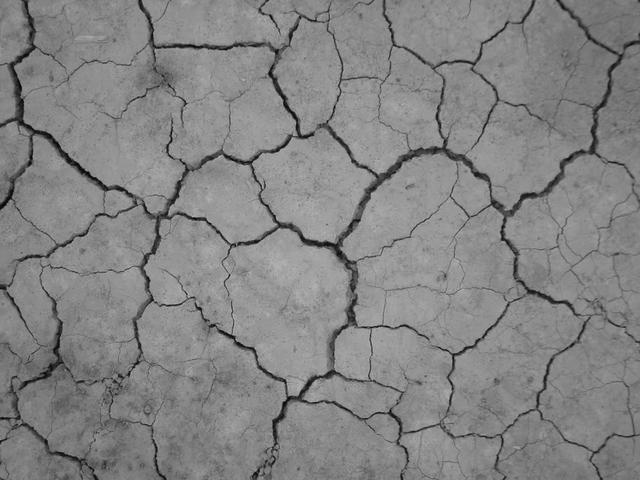

Supplement: Supplemental Information 2 [file peerj-cs-08-869-s002.zip › 0_part2/171_cracked_0085.jpg]

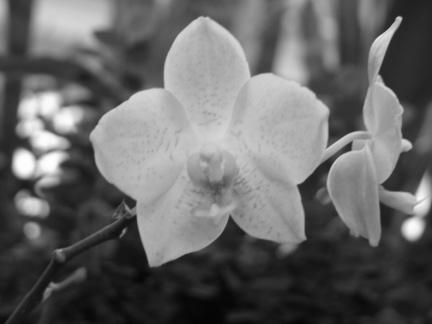

Supplement: Supplemental Information 2 [file peerj-cs-08-869-s002.zip › 0_part2/172_Flora31_66.jpg]

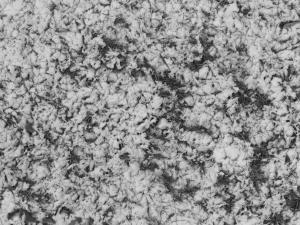

Supplement: Supplemental Information 2 [file peerj-cs-08-869-s002.zip › 0_part2/173_ground_frozen_ground_0045_01_thumb.jpg]

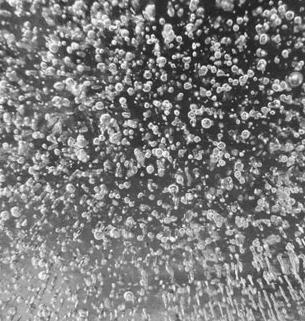

Supplement: Supplemental Information 2 [file peerj-cs-08-869-s002.zip › 0_part2/174_bubbly_0084.jpg]

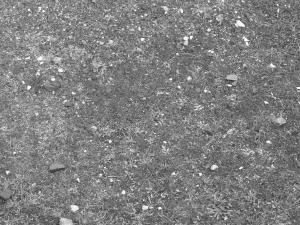

Supplement: Supplemental Information 2 [file peerj-cs-08-869-s002.zip › 0_part2/175_grass_on_stones_0026_01_thumb.jpg]

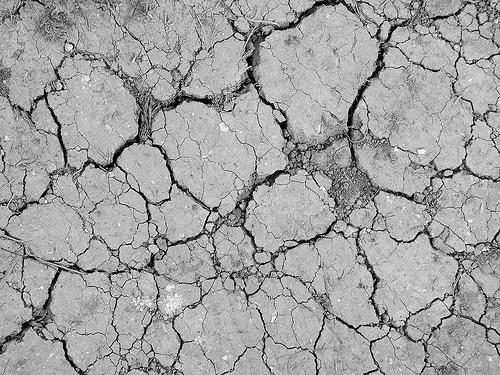

Supplement: Supplemental Information 2 [file peerj-cs-08-869-s002.zip › 0_part2/176_cracked_0046.jpg]

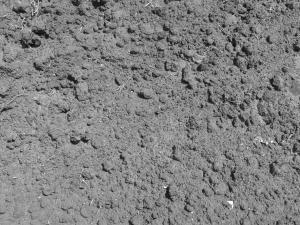

Supplement: Supplemental Information 2 [file peerj-cs-08-869-s002.zip › 0_part2/177_soil_ground_0077_01_thumb.jpg]

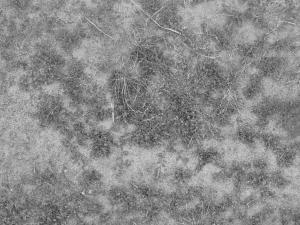

Supplement: Supplemental Information 2 [file peerj-cs-08-869-s002.zip › 0_part2/178_grass_grass_0062_01_thumb.jpg]

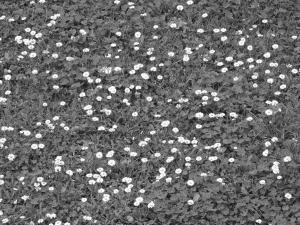

Supplement: Supplemental Information 2 [file peerj-cs-08-869-s002.zip › 0_part2/179_grass_grass_0126_01_thumb.jpg]

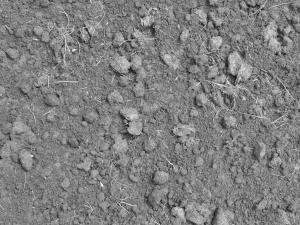

Supplement: Supplemental Information 2 [file peerj-cs-08-869-s002.zip › 0_part2/17_soil_ground_0078_01_thumb.jpg]

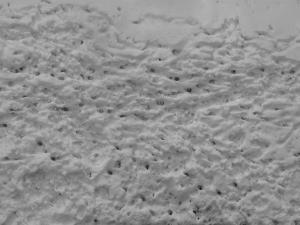

Supplement: Supplemental Information 2 [file peerj-cs-08-869-s002.zip › 0_part2/180_ground_frozen_ground_0060_01_thumb.jpg]

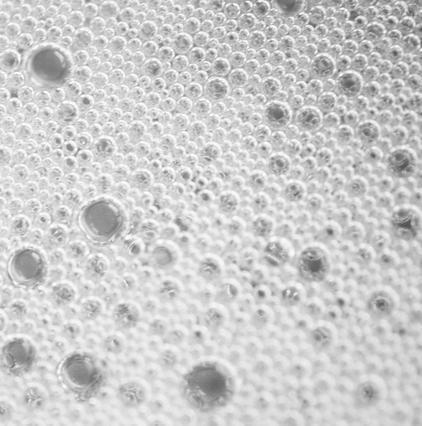

Supplement: Supplemental Information 2 [file peerj-cs-08-869-s002.zip › 0_part2/181_bubbly_0045.jpg]

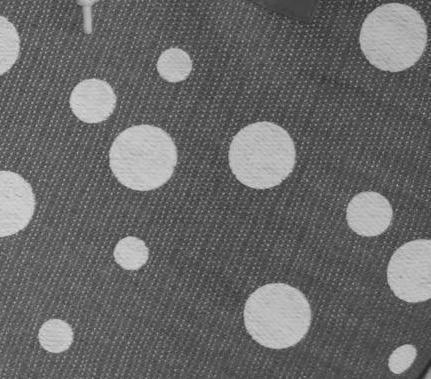

Supplement: Supplemental Information 2 [file peerj-cs-08-869-s002.zip › 0_part2/182_dotted_0159.jpg]

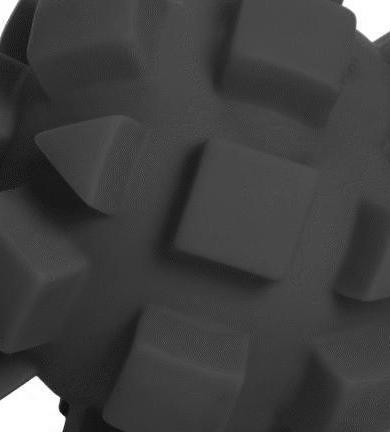

Supplement: Supplemental Information 2 [file peerj-cs-08-869-s002.zip › 0_part2/183_bumpy_0125.jpg]

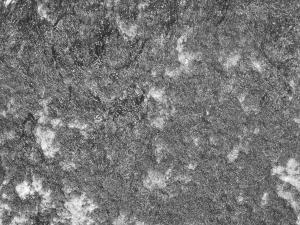

Supplement: Supplemental Information 2 [file peerj-cs-08-869-s002.zip › 0_part2/184_nature_moss_0051_01_thumb.jpg]

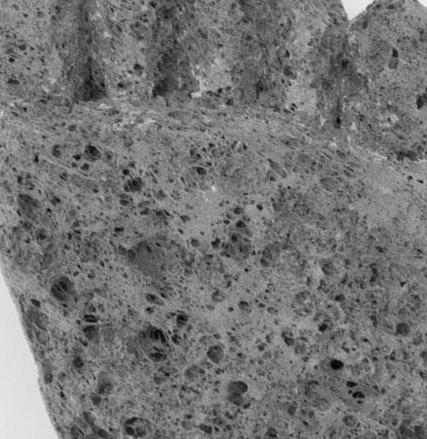

Supplement: Supplemental Information 2 [file peerj-cs-08-869-s002.zip › 0_part2/185_porous_0154.jpg]

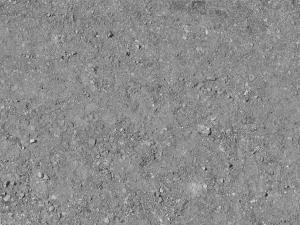

Supplement: Supplemental Information 2 [file peerj-cs-08-869-s002.zip › 0_part2/186_ground_stone_ground_0019_01_thumb.jpg]

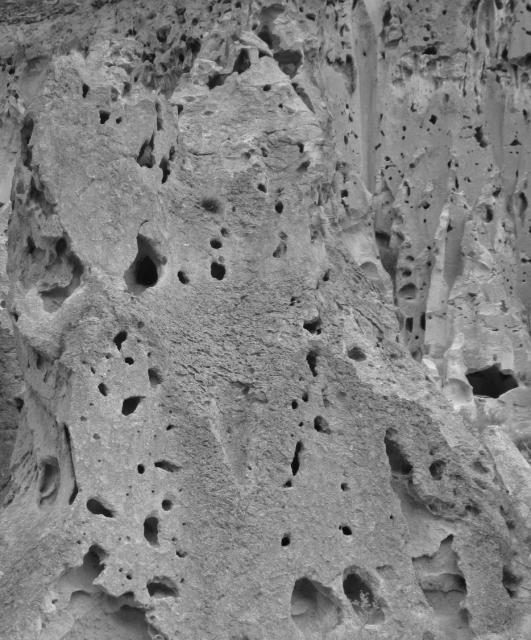

Supplement: Supplemental Information 2 [file peerj-cs-08-869-s002.zip › 0_part2/187_porous_0160.jpg]

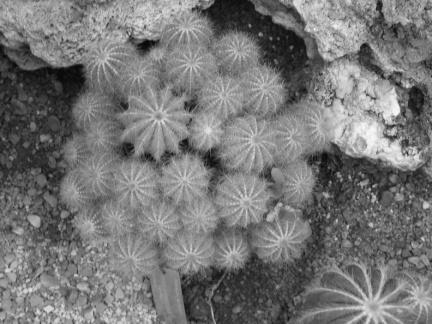

Supplement: Supplemental Information 2 [file peerj-cs-08-869-s002.zip › 0_part2/188_Flora31_36.jpg]

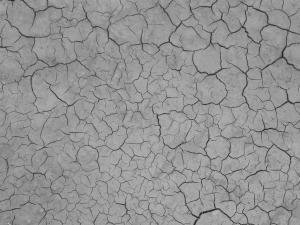

Supplement: Supplemental Information 2 [file peerj-cs-08-869-s002.zip › 0_part2/189_soil_cracked_0040_01_thumb.jpg]

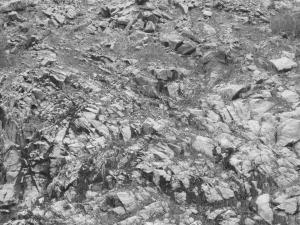

Supplement: Supplemental Information 2 [file peerj-cs-08-869-s002.zip › 0_part2/18_ground_slope_0043_01_thumb.jpg]

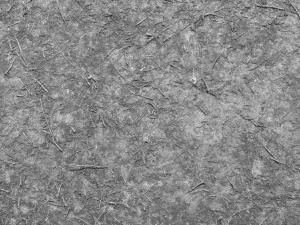

Supplement: Supplemental Information 2 [file peerj-cs-08-869-s002.zip › 0_part2/190_debris_wood_chips_0012_01_thumb.jpg]

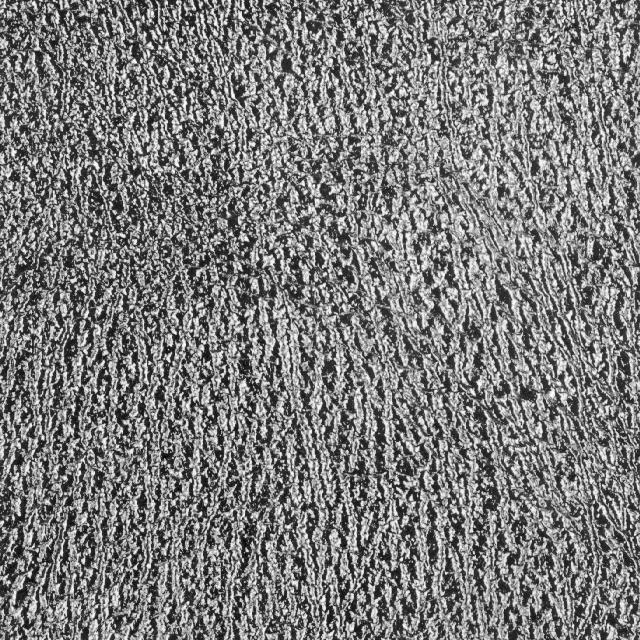

Supplement: Supplemental Information 2 [file peerj-cs-08-869-s002.zip › 0_part2/191_D24.jpg]
