# Supplementary figures and images for: Deep convolutional neural networks for regular texture recognition (part 6 of 8)
Source: PeerJ Comput Sci. 2022 Feb 9;8:e869. doi: 10.7717/peerj-cs.869 (PMC9044313; doi:10.7717/peerj-cs.869)

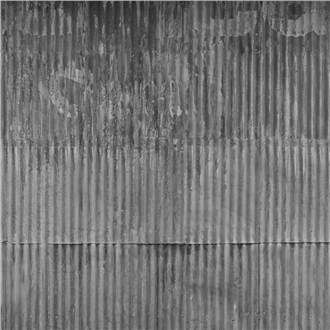

Supplement: Supplemental Information 3 [file peerj-cs-08-869-s003.zip › 1_part1/297_ooved_0047.jpg]

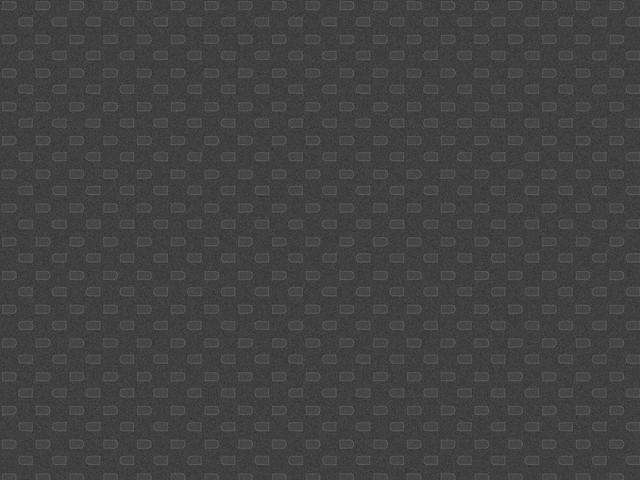

Supplement: Supplemental Information 3 [file peerj-cs-08-869-s003.zip › 1_part1/297_page13_11.jpg]

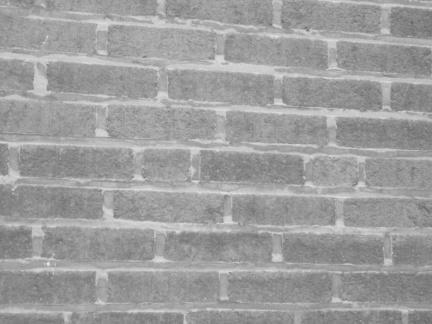

Supplement: Supplemental Information 3 [file peerj-cs-08-869-s003.zip › 1_part1/298_Pure Texture 171_60.jpg]

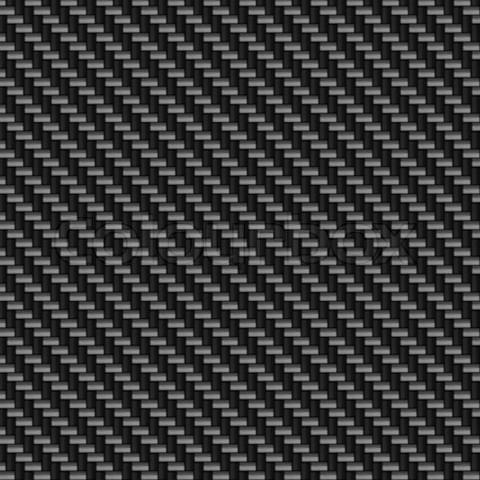

Supplement: Supplemental Information 3 [file peerj-cs-08-869-s003.zip › 1_part1/298_ven_0039.jpg]

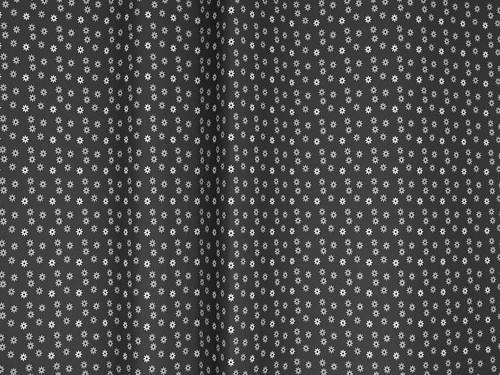

Supplement: Supplemental Information 3 [file peerj-cs-08-869-s003.zip › 1_part1/299_page13_4.jpg]

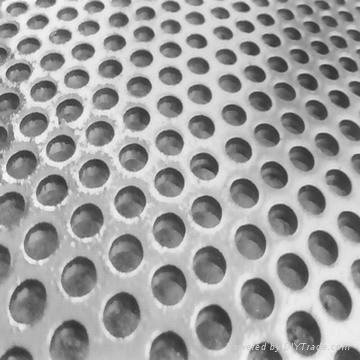

Supplement: Supplemental Information 3 [file peerj-cs-08-869-s003.zip › 1_part1/299_rforated_0042.jpg]

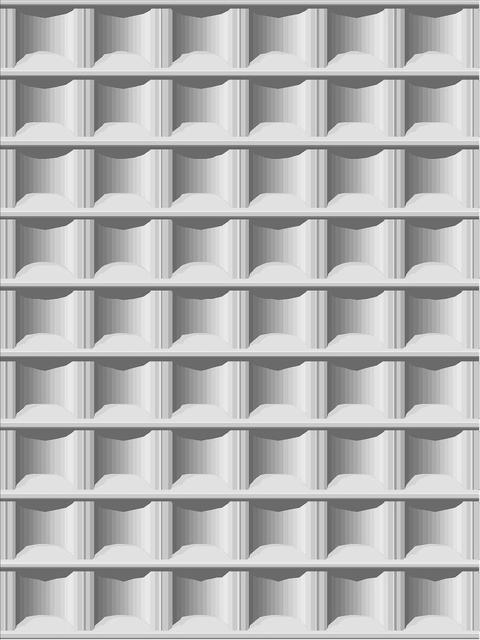

Supplement: Supplemental Information 3 [file peerj-cs-08-869-s003.zip › 1_part1/300_ffled_0090.jpg]

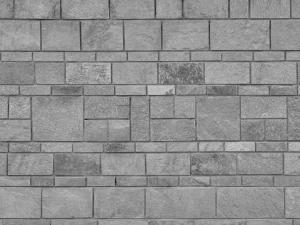

Supplement: Supplemental Information 3 [file peerj-cs-08-869-s003.zip › 1_part1/300_stone_wall_87.jpg]

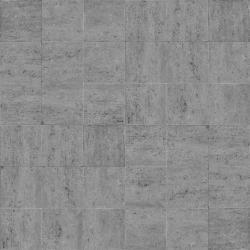

Supplement: Supplemental Information 3 [file peerj-cs-08-869-s003.zip › 1_part1/301_brick_pavement_48.jpg]

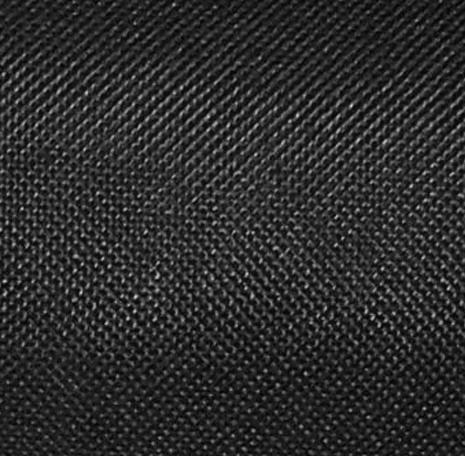

Supplement: Supplemental Information 3 [file peerj-cs-08-869-s003.zip › 1_part1/301_ven_0101.jpg]

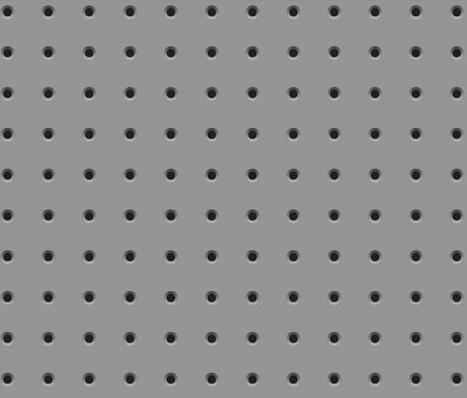

Supplement: Supplemental Information 3 [file peerj-cs-08-869-s003.zip › 1_part1/302_rforated_0045.jpg]

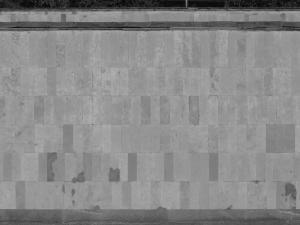

Supplement: Supplemental Information 3 [file peerj-cs-08-869-s003.zip › 1_part1/302_tile_tile_8.jpg]

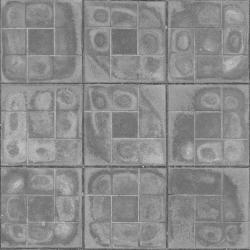

Supplement: Supplemental Information 3 [file peerj-cs-08-869-s003.zip › 1_part1/303_brick_pavement_29.jpg]

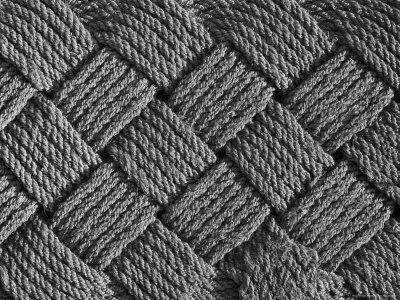

Supplement: Supplemental Information 3 [file peerj-cs-08-869-s003.zip › 1_part1/303_ven_0064.jpg]

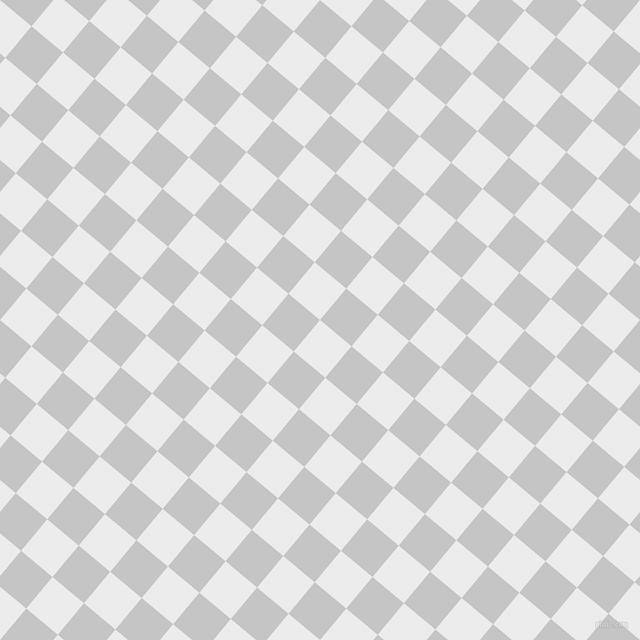

Supplement: Supplemental Information 3 [file peerj-cs-08-869-s003.zip › 1_part1/304_equered_0052.jpg]

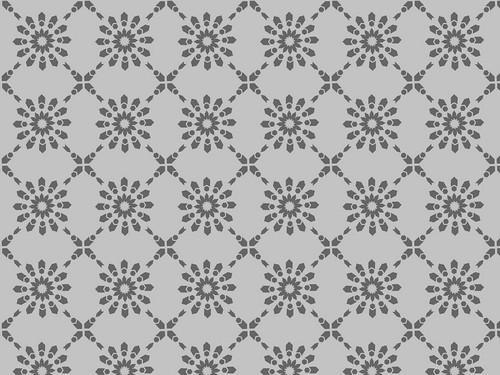

Supplement: Supplemental Information 3 [file peerj-cs-08-869-s003.zip › 1_part1/304_page10_0.jpg]

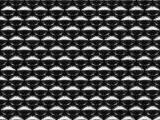

Supplement: Supplemental Information 3 [file peerj-cs-08-869-s003.zip › 1_part1/305_S_S_Beebees.jpg]

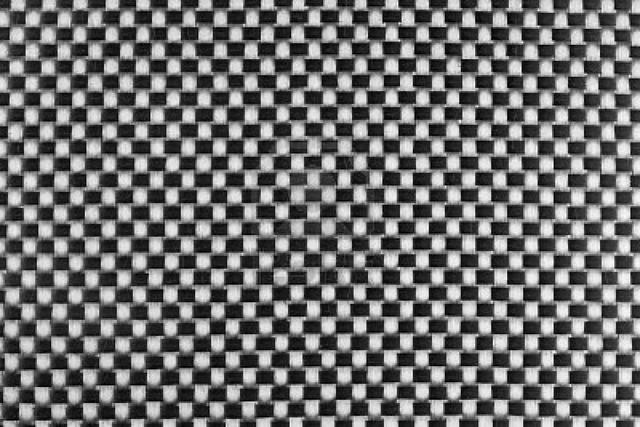

Supplement: Supplemental Information 3 [file peerj-cs-08-869-s003.zip › 1_part1/305_ven_0094.jpg]

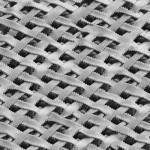

Supplement: Supplemental Information 3 [file peerj-cs-08-869-s003.zip › 1_part1/306_Graph cut texture synthesis results 92_25.jpg]

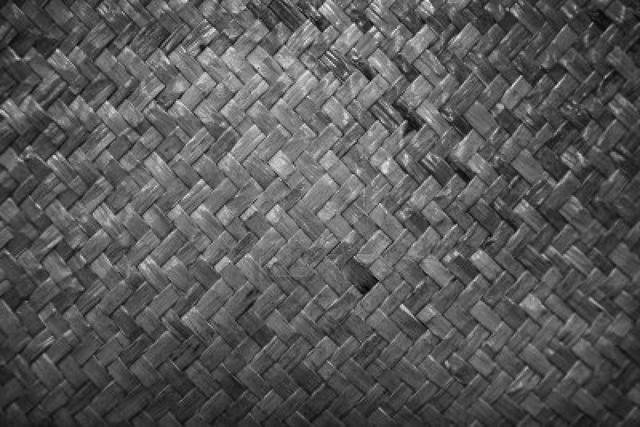

Supplement: Supplemental Information 3 [file peerj-cs-08-869-s003.zip › 1_part1/306_ven_0048.jpg]

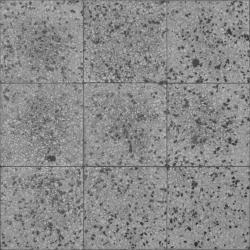

Supplement: Supplemental Information 3 [file peerj-cs-08-869-s003.zip › 1_part1/307_brick_pavement_17.jpg]

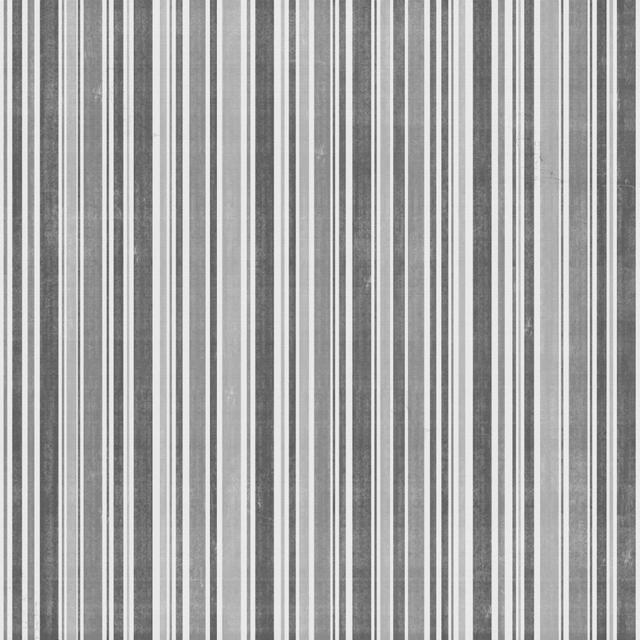

Supplement: Supplemental Information 3 [file peerj-cs-08-869-s003.zip › 1_part1/307_nded_0021.jpg]

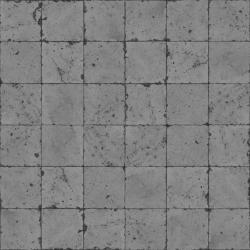

Supplement: Supplemental Information 3 [file peerj-cs-08-869-s003.zip › 1_part1/308_brick_pavement_36.jpg]

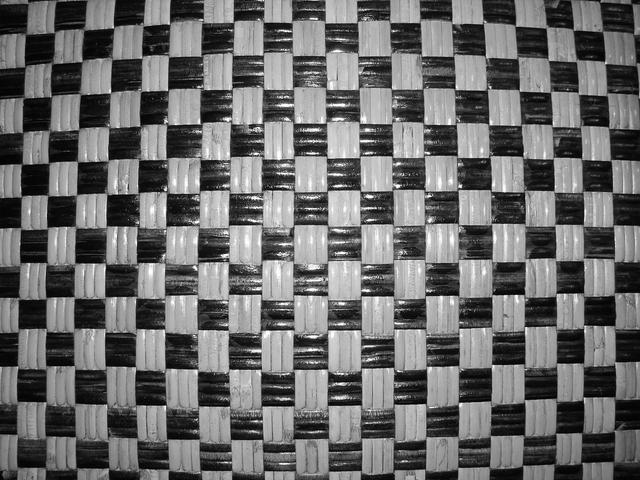

Supplement: Supplemental Information 3 [file peerj-cs-08-869-s003.zip › 1_part1/308_ven_0061.jpg]

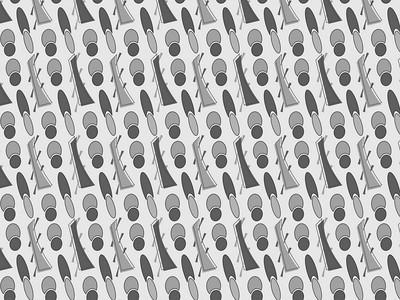

Supplement: Supplemental Information 3 [file peerj-cs-08-869-s003.zip › 1_part1/309_48479216701_5191d6984b_w.jpg]

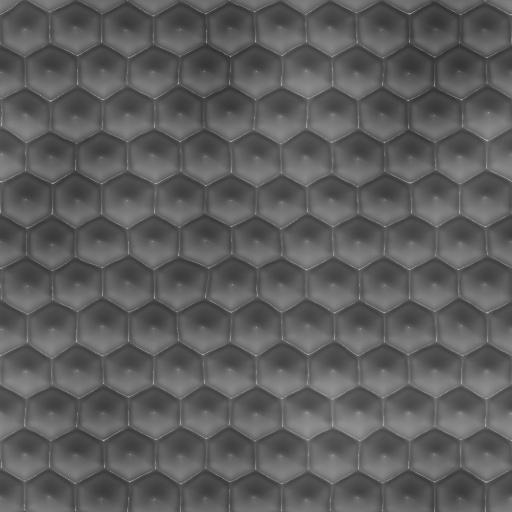

Supplement: Supplemental Information 3 [file peerj-cs-08-869-s003.zip › 1_part1/309_neycombed_0058.jpg]

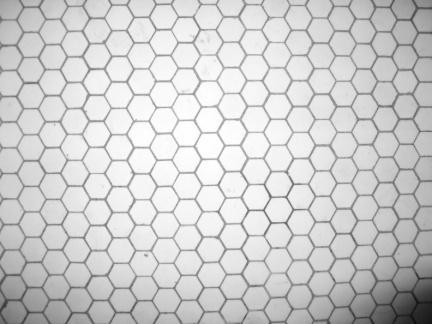

Supplement: Supplemental Information 3 [file peerj-cs-08-869-s003.zip › 1_part1/310_New Regular Textures 15_7.jpg]

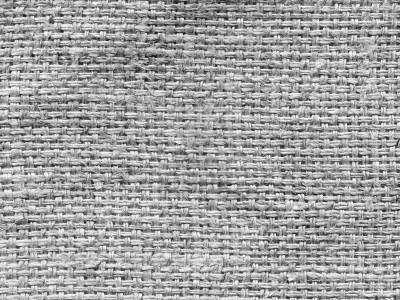

Supplement: Supplemental Information 3 [file peerj-cs-08-869-s003.zip › 1_part1/310_ven_0122.jpg]

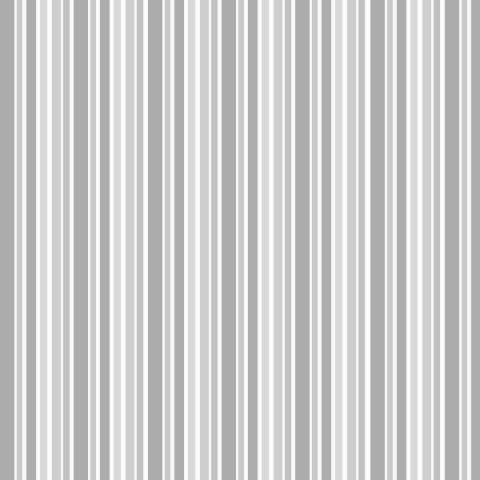

Supplement: Supplemental Information 3 [file peerj-cs-08-869-s003.zip › 1_part1/311_nded_0033.jpg]

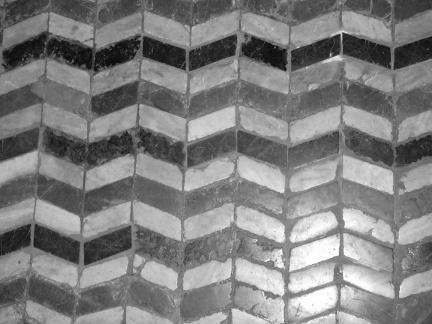

Supplement: Supplemental Information 3 [file peerj-cs-08-869-s003.zip › 1_part1/311_Pure Texture 171_15.jpg]

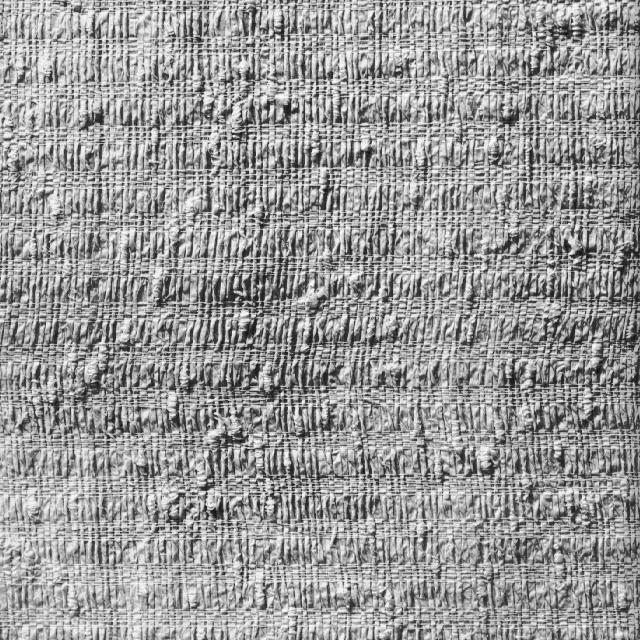

Supplement: Supplemental Information 3 [file peerj-cs-08-869-s003.zip › 1_part1/312_D80.jpg]

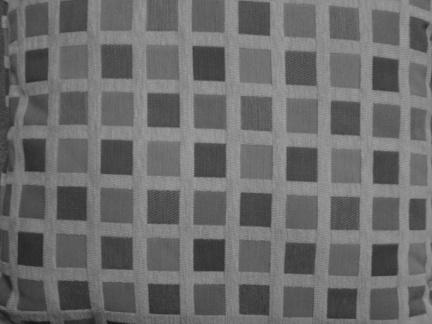

Supplement: Supplemental Information 3 [file peerj-cs-08-869-s003.zip › 1_part1/313_Pure Texture 171_86.jpg]

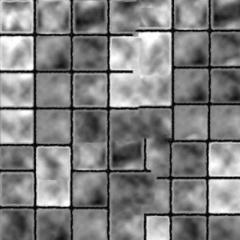

Supplement: Supplemental Information 3 [file peerj-cs-08-869-s003.zip › 1_part1/314_Graph cut texture synthesis results 92_19.jpg]

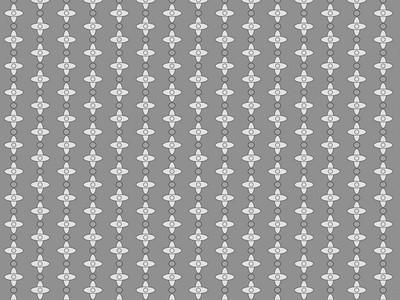

Supplement: Supplemental Information 3 [file peerj-cs-08-869-s003.zip › 1_part1/315_47084136744_a38c6bd401_w.jpg]

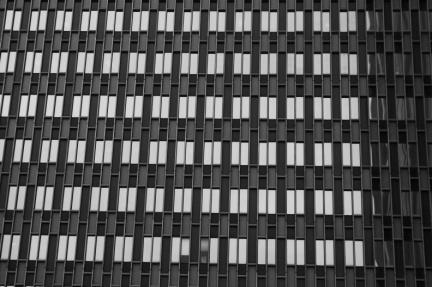

Supplement: Supplemental Information 3 [file peerj-cs-08-869-s003.zip › 1_part1/316_Pure Texture 171_57.jpg]

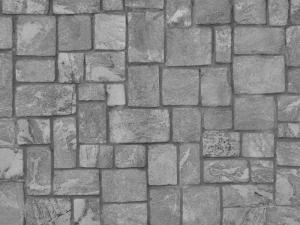

Supplement: Supplemental Information 3 [file peerj-cs-08-869-s003.zip › 1_part1/317_stone_wall_39.jpg]

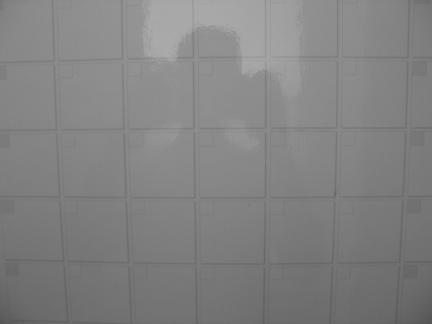

Supplement: Supplemental Information 3 [file peerj-cs-08-869-s003.zip › 1_part1/318_Pure Texture 171_96.jpg]

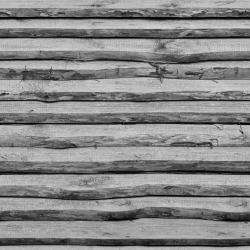

Supplement: Supplemental Information 3 [file peerj-cs-08-869-s003.zip › 1_part1/319_Planks old_42.jpg]

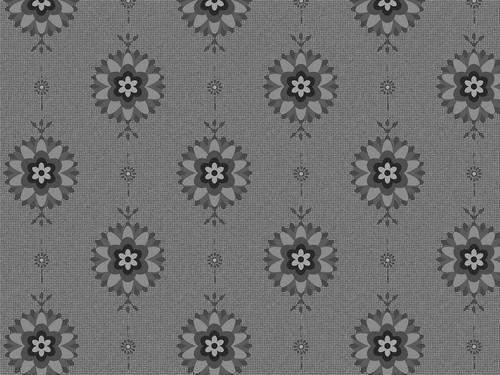

Supplement: Supplemental Information 3 [file peerj-cs-08-869-s003.zip › 1_part1/320_page7_4.jpg]

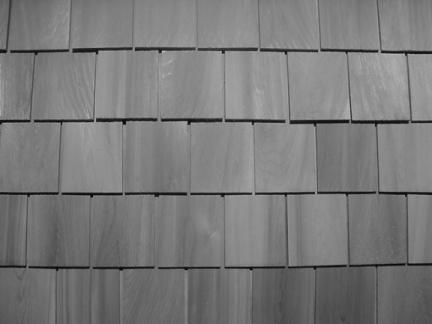

Supplement: Supplemental Information 3 [file peerj-cs-08-869-s003.zip › 1_part1/321_New Regular Textures 15_12.jpg]

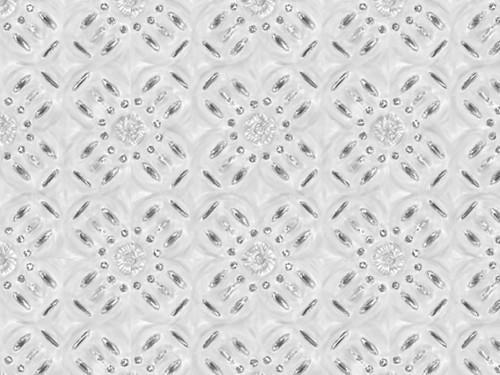

Supplement: Supplemental Information 3 [file peerj-cs-08-869-s003.zip › 1_part1/322_page8_11.jpg]

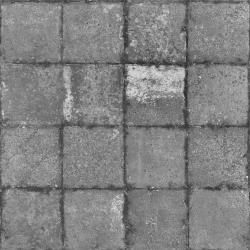

Supplement: Supplemental Information 3 [file peerj-cs-08-869-s003.zip › 1_part1/323_brick_pavement_80.jpg]

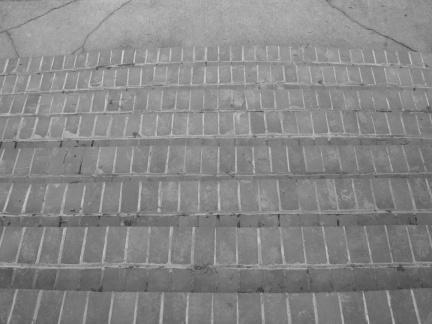

Supplement: Supplemental Information 3 [file peerj-cs-08-869-s003.zip › 1_part1/324_Pure Texture 171_157.jpg]

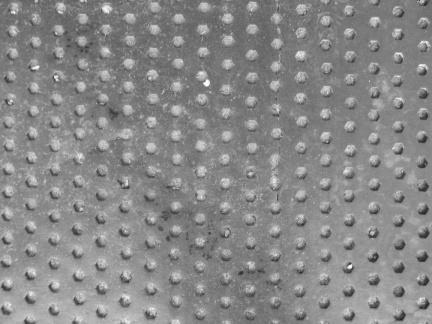

Supplement: Supplemental Information 3 [file peerj-cs-08-869-s003.zip › 1_part1/325_Pure Texture 171_139.jpg]

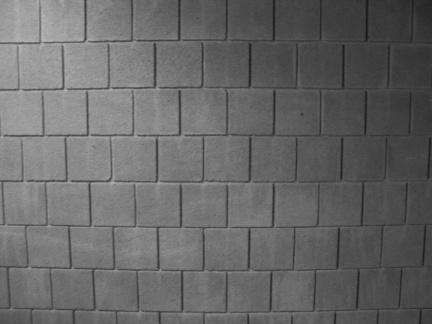

Supplement: Supplemental Information 3 [file peerj-cs-08-869-s003.zip › 1_part1/326_Pure Texture 171_142.jpg]

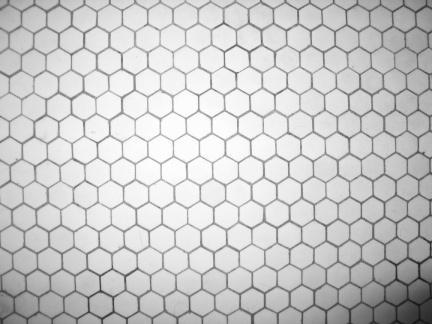

Supplement: Supplemental Information 3 [file peerj-cs-08-869-s003.zip › 1_part1/327_New Regular Textures 15_8.jpg]

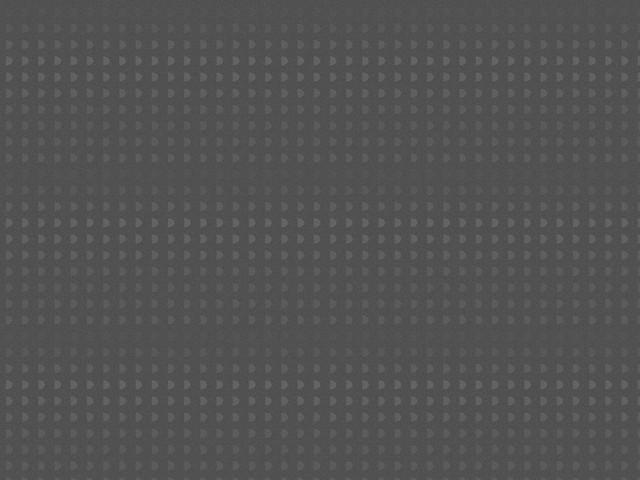

Supplement: Supplemental Information 3 [file peerj-cs-08-869-s003.zip › 1_part1/328_page13_10.jpg]

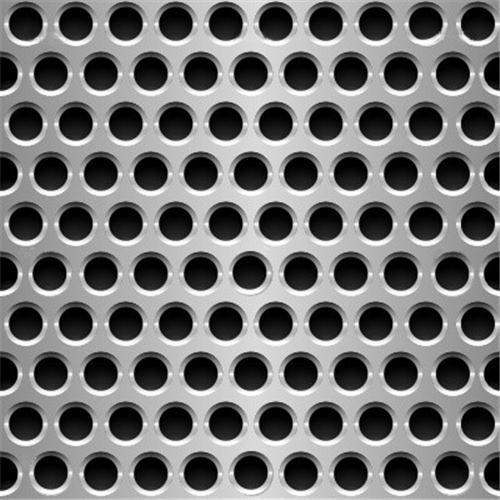

Supplement: Supplemental Information 3 [file peerj-cs-08-869-s003.zip › 1_part1/329_perforated_0012.jpg]

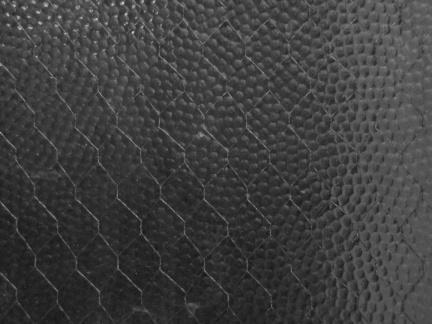

Supplement: Supplemental Information 3 [file peerj-cs-08-869-s003.zip › 1_part1/330_Pure Texture 171_130.jpg]

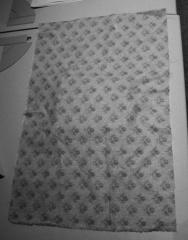

Supplement: Supplemental Information 3 [file peerj-cs-08-869-s003.zip › 1_part1/331_Normal nrt images 68_49.jpg]

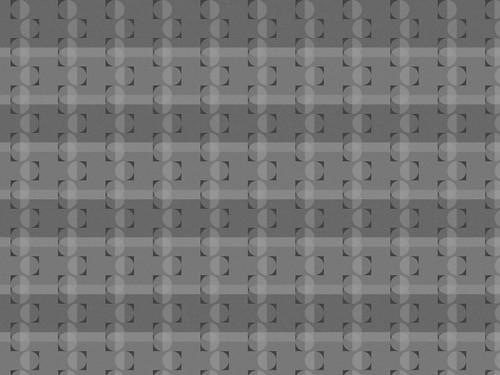

Supplement: Supplemental Information 3 [file peerj-cs-08-869-s003.zip › 1_part1/332_page13_6.jpg]

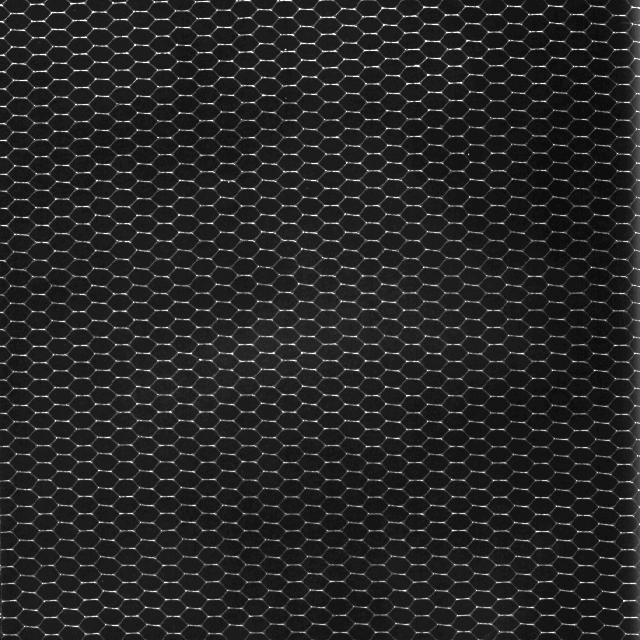

Supplement: Supplemental Information 3 [file peerj-cs-08-869-s003.zip › 1_part1/333_D34.jpg]

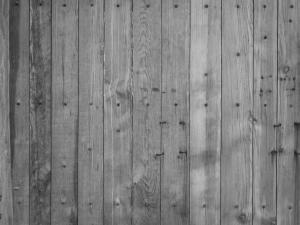

Supplement: Supplemental Information 3 [file peerj-cs-08-869-s003.zip › 1_part1/334_Planks new_41.jpg]

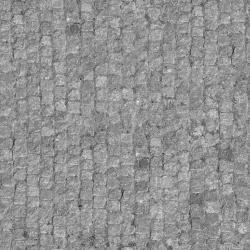

Supplement: Supplemental Information 3 [file peerj-cs-08-869-s003.zip › 1_part1/335_brick_pavement_41.jpg]

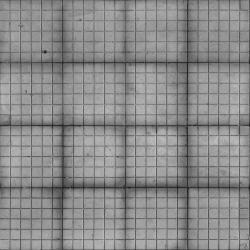

Supplement: Supplemental Information 3 [file peerj-cs-08-869-s003.zip › 1_part1/336_brick_pavement_0046_03_thumb.jpg]

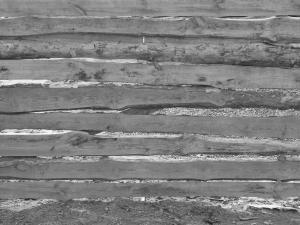

Supplement: Supplemental Information 3 [file peerj-cs-08-869-s003.zip › 1_part1/337_Planks new_38.jpg]

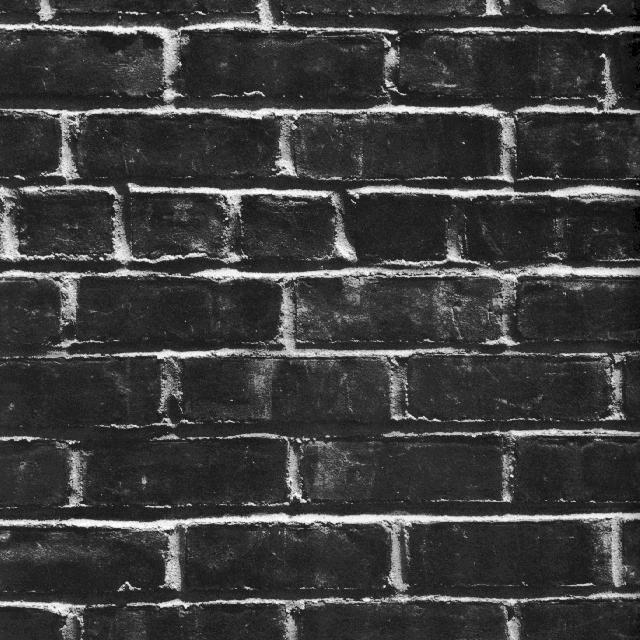

Supplement: Supplemental Information 3 [file peerj-cs-08-869-s003.zip › 1_part1/338_D96.jpg]

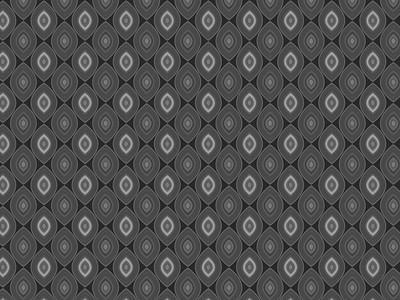

Supplement: Supplemental Information 3 [file peerj-cs-08-869-s003.zip › 1_part1/339_48501815981_c8605bf6bd_w.jpg]

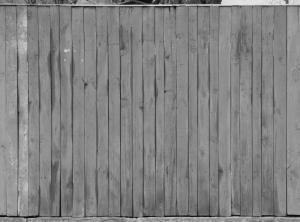

Supplement: Supplemental Information 3 [file peerj-cs-08-869-s003.zip › 1_part1/340_Planks old_27.jpg]

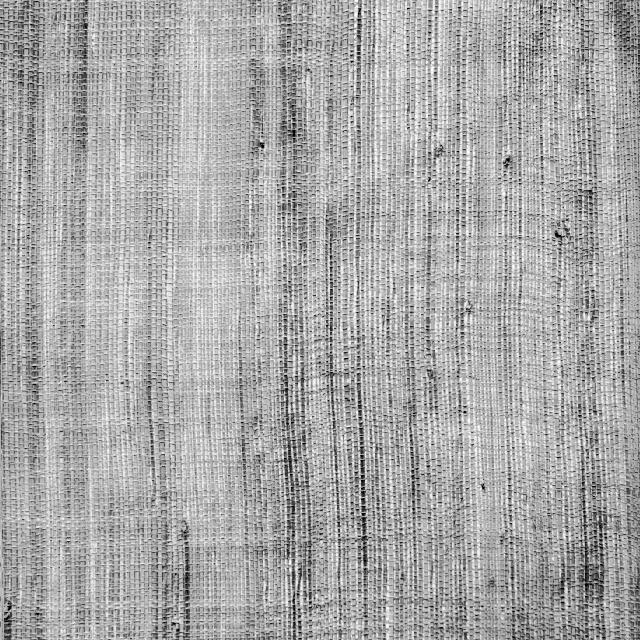

Supplement: Supplemental Information 3 [file peerj-cs-08-869-s003.zip › 1_part1/341_D78.jpg]

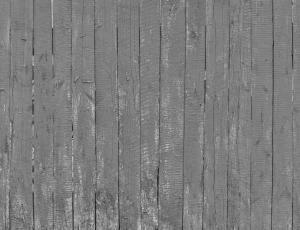

Supplement: Supplemental Information 3 [file peerj-cs-08-869-s003.zip › 1_part1/342_Planks old_97.jpg]

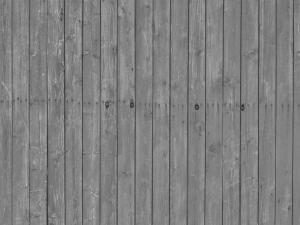

Supplement: Supplemental Information 3 [file peerj-cs-08-869-s003.zip › 1_part1/343_Planks old_50.jpg]

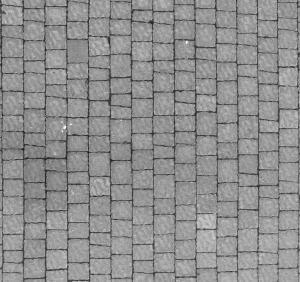

Supplement: Supplemental Information 3 [file peerj-cs-08-869-s003.zip › 1_part1/344_brick_pavement_0005_01_thumb.jpg]

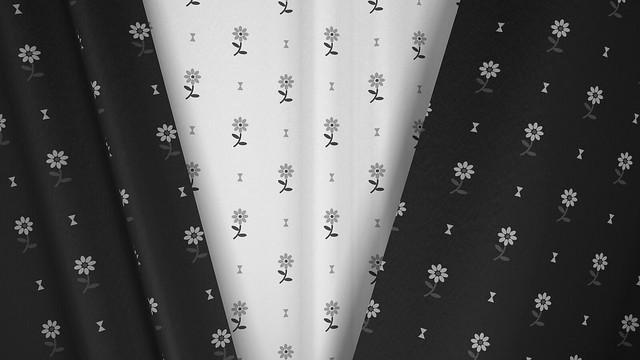

Supplement: Supplemental Information 3 [file peerj-cs-08-869-s003.zip › 1_part1/345_page10_5.jpg]

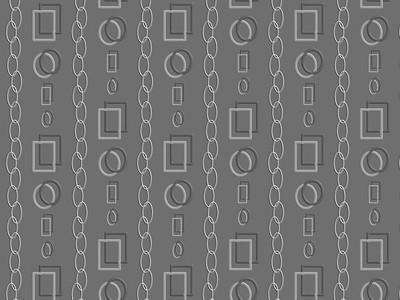

Supplement: Supplemental Information 3 [file peerj-cs-08-869-s003.zip › 1_part1/346_page5_0.jpg]

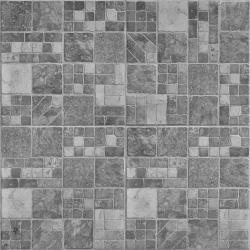

Supplement: Supplemental Information 3 [file peerj-cs-08-869-s003.zip › 1_part1/347_brick_pavement_72.jpg]

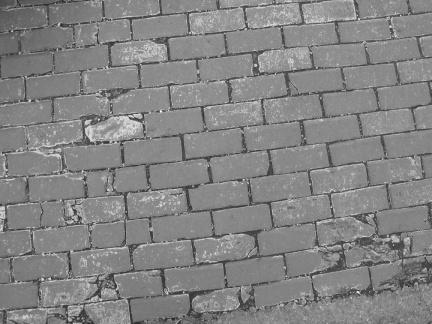

Supplement: Supplemental Information 3 [file peerj-cs-08-869-s003.zip › 1_part1/348_Pure Texture 171_163.jpg]

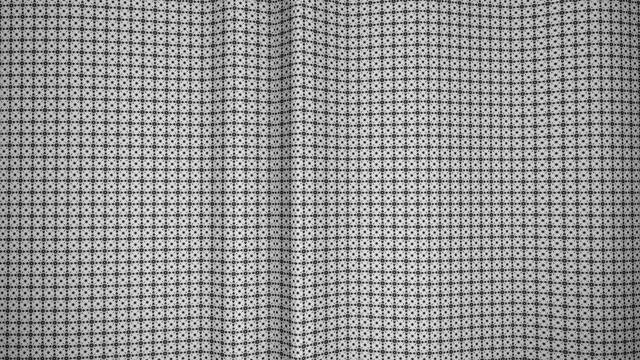

Supplement: Supplemental Information 3 [file peerj-cs-08-869-s003.zip › 1_part1/349_page10_22.jpg]

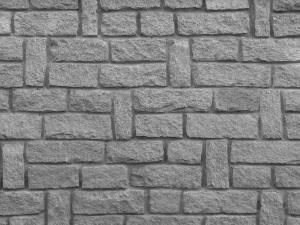

Supplement: Supplemental Information 3 [file peerj-cs-08-869-s003.zip › 1_part1/350_stone_wall_89.jpg]

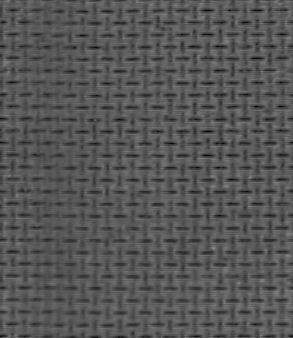

Supplement: Supplemental Information 3 [file peerj-cs-08-869-s003.zip › 1_part1/351_Masont_t.jpg]

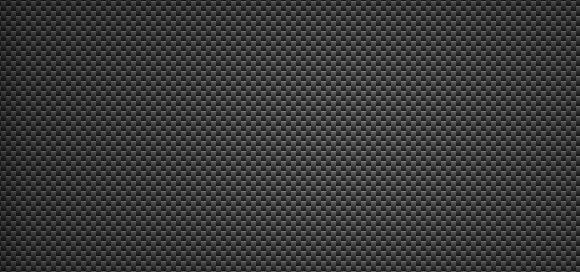

Supplement: Supplemental Information 3 [file peerj-cs-08-869-s003.zip › 1_part1/352_09_free_subtle_textures_carbon_fibre.jpg]

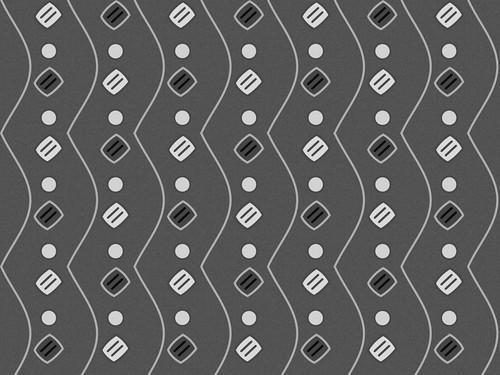

Supplement: Supplemental Information 3 [file peerj-cs-08-869-s003.zip › 1_part1/353_page12_20.jpg]

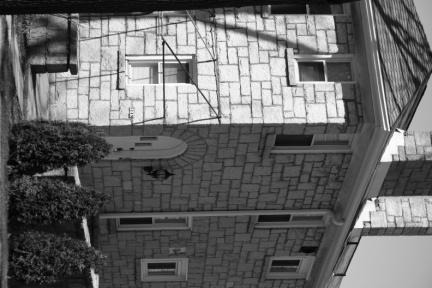

Supplement: Supplemental Information 3 [file peerj-cs-08-869-s003.zip › 1_part1/354_Borderline Near-Regular Textures 65_35.jpg]

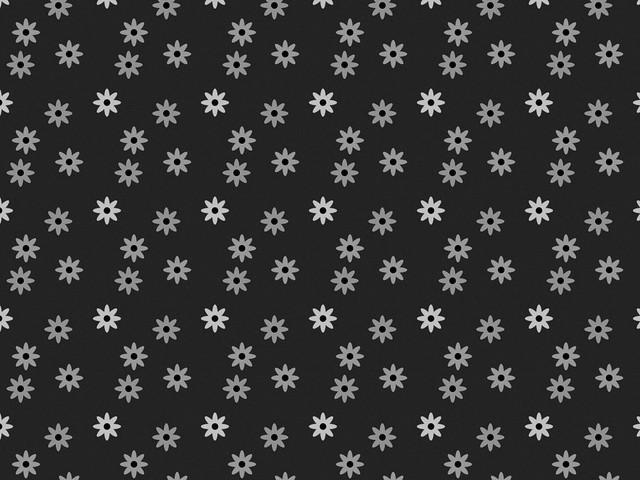

Supplement: Supplemental Information 3 [file peerj-cs-08-869-s003.zip › 1_part1/355_page13_3.jpg]

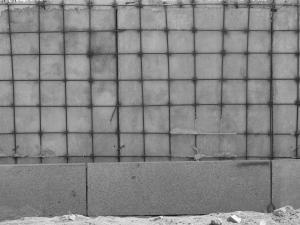

Supplement: Supplemental Information 3 [file peerj-cs-08-869-s003.zip › 1_part1/356_concrete other_22.jpg]

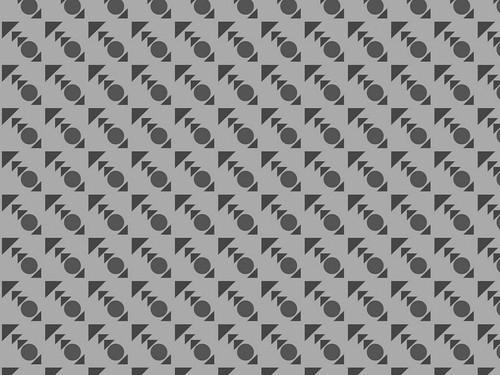

Supplement: Supplemental Information 3 [file peerj-cs-08-869-s003.zip › 1_part1/357_page7_8.jpg]

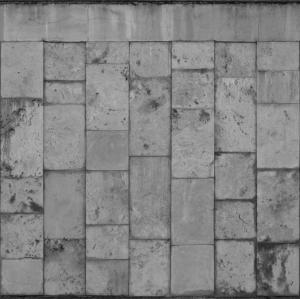

Supplement: Supplemental Information 3 [file peerj-cs-08-869-s003.zip › 1_part1/358_tile_tile_21.jpg]

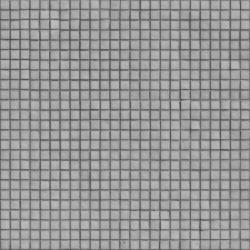

Supplement: Supplemental Information 3 [file peerj-cs-08-869-s003.zip › 1_part1/359_tile_tile_31.jpg]

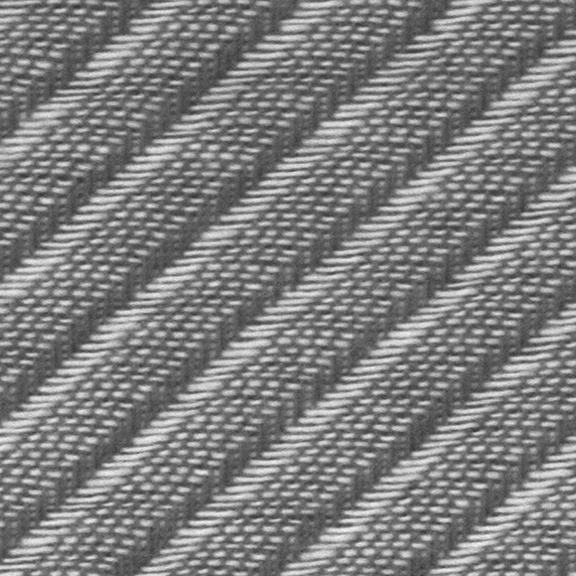

Supplement: Supplemental Information 3 [file peerj-cs-08-869-s003.zip › 1_part1/360_screen1-a-p011.jpg]

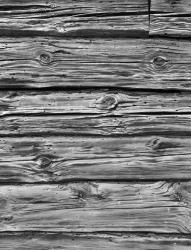

Supplement: Supplemental Information 3 [file peerj-cs-08-869-s003.zip › 1_part1/361_Planks old_23.jpg]

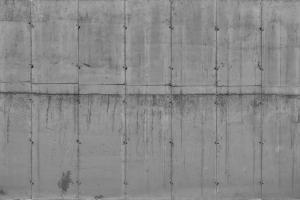

Supplement: Supplemental Information 3 [file peerj-cs-08-869-s003.zip › 1_part1/362_concrete massive_1.jpg]

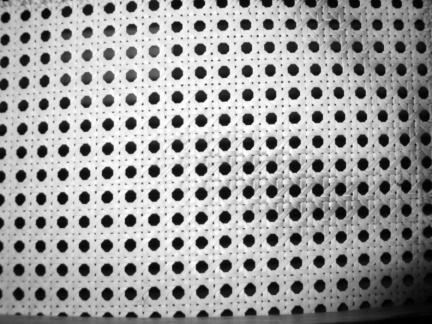

Supplement: Supplemental Information 3 [file peerj-cs-08-869-s003.zip › 1_part1/363_Pure Texture 171_104.jpg]

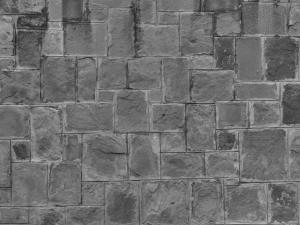

Supplement: Supplemental Information 3 [file peerj-cs-08-869-s003.zip › 1_part1/364_stone_wall_67.jpg]

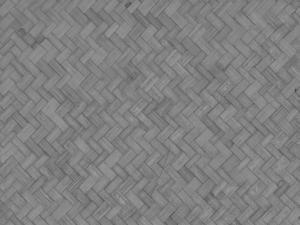

Supplement: Supplemental Information 3 [file peerj-cs-08-869-s003.zip › 1_part1/365_wood_constructions_0072_01_thumb.jpg]

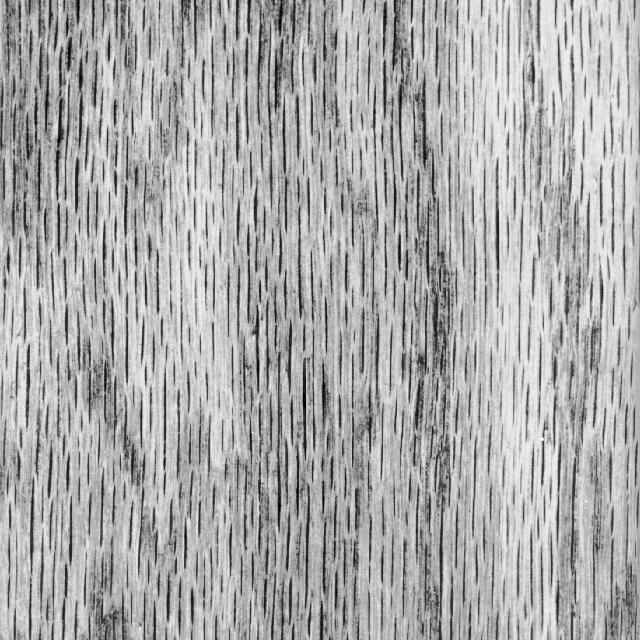

Supplement: Supplemental Information 3 [file peerj-cs-08-869-s003.zip › 1_part1/366_D68.jpg]

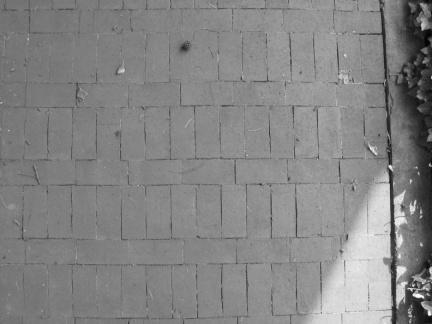

Supplement: Supplemental Information 3 [file peerj-cs-08-869-s003.zip › 1_part1/367_Pure Texture 171_109.jpg]

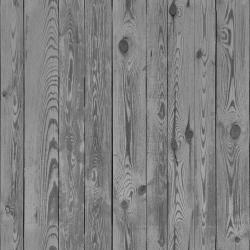

Supplement: Supplemental Information 3 [file peerj-cs-08-869-s003.zip › 1_part1/368_Planks old_51.jpg]

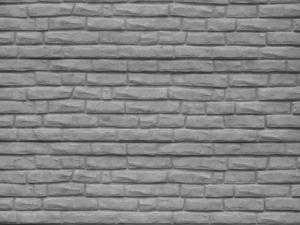

Supplement: Supplemental Information 3 [file peerj-cs-08-869-s003.zip › 1_part1/369_stone_wall_52.jpg]

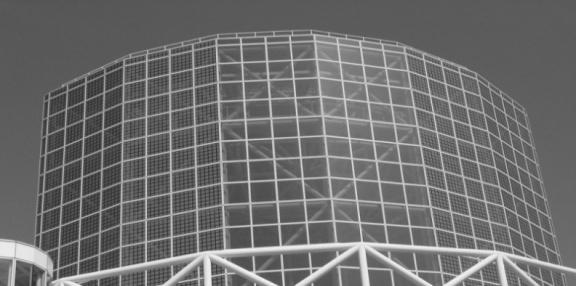

Supplement: Supplemental Information 3 [file peerj-cs-08-869-s003.zip › 1_part1/370_Normal nrt images 68_37.jpg]

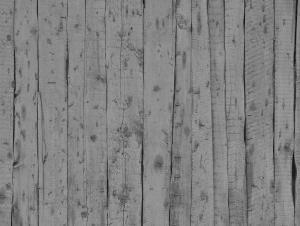

Supplement: Supplemental Information 3 [file peerj-cs-08-869-s003.zip › 1_part1/371_Planks old_83.jpg]

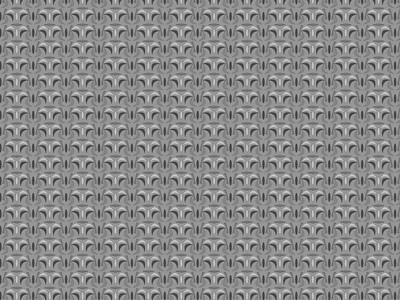

Supplement: Supplemental Information 3 [file peerj-cs-08-869-s003.zip › 1_part1/372_40511826303_c18a379ab6_w.jpg]

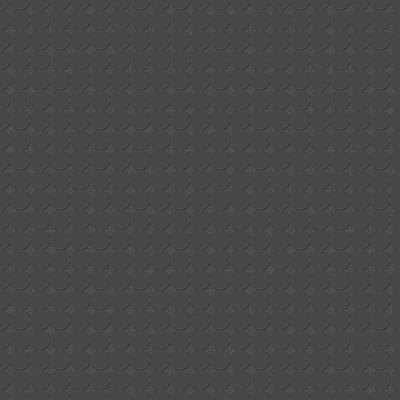

Supplement: Supplemental Information 3 [file peerj-cs-08-869-s003.zip › 1_part1/373_page13_18.jpg]

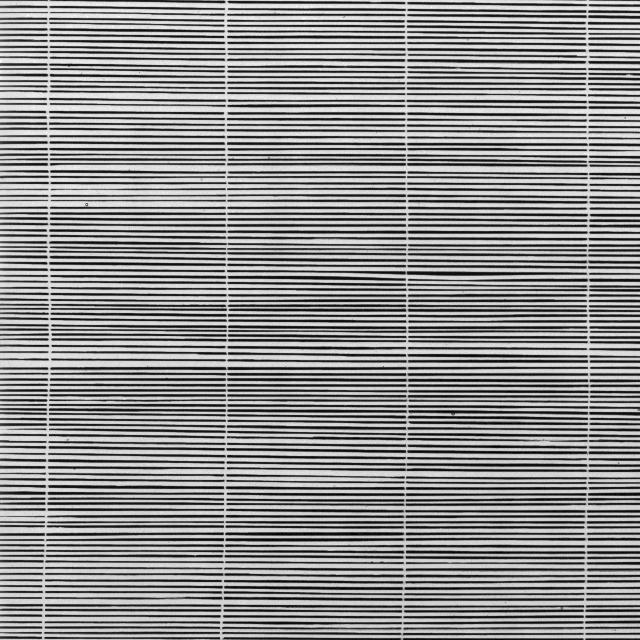

Supplement: Supplemental Information 3 [file peerj-cs-08-869-s003.zip › 1_part1/374_D49.jpg]

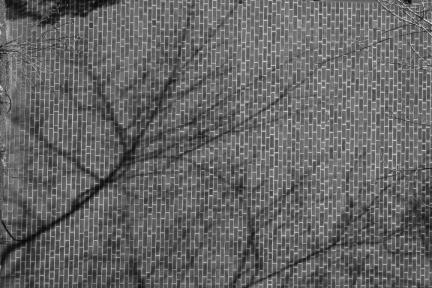

Supplement: Supplemental Information 3 [file peerj-cs-08-869-s003.zip › 1_part1/375_Pure Texture 171_150.jpg]

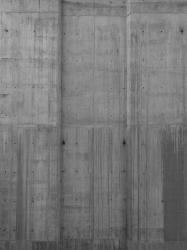

Supplement: Supplemental Information 3 [file peerj-cs-08-869-s003.zip › 1_part1/376_concrete massive_75.jpg]

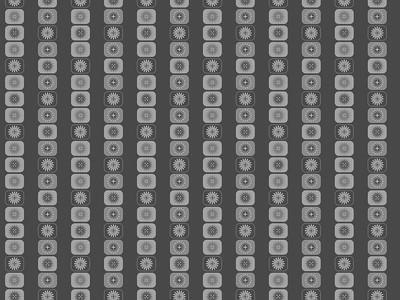

Supplement: Supplemental Information 3 [file peerj-cs-08-869-s003.zip › 1_part1/377_48278786841_ff73d629ec_w.jpg]

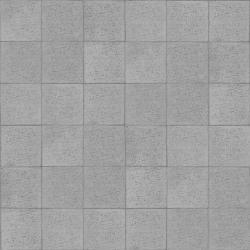

Supplement: Supplemental Information 3 [file peerj-cs-08-869-s003.zip › 1_part1/378_brick_pavement_46.jpg]

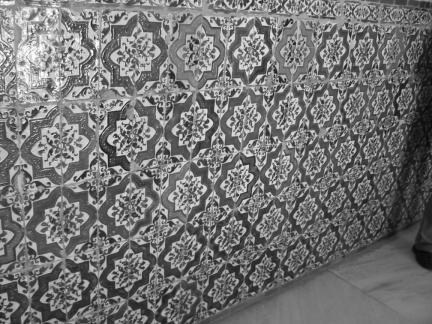

Supplement: Supplemental Information 3 [file peerj-cs-08-869-s003.zip › 1_part1/379_Pure Texture 171_42.jpg]

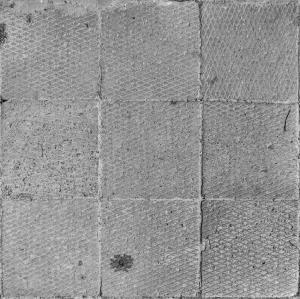

Supplement: Supplemental Information 4 [file peerj-cs-08-869-s004.zip › 1_part2/100_brick_pavement_126.jpg]

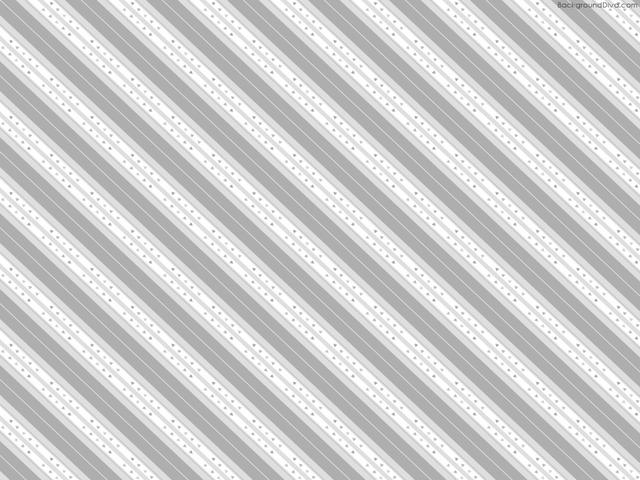

Supplement: Supplemental Information 4 [file peerj-cs-08-869-s004.zip › 1_part2/100_nded_0008.jpg]
