# Supplementary figures and images for: Deep convolutional neural networks for regular texture recognition (part 5 of 8)
Source: PeerJ Comput Sci. 2022 Feb 9;8:e869. doi: 10.7717/peerj-cs.869 (PMC9044313; doi:10.7717/peerj-cs.869)

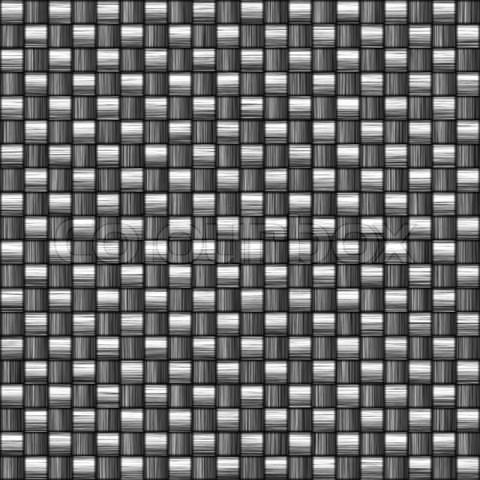

Supplement: Supplemental Information 3 [file peerj-cs-08-869-s003.zip › 1_part1/246_ven_0099.jpg]

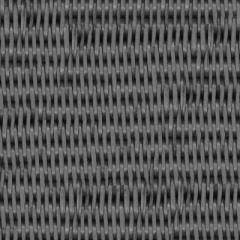

Supplement: Supplemental Information 3 [file peerj-cs-08-869-s003.zip › 1_part1/247_Graph cut texture synthesis results 92_2.jpg]

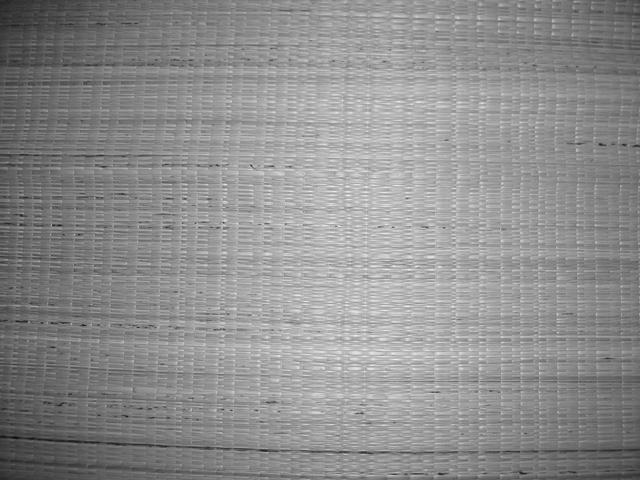

Supplement: Supplemental Information 3 [file peerj-cs-08-869-s003.zip › 1_part1/247_ven_0016.jpg]

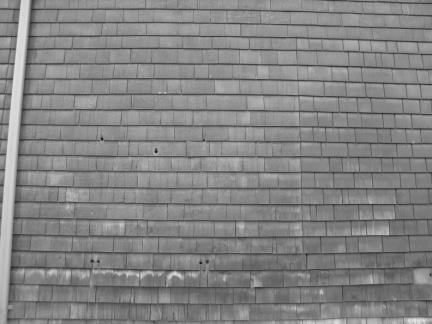

Supplement: Supplemental Information 3 [file peerj-cs-08-869-s003.zip › 1_part1/248_Borderline Near-Regular Textures 65_53.jpg]

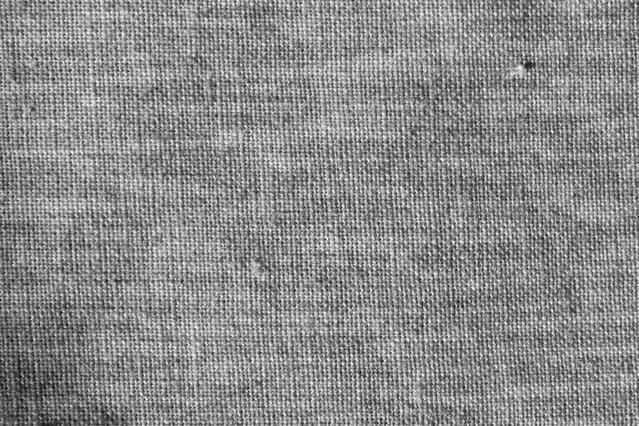

Supplement: Supplemental Information 3 [file peerj-cs-08-869-s003.zip › 1_part1/248_ven_0013.jpg]

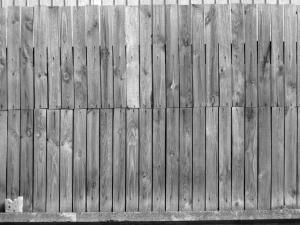

Supplement: Supplemental Information 3 [file peerj-cs-08-869-s003.zip › 1_part1/249_Planks old_100.jpg]

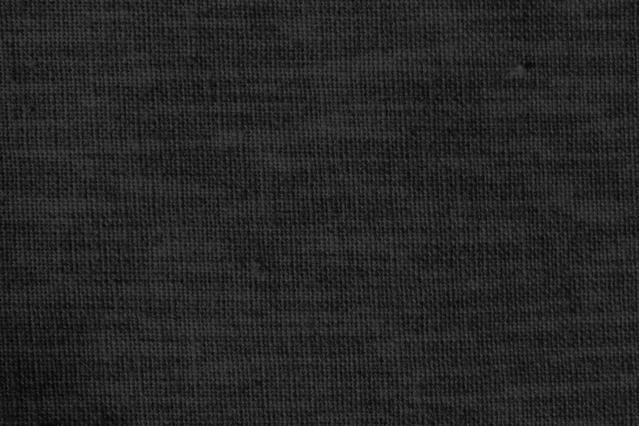

Supplement: Supplemental Information 3 [file peerj-cs-08-869-s003.zip › 1_part1/249_ven_0014.jpg]

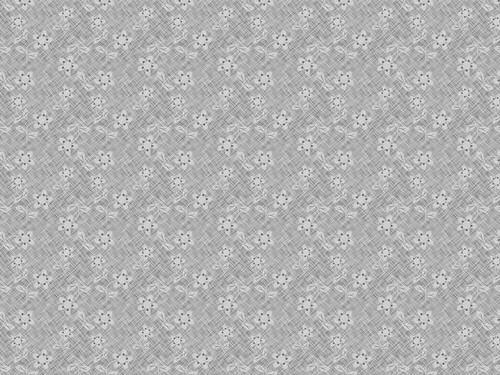

Supplement: Supplemental Information 3 [file peerj-cs-08-869-s003.zip › 1_part1/250_page6_20.jpg]

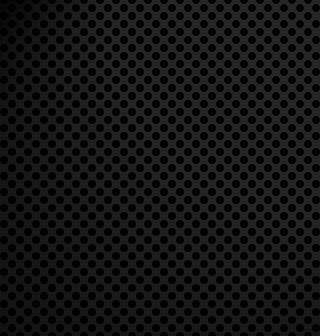

Supplement: Supplemental Information 3 [file peerj-cs-08-869-s003.zip › 1_part1/250_rforated_0046.jpg]

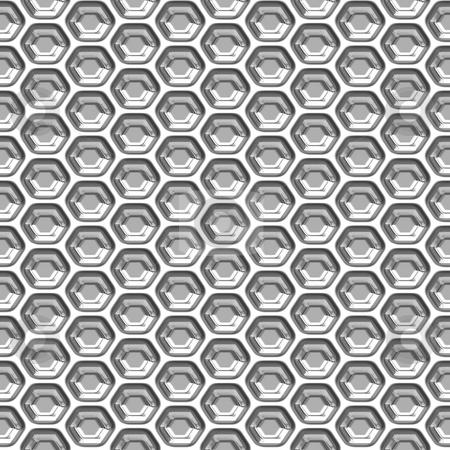

Supplement: Supplemental Information 3 [file peerj-cs-08-869-s003.zip › 1_part1/251_neycombed_0098.jpg]

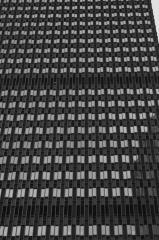

Supplement: Supplemental Information 3 [file peerj-cs-08-869-s003.zip › 1_part1/251_Pure Texture 171_56.jpg]

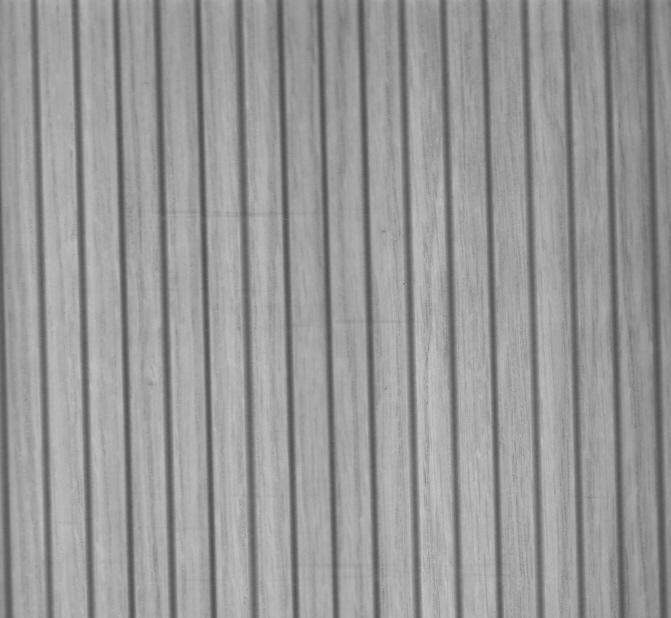

Supplement: Supplemental Information 3 [file peerj-cs-08-869-s003.zip › 1_part1/252_ooved_0164.jpg]

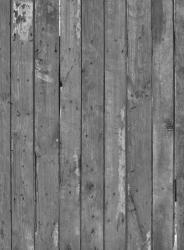

Supplement: Supplemental Information 3 [file peerj-cs-08-869-s003.zip › 1_part1/252_Planks old_98.jpg]

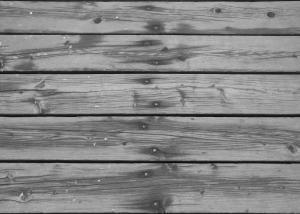

Supplement: Supplemental Information 3 [file peerj-cs-08-869-s003.zip › 1_part1/253_Planks old_9.jpg]

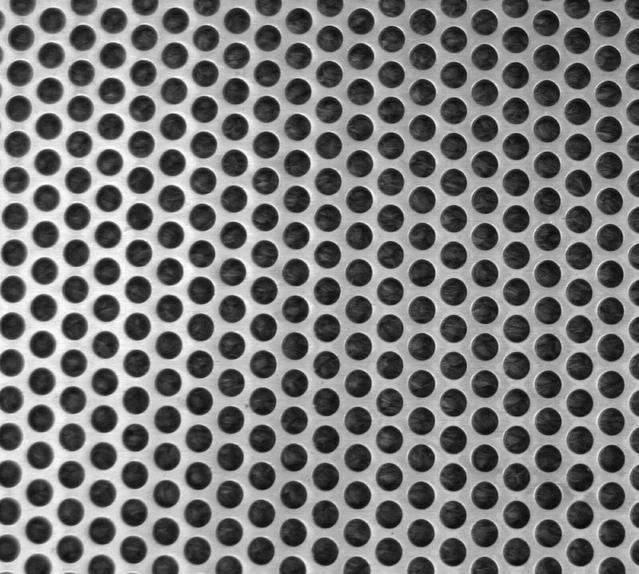

Supplement: Supplemental Information 3 [file peerj-cs-08-869-s003.zip › 1_part1/253_rforated_0068.jpg]

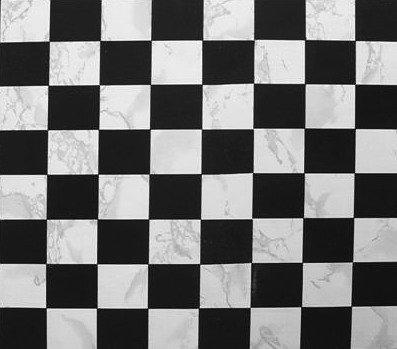

Supplement: Supplemental Information 3 [file peerj-cs-08-869-s003.zip › 1_part1/254_equered_0123.jpg]

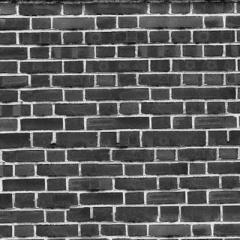

Supplement: Supplemental Information 3 [file peerj-cs-08-869-s003.zip › 1_part1/254_Graph cut texture synthesis results 92_44.jpg]

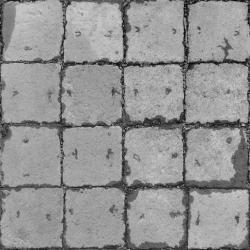

Supplement: Supplemental Information 3 [file peerj-cs-08-869-s003.zip › 1_part1/255_brick_pavement_78.jpg]

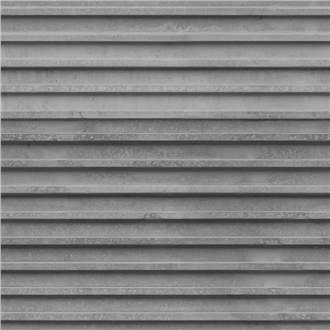

Supplement: Supplemental Information 3 [file peerj-cs-08-869-s003.zip › 1_part1/255_ooved_0048.jpg]

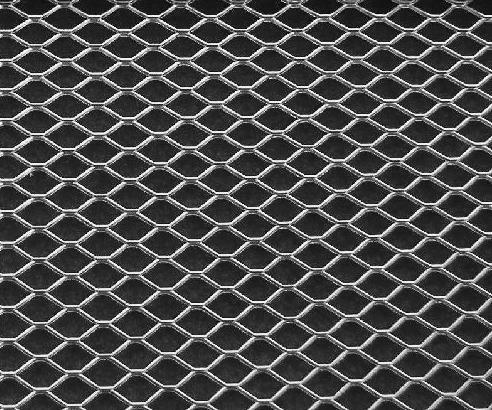

Supplement: Supplemental Information 3 [file peerj-cs-08-869-s003.zip › 1_part1/256_shed_0161.jpg]

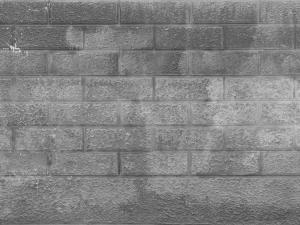

Supplement: Supplemental Information 3 [file peerj-cs-08-869-s003.zip › 1_part1/256_stone_wall_15.jpg]

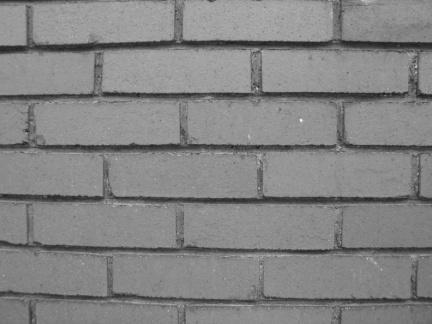

Supplement: Supplemental Information 3 [file peerj-cs-08-869-s003.zip › 1_part1/257_Pure Texture 171_99.jpg]

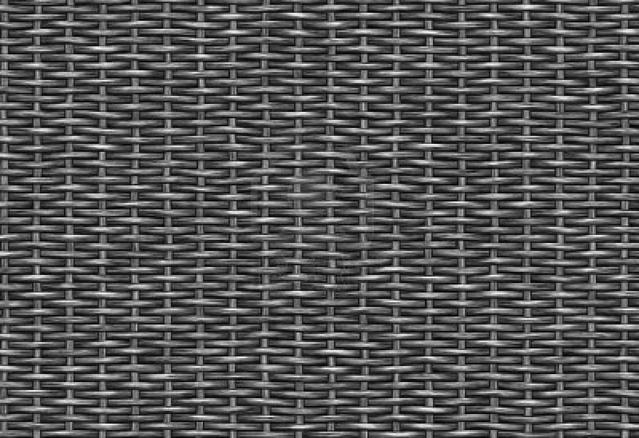

Supplement: Supplemental Information 3 [file peerj-cs-08-869-s003.zip › 1_part1/257_ven_0081.jpg]

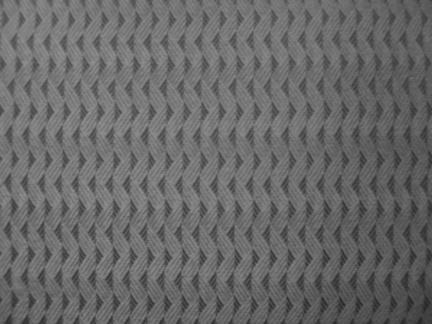

Supplement: Supplemental Information 3 [file peerj-cs-08-869-s003.zip › 1_part1/258_New Regular Textures 15_2.jpg]

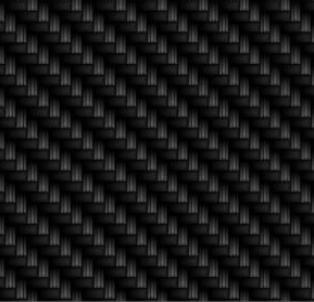

Supplement: Supplemental Information 3 [file peerj-cs-08-869-s003.zip › 1_part1/258_ven_0115.jpg]

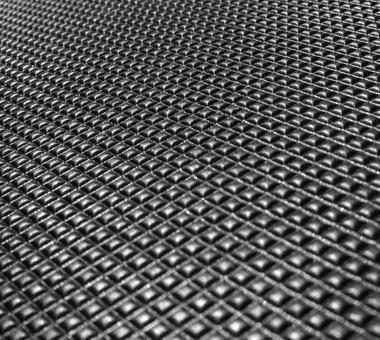

Supplement: Supplemental Information 3 [file peerj-cs-08-869-s003.zip › 1_part1/259_mpy_0072.jpg]

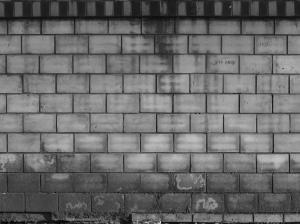

Supplement: Supplemental Information 3 [file peerj-cs-08-869-s003.zip › 1_part1/259_tile_tile_50.jpg]

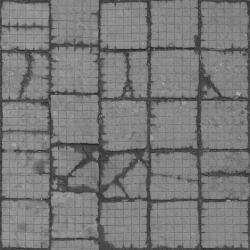

Supplement: Supplemental Information 3 [file peerj-cs-08-869-s003.zip › 1_part1/260_brick_pavement_85.jpg]

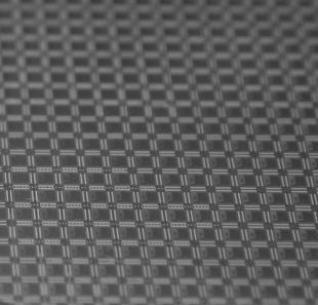

Supplement: Supplemental Information 3 [file peerj-cs-08-869-s003.zip › 1_part1/260_ffled_0156.jpg]

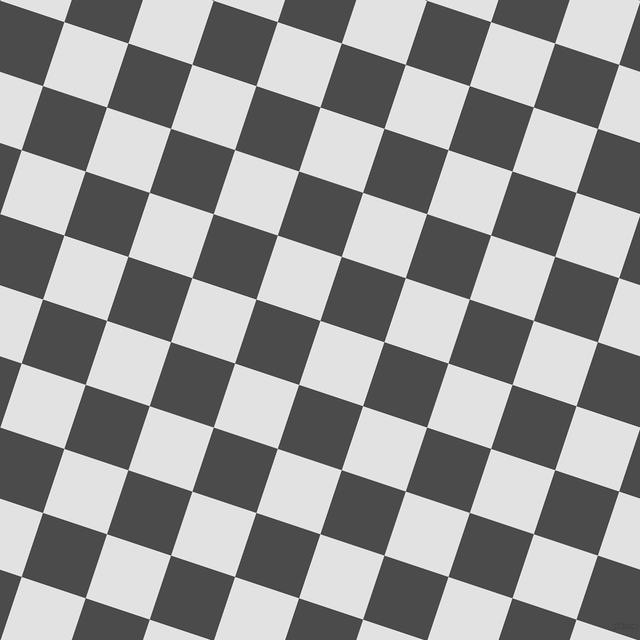

Supplement: Supplemental Information 3 [file peerj-cs-08-869-s003.zip › 1_part1/261_equered_0046.jpg]

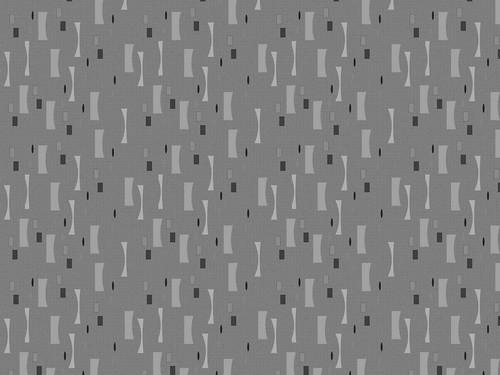

Supplement: Supplemental Information 3 [file peerj-cs-08-869-s003.zip › 1_part1/261_page12_10.jpg]

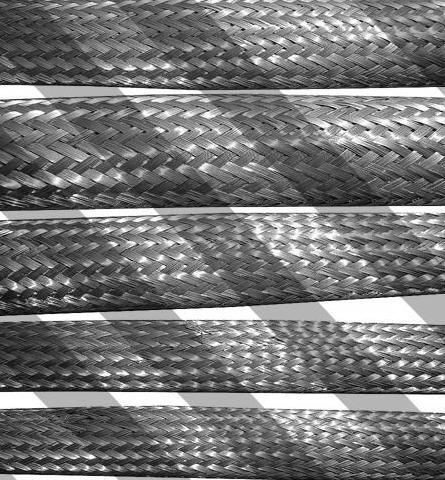

Supplement: Supplemental Information 3 [file peerj-cs-08-869-s003.zip › 1_part1/262_aided_0188.jpg]

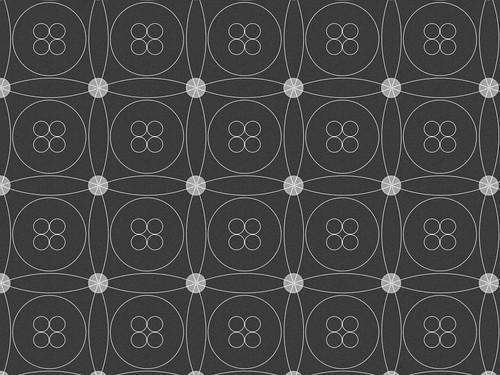

Supplement: Supplemental Information 3 [file peerj-cs-08-869-s003.zip › 1_part1/262_page12_14.jpg]

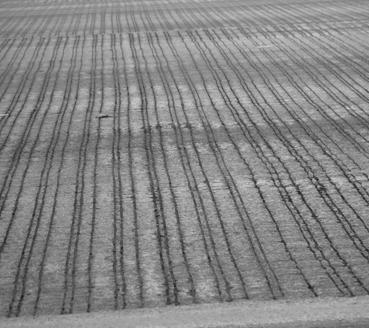

Supplement: Supplemental Information 3 [file peerj-cs-08-869-s003.zip › 1_part1/263_ooved_0118.jpg]

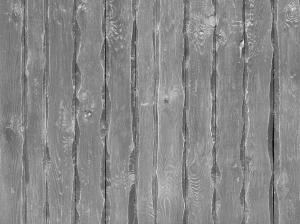

Supplement: Supplemental Information 3 [file peerj-cs-08-869-s003.zip › 1_part1/263_Planks new_22.jpg]

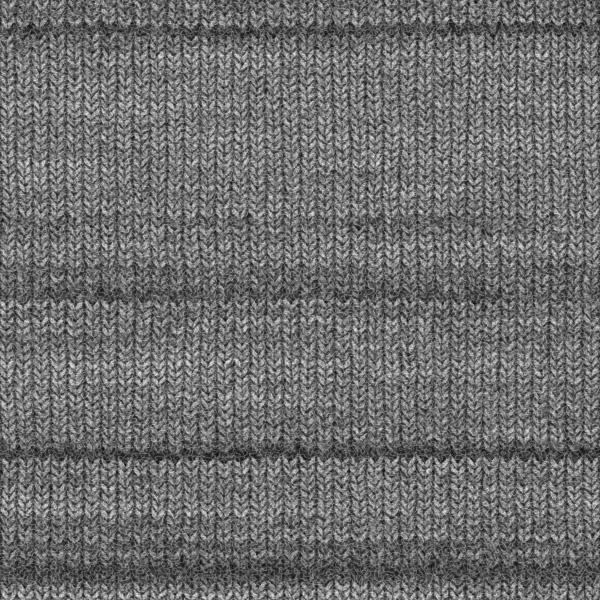

Supplement: Supplemental Information 3 [file peerj-cs-08-869-s003.zip › 1_part1/264_itted_0102.jpg]

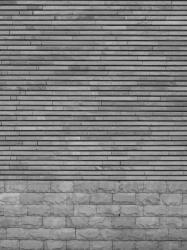

Supplement: Supplemental Information 3 [file peerj-cs-08-869-s003.zip › 1_part1/264_stone_wall_104.jpg]

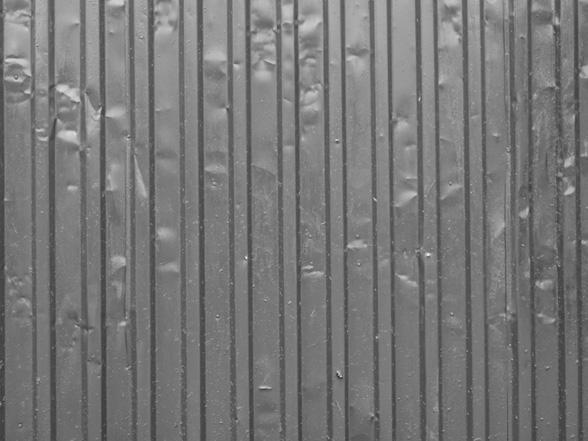

Supplement: Supplemental Information 3 [file peerj-cs-08-869-s003.zip › 1_part1/265_ooved_0083.jpg]

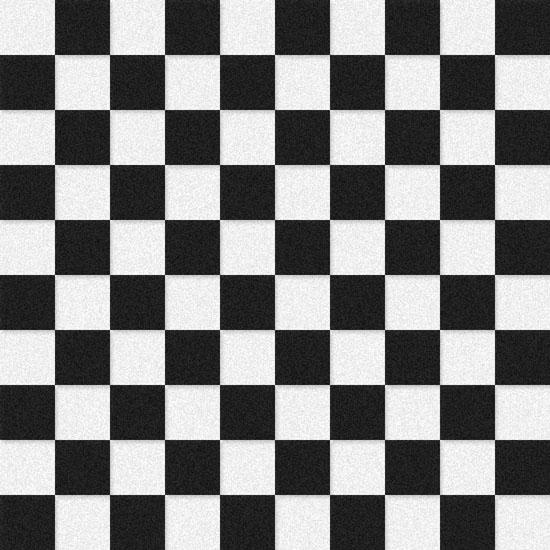

Supplement: Supplemental Information 3 [file peerj-cs-08-869-s003.zip › 1_part1/266_equered_0098.jpg]

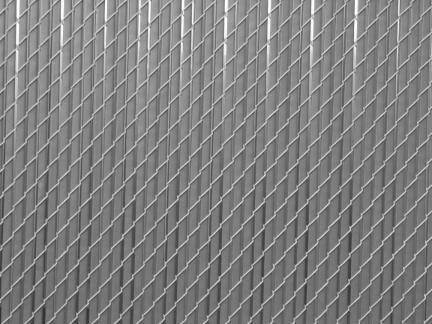

Supplement: Supplemental Information 3 [file peerj-cs-08-869-s003.zip › 1_part1/266_Pure Texture 171_4.jpg]

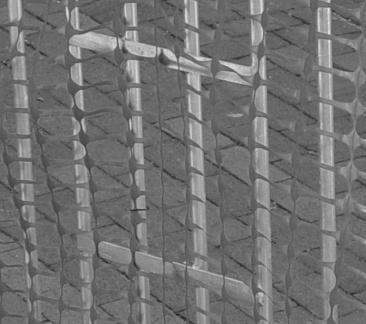

Supplement: Supplemental Information 3 [file peerj-cs-08-869-s003.zip › 1_part1/267_Normal nrt images 68_34.jpg]

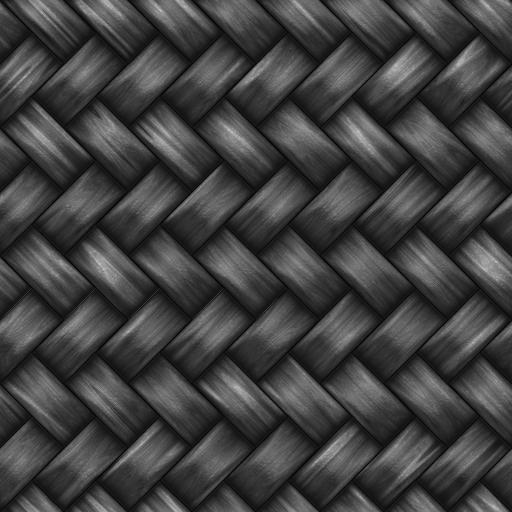

Supplement: Supplemental Information 3 [file peerj-cs-08-869-s003.zip › 1_part1/267_ven_0093.jpg]

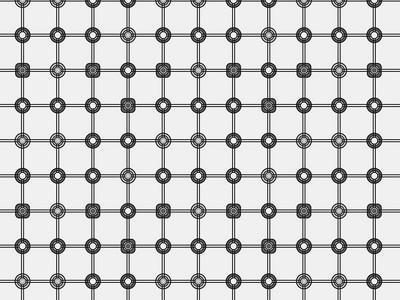

Supplement: Supplemental Information 3 [file peerj-cs-08-869-s003.zip › 1_part1/268_47835836552_e47491464c_w (1).jpg]

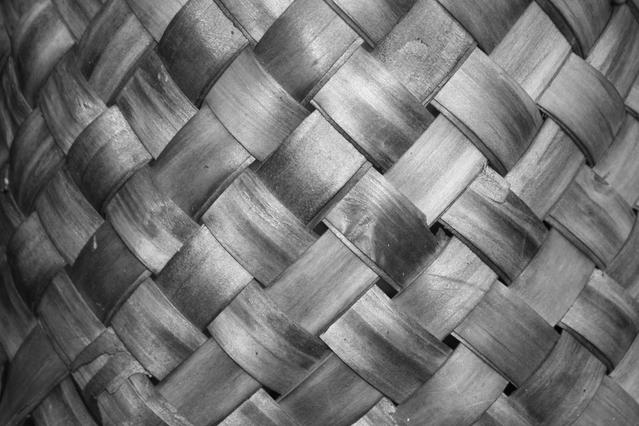

Supplement: Supplemental Information 3 [file peerj-cs-08-869-s003.zip › 1_part1/268_ven_0047.jpg]

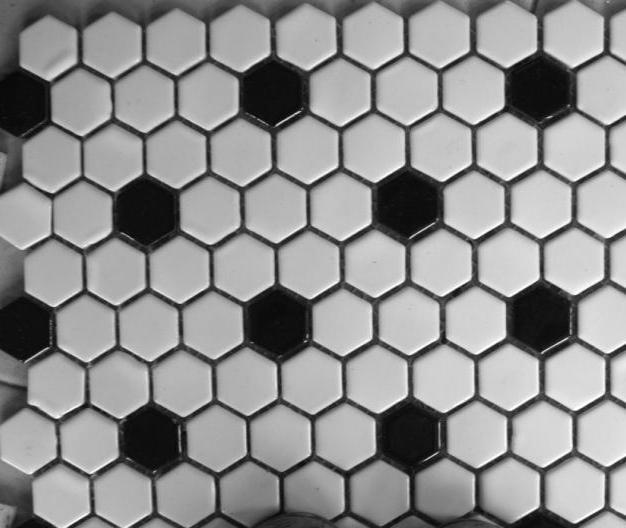

Supplement: Supplemental Information 3 [file peerj-cs-08-869-s003.zip › 1_part1/269_neycombed_0129.jpg]

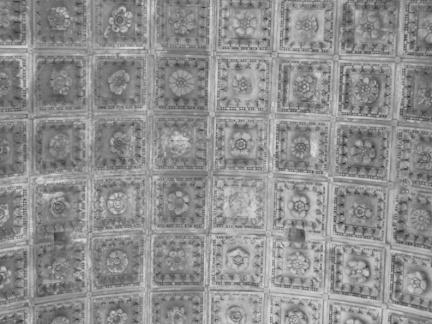

Supplement: Supplemental Information 3 [file peerj-cs-08-869-s003.zip › 1_part1/269_Normal nrt images 68_57.jpg]

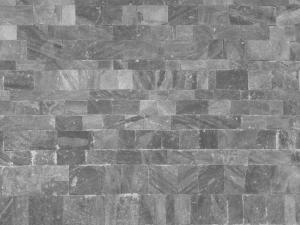

Supplement: Supplemental Information 3 [file peerj-cs-08-869-s003.zip › 1_part1/270_brick_pavement_107.jpg]

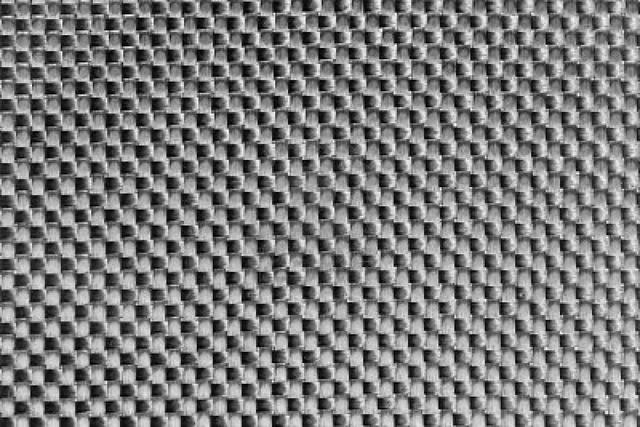

Supplement: Supplemental Information 3 [file peerj-cs-08-869-s003.zip › 1_part1/270_ven_0095.jpg]

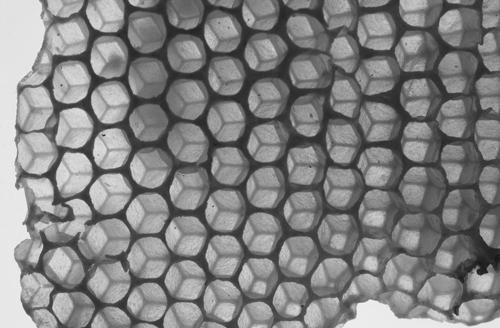

Supplement: Supplemental Information 3 [file peerj-cs-08-869-s003.zip › 1_part1/271_neycombed_0051.jpg]

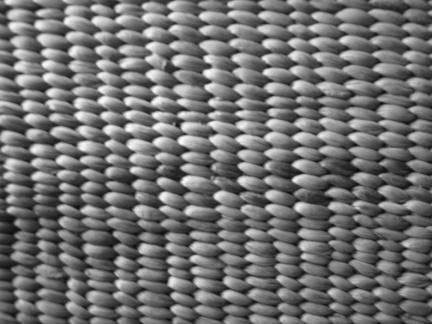

Supplement: Supplemental Information 3 [file peerj-cs-08-869-s003.zip › 1_part1/271_Pure Texture 171_95.jpg]

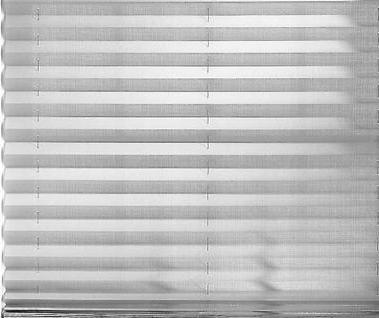

Supplement: Supplemental Information 3 [file peerj-cs-08-869-s003.zip › 1_part1/272_eated_0160.jpg]

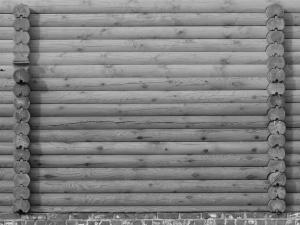

Supplement: Supplemental Information 3 [file peerj-cs-08-869-s003.zip › 1_part1/272_Planks new_23.jpg]

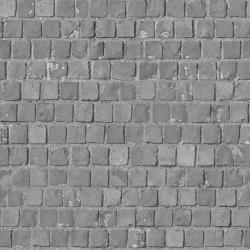

Supplement: Supplemental Information 3 [file peerj-cs-08-869-s003.zip › 1_part1/273_brick_pavement_111.jpg]

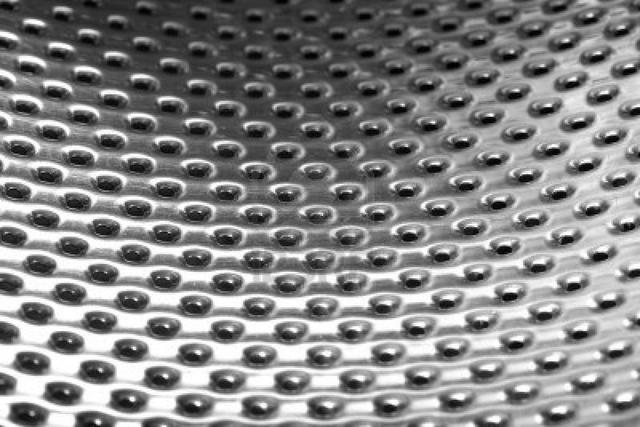

Supplement: Supplemental Information 3 [file peerj-cs-08-869-s003.zip › 1_part1/273_rforated_0077.jpg]

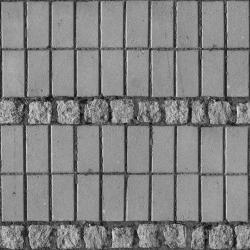

Supplement: Supplemental Information 3 [file peerj-cs-08-869-s003.zip › 1_part1/274_brick_pavement_64.jpg]

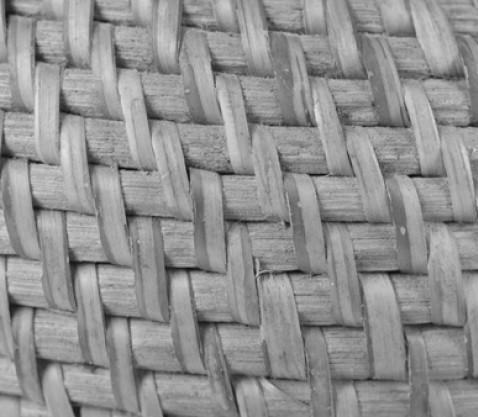

Supplement: Supplemental Information 3 [file peerj-cs-08-869-s003.zip › 1_part1/274_ven_0109.jpg]

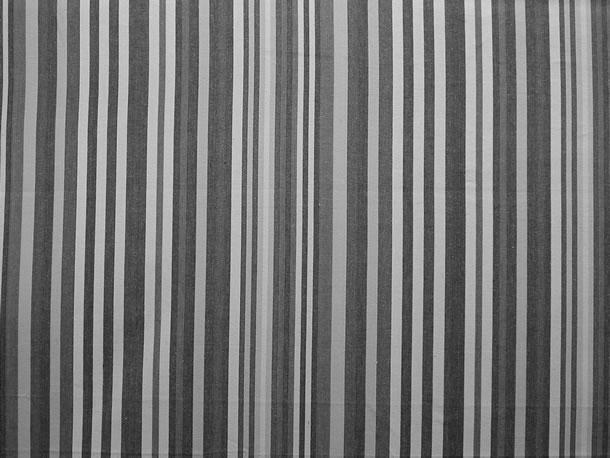

Supplement: Supplemental Information 3 [file peerj-cs-08-869-s003.zip › 1_part1/275_nded_0019.jpg]

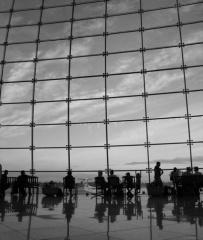

Supplement: Supplemental Information 3 [file peerj-cs-08-869-s003.zip › 1_part1/275_Normal nrt images 68_67.jpg]

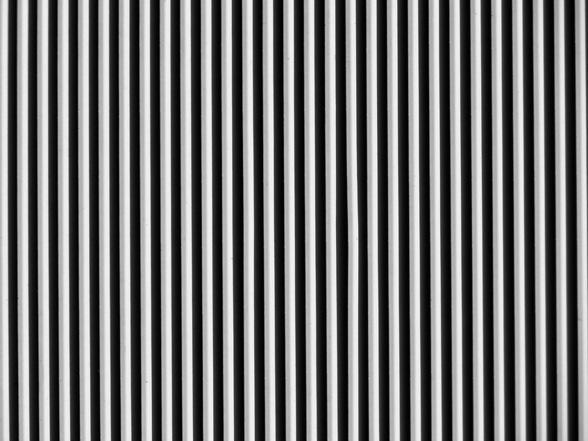

Supplement: Supplemental Information 3 [file peerj-cs-08-869-s003.zip › 1_part1/276_ooved_0085.jpg]

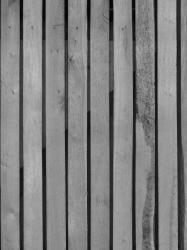

Supplement: Supplemental Information 3 [file peerj-cs-08-869-s003.zip › 1_part1/276_Planks new_21.jpg]

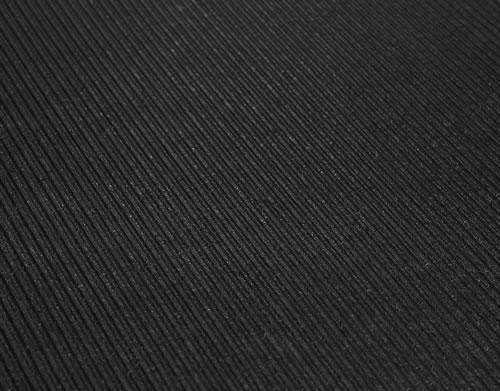

Supplement: Supplemental Information 3 [file peerj-cs-08-869-s003.zip › 1_part1/277_ooved_0126.jpg]

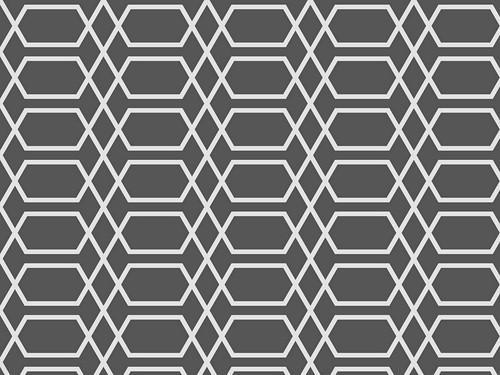

Supplement: Supplemental Information 3 [file peerj-cs-08-869-s003.zip › 1_part1/277_page8_16.jpg]

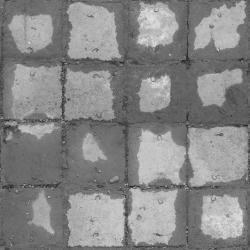

Supplement: Supplemental Information 3 [file peerj-cs-08-869-s003.zip › 1_part1/278_brick_pavement_74.jpg]

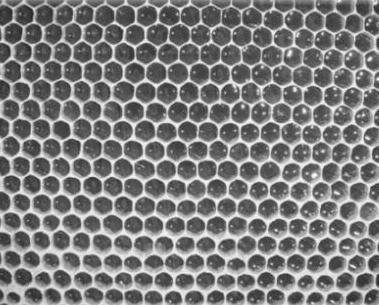

Supplement: Supplemental Information 3 [file peerj-cs-08-869-s003.zip › 1_part1/278_neycombed_0161.jpg]

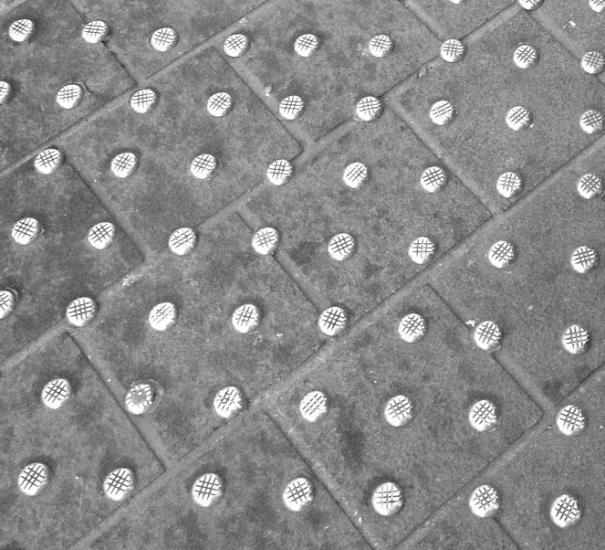

Supplement: Supplemental Information 3 [file peerj-cs-08-869-s003.zip › 1_part1/279_mpy_0101.jpg]

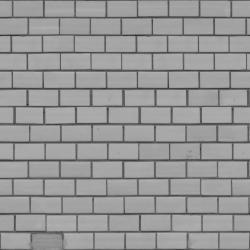

Supplement: Supplemental Information 3 [file peerj-cs-08-869-s003.zip › 1_part1/279_tile_tile_46.jpg]

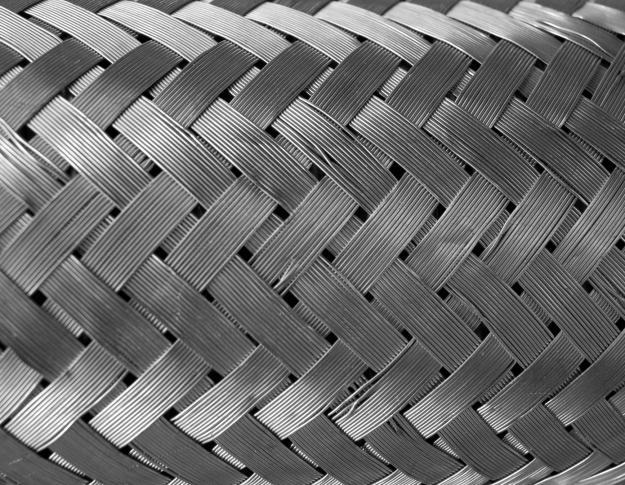

Supplement: Supplemental Information 3 [file peerj-cs-08-869-s003.zip › 1_part1/280_aided_0101.jpg]

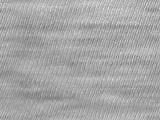

Supplement: Supplemental Information 3 [file peerj-cs-08-869-s003.zip › 1_part1/280_S_S_Cottn2_t.jpg]

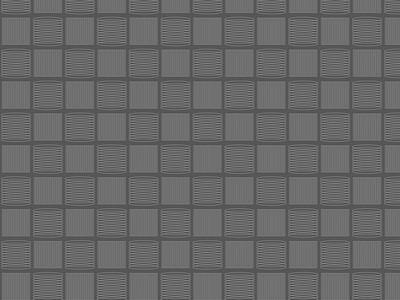

Supplement: Supplemental Information 3 [file peerj-cs-08-869-s003.zip › 1_part1/281_48018189013_13404e4f48_w.jpg]

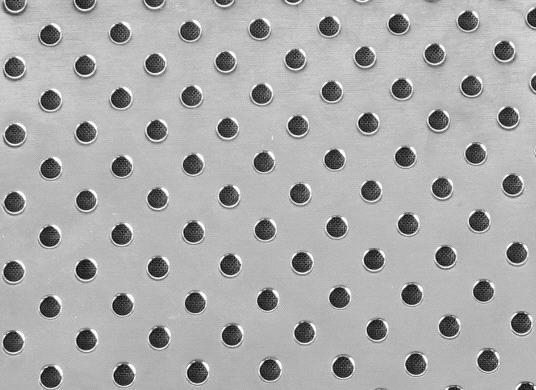

Supplement: Supplemental Information 3 [file peerj-cs-08-869-s003.zip › 1_part1/281_rforated_0076.jpg]

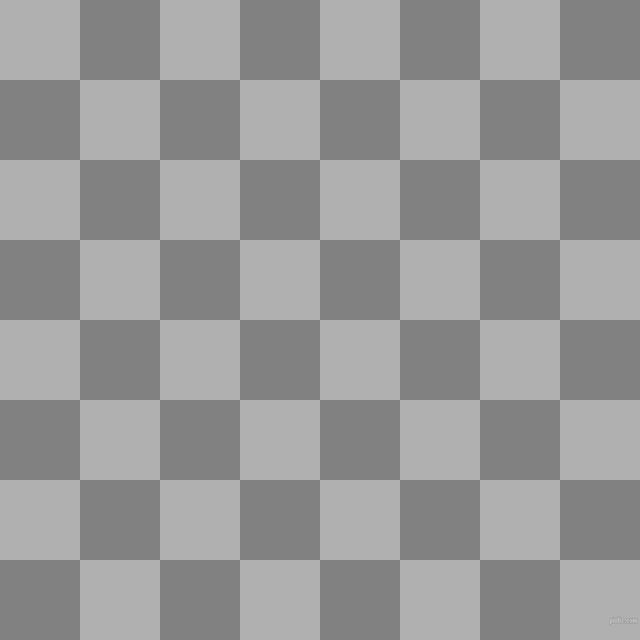

Supplement: Supplemental Information 3 [file peerj-cs-08-869-s003.zip › 1_part1/282_equered_0042.jpg]

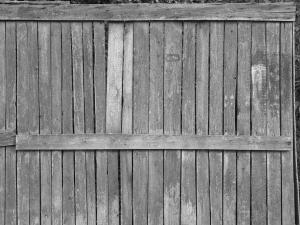

Supplement: Supplemental Information 3 [file peerj-cs-08-869-s003.zip › 1_part1/282_Planks old_102.jpg]

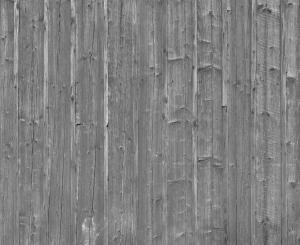

Supplement: Supplemental Information 3 [file peerj-cs-08-869-s003.zip › 1_part1/283_Planks old_17.jpg]

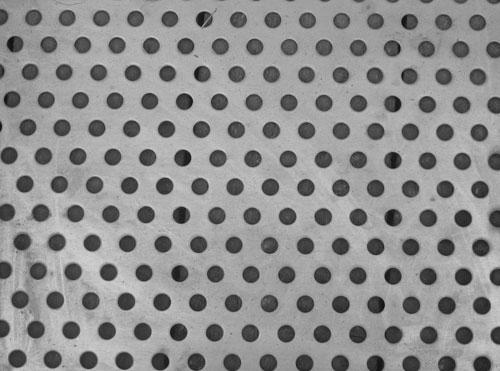

Supplement: Supplemental Information 3 [file peerj-cs-08-869-s003.zip › 1_part1/283_rforated_0016.jpg]

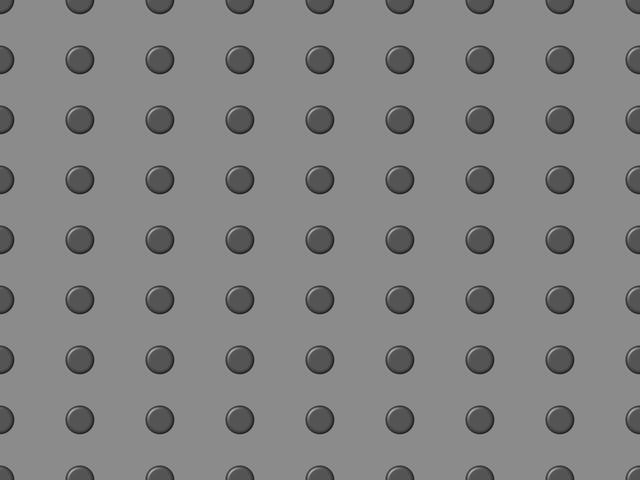

Supplement: Supplemental Information 3 [file peerj-cs-08-869-s003.zip › 1_part1/284_lka-dotted_0093.jpg]

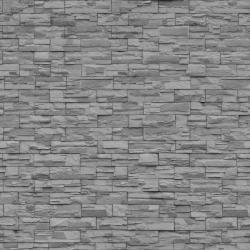

Supplement: Supplemental Information 3 [file peerj-cs-08-869-s003.zip › 1_part1/284_tile_tile_26.jpg]

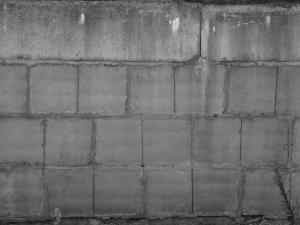

Supplement: Supplemental Information 3 [file peerj-cs-08-869-s003.zip › 1_part1/285_concrete massive_87.jpg]

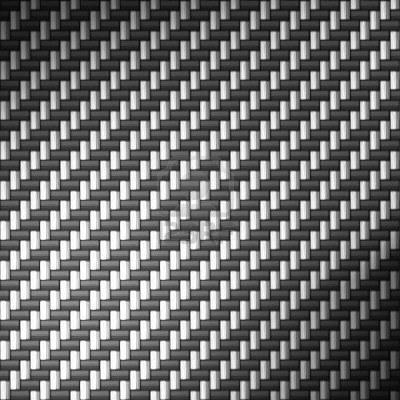

Supplement: Supplemental Information 3 [file peerj-cs-08-869-s003.zip › 1_part1/285_ven_0040.jpg]

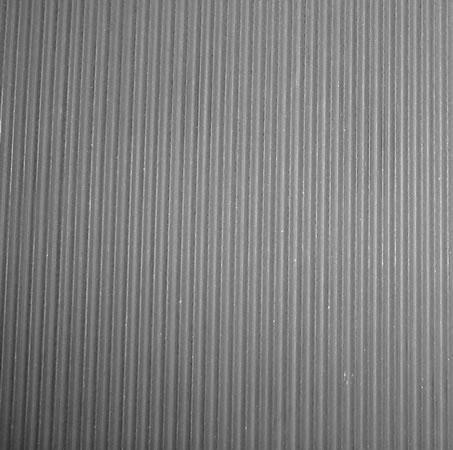

Supplement: Supplemental Information 3 [file peerj-cs-08-869-s003.zip › 1_part1/286_ooved_0139.jpg]

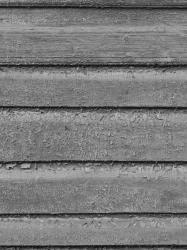

Supplement: Supplemental Information 3 [file peerj-cs-08-869-s003.zip › 1_part1/286_Planks old_56.jpg]

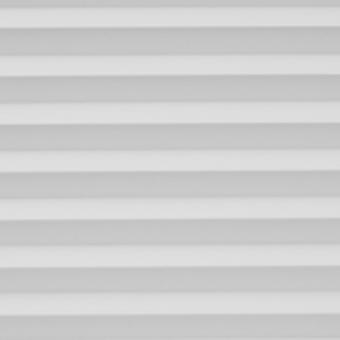

Supplement: Supplemental Information 3 [file peerj-cs-08-869-s003.zip › 1_part1/287_eated_0082.jpg]

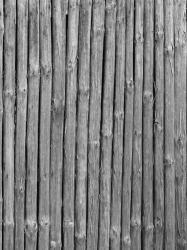

Supplement: Supplemental Information 3 [file peerj-cs-08-869-s003.zip › 1_part1/287_Planks old_44.jpg]

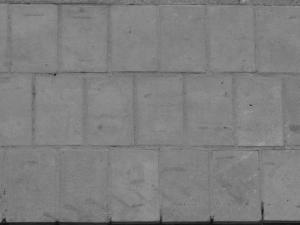

Supplement: Supplemental Information 3 [file peerj-cs-08-869-s003.zip › 1_part1/288_brick_pavement_117.jpg]

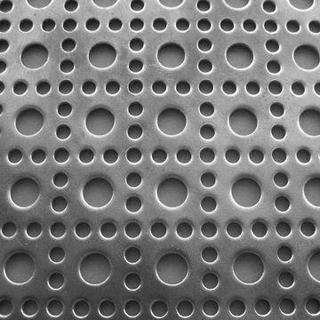

Supplement: Supplemental Information 3 [file peerj-cs-08-869-s003.zip › 1_part1/288_rforated_0097.jpg]

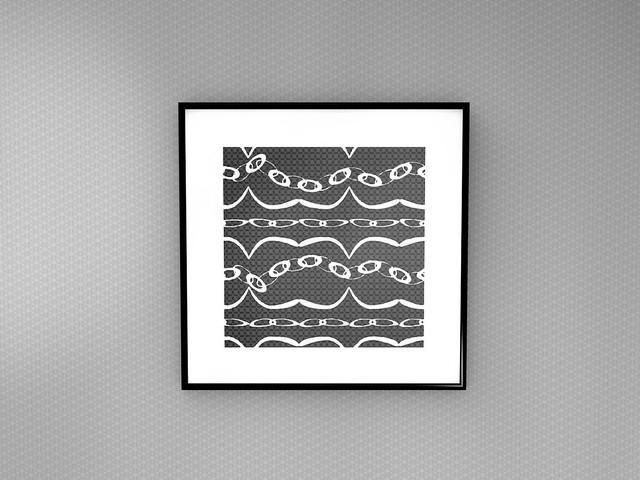

Supplement: Supplemental Information 3 [file peerj-cs-08-869-s003.zip › 1_part1/289_page5_6.jpg]

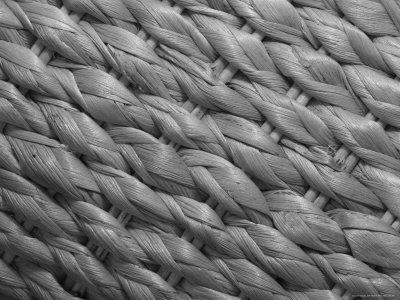

Supplement: Supplemental Information 3 [file peerj-cs-08-869-s003.zip › 1_part1/289_ven_0110.jpg]

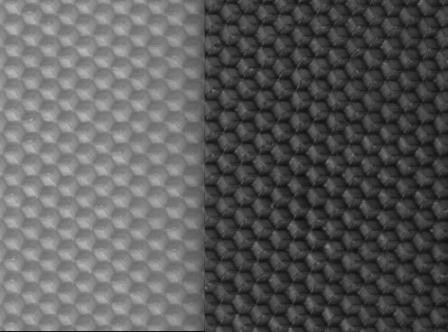

Supplement: Supplemental Information 3 [file peerj-cs-08-869-s003.zip › 1_part1/290_neycombed_0054.jpg]

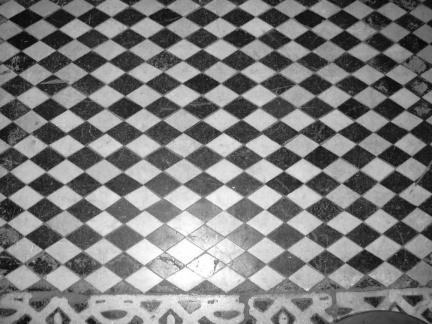

Supplement: Supplemental Information 3 [file peerj-cs-08-869-s003.zip › 1_part1/290_Pure Texture 171_14.jpg]

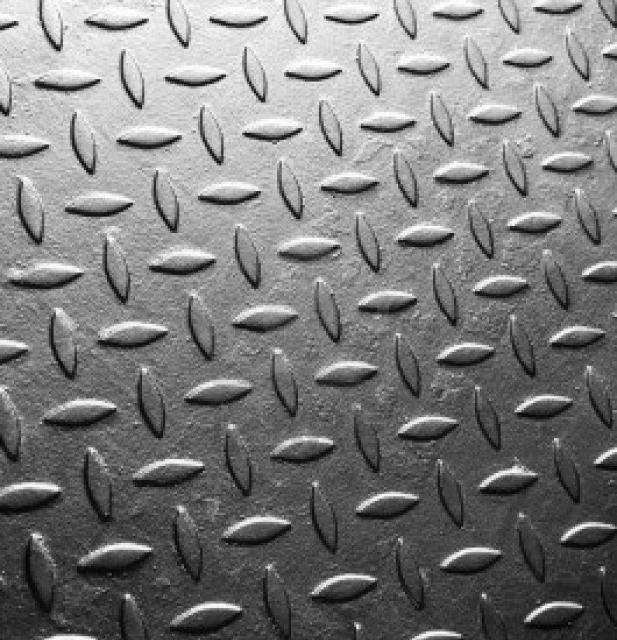

Supplement: Supplemental Information 3 [file peerj-cs-08-869-s003.zip › 1_part1/291_mpy_0149.jpg]

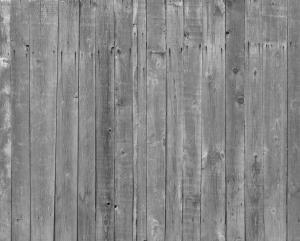

Supplement: Supplemental Information 3 [file peerj-cs-08-869-s003.zip › 1_part1/291_Planks old_88.jpg]

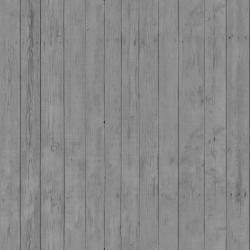

Supplement: Supplemental Information 3 [file peerj-cs-08-869-s003.zip › 1_part1/292_Planks old_37.jpg]

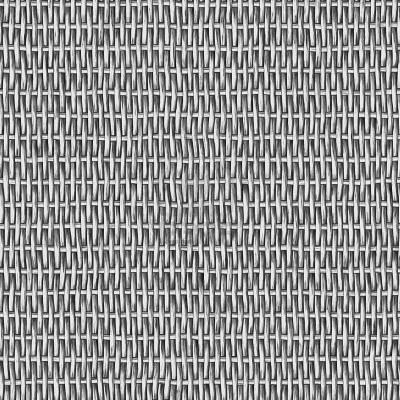

Supplement: Supplemental Information 3 [file peerj-cs-08-869-s003.zip › 1_part1/292_ven_0068.jpg]

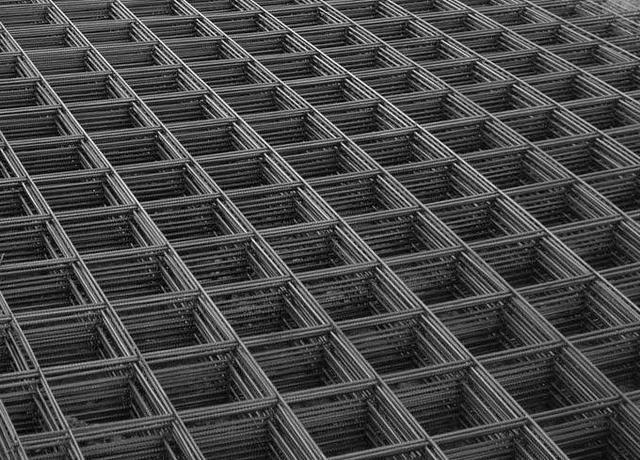

Supplement: Supplemental Information 3 [file peerj-cs-08-869-s003.zip › 1_part1/293_ffled_0148.jpg]

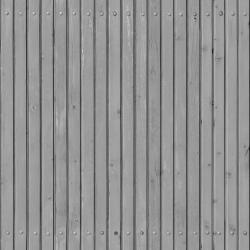

Supplement: Supplemental Information 3 [file peerj-cs-08-869-s003.zip › 1_part1/293_wood_planks_new_0031_01_thumb.jpg]

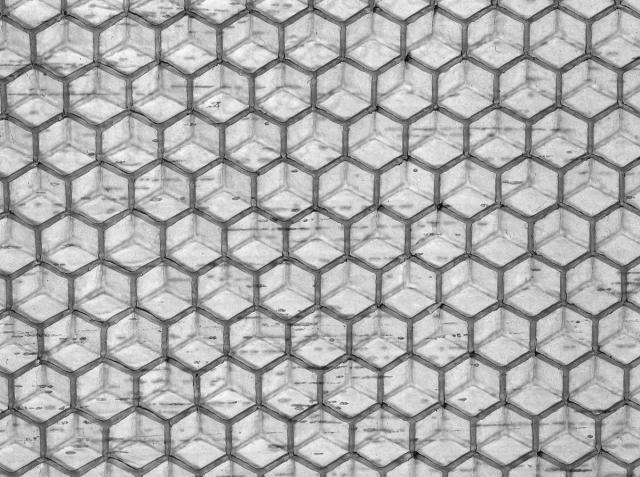

Supplement: Supplemental Information 3 [file peerj-cs-08-869-s003.zip › 1_part1/294_neycombed_0117.jpg]

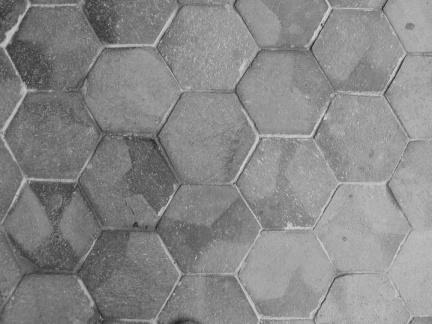

Supplement: Supplemental Information 3 [file peerj-cs-08-869-s003.zip › 1_part1/294_Pure Texture 171_141.jpg]

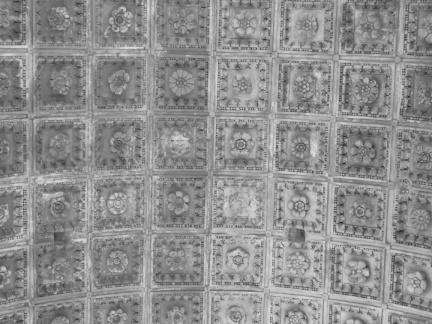

Supplement: Supplemental Information 3 [file peerj-cs-08-869-s003.zip › 1_part1/295_Pure Texture 171_10.jpg]

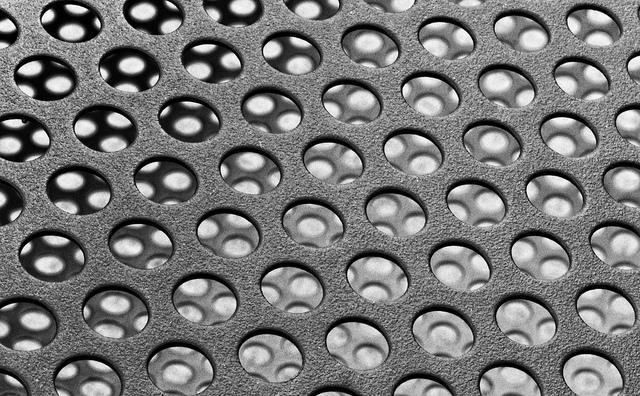

Supplement: Supplemental Information 3 [file peerj-cs-08-869-s003.zip › 1_part1/295_rforated_0023.jpg]

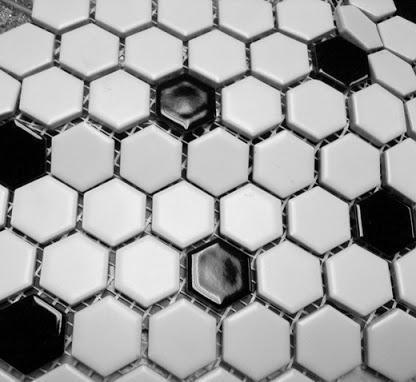

Supplement: Supplemental Information 3 [file peerj-cs-08-869-s003.zip › 1_part1/296_neycombed_0121.jpg]

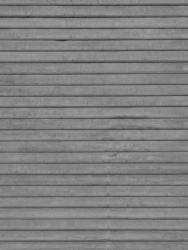

Supplement: Supplemental Information 3 [file peerj-cs-08-869-s003.zip › 1_part1/296_wood_planks_new_0029_01_thumb.jpg]
