# Supplementary figures and images for: Deep convolutional neural networks for regular texture recognition (part 8 of 8)
Source: PeerJ Comput Sci. 2022 Feb 9;8:e869. doi: 10.7717/peerj-cs.869 (PMC9044313; doi:10.7717/peerj-cs.869)

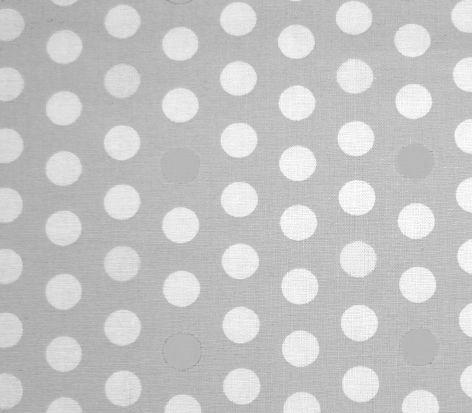

Supplement: Supplemental Information 4 [file peerj-cs-08-869-s004.zip › 1_part2/148_lka-dotted_0032.jpg]

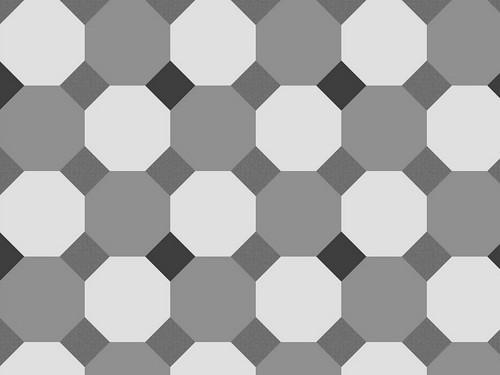

Supplement: Supplemental Information 4 [file peerj-cs-08-869-s004.zip › 1_part2/148_page11_1.jpg]

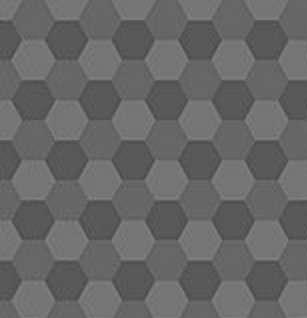

Supplement: Supplemental Information 4 [file peerj-cs-08-869-s004.zip › 1_part2/149_neycombed_0171.jpg]

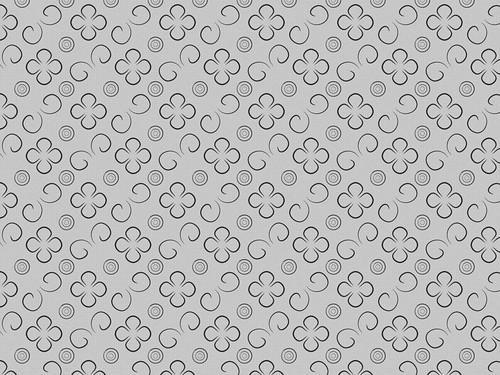

Supplement: Supplemental Information 4 [file peerj-cs-08-869-s004.zip › 1_part2/149_page12_8.jpg]

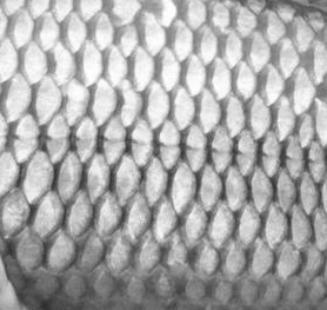

Supplement: Supplemental Information 4 [file peerj-cs-08-869-s004.zip › 1_part2/14_aly_0204.jpg]

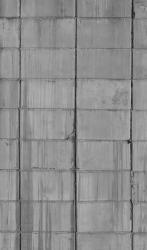

Supplement: Supplemental Information 4 [file peerj-cs-08-869-s004.zip › 1_part2/14_concrete massive_10.jpg]

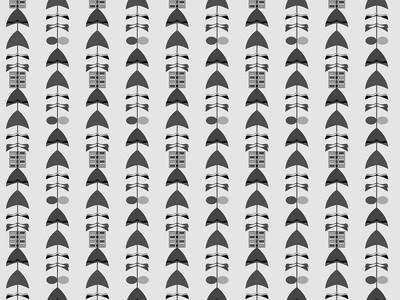

Supplement: Supplemental Information 4 [file peerj-cs-08-869-s004.zip › 1_part2/150_46528431465_86d364015f_w.jpg]

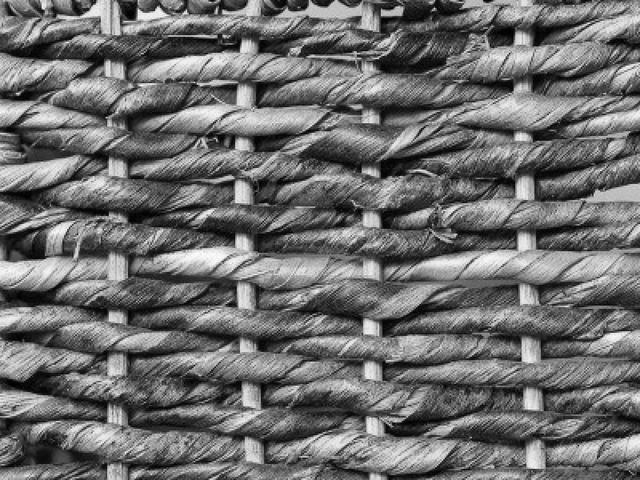

Supplement: Supplemental Information 4 [file peerj-cs-08-869-s004.zip › 1_part2/150_ven_0083.jpg]

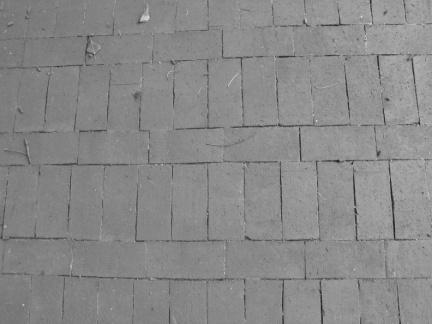

Supplement: Supplemental Information 4 [file peerj-cs-08-869-s004.zip › 1_part2/151_Pure Texture 171_108.jpg]

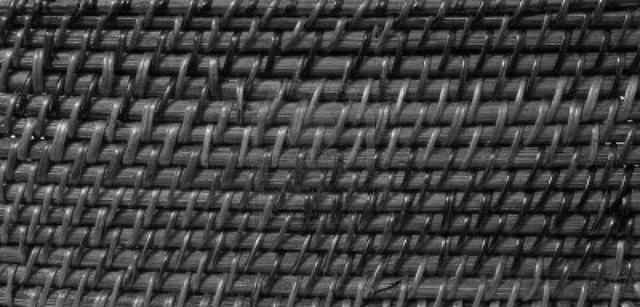

Supplement: Supplemental Information 4 [file peerj-cs-08-869-s004.zip › 1_part2/151_ven_0050.jpg]

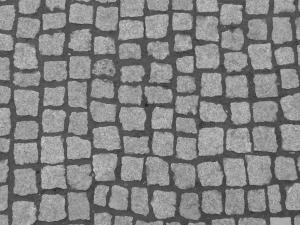

Supplement: Supplemental Information 4 [file peerj-cs-08-869-s004.zip › 1_part2/152_brick_pavement_71.jpg]

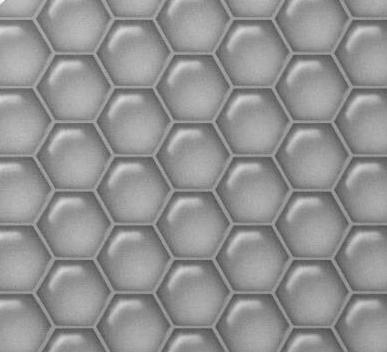

Supplement: Supplemental Information 4 [file peerj-cs-08-869-s004.zip › 1_part2/152_neycombed_0125.jpg]

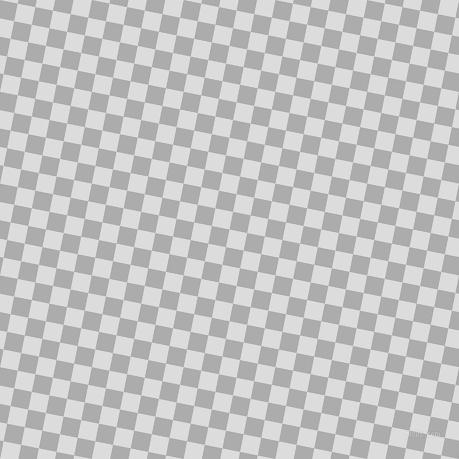

Supplement: Supplemental Information 4 [file peerj-cs-08-869-s004.zip › 1_part2/153_equered_0050.jpg]

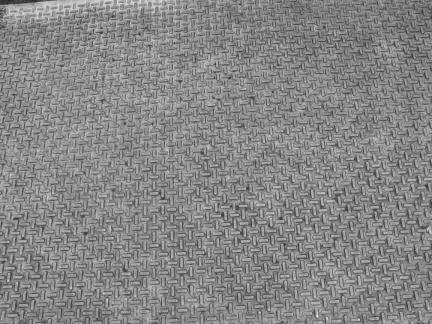

Supplement: Supplemental Information 4 [file peerj-cs-08-869-s004.zip › 1_part2/153_Pure Texture 171_5.jpg]

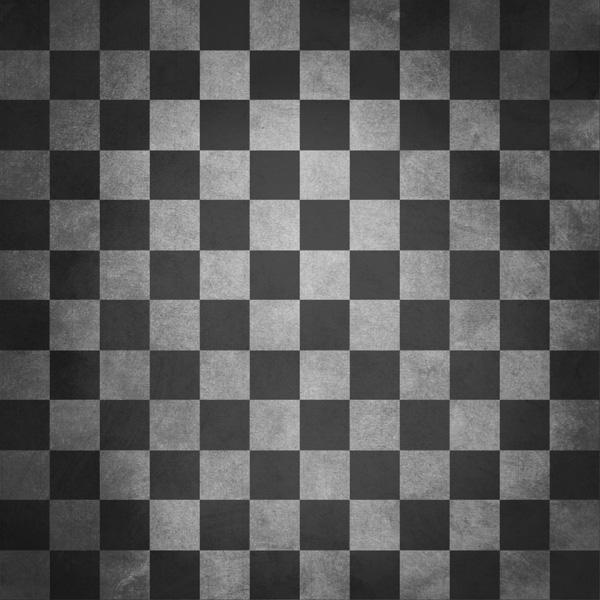

Supplement: Supplemental Information 4 [file peerj-cs-08-869-s004.zip › 1_part2/154_equered_0107.jpg]

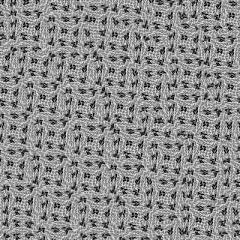

Supplement: Supplemental Information 4 [file peerj-cs-08-869-s004.zip › 1_part2/154_Graph cut texture synthesis results 92_12.jpg]

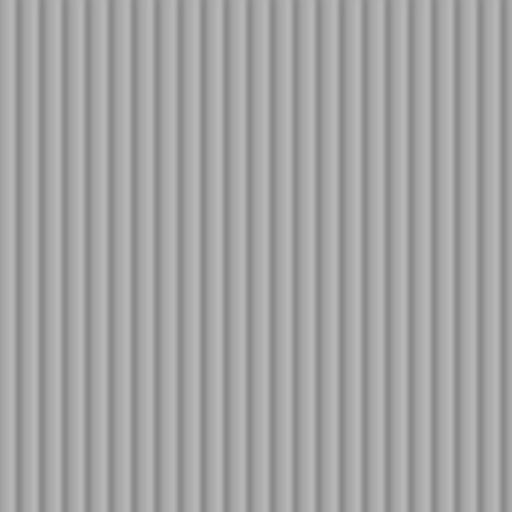

Supplement: Supplemental Information 4 [file peerj-cs-08-869-s004.zip › 1_part2/155_ooved_0078.jpg]

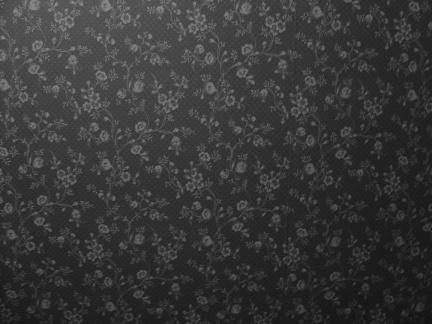

Supplement: Supplemental Information 4 [file peerj-cs-08-869-s004.zip › 1_part2/155_Pure Texture 171_33.jpg]

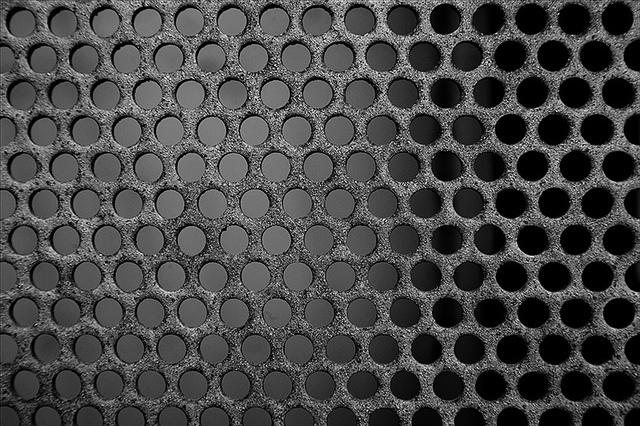

Supplement: Supplemental Information 4 [file peerj-cs-08-869-s004.zip › 1_part2/156_rforated_0025.jpg]

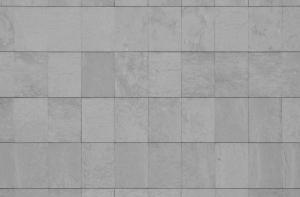

Supplement: Supplemental Information 4 [file peerj-cs-08-869-s004.zip › 1_part2/156_tile_tile_3.jpg]

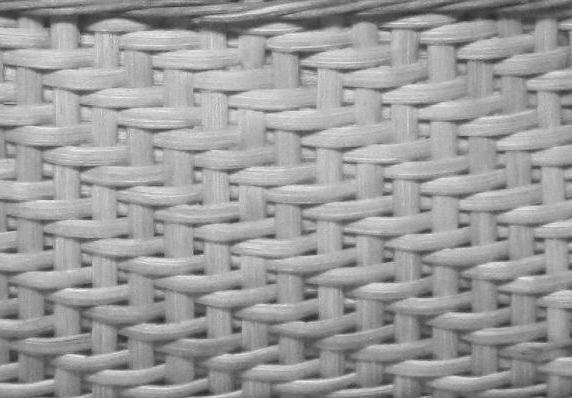

Supplement: Supplemental Information 4 [file peerj-cs-08-869-s004.zip › 1_part2/157_aided_0069.jpg]

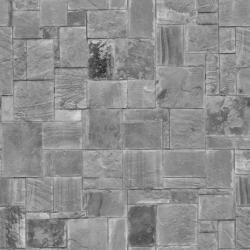

Supplement: Supplemental Information 4 [file peerj-cs-08-869-s004.zip › 1_part2/157_stone_wall_105.jpg]

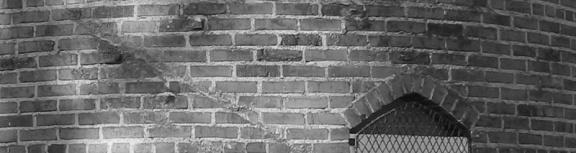

Supplement: Supplemental Information 4 [file peerj-cs-08-869-s004.zip › 1_part2/158_Normal nrt images 68_60.jpg]

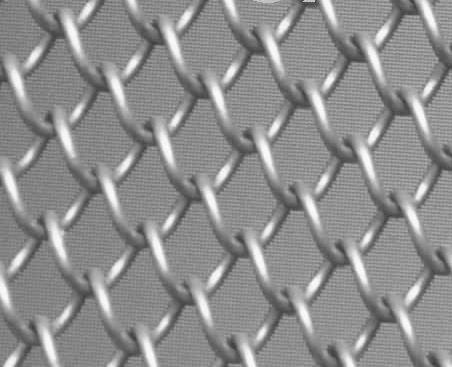

Supplement: Supplemental Information 4 [file peerj-cs-08-869-s004.zip › 1_part2/158_shed_0138.jpg]

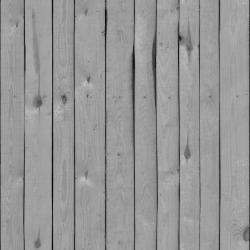

Supplement: Supplemental Information 4 [file peerj-cs-08-869-s004.zip › 1_part2/159_Planks new_6.jpg]

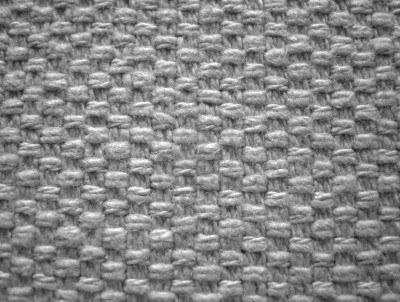

Supplement: Supplemental Information 4 [file peerj-cs-08-869-s004.zip › 1_part2/159_ven_0088.jpg]

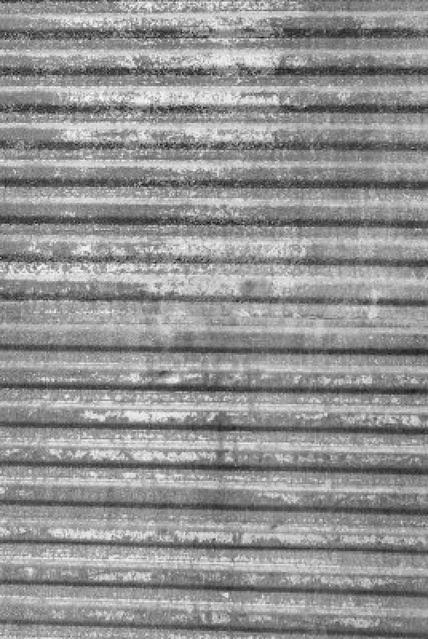

Supplement: Supplemental Information 4 [file peerj-cs-08-869-s004.zip › 1_part2/15_ooved_0081.jpg]

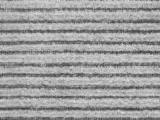

Supplement: Supplemental Information 4 [file peerj-cs-08-869-s004.zip › 1_part2/15_S_S_Cordry_t.jpg]

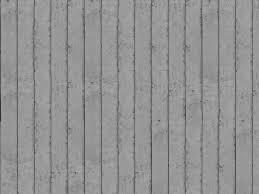

Supplement: Supplemental Information 4 [file peerj-cs-08-869-s004.zip › 1_part2/160_images (3).jpg]

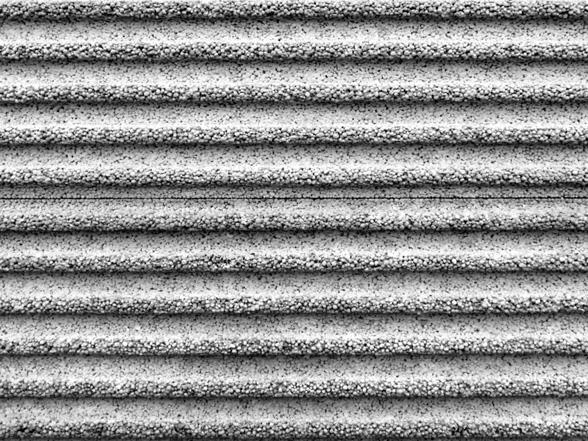

Supplement: Supplemental Information 4 [file peerj-cs-08-869-s004.zip › 1_part2/160_ooved_0089.jpg]

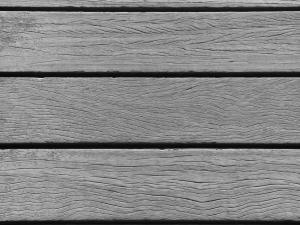

Supplement: Supplemental Information 4 [file peerj-cs-08-869-s004.zip › 1_part2/161_Planks old_14.jpg]

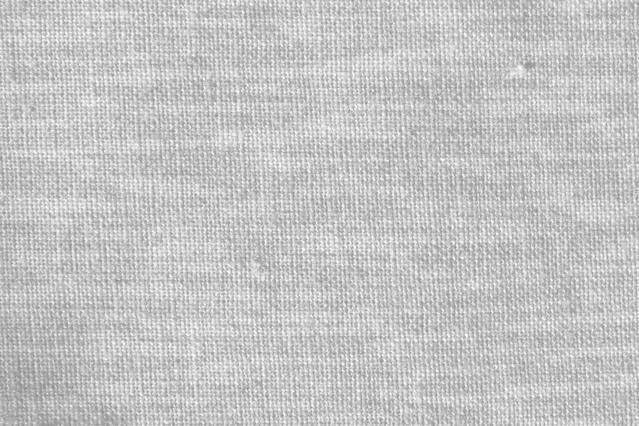

Supplement: Supplemental Information 4 [file peerj-cs-08-869-s004.zip › 1_part2/161_ven_0012.jpg]

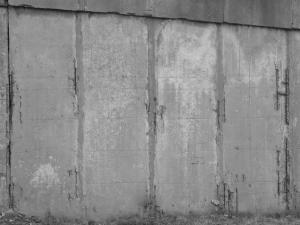

Supplement: Supplemental Information 4 [file peerj-cs-08-869-s004.zip › 1_part2/162_concrete massive_50.jpg]

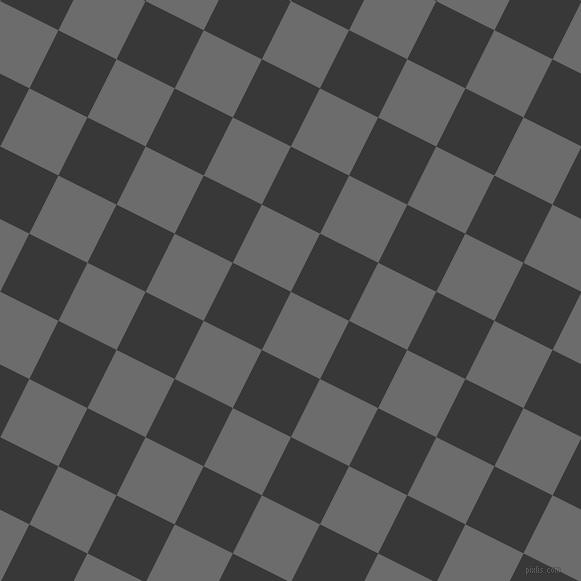

Supplement: Supplemental Information 4 [file peerj-cs-08-869-s004.zip › 1_part2/162_equered_0044.jpg]

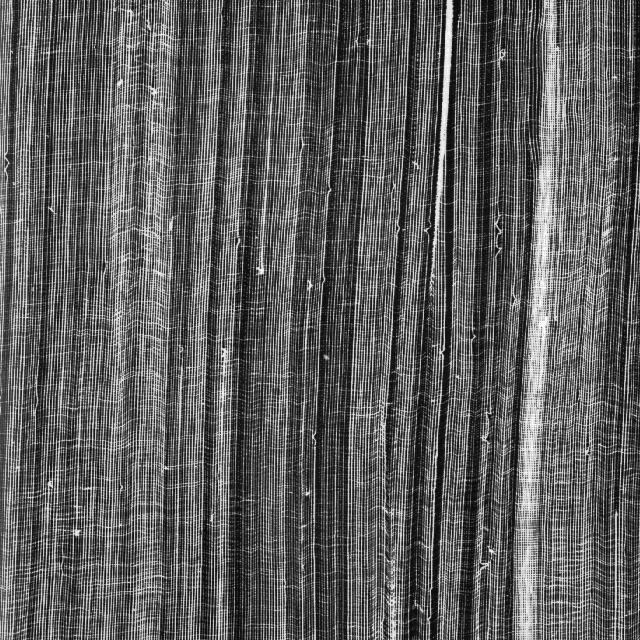

Supplement: Supplemental Information 4 [file peerj-cs-08-869-s004.zip › 1_part2/163_D105.jpg]

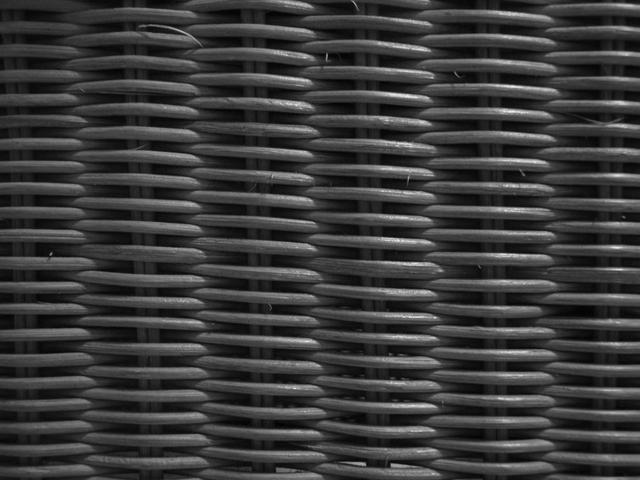

Supplement: Supplemental Information 4 [file peerj-cs-08-869-s004.zip › 1_part2/163_ven_0021.jpg]

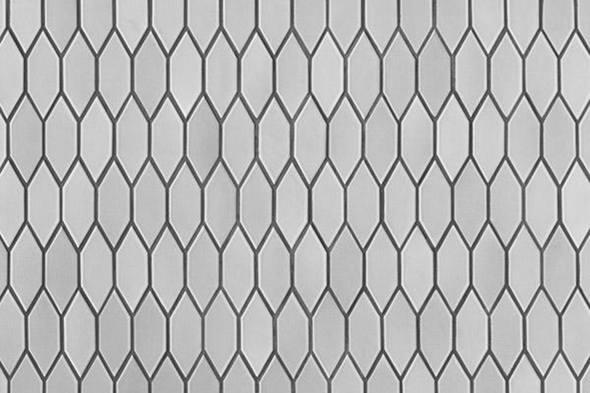

Supplement: Supplemental Information 4 [file peerj-cs-08-869-s004.zip › 1_part2/164_neycombed_0061.jpg]

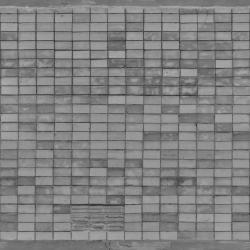

Supplement: Supplemental Information 4 [file peerj-cs-08-869-s004.zip › 1_part2/164_tile_tile_25.jpg]

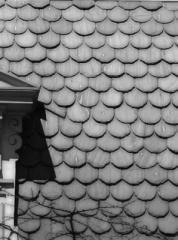

Supplement: Supplemental Information 4 [file peerj-cs-08-869-s004.zip › 1_part2/165_Normal nrt images 68_36.jpg]

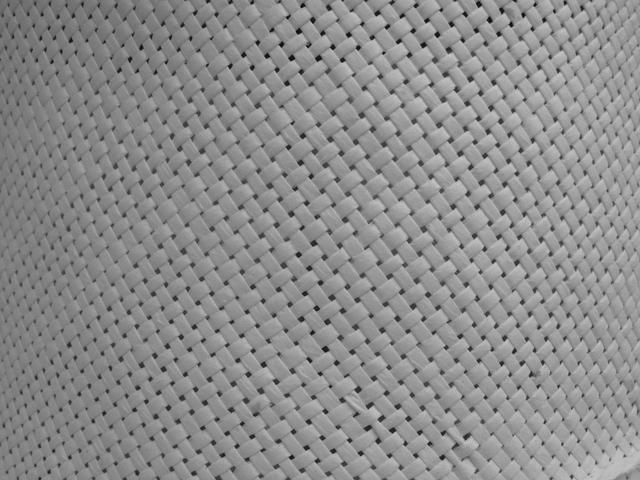

Supplement: Supplemental Information 4 [file peerj-cs-08-869-s004.zip › 1_part2/165_ven_0097.jpg]

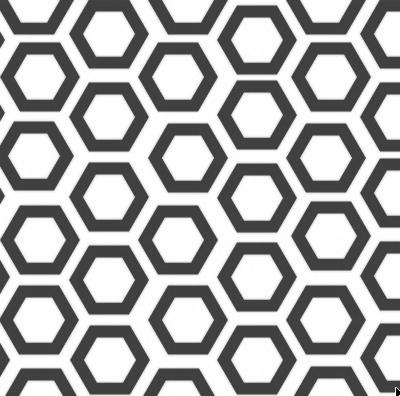

Supplement: Supplemental Information 4 [file peerj-cs-08-869-s004.zip › 1_part2/166_neycombed_0078.jpg]

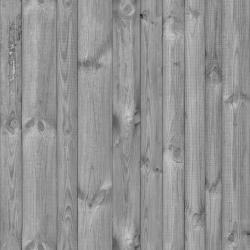

Supplement: Supplemental Information 4 [file peerj-cs-08-869-s004.zip › 1_part2/166_Planks new_10.jpg]

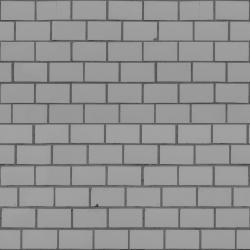

Supplement: Supplemental Information 4 [file peerj-cs-08-869-s004.zip › 1_part2/167_tile_tile_36.jpg]

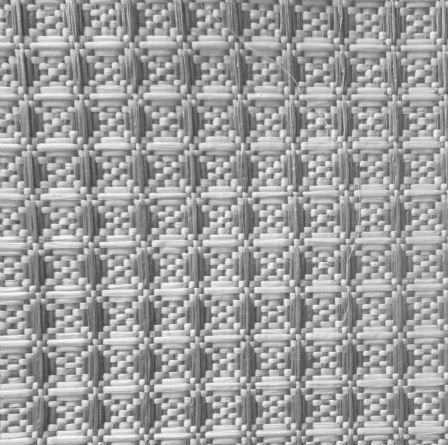

Supplement: Supplemental Information 4 [file peerj-cs-08-869-s004.zip › 1_part2/167_ven_0126.jpg]

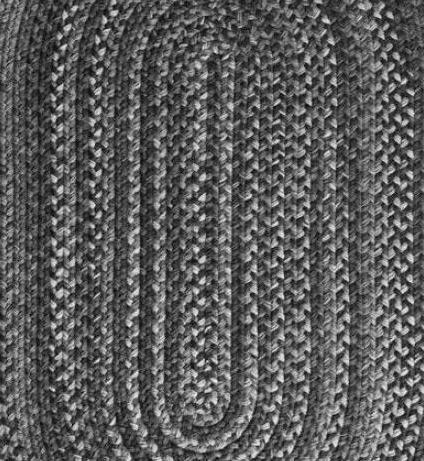

Supplement: Supplemental Information 4 [file peerj-cs-08-869-s004.zip › 1_part2/168_aided_0006.jpg]

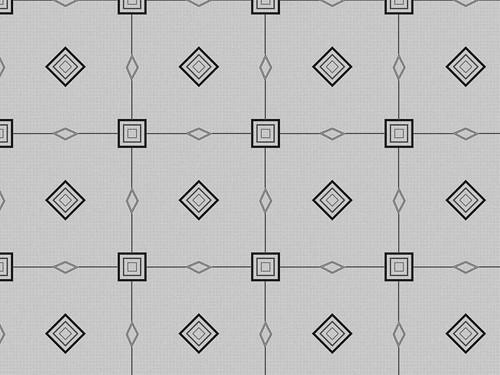

Supplement: Supplemental Information 4 [file peerj-cs-08-869-s004.zip › 1_part2/168_page12_17.jpg]

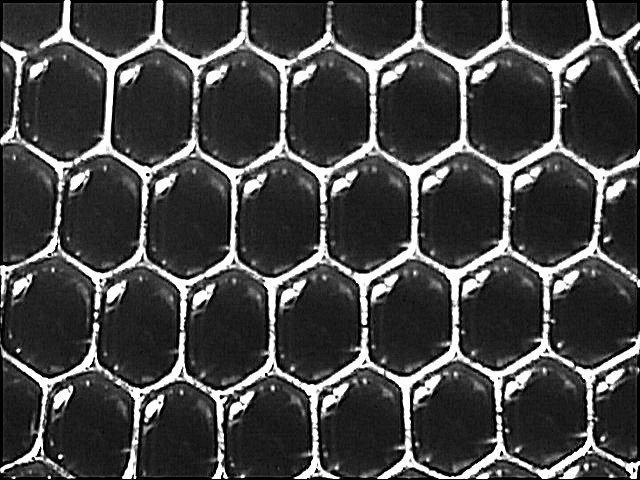

Supplement: Supplemental Information 4 [file peerj-cs-08-869-s004.zip › 1_part2/169_neycombed_0112.jpg]

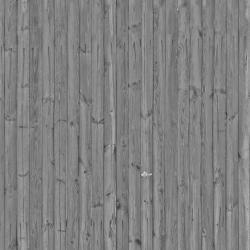

Supplement: Supplemental Information 4 [file peerj-cs-08-869-s004.zip › 1_part2/169_Planks new_25.jpg]

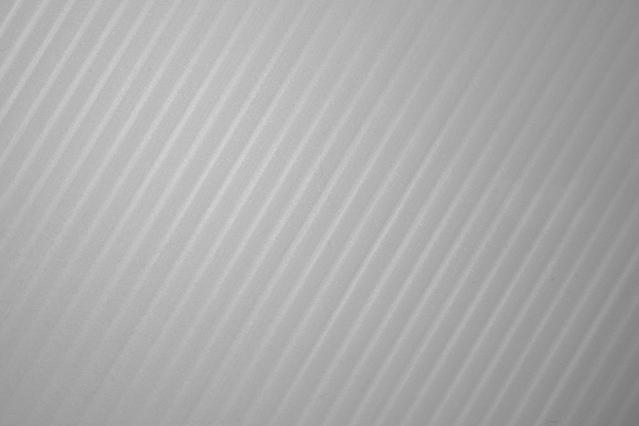

Supplement: Supplemental Information 4 [file peerj-cs-08-869-s004.zip › 1_part2/16_ooved_0098.jpg]

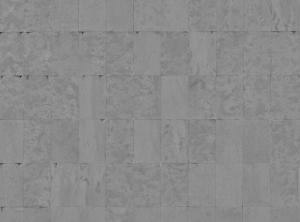

Supplement: Supplemental Information 4 [file peerj-cs-08-869-s004.zip › 1_part2/16_tile_tile_41.jpg]

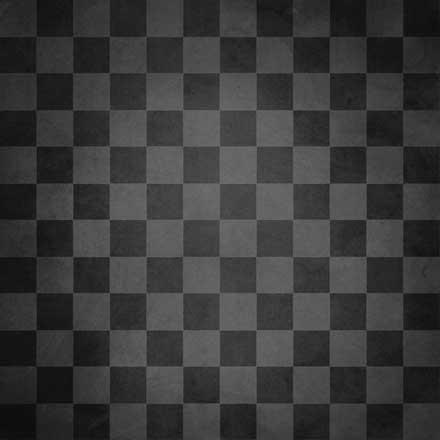

Supplement: Supplemental Information 4 [file peerj-cs-08-869-s004.zip › 1_part2/170_equered_0070.jpg]

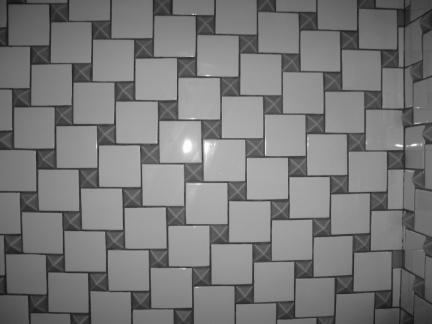

Supplement: Supplemental Information 4 [file peerj-cs-08-869-s004.zip › 1_part2/170_Pure Texture 171_167.jpg]

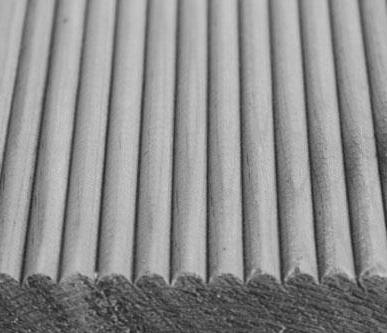

Supplement: Supplemental Information 4 [file peerj-cs-08-869-s004.zip › 1_part2/171_ooved_0144.jpg]

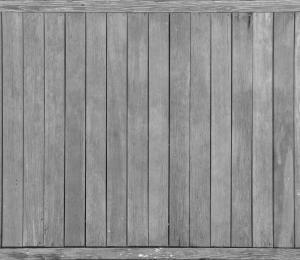

Supplement: Supplemental Information 4 [file peerj-cs-08-869-s004.zip › 1_part2/171_Planks old_4.jpg]

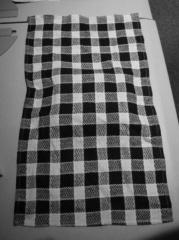

Supplement: Supplemental Information 4 [file peerj-cs-08-869-s004.zip › 1_part2/172_Normal nrt images 68_43.jpg]

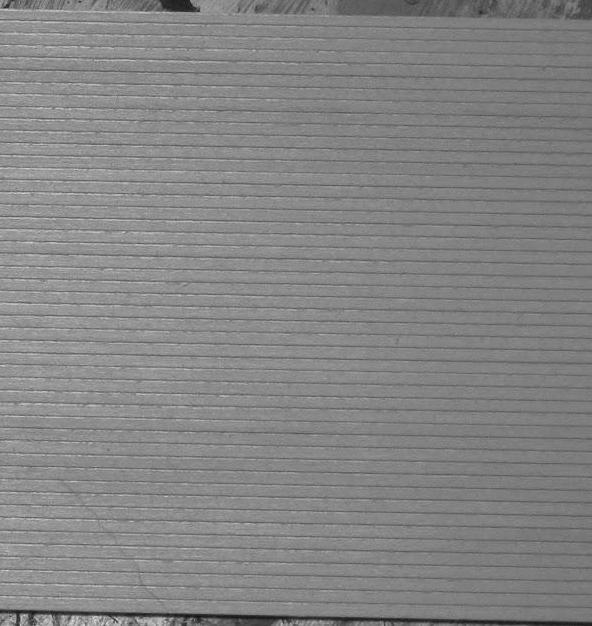

Supplement: Supplemental Information 4 [file peerj-cs-08-869-s004.zip › 1_part2/172_ooved_0125.jpg]

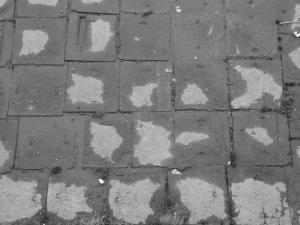

Supplement: Supplemental Information 4 [file peerj-cs-08-869-s004.zip › 1_part2/173_brick_pavement_75.jpg]

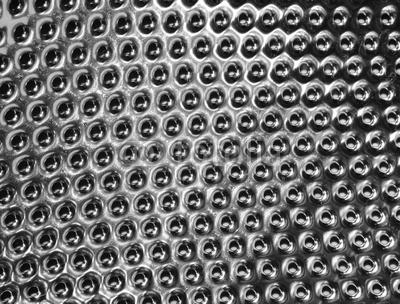

Supplement: Supplemental Information 4 [file peerj-cs-08-869-s004.zip › 1_part2/173_rforated_0034.jpg]

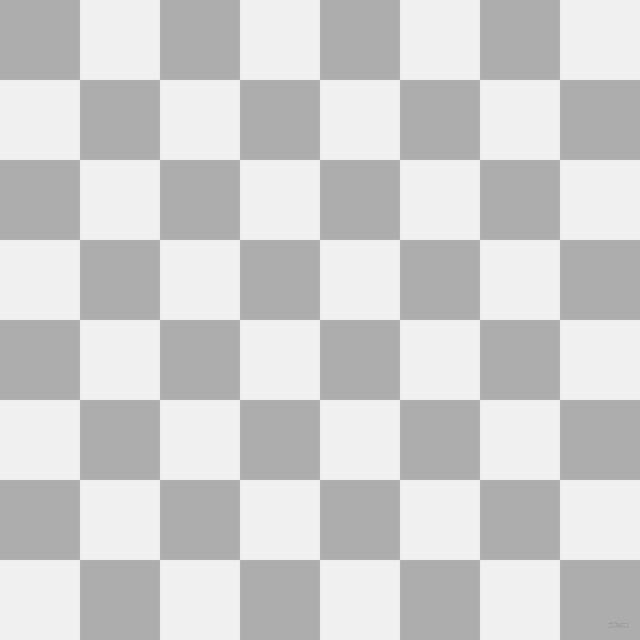

Supplement: Supplemental Information 4 [file peerj-cs-08-869-s004.zip › 1_part2/174_equered_0048.jpg]

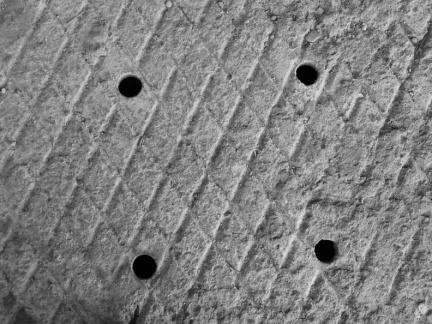

Supplement: Supplemental Information 4 [file peerj-cs-08-869-s004.zip › 1_part2/174_Pure Texture 171_112.jpg]

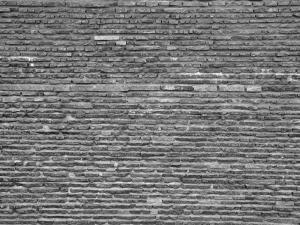

Supplement: Supplemental Information 4 [file peerj-cs-08-869-s004.zip › 1_part2/175_stone_wall_113.jpg]

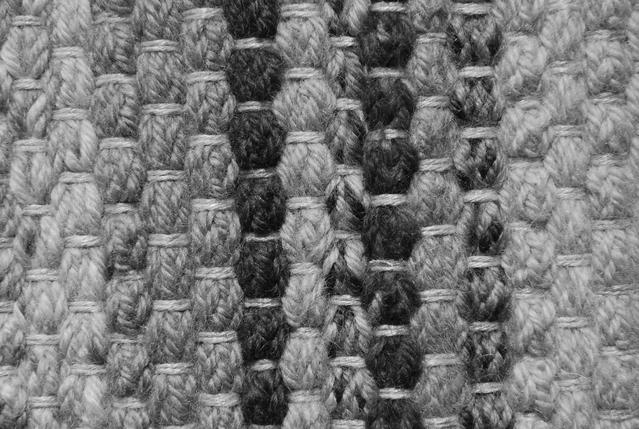

Supplement: Supplemental Information 4 [file peerj-cs-08-869-s004.zip › 1_part2/175_ven_0036.jpg]

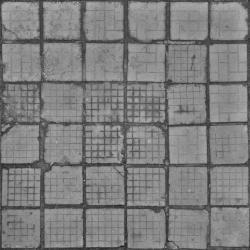

Supplement: Supplemental Information 4 [file peerj-cs-08-869-s004.zip › 1_part2/176_brick_pavement_87.jpg]

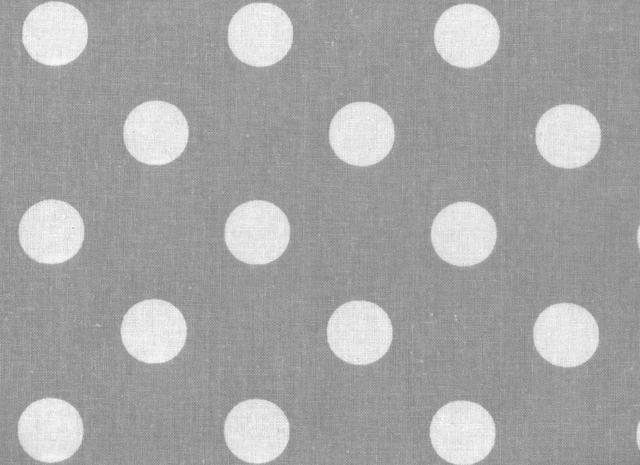

Supplement: Supplemental Information 4 [file peerj-cs-08-869-s004.zip › 1_part2/176_lka-dotted_0114.jpg]

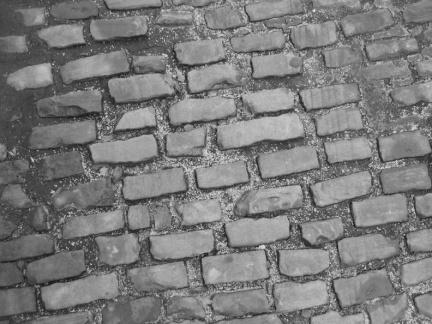

Supplement: Supplemental Information 4 [file peerj-cs-08-869-s004.zip › 1_part2/177_Borderline Near-Regular Textures 65_55.jpg]

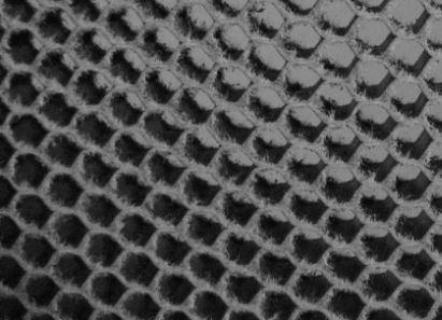

Supplement: Supplemental Information 4 [file peerj-cs-08-869-s004.zip › 1_part2/177_neycombed_0132.jpg]

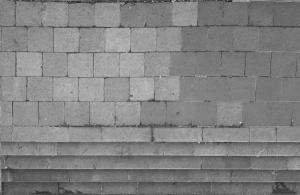

Supplement: Supplemental Information 4 [file peerj-cs-08-869-s004.zip › 1_part2/178_brick_pavement_130.jpg]

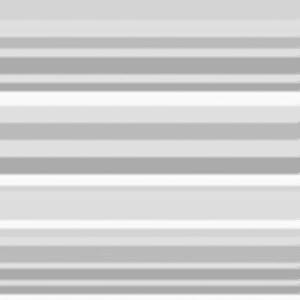

Supplement: Supplemental Information 4 [file peerj-cs-08-869-s004.zip › 1_part2/178_nded_0010.jpg]

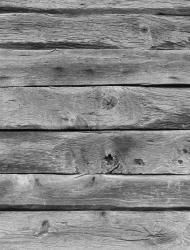

Supplement: Supplemental Information 4 [file peerj-cs-08-869-s004.zip › 1_part2/179_Planks old_21.jpg]

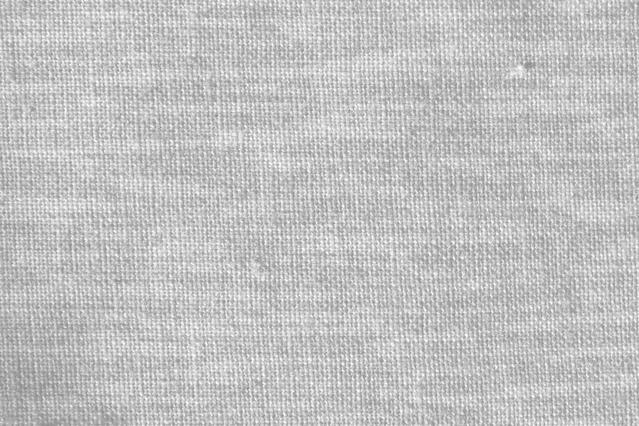

Supplement: Supplemental Information 4 [file peerj-cs-08-869-s004.zip › 1_part2/179_ven_0009.jpg]

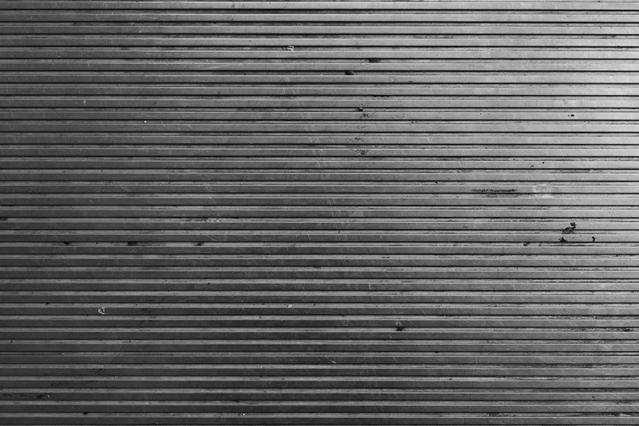

Supplement: Supplemental Information 4 [file peerj-cs-08-869-s004.zip › 1_part2/17_ooved_0099.jpg]

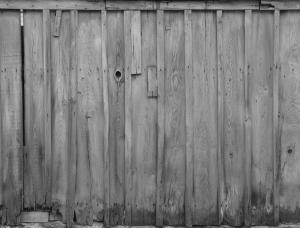

Supplement: Supplemental Information 4 [file peerj-cs-08-869-s004.zip › 1_part2/17_Planks old_38.jpg]

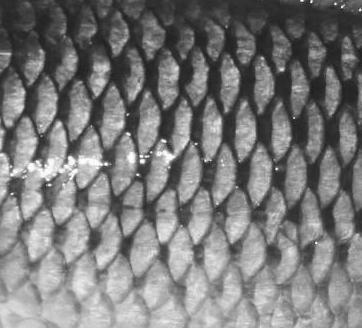

Supplement: Supplemental Information 4 [file peerj-cs-08-869-s004.zip › 1_part2/180_aly_0173.jpg]

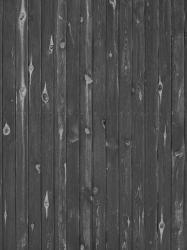

Supplement: Supplemental Information 4 [file peerj-cs-08-869-s004.zip › 1_part2/180_Planks old_63.jpg]

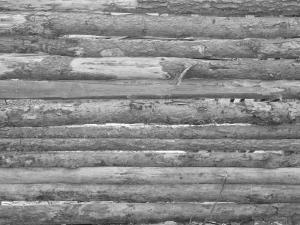

Supplement: Supplemental Information 4 [file peerj-cs-08-869-s004.zip › 1_part2/181_Planks new_39.jpg]

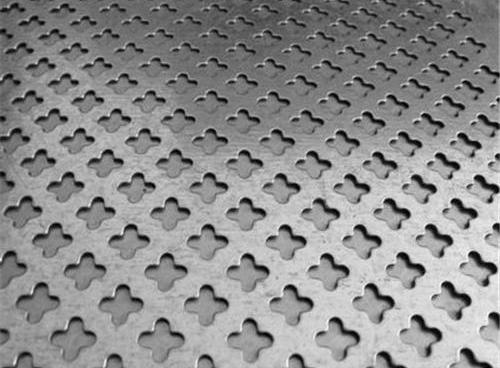

Supplement: Supplemental Information 4 [file peerj-cs-08-869-s004.zip › 1_part2/181_rforated_0063.jpg]

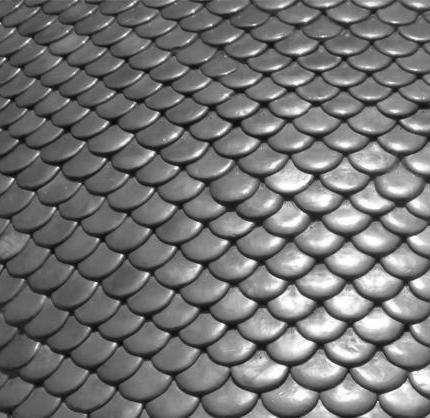

Supplement: Supplemental Information 4 [file peerj-cs-08-869-s004.zip › 1_part2/182_aly_0222.jpg]

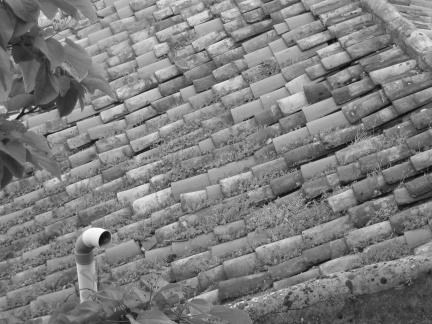

Supplement: Supplemental Information 4 [file peerj-cs-08-869-s004.zip › 1_part2/182_Borderline Near-Regular Textures 65_64.jpg]

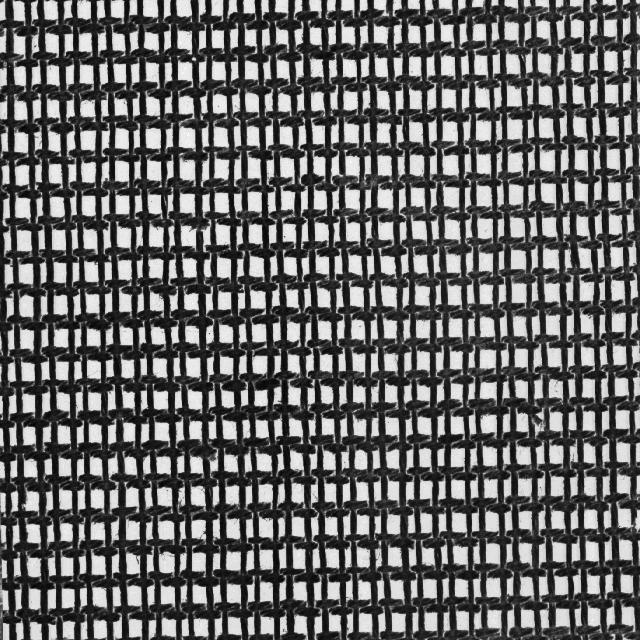

Supplement: Supplemental Information 4 [file peerj-cs-08-869-s004.zip › 1_part2/183_D20.jpg]

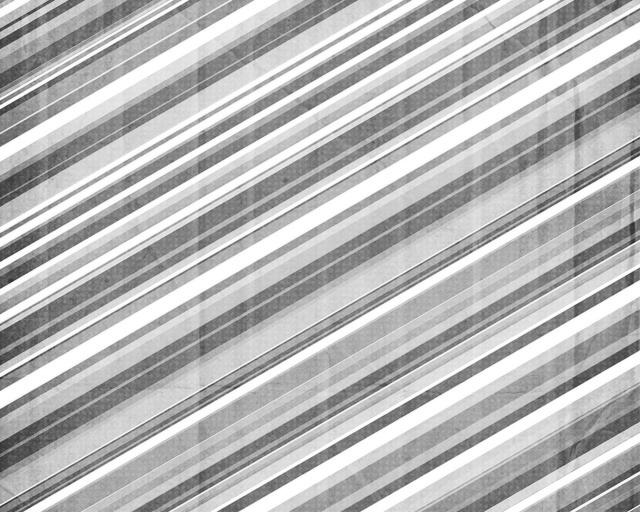

Supplement: Supplemental Information 4 [file peerj-cs-08-869-s004.zip › 1_part2/183_nded_0062.jpg]

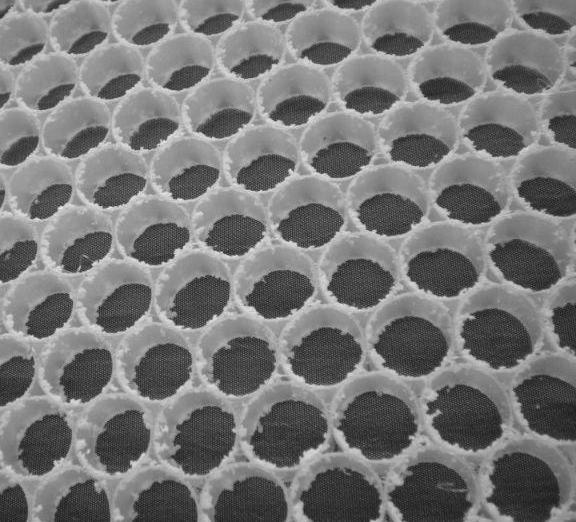

Supplement: Supplemental Information 4 [file peerj-cs-08-869-s004.zip › 1_part2/184_neycombed_0139.jpg]

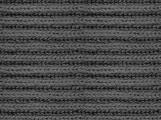

Supplement: Supplemental Information 4 [file peerj-cs-08-869-s004.zip › 1_part2/184_S_S_Weave2_t.jpg]

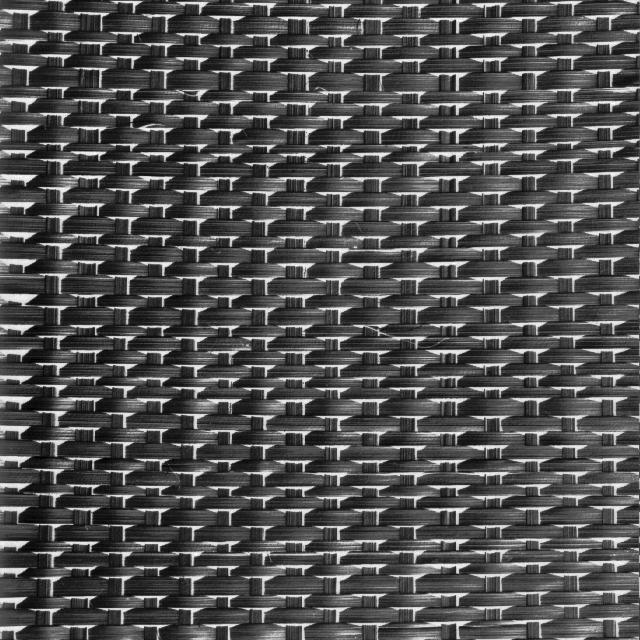

Supplement: Supplemental Information 4 [file peerj-cs-08-869-s004.zip › 1_part2/185_D65.jpg]

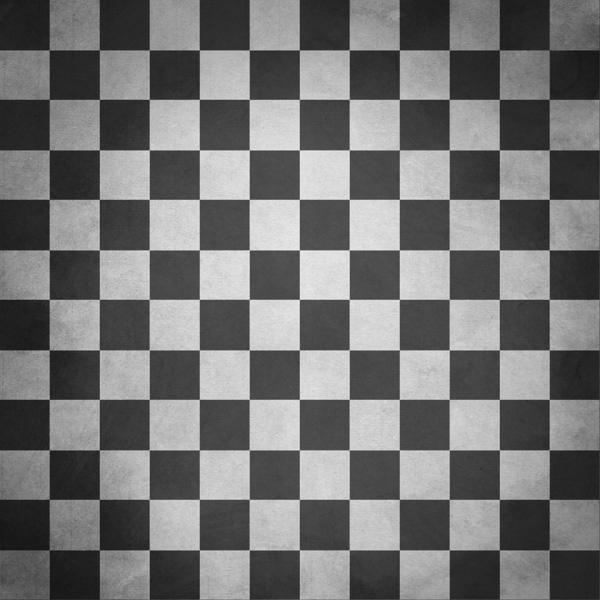

Supplement: Supplemental Information 4 [file peerj-cs-08-869-s004.zip › 1_part2/185_equered_0084.jpg]

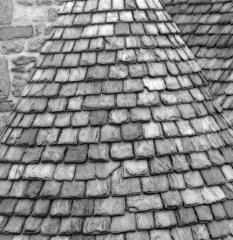

Supplement: Supplemental Information 4 [file peerj-cs-08-869-s004.zip › 1_part2/186_Normal nrt images 68_23.jpg]

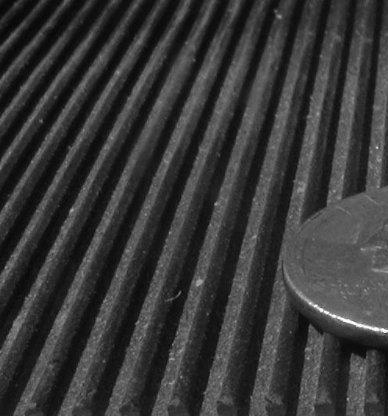

Supplement: Supplemental Information 4 [file peerj-cs-08-869-s004.zip › 1_part2/186_ooved_0149.jpg]

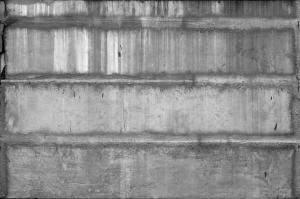

Supplement: Supplemental Information 4 [file peerj-cs-08-869-s004.zip › 1_part2/187_concrete massive_71.jpg]

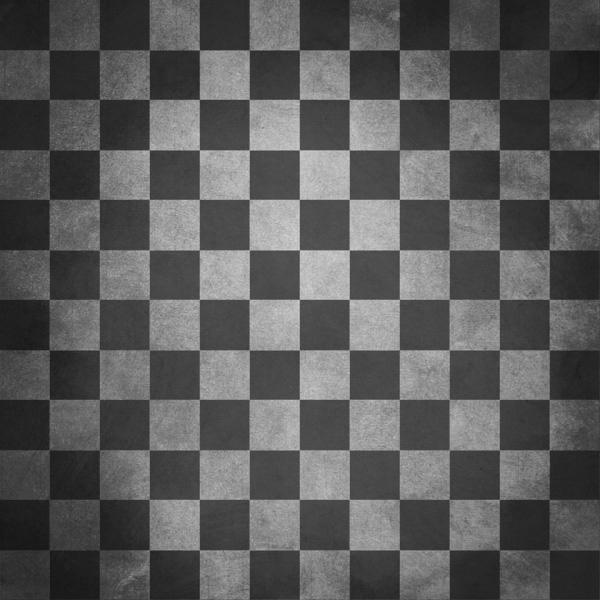

Supplement: Supplemental Information 4 [file peerj-cs-08-869-s004.zip › 1_part2/187_equered_0093.jpg]

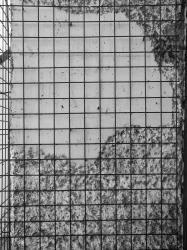

Supplement: Supplemental Information 4 [file peerj-cs-08-869-s004.zip › 1_part2/188_concrete other_12.jpg]

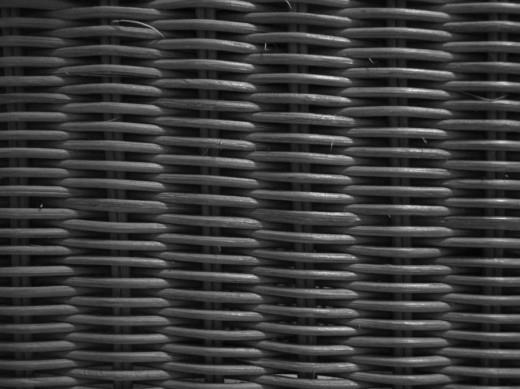

Supplement: Supplemental Information 4 [file peerj-cs-08-869-s004.zip › 1_part2/188_ven_0086.jpg]

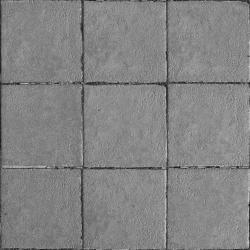

Supplement: Supplemental Information 4 [file peerj-cs-08-869-s004.zip › 1_part2/189_brick_pavement_66.jpg]

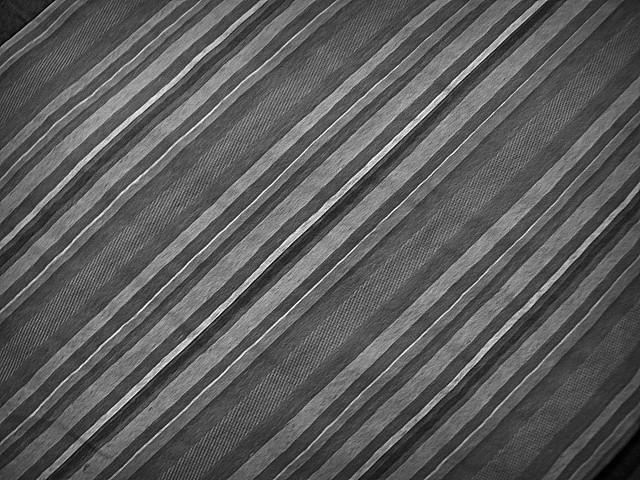

Supplement: Supplemental Information 4 [file peerj-cs-08-869-s004.zip › 1_part2/189_nded_0022.jpg]

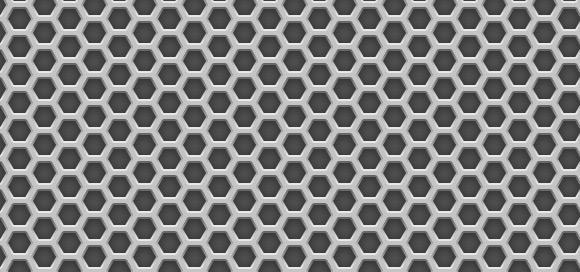

Supplement: Supplemental Information 4 [file peerj-cs-08-869-s004.zip › 1_part2/18_08_free_subtle_textures_seamless_light_metal_grid_patterns.jpg]

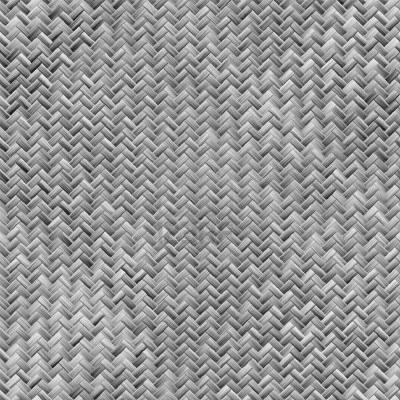

Supplement: Supplemental Information 4 [file peerj-cs-08-869-s004.zip › 1_part2/18_ven_0049.jpg]

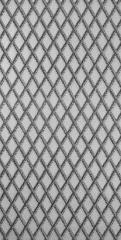

Supplement: Supplemental Information 4 [file peerj-cs-08-869-s004.zip › 1_part2/190_20150911134909-4c8f8071-2s.jpg]

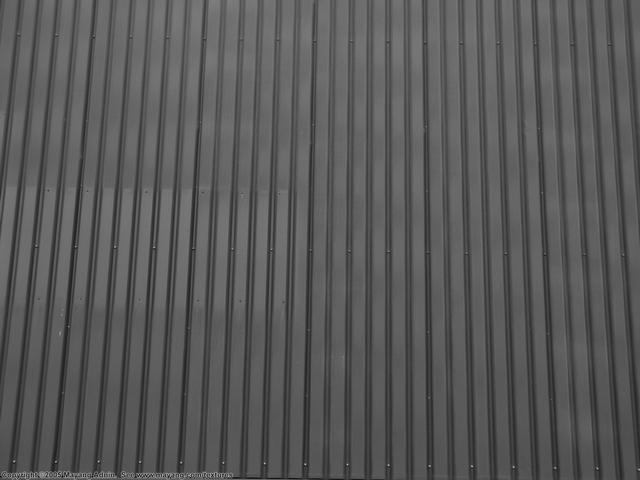

Supplement: Supplemental Information 4 [file peerj-cs-08-869-s004.zip › 1_part2/190_ooved_0093.jpg]
