# Supplementary figures and images for: Deep convolutional neural networks for regular texture recognition (part 7 of 8)
Source: PeerJ Comput Sci. 2022 Feb 9;8:e869. doi: 10.7717/peerj-cs.869 (PMC9044313; doi:10.7717/peerj-cs.869)

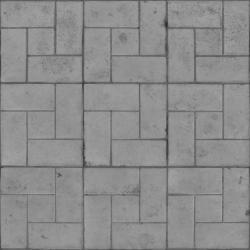

Supplement: Supplemental Information 4 [file peerj-cs-08-869-s004.zip › 1_part2/101_brick_pavement_59.jpg]

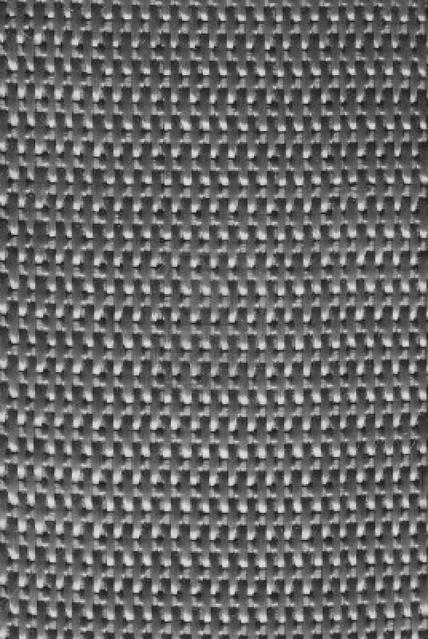

Supplement: Supplemental Information 4 [file peerj-cs-08-869-s004.zip › 1_part2/101_ven_0005.jpg]

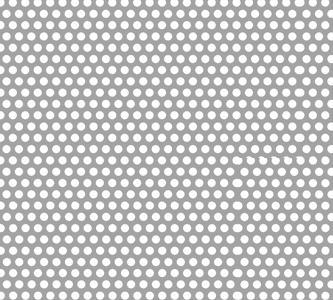

Supplement: Supplemental Information 4 [file peerj-cs-08-869-s004.zip › 1_part2/102_rforated_0080.jpg]

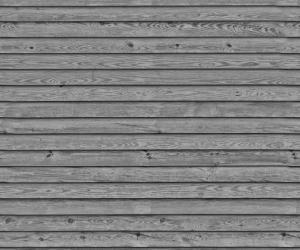

Supplement: Supplemental Information 4 [file peerj-cs-08-869-s004.zip › 1_part2/102_thumb (3).jpg]

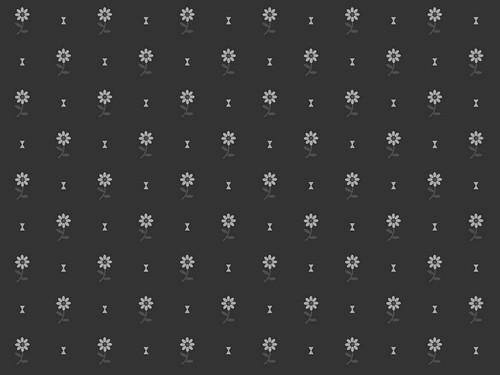

Supplement: Supplemental Information 4 [file peerj-cs-08-869-s004.zip › 1_part2/103_page10_4.jpg]

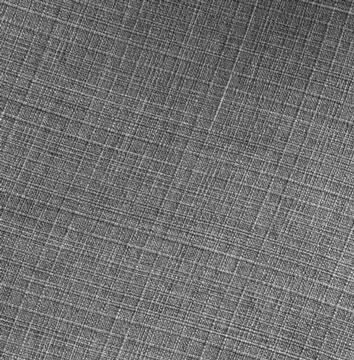

Supplement: Supplemental Information 4 [file peerj-cs-08-869-s004.zip › 1_part2/103_ven_0125.jpg]

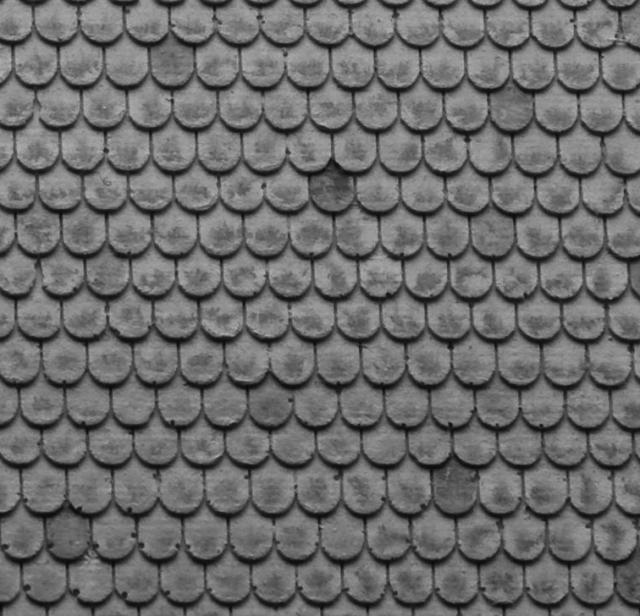

Supplement: Supplemental Information 4 [file peerj-cs-08-869-s004.zip › 1_part2/104_aly_0131.jpg]

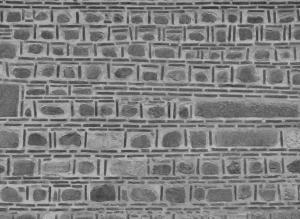

Supplement: Supplemental Information 4 [file peerj-cs-08-869-s004.zip › 1_part2/104_stone_wall_68.jpg]

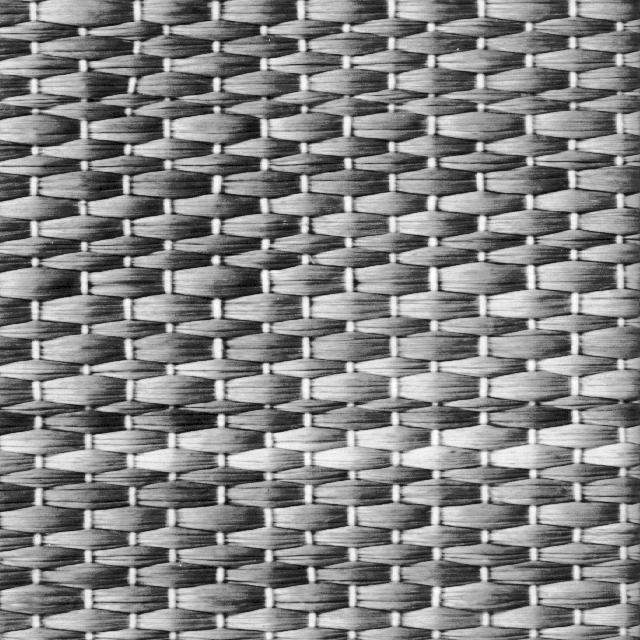

Supplement: Supplemental Information 4 [file peerj-cs-08-869-s004.zip › 1_part2/105_D56.jpg]

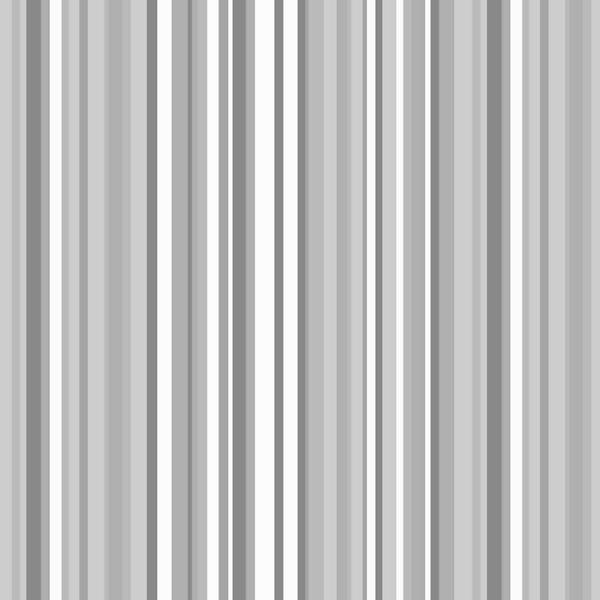

Supplement: Supplemental Information 4 [file peerj-cs-08-869-s004.zip › 1_part2/105_nded_0006.jpg]

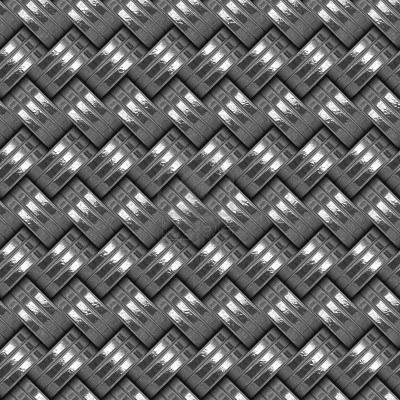

Supplement: Supplemental Information 4 [file peerj-cs-08-869-s004.zip › 1_part2/106_aided_0064.jpg]

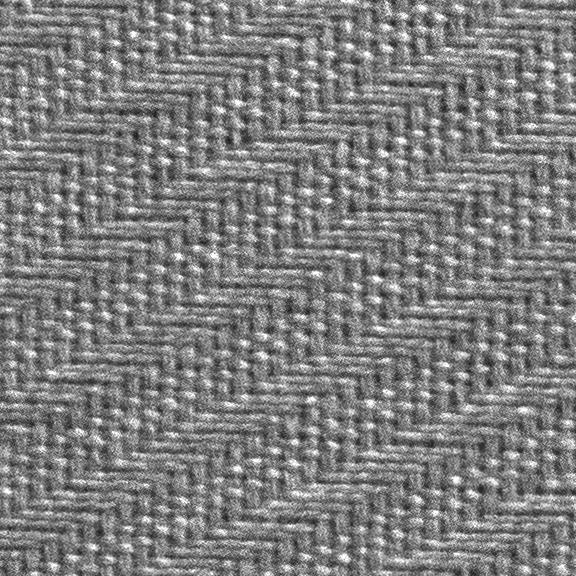

Supplement: Supplemental Information 4 [file peerj-cs-08-869-s004.zip › 1_part2/106_seat1-a-p002.jpg]

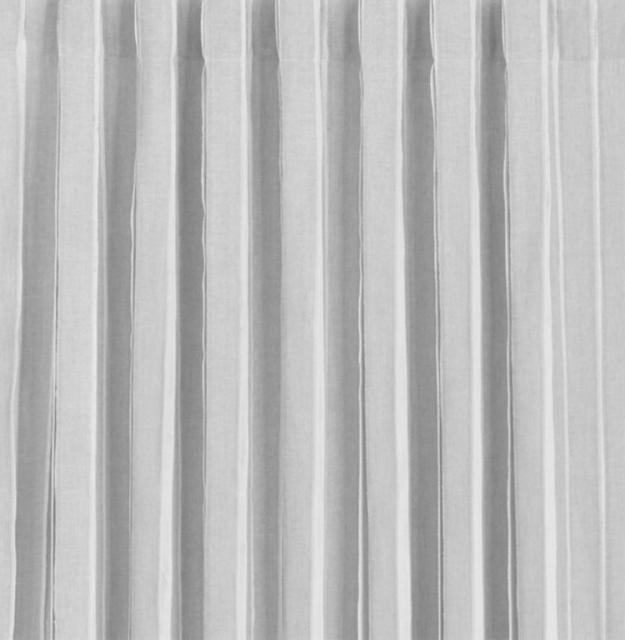

Supplement: Supplemental Information 4 [file peerj-cs-08-869-s004.zip › 1_part2/107_eated_0150.jpg]

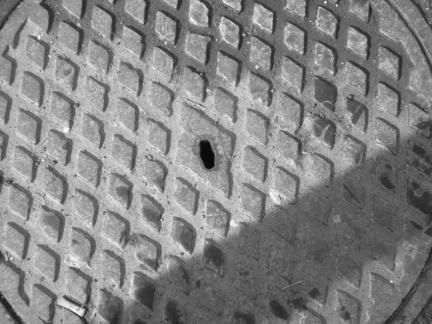

Supplement: Supplemental Information 4 [file peerj-cs-08-869-s004.zip › 1_part2/107_Normal nrt images 68_33.jpg]

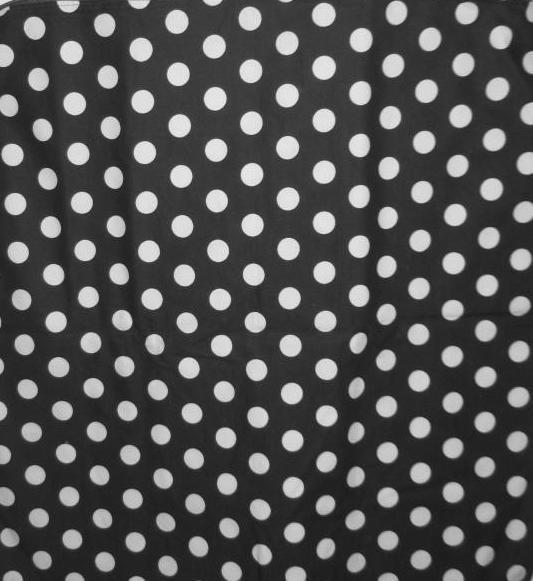

Supplement: Supplemental Information 4 [file peerj-cs-08-869-s004.zip › 1_part2/108_lka-dotted_0142.jpg]

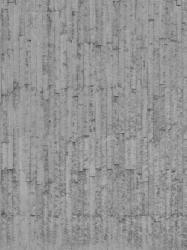

Supplement: Supplemental Information 4 [file peerj-cs-08-869-s004.zip › 1_part2/108_stone_wall_61.jpg]

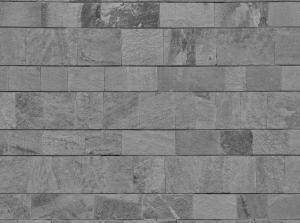

Supplement: Supplemental Information 4 [file peerj-cs-08-869-s004.zip › 1_part2/109_stone_wall_74.jpg]

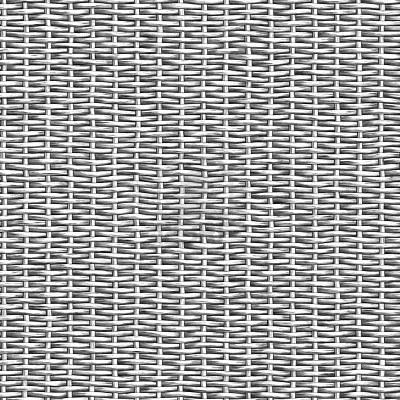

Supplement: Supplemental Information 4 [file peerj-cs-08-869-s004.zip › 1_part2/109_ven_0067.jpg]

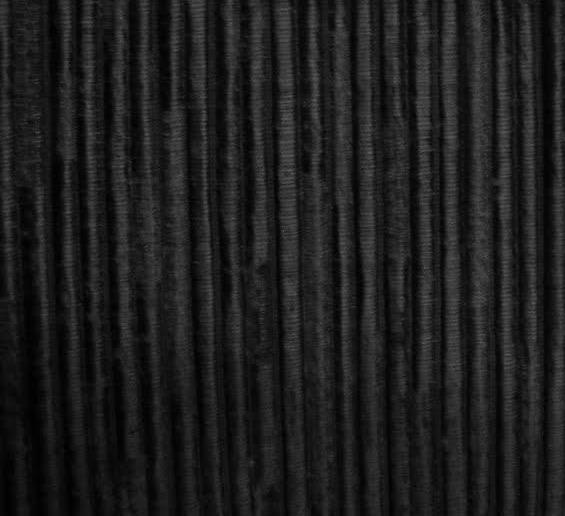

Supplement: Supplemental Information 4 [file peerj-cs-08-869-s004.zip › 1_part2/10_ooved_0111.jpg]

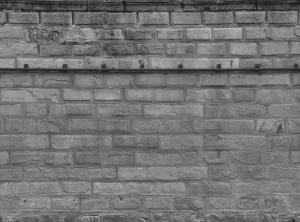

Supplement: Supplemental Information 4 [file peerj-cs-08-869-s004.zip › 1_part2/10_stone_wall_64.jpg]

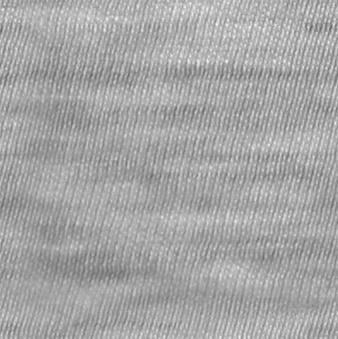

Supplement: Supplemental Information 4 [file peerj-cs-08-869-s004.zip › 1_part2/110_Cottn2_t.jpg]

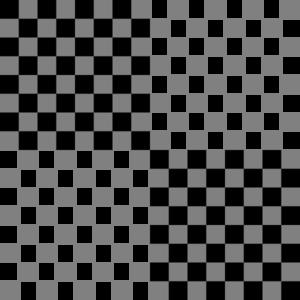

Supplement: Supplemental Information 4 [file peerj-cs-08-869-s004.zip › 1_part2/110_equered_0066.jpg]

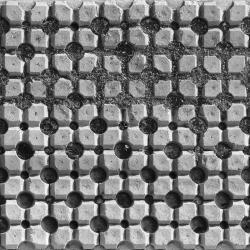

Supplement: Supplemental Information 4 [file peerj-cs-08-869-s004.zip › 1_part2/111_brick_pavement_84.jpg]

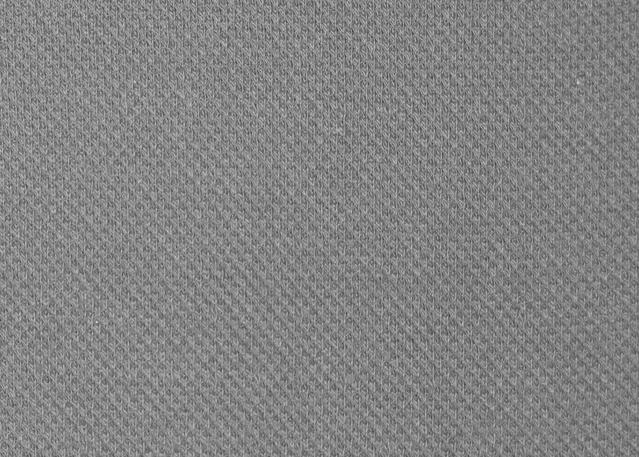

Supplement: Supplemental Information 4 [file peerj-cs-08-869-s004.zip › 1_part2/111_ven_0022.jpg]

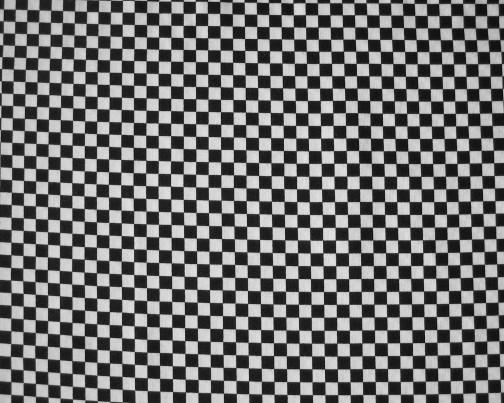

Supplement: Supplemental Information 4 [file peerj-cs-08-869-s004.zip › 1_part2/112_equered_0106.jpg]

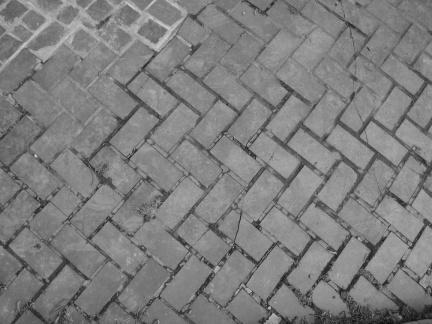

Supplement: Supplemental Information 4 [file peerj-cs-08-869-s004.zip › 1_part2/112_Pure Texture 171_158.jpg]

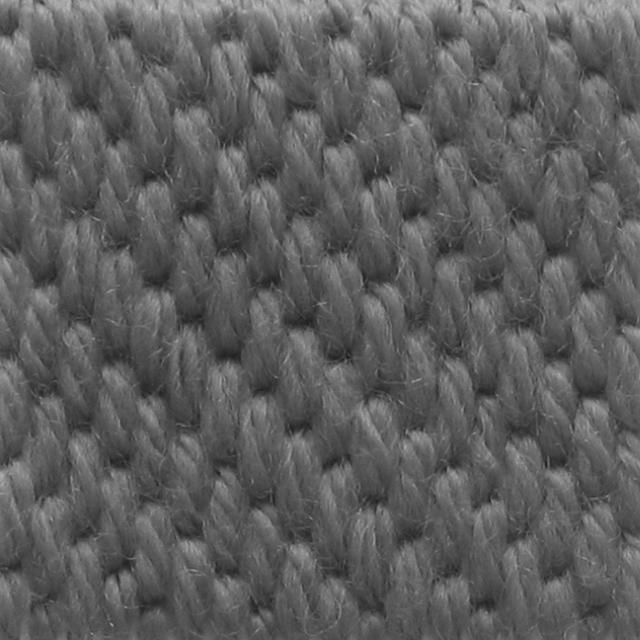

Supplement: Supplemental Information 4 [file peerj-cs-08-869-s004.zip › 1_part2/113_ven_0002.jpg]

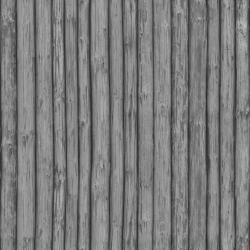

Supplement: Supplemental Information 4 [file peerj-cs-08-869-s004.zip › 1_part2/113_wood_planks_new_0004_04_thumb.jpg]

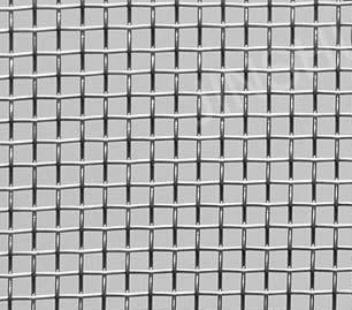

Supplement: Supplemental Information 4 [file peerj-cs-08-869-s004.zip › 1_part2/114_shed_0105.jpg]

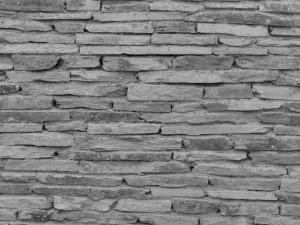

Supplement: Supplemental Information 4 [file peerj-cs-08-869-s004.zip › 1_part2/114_stone_wall_71.jpg]

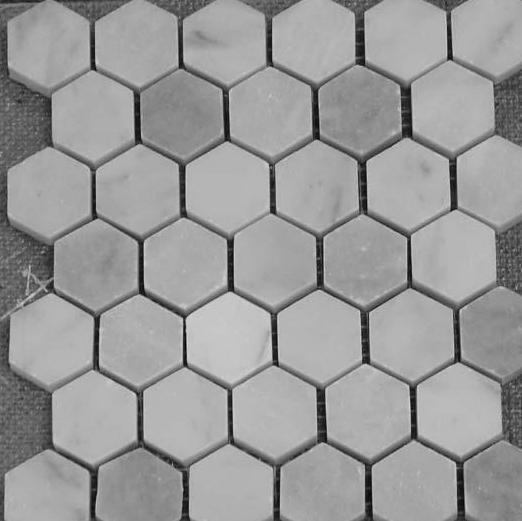

Supplement: Supplemental Information 4 [file peerj-cs-08-869-s004.zip › 1_part2/115_neycombed_0142.jpg]

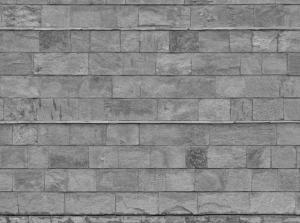

Supplement: Supplemental Information 4 [file peerj-cs-08-869-s004.zip › 1_part2/115_stone_wall_75.jpg]

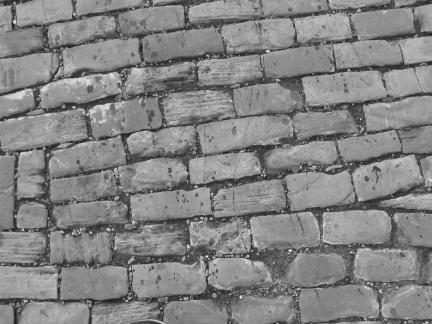

Supplement: Supplemental Information 4 [file peerj-cs-08-869-s004.zip › 1_part2/116_Borderline Near-Regular Textures 65_61.jpg]

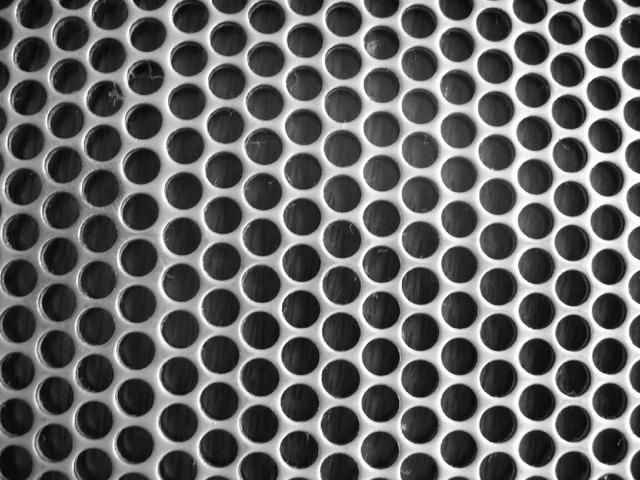

Supplement: Supplemental Information 4 [file peerj-cs-08-869-s004.zip › 1_part2/116_rforated_0074.jpg]

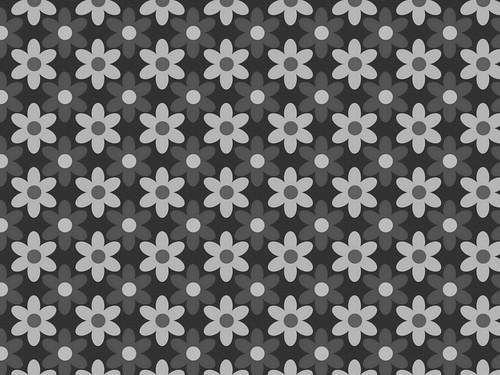

Supplement: Supplemental Information 4 [file peerj-cs-08-869-s004.zip › 1_part2/117_page11_5.jpg]

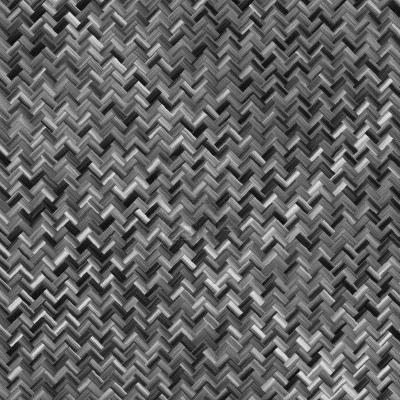

Supplement: Supplemental Information 4 [file peerj-cs-08-869-s004.zip › 1_part2/117_ven_0056.jpg]

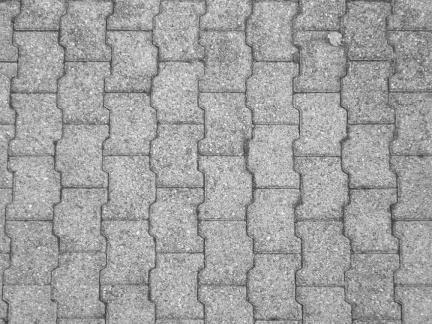

Supplement: Supplemental Information 4 [file peerj-cs-08-869-s004.zip › 1_part2/118_Pure Texture 171_34.jpg]

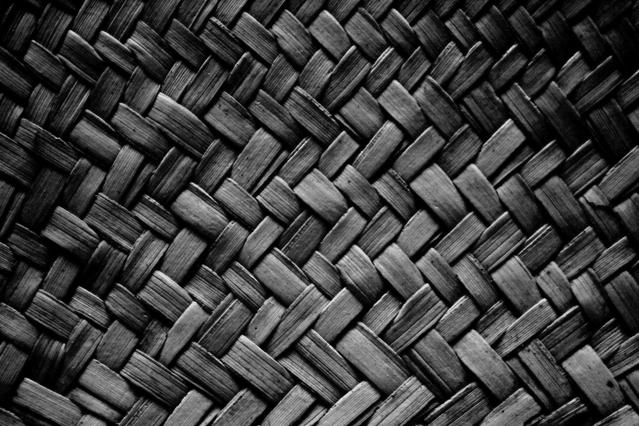

Supplement: Supplemental Information 4 [file peerj-cs-08-869-s004.zip › 1_part2/118_ven_0073.jpg]

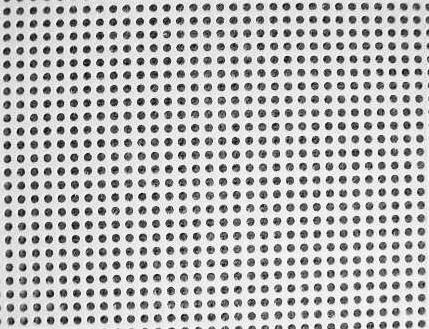

Supplement: Supplemental Information 4 [file peerj-cs-08-869-s004.zip › 1_part2/119_rforated_0026.jpg]

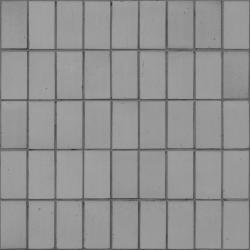

Supplement: Supplemental Information 4 [file peerj-cs-08-869-s004.zip › 1_part2/119_tile_tile_44.jpg]

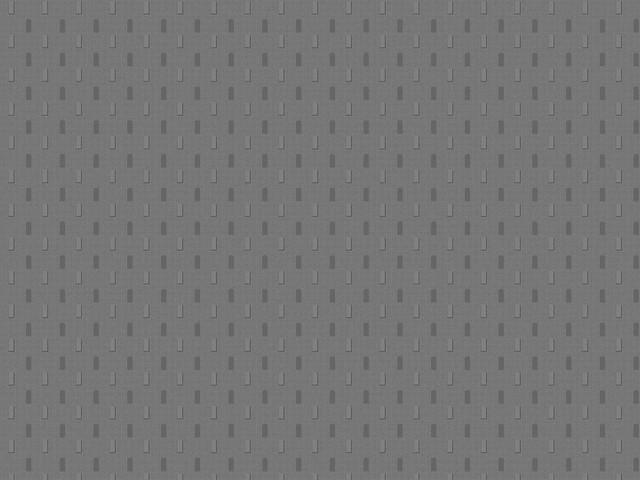

Supplement: Supplemental Information 4 [file peerj-cs-08-869-s004.zip › 1_part2/11_page13_13.jpg]

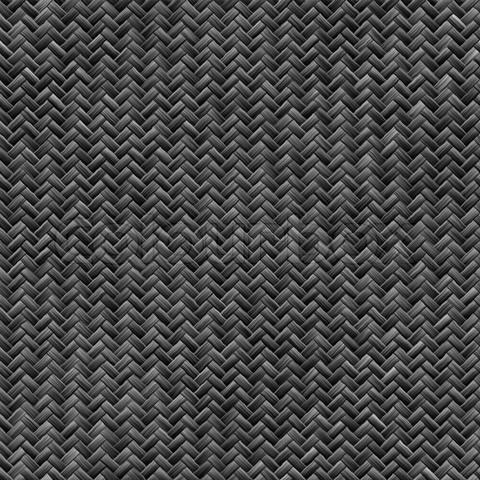

Supplement: Supplemental Information 4 [file peerj-cs-08-869-s004.zip › 1_part2/11_ven_0044.jpg]

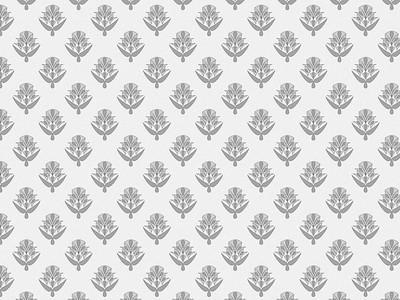

Supplement: Supplemental Information 4 [file peerj-cs-08-869-s004.zip › 1_part2/120_43215461150_558a5ef6c8_w.jpg]

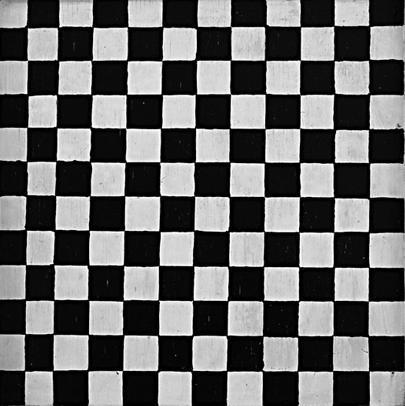

Supplement: Supplemental Information 4 [file peerj-cs-08-869-s004.zip › 1_part2/120_equered_0121.jpg]

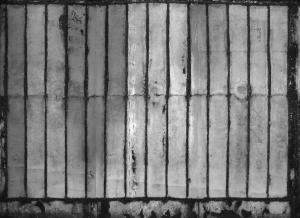

Supplement: Supplemental Information 4 [file peerj-cs-08-869-s004.zip › 1_part2/121_concrete massive_53.jpg]

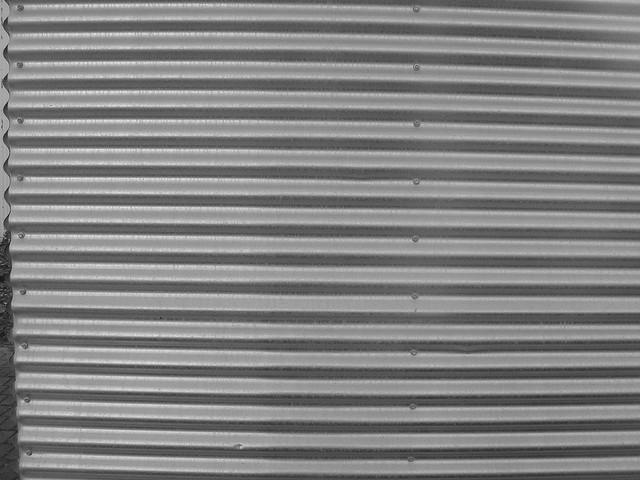

Supplement: Supplemental Information 4 [file peerj-cs-08-869-s004.zip › 1_part2/121_ooved_0063.jpg]

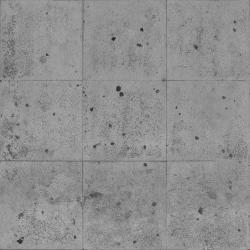

Supplement: Supplemental Information 4 [file peerj-cs-08-869-s004.zip › 1_part2/122_brick_pavement_18.jpg]

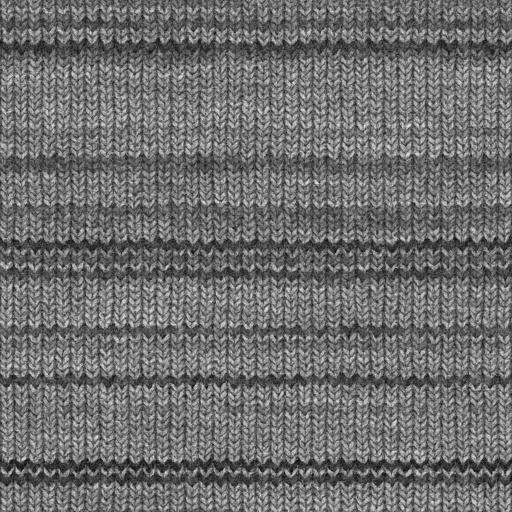

Supplement: Supplemental Information 4 [file peerj-cs-08-869-s004.zip › 1_part2/122_itted_0178.jpg]

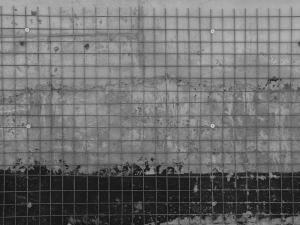

Supplement: Supplemental Information 4 [file peerj-cs-08-869-s004.zip › 1_part2/123_concrete other_26.jpg]

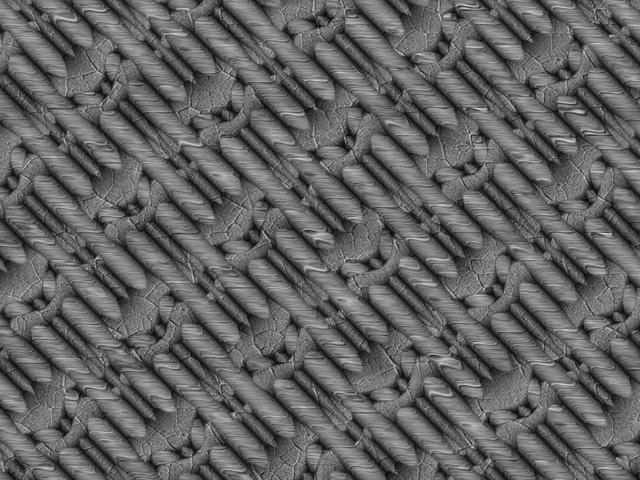

Supplement: Supplemental Information 4 [file peerj-cs-08-869-s004.zip › 1_part2/123_ven_0069.jpg]

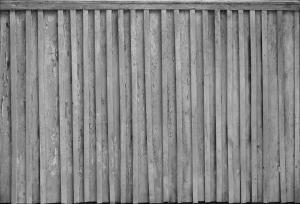

Supplement: Supplemental Information 4 [file peerj-cs-08-869-s004.zip › 1_part2/124_Planks old_96.jpg]

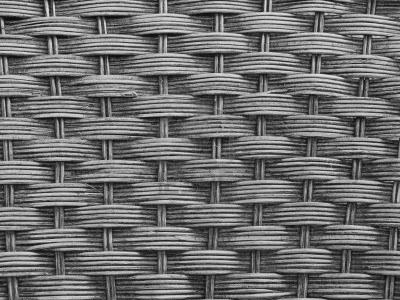

Supplement: Supplemental Information 4 [file peerj-cs-08-869-s004.zip › 1_part2/124_ven_0043.jpg]

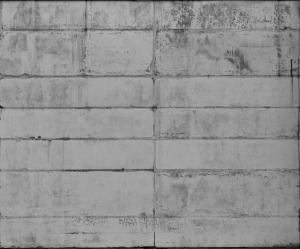

Supplement: Supplemental Information 4 [file peerj-cs-08-869-s004.zip › 1_part2/125_concrete massive_69.jpg]

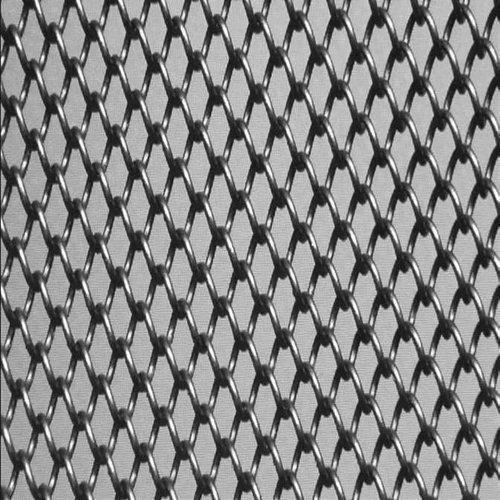

Supplement: Supplemental Information 4 [file peerj-cs-08-869-s004.zip › 1_part2/125_shed_0176.jpg]

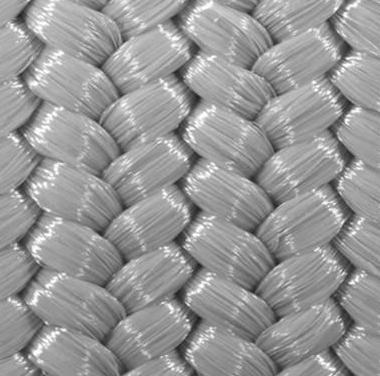

Supplement: Supplemental Information 4 [file peerj-cs-08-869-s004.zip › 1_part2/126_aided_0191.jpg]

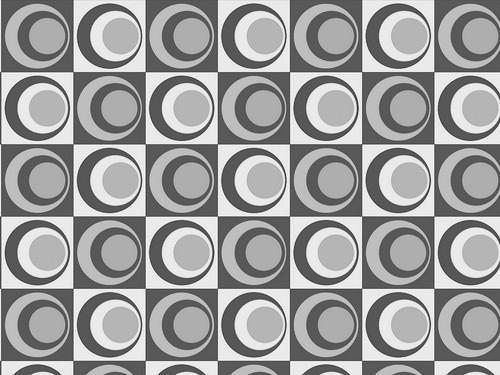

Supplement: Supplemental Information 4 [file peerj-cs-08-869-s004.zip › 1_part2/126_page8_18.jpg]

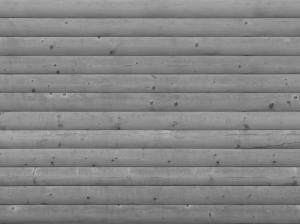

Supplement: Supplemental Information 4 [file peerj-cs-08-869-s004.zip › 1_part2/127_Planks old_61.jpg]

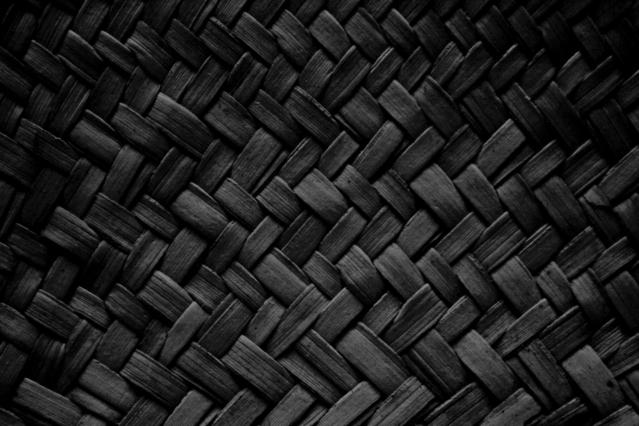

Supplement: Supplemental Information 4 [file peerj-cs-08-869-s004.zip › 1_part2/127_ven_0072.jpg]

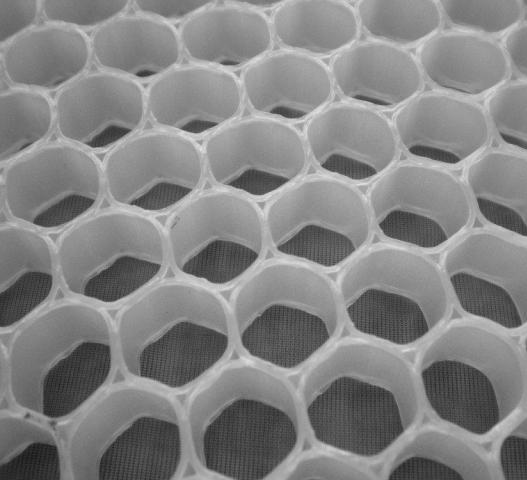

Supplement: Supplemental Information 4 [file peerj-cs-08-869-s004.zip › 1_part2/128_neycombed_0173.jpg]

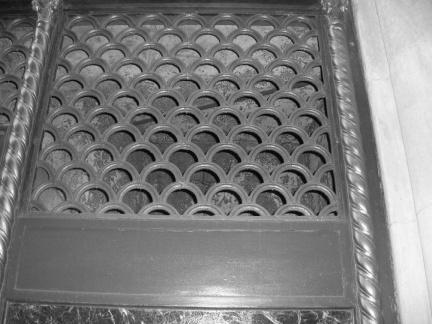

Supplement: Supplemental Information 4 [file peerj-cs-08-869-s004.zip › 1_part2/128_Pure Texture 171_36.jpg]

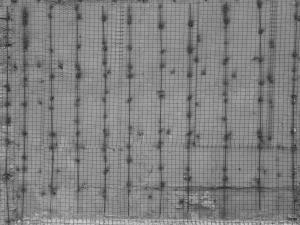

Supplement: Supplemental Information 4 [file peerj-cs-08-869-s004.zip › 1_part2/129_concrete other_30.jpg]

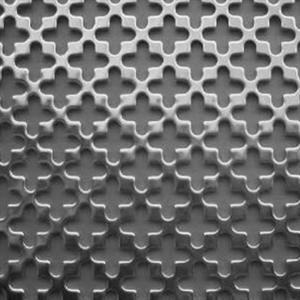

Supplement: Supplemental Information 4 [file peerj-cs-08-869-s004.zip › 1_part2/129_rforated_0079.jpg]

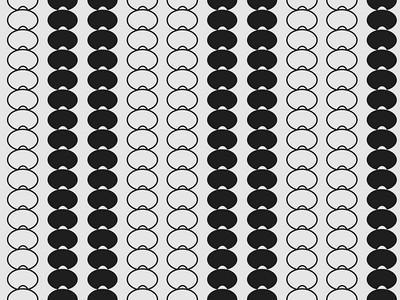

Supplement: Supplemental Information 4 [file peerj-cs-08-869-s004.zip › 1_part2/12_46726226585_e886a0442e_w.jpg]

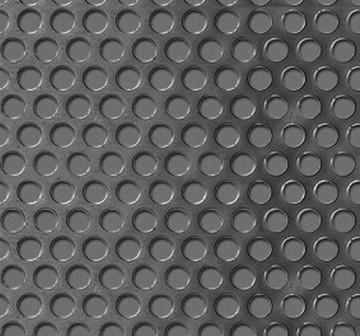

Supplement: Supplemental Information 4 [file peerj-cs-08-869-s004.zip › 1_part2/12_rforated_0041.jpg]

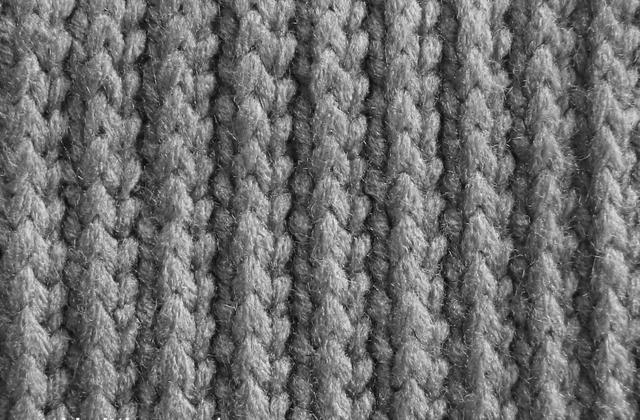

Supplement: Supplemental Information 4 [file peerj-cs-08-869-s004.zip › 1_part2/130_itted_0150.jpg]

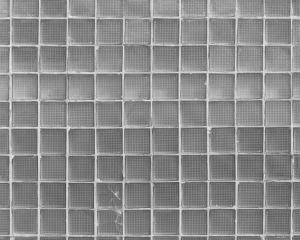

Supplement: Supplemental Information 4 [file peerj-cs-08-869-s004.zip › 1_part2/130_tile_tile_6.jpg]

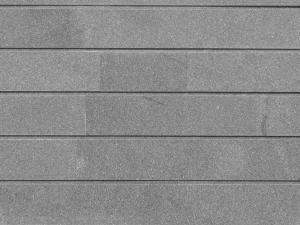

Supplement: Supplemental Information 4 [file peerj-cs-08-869-s004.zip › 1_part2/131_tile_tile_12.jpg]

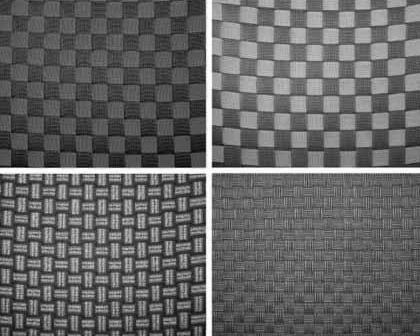

Supplement: Supplemental Information 4 [file peerj-cs-08-869-s004.zip › 1_part2/131_ven_0117.jpg]

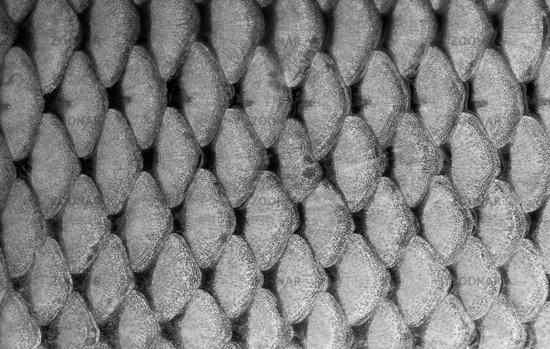

Supplement: Supplemental Information 4 [file peerj-cs-08-869-s004.zip › 1_part2/132_aly_0160.jpg]

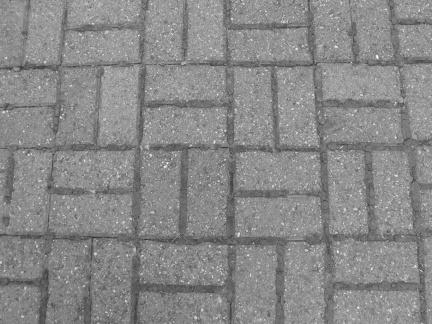

Supplement: Supplemental Information 4 [file peerj-cs-08-869-s004.zip › 1_part2/132_Pure Texture 171_107.jpg]

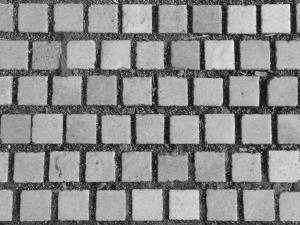

Supplement: Supplemental Information 4 [file peerj-cs-08-869-s004.zip › 1_part2/133_brick_pavement_90.jpg]

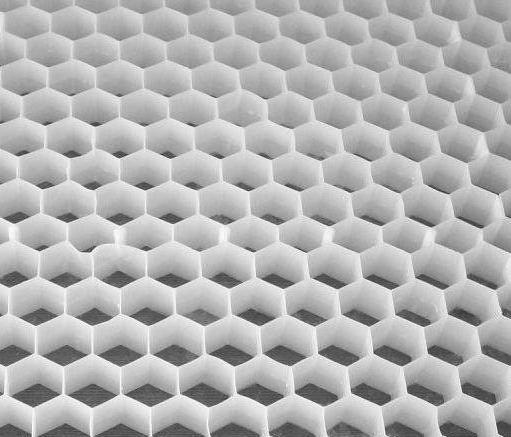

Supplement: Supplemental Information 4 [file peerj-cs-08-869-s004.zip › 1_part2/133_neycombed_0162.jpg]

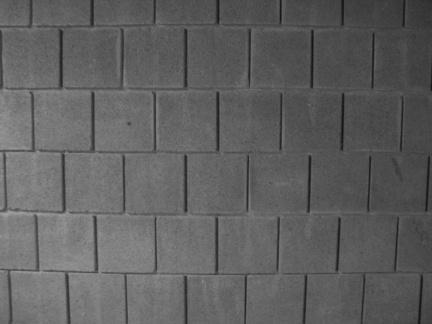

Supplement: Supplemental Information 4 [file peerj-cs-08-869-s004.zip › 1_part2/134_Pure Texture 171_140.jpg]

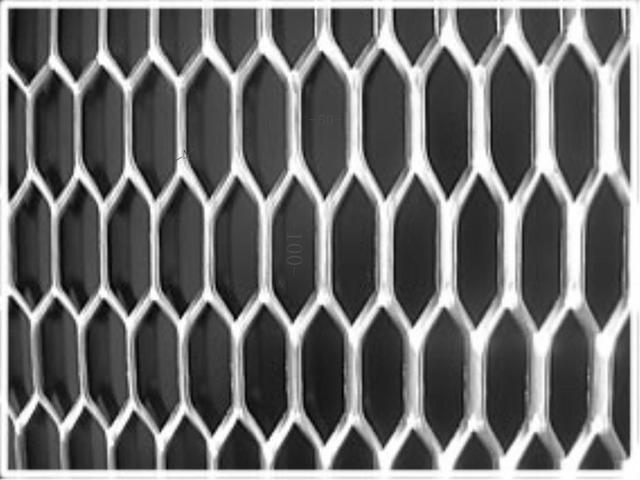

Supplement: Supplemental Information 4 [file peerj-cs-08-869-s004.zip › 1_part2/134_shed_0123.jpg]

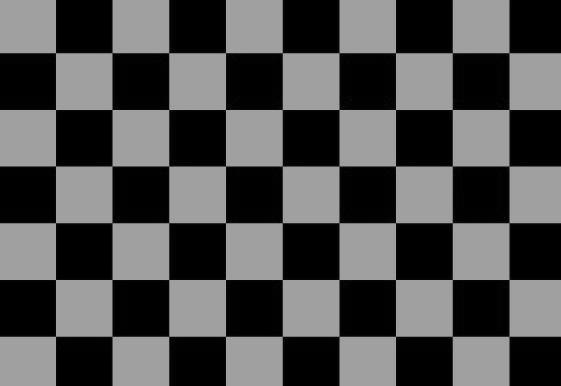

Supplement: Supplemental Information 4 [file peerj-cs-08-869-s004.zip › 1_part2/135_equered_0096.jpg]

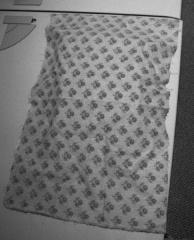

Supplement: Supplemental Information 4 [file peerj-cs-08-869-s004.zip › 1_part2/135_Normal nrt images 68_48.jpg]

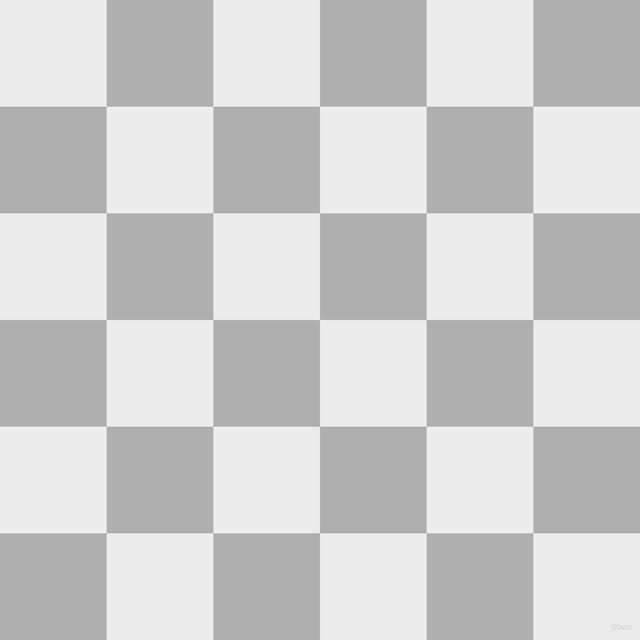

Supplement: Supplemental Information 4 [file peerj-cs-08-869-s004.zip › 1_part2/136_equered_0051.jpg]

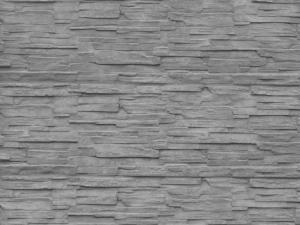

Supplement: Supplemental Information 4 [file peerj-cs-08-869-s004.zip › 1_part2/136_stone_wall_45.jpg]

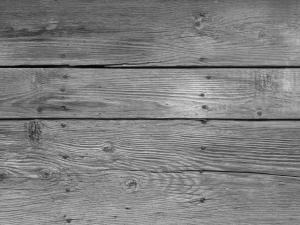

Supplement: Supplemental Information 4 [file peerj-cs-08-869-s004.zip › 1_part2/137_Planks old_7.jpg]

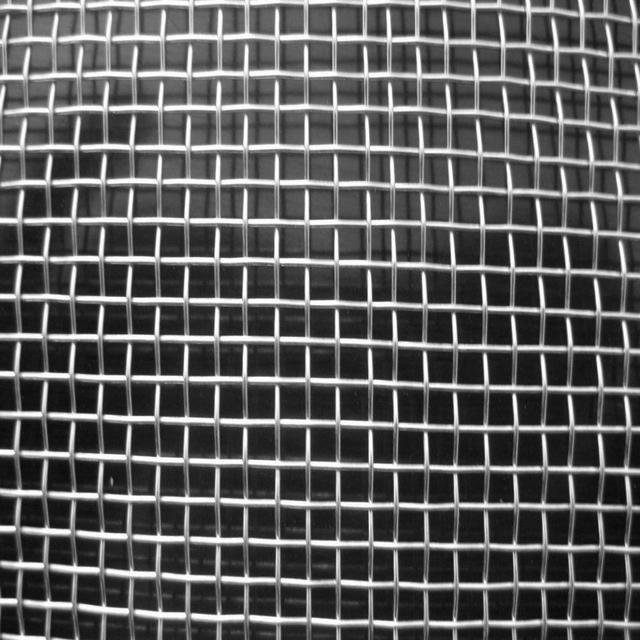

Supplement: Supplemental Information 4 [file peerj-cs-08-869-s004.zip › 1_part2/137_shed_0112.jpg]

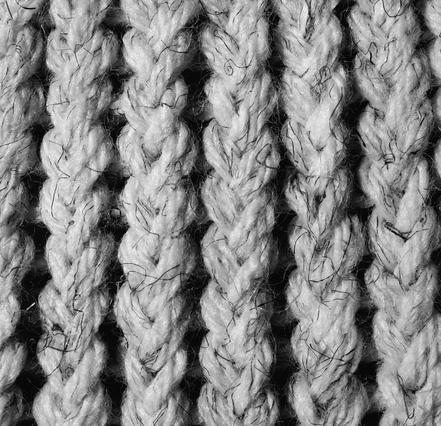

Supplement: Supplemental Information 4 [file peerj-cs-08-869-s004.zip › 1_part2/138_aided_0067.jpg]

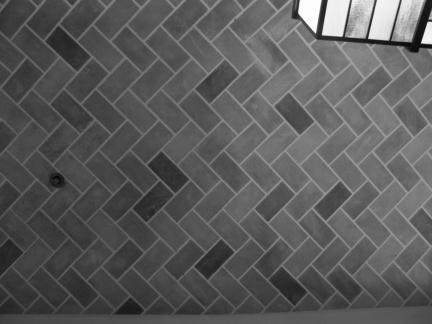

Supplement: Supplemental Information 4 [file peerj-cs-08-869-s004.zip › 1_part2/138_Pure Texture 171_169.jpg]

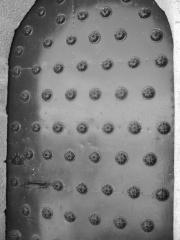

Supplement: Supplemental Information 4 [file peerj-cs-08-869-s004.zip › 1_part2/139_Normal nrt images 68_62.jpg]

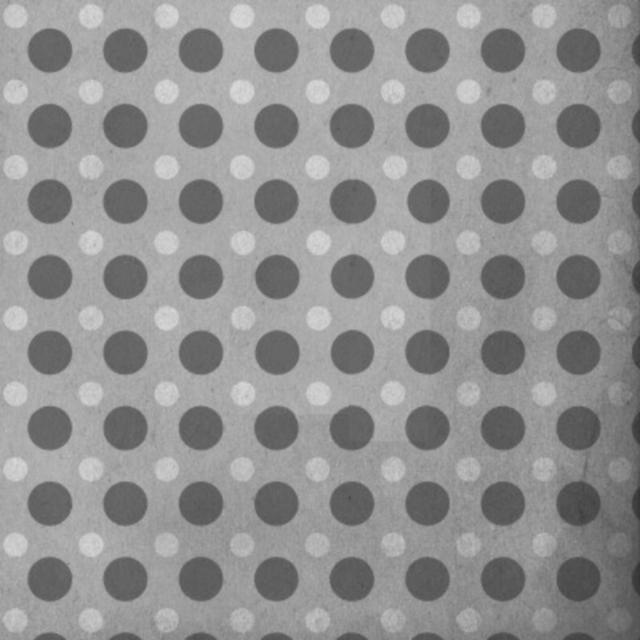

Supplement: Supplemental Information 4 [file peerj-cs-08-869-s004.zip › 1_part2/139_tted_0170.jpg]

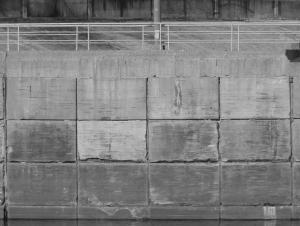

Supplement: Supplemental Information 4 [file peerj-cs-08-869-s004.zip › 1_part2/13_concrete massive_21.jpg]

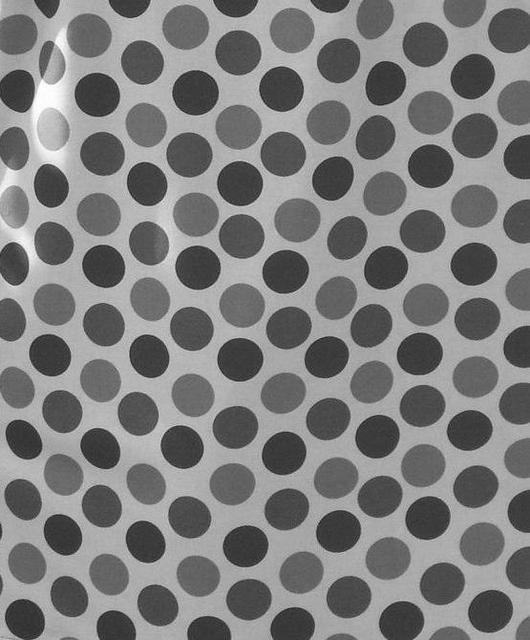

Supplement: Supplemental Information 4 [file peerj-cs-08-869-s004.zip › 1_part2/13_lka-dotted_0112.jpg]

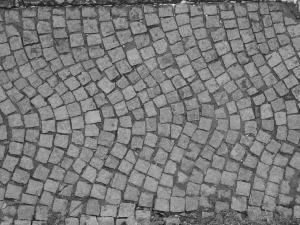

Supplement: Supplemental Information 4 [file peerj-cs-08-869-s004.zip › 1_part2/140_brick_pavement_98.jpg]

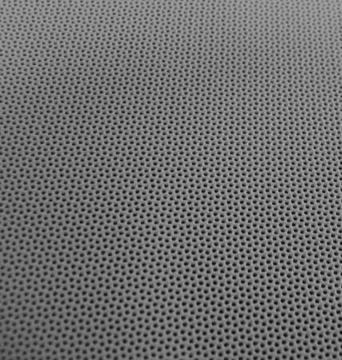

Supplement: Supplemental Information 4 [file peerj-cs-08-869-s004.zip › 1_part2/140_rforated_0043.jpg]

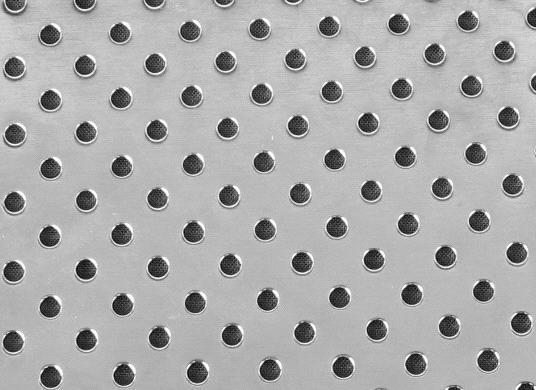

Supplement: Supplemental Information 4 [file peerj-cs-08-869-s004.zip › 1_part2/141_perforated_0076.jpg]

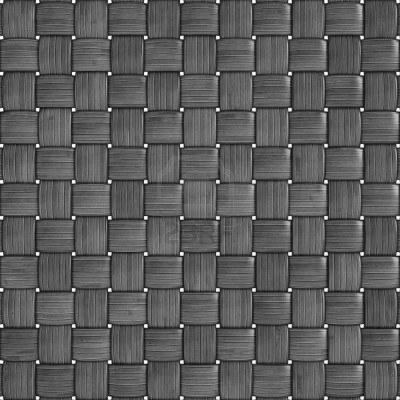

Supplement: Supplemental Information 4 [file peerj-cs-08-869-s004.zip › 1_part2/141_ven_0029.jpg]

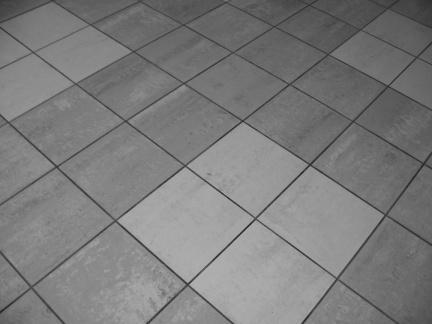

Supplement: Supplemental Information 4 [file peerj-cs-08-869-s004.zip › 1_part2/142_Pure Texture 171_19.jpg]

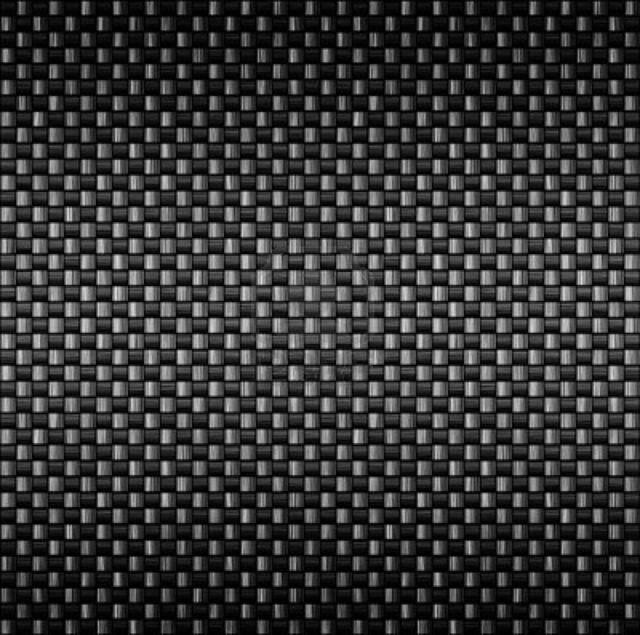

Supplement: Supplemental Information 4 [file peerj-cs-08-869-s004.zip › 1_part2/142_ven_0076.jpg]

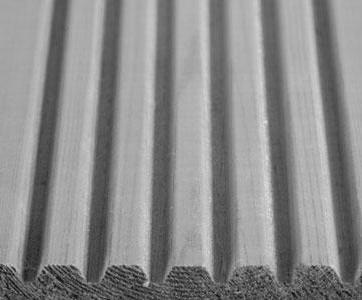

Supplement: Supplemental Information 4 [file peerj-cs-08-869-s004.zip › 1_part2/143_ooved_0113.jpg]

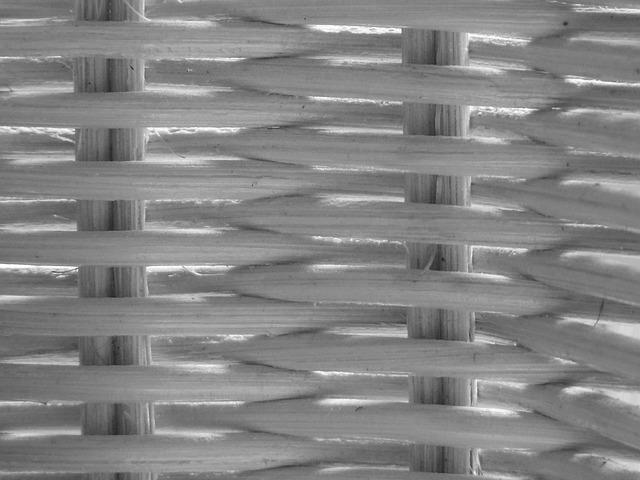

Supplement: Supplemental Information 4 [file peerj-cs-08-869-s004.zip › 1_part2/144_aided_0141.jpg]

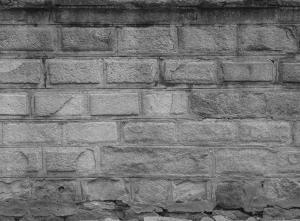

Supplement: Supplemental Information 4 [file peerj-cs-08-869-s004.zip › 1_part2/144_stone_wall_65.jpg]

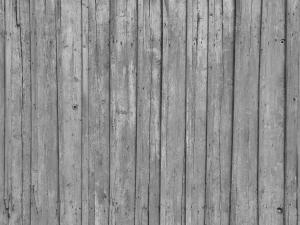

Supplement: Supplemental Information 4 [file peerj-cs-08-869-s004.zip › 1_part2/145_Planks old_87.jpg]

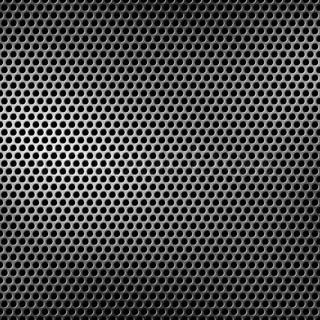

Supplement: Supplemental Information 4 [file peerj-cs-08-869-s004.zip › 1_part2/145_rforated_0037.jpg]

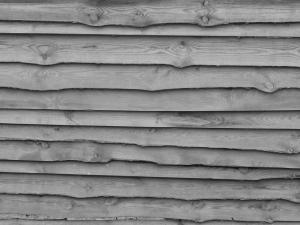

Supplement: Supplemental Information 4 [file peerj-cs-08-869-s004.zip › 1_part2/146_Planks old_74.jpg]

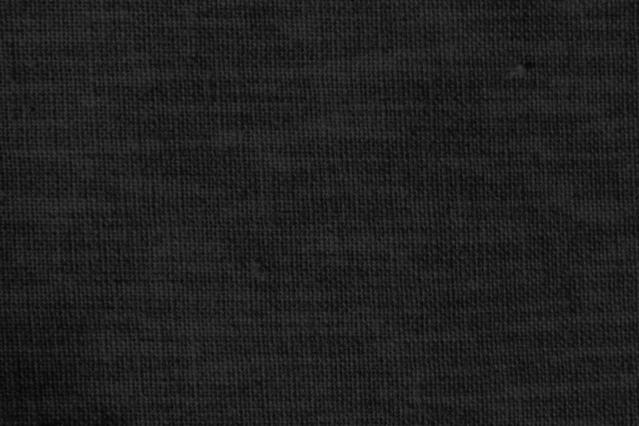

Supplement: Supplemental Information 4 [file peerj-cs-08-869-s004.zip › 1_part2/146_ven_0010.jpg]

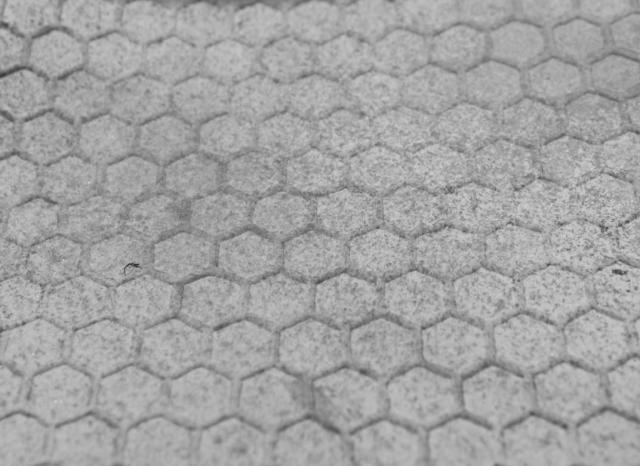

Supplement: Supplemental Information 4 [file peerj-cs-08-869-s004.zip › 1_part2/147_neycombed_0119.jpg]
